# One Rule to Grow Them All: A General Theory of Neuronal Branching and Its Practical Application

**DOI:** 10.1371/journal.pcbi.1000877

**Published:** 2010-08-05

**Authors:** Hermann Cuntz, Friedrich Forstner, Alexander Borst, Michael Häusser

**Affiliations:** 1 Wolfson Institute for Biomedical Research and Department of Neuroscience, Physiology and Pharmacology, University College London, London, United Kingdom; 2 Department of Systems and Computational Neurobiology, Max-Planck Institute of Neurobiology, Martinsried, Germany; RIKEN Brain Science Institute, Japan

## Abstract

Understanding the principles governing axonal and dendritic branching is essential for unravelling the functionality of single neurons and the way in which they connect. Nevertheless, no formalism has yet been described which can capture the general features of neuronal branching. Here we propose such a formalism, which is derived from the expression of dendritic arborizations as locally optimized graphs. Inspired by Ramón y Cajal's laws of conservation of cytoplasm and conduction time in neural circuitry, we show that this graphical representation can be used to optimize these variables. This approach allows us to generate synthetic branching geometries which replicate morphological features of any tested neuron. The essential structure of a neuronal tree is thereby captured by the density profile of its spanning field and by a single parameter, a balancing factor weighing the costs for material and conduction time. This balancing factor determines a neuron's electrotonic compartmentalization. Additions to this rule, when required in the construction process, can be directly attributed to developmental processes or a neuron's computational role within its neural circuit. The simulations presented here are implemented in an open-source software package, the “TREES toolbox,” which provides a general set of tools for analyzing, manipulating, and generating dendritic structure, including a tool to create synthetic members of any particular cell group and an approach for a model-based supervised automatic morphological reconstruction from fluorescent image stacks. These approaches provide new insights into the constraints governing dendritic architectures. They also provide a novel framework for modelling and analyzing neuronal branching structures and for constructing realistic synthetic neural networks.

## Author Summary

More than a century has passed since Ramón y Cajal presented a set of fundamental biological laws of neuronal branching. He described how the shape of the core elements of the neural circuitry – axons and dendrites – are constrained by physical parameters such as space, cytoplasmic volume, and conduction time. The existence of these laws enabled him to organize his histological observations, to formulate the neuron doctrine, and to infer directionality in signal flow in the nervous system. We show that Cajal's principles can be used computationally to generate synthetic neural circuits. These principles rigorously constrain the shape of real neuronal structures, providing direct validation of his theories. At the same time, this strategy provides us with a powerful set of tools for generating synthetic neurons, as well as a model-based approach for automated reconstructions of neuronal trees from confocal image stacks.

## Introduction

Neuronal circuits are composed of a large variety of branched structures – axons and dendrites – forming a highly entangled web, reminiscent of a stochastic fractal [1]. Despite this apparent chaos, more than a century ago Ramón y Cajal was able to extract order from this neuroanatomical complexity, formulating fundamental anatomical principles of nerve cell organization [2]. Cajal described three biological laws of neuronal architecture (Chapter V, p.115–125, in [2]): optimization principles for conservation of space, cytoplasm and conduction time in the neural circuitry. These principles helped him to classify his observations and allowed him to postulate a wide variety of theories of functionality and directionality of signal flow in various brain areas. In the meantime, many of these ideas have been substantiated by applying more rigorous statistical analysis: circuitry and connectivity considerations as well as simple wire-packing constraints have been shown to determine the statistics of dendritic morphology [3]–[5]. It has also been shown mathematically that the specific organization and architecture of many parts of the brain reflect the selection pressure to reduce wiring costs for the circuitry [6]–[9].

In parallel, the development of compartmental modelling techniques based on the theories of Wilfrid Rall have highlighted the importance of a neuron's precise branching morphology for its electrophysiological properties [10], and have shown that dendrites can play an important role in the computations performed on the inputs to the cell [11], [12]. In fact, requirements for highly selective connectivity [13], [14], coherent topographic mapping, sophisticated computation or signal compartmentalization at the level of the single cell [15] and the network could all contribute to this observed intricacy of brain wiring.

These two lines of investigation raise the question as to whether computation plays the determining role in shaping the morphological appearance of neuronal branching structures. Alternatively, the simple laws of material and conduction time preservation of Ramón y Cajal could have more influence.

Using computational techniques it has become possible to construct synthetic neuronal structures *in silico* governed by the simulation of physical and biological constraints [1], [16]–[21]. In two recent papers [19], [20], we derived a growth algorithm for building dendritic arborisations following closely the constraints previously described by Ramón y Cajal. The algorithm builds tree structures which minimize the total amount of wiring and the path from the root to all points on the tree, corresponding to material and conduction time conservation respectively. Synthetic insect dendrite morphologies were faithfully reproduced in terms of their visual appearance and their branching parameters in this way.

Here we explore the algorithm's general applicability and its potential to describe any type of dendritic branching. If the algorithm is sufficient to accurately mimic the essential structure of neuronal circuitry we can resolve the relative importance of computation and wiring constraints in shaping neuronal morphology. We can then claim that Ramón y Cajal's laws are sufficient for shaping neuronal morphology. Specific computation will then only play a subordinate role in determining a neuron's branching pattern. We show here that while Cajal's laws do represent a strict constraint on neuronal branching, a neuronal morphology has a certain freedom to operate within these constraints. Firstly, by adjusting the balance between the two wiring costs, a dendrite can efficiently set its electrotonic compartmentalization, a quantity attributable to computation. Secondly, the density profile of the spanning field in which a dendrite grows determines its shape dramatically. Thirdly, a few weaker constraints such as the suppression of multifurcations, the addition of spatial jitter or the sequential growth of sub-regions of a dendrite are helpful for reproducing the dendritic branching patterns of particular preparations. These additional constraints might shed light on further functional, computational, developmental or network determinants for certain dendritic structures, and more of these will follow when applying our method to many more preparations. Moreover, the simple principles presented in this study can be used to efficiently edit, visualize, and analyze neuronal trees. Finally, these approaches allow one to generate highly realistic synthetic branched structures, and to automatically reconstruct neuronal branching from microscopy image stacks.

## Results

### The neuronal tree as a graph

**Figure 1:**
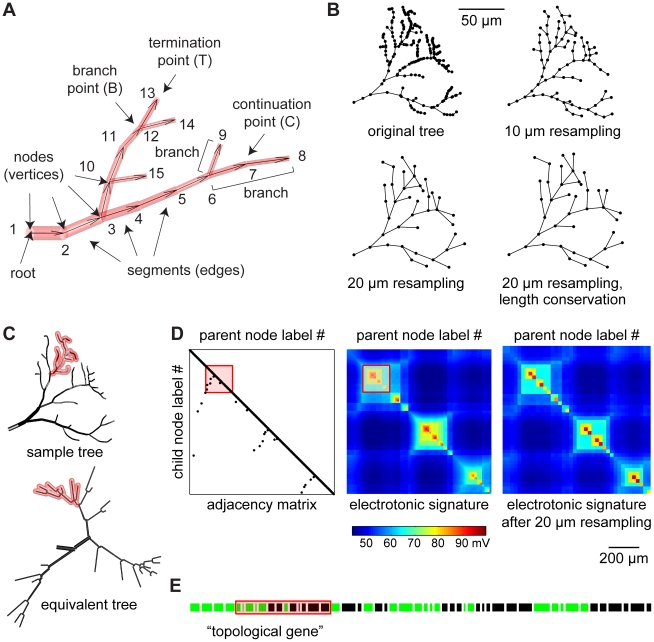
The topological and electrotonic identity of a neuronal tree. (A) The tree consists of cylinders or frusta (red) connecting each two nodes along the directed edges (away from the root node, arrows). Branch points and termination points represent the topology (topological points). A branch is a set of continuation points between two topological points. The labelling of the nodes is unique following three principles: hierarchical sorting, continuous labelling preserving sub-tree consistency and topological sorting (see text). (B) Rearrangement of node locations on a sample tree. Examples of equidistant node redistribution resulting in 10 or 20 µm resampling and a 20 µm resampling including length conservation (see text and “resampling” section of Methods). (C–E) Unique representations of topology and electrotonic properties from sample tree from (B). (C) Applying topological sorting, a unique electrotonic equivalent tree can be constructed by mapping node label hierarchy on the branch angle (equivalent tree). (D) The adjacency matrix depicts the connectivity between the nodes of a tree. The corresponding electrotonic signature (current transfer from a node to another, i.e. the potential difference measured in one node as a result of a current injection into another) describes the dendritic compartmentalization (see text). The electrotonic signature corresponding to the 20 µm resampled tree preserves the compartmentalization of the original tree. (E) A one-dimensional string fully describes the topology once the nodes of a tree are sorted topologically. Green pieces represent branches ending with a branch point while black pieces end with a termination point. Branch lengths correspond to real metric length and their order follows the node label sorting. Because all representations observe the same continuous labelling, they preserve the sub-tree structure (a red transparent patch highlights one such sub-tree throughout all representations in (C–E)).

Before generating complex neuronal morphologies, a simple formalism is required to compare and assess natural and synthetic neuronal trees. We derive such a formalism from graph theory: a neuronal tree is a graph which connects a set of labelled nodes via directed edges away from a root labelled “1” (Figure 1A). This distinct directionality is useful since properties describing a neuron's branching typically relate to the root of the tree, e.g. the branch order which increases after each branch point away from the root. In general, the graph describing a specific neuronal tree should be entirely unique in order to be used to compare two trees topologically (their branching properties) or electrotonically (their functional properties). To achieve this, a unique labelling of the nodes is required. We constrain labelling by imposing a hierarchical order (node label values increase with distance from the root), continuous labelling within sub-trees (see for example nodes “6”, “7”, “8” and “9”, which form a sub-tree, Figure 1A) and a topological sorting in which at any branch point the sub-tree with a higher topological depth is labelled first (see Figure 1A and “label sorting” section in Methods). Apart from requiring unique labelling, a unique representation of a tree requires its nodes to be precisely distributed along its geometry. The process of manual reconstruction assigns node locations in an arbitrary manner (Figure 1B, original tree). We introduce a process we term resampling, in which nodes are redistributed on the same tree structure, assigning homogeneous inter-nodal distances (Figure 1B shows 10 and 20 µm resampling; see “resampling” section in Methods).

When node labelling and distribution are attributed in a distinct manner, a unique representation of the tree is provided. By rearranging the node locations of a sample tree (Figure 1C, sample tree) based on its label order while preserving segment lengths, one obtains a unique electrotonic equivalent (Figure 1C, equivalent tree; see “Electrotonic equivalent” section in Methods). Dendritic structure can then also be described by a single unique adjacency matrix, which indicates for each node its direct parent node (Figure 1D, adjacency matrix). Consistent with the adjacency matrix, a matrix containing the current transfer from any node to any other in the tree is a good representation of its electrotonic properties (Figure 1D, electrotonic signature). As a result of the continuous node labelling within sub-trees, electrotonic compartmentalization expresses itself as square sub-regions with high reciprocal current transfer. Because of the unique graphical representation, the electrotonic signature is independent of the reconstruction procedure. Note that simplified tree structures, which preserve the electrotonic compartmentalization, can be obtained by a coarser resampling (Figure 1D, electrotonic signature of the same tree as in Figure 1B with 20 µm sampling). Computing current flow in a corresponding compartmental model is much faster since the number of nodes is decreased drastically (from 297 to 39 in the example of Figure 1D). Finally, a simple and unique one-dimensional string can be used to describe the topology of the tree entirely, which we term the “topological gene”. In this string each branch is described in the order of its node labels by its length value followed by a “B” if the branch ends in a branch point or by a “T” if it ends in a termination point. The “topological gene” can be displayed as a sequence of green (“B”) and black (“T”) pieces (Figure 1E). If diameter values for each node are known, the electrotonic signature can be reconstructed solely from this one-dimensional string, since segment lengths and topology are conserved.

### Optimized graphs for implementing Cajal's laws

**Figure 2:**
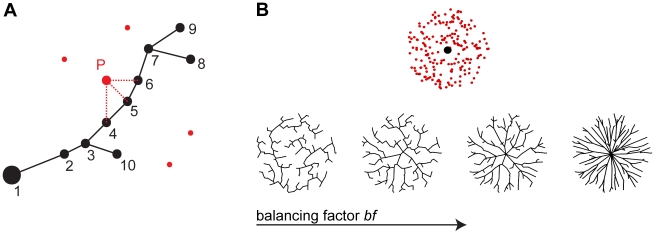
Generating neuronal branching structures using optimized graphs. (A) The growth described by an extended minimum spanning tree algorithm (see text). Unconnected carrier points (red) are connected one by one to the nodes of a tree (black). Red dashed lines indicate three sample Euclidean distances to the nodes of the tree for sample point *P*. (B) Example trees grown on homogeneously distributed random carrier points in a circular hull starting from a root located at its centre (see top). Plotted as a function of the balancing factor *bf*, the trees range from perfect minimum spanning trees (left) to almost direct connections from the root to any point (right).

In order to incorporate Cajal's hypotheses about wiring optimization in our theoretical description of a neuronal tree, we implemented optimization procedures known from graph theory. This approach was previously shown to be successful for generating synthetic dendritic structures of fly interneurons [19], [20] as well as recently for neocortical axons [22]. We now generalize it to more arbitrary neuronal geometries. Figure 2A exemplifies the general approach of assembling a set of unconnected carrier points to such an optimized graph. A greedy algorithm based on the minimum spanning tree algorithm [23] starts at the root with an empty tree and connects unconnected carrier points (red dots) one by one to the nodes of the tree (black dots). At each step, the unconnected carrier point which is the point closest to the tree according to a cost function connects to the node in the tree to which it is nearest. The distance cost is composed of two factors: 1) A wiring cost represented by the Euclidean distance between the carrier point and the node in the tree (red dashed lines show three sample segment distances for point *P*); this quantity loosely corresponds to the material conservation constraint by Cajal; 2) A path length cost of the path along the tree from the root (large black node) to the carrier point; this quantity is consistent with the conduction time conservation constraint by Cajal. In the example here, even though *P* is closer to node *5* in Euclidean terms, the additional cost of path length (adding node *5* on the path) might tip the balance in favour of node *4*. A balancing factor *bf*, which weighs these two costs against each other in the cost function (*total cost = wiring cost +bf* ***·*** *path length cost*), represents the one and only parameter of the model.

Figure 2B illustrates the approach for neuronal trees grown on homogeneously distributed random carrier points in a circular envelope when the root is located at its centre. Since the two constraints (minimizing wiring and minimizing path length to the root) are weighted according to the balancing factor *bf* determining the contribution of the second constraint, the synthetic trees range along the dimension of that parameter from a pure minimum spanning tree, which grows in a wide spiral, to a purely stellate architecture (Figure 2B, from left to right).

In the following, we will apply this method of creating optimized graphs to reproduce morphologies in various neuronal preparations. The main effort will be to obtain an adequate set of carrier points for the application of the algorithm; this will prove to depend strongly on the density profile of the spanning field in the respective geometries. When additional constraints will be required in generating neurons in specific brain areas, this will provide clues pointing to actual computational or functional features of neuronal morphology.

### A geometric approach for generating neuronal trees

**Figure 3:**
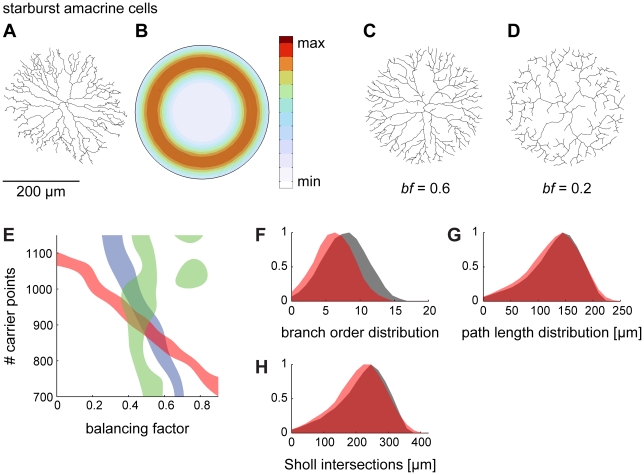
Generating dendritic structures by constructing geometric spanning fields: I. the retinal starburst amacrine cell. (A) Reconstruction of a starburst amacrine cell in the inner plexiform layer of the rabbit retina (data from [24]). (B) Synthetic starburst amacrine cell morphologies can be best obtained by distributing random carrier points along a density ring limited by a circular hull. (C) An example tree grown on random carrier points distributed according to B following the algorithm described in Figure 2. Spatial jitter was added to reproduce the wriggliness of the original structure. (D) A tree grown on exactly the same points as (C) with a lower balancing factor. (E) The number of randomly distributed carrier points and the balancing factor *bf* determine the synthetically generated morphology. Here, the areas are plotted in which the synthetic trees match the original according to certain criteria (blue: total cable length ±200 µm; red: total number of branch points ±5; green: mean path length to the root ±3 µm). The area of overlap corresponds to a reasonable parameter set for the synthetic trees. (F–H) Branch order distribution, path length distribution and Sholl intersections are compared for the original tree (red) and for one sample synthetic tree (grey).

Whereas our previous work was limited to insect dendrites [19], [20], here we explored whether the algorithm is also able to reproduce a variety of neuronal structures. We first investigated the simple case of a planar neuron: the starburst amacrine cell of the mammalian retina. Its root is invariably located at the centre of a circular planar structure (data from [24]; Figure 3A). This arrangement provides a common geometrical context for these cells. In order to best generate synthetic starburst amacrine cell-like neurites, random carrier points were distributed according to a ring-shaped density function around the centre in the root, limited by a simple circular hull (Figure 3B). The locus of increased density most likely corresponds to the area where an increased number of connections is being made in the real cell, with directional selectivity probably being computed there [25], [26]. Figure 3C demonstrates that this process successfully generates a synthetic neurite. The right balance between the two optimization constraints plays a crucial role, as is evident from a synthetic tree grown with a different balancing factor (*bf* = 0.2, Figure 3D). An appropriate balancing factor was determined by quantitatively comparing total cable length, mean path length to the root and number of branch points to the original real tree (Figure 3E). Using the corresponding balancing factor resulted in realistic distributions of branch order and path length values as well as a realistic Sholl plot [27], which counts the number of intersections of the tree with root-centred concentric spheres of increasing diameter values (Figure 3F–H). The starburst amacrine cell neurite required a higher *bf* than did the insect dendrites (0.6 versus 0.4, see [19], [20]). Additionally, suppressing multifurcations improved the growth process (compare Figure 3CD with Figure 2B). This was generally beneficial for all neurons studied here, and might reflect a constraint for the underlying developmental growth process. To better reproduce the appearance of reconstructions of real neurons, spatial jitter was added in all cases in the form of low-pass filtered spatial noise applied directly on the coordinates of the nodes in the resulting tree. Note that homogeneous noise application was only possible after the tree was resampled to a fixed segment length. Spatial noise in real reconstructions is partly due to fixation (e.g. shrinkage or reconstruction artefacts) and should therefore not necessarily be reproduced by the synthetic morphologies. However, wriggly paths in neuronal branching, corresponding to a spatial jitter along the branches, can be a result of obstacle avoidance and therefore can be associated with space packing issues [3], relating to the third law described by Ramón y Cajal. In this study, however, we do not model volumetric optimization or space packing of other neuronal and non-neuronal structures in the tissue. We thus simply note here that in order to fully reproduce starburst amacrine cell reconstructions, multifurcations were suppressed and spatial jitter was added.

**Figure 4:**
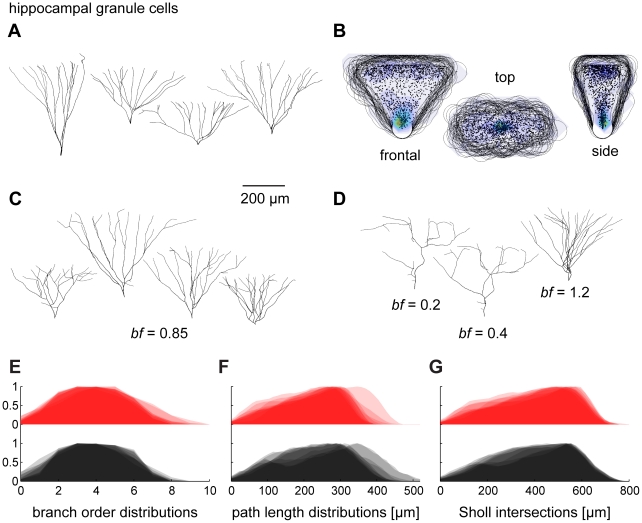
Generating dendritic structures by constructing geometric spanning fields: II. the hippocampal dentate gyrus granule cell. (A) Reconstructions of four sample hippocampal granule cells (data from [28]). (B) After centring, rotating and scaling all cells adequately, the 50 µm iso-distance volume hulls (black lines) around the set of all topological points (black dots) overlap in all dimensions. Left, xy-projection; Middle, xz-projection; Right; yz-projection. Overlay colours represent local density with same colormap as in Figure 3. (C) Examples of synthetically generated granule cells (based on the data in AB) with *bf* = 0.85. (D) Third cell from the left in C was grown on the same carrier points with different balancing factors to show the effect of *bf* here. (E–G) Overlaid branch order distributions, path length distributions and Sholl intersections for original trees (red) and for synthetic trees with suitable parameter *bf* = 0.85 (black).

We next studied dendrites of hippocampal granule cells, which fill a three-dimensional volume rather than a plane (template data from [28]; see sample cells in Figure 4A). We first centred the original cell reconstructions on the soma location and rotated them manually in all three dimensions to produce axial symmetry with respect to each axis. Then, the dendrites were scaled to the average limits over the population of real morphologies for each of the three dimensions. Surprisingly, the spanning fields overlapped delineating again a common context for all cells (Figure 4B). A geometric approach to describing the envelope of the dendrites is to intersect an elliptical cone with a sphere whose centre lies outside beyond the tip of the cone along the cone's central axis. The density profile of topological points (branch and termination points only) seemed to increase close to the origin of the volume and again further out at the rim (Figure 4B). Growing synthetic trees on random carrier points distributed according to this type of density profile within the constructed cone-like volume resulted in realistic dendritic structures (see examples in Figure 4C). Again, altering the balancing factor resulted in significant changes in branching behaviour (Figure 4D). This could be used to determine an appropriate balancing factor, which was higher for these cells. For comparison, full distributions of branching statistics are shown for synthetic granule cells and real counterparts in Figure 4E–G in analogy to Figure 3F–H. The two cases indicate that adequately balancing the costs for wiring with the costs for path length distances to the root is crucial to describing the dendritic morphology.

### A generalized approach for generating neuronal trees

**Figure 5:**
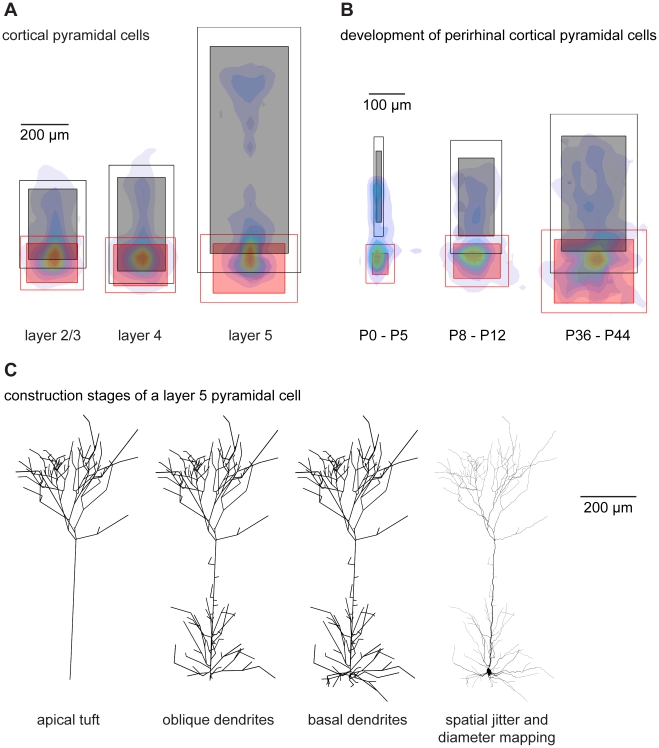
A general strategy for generating synthetic morphologies: Cortical pyramidal cells. (A) After rotating rat somatosensory cortex layer 2/3, 4 and 5 pyramidal cells to overlap, the limits of their individual regions were extracted: black shaded boxes show the mean limits in XY for the apical region; the black empty boxes delineate one standard deviation away from the mean. Corresponding red boxes duplicate this procedure for the basal dendrites. Cells are then scaled region-by-region to the mean limits of each region. Overlay colours describe local density (colormap see Figure 2D) of lumped topological points of scaled trees. (B) Same procedure for three groups of cortical pyramidal cells during development. (C) Construction stages of a sample layer 5 pyramidal cell according to spanning fields described in A. First the apical tuft is constructed, then oblique dendrites and finally the basal dendrite. Spatial jitter and diameter values are added subsequently.

The two cases described in Figures 3 and 4 used a geometrical construction to produce the density profile from which the carrier points were obtained, i.e. a ring-like density confined by a circle or a bimodal density profile within a volume obtained by intersecting a cone with a sphere. We next tried to generalize the approach taken with hippocampal granule cells to all neuron types. We derived context and spanning fields for a wide variety of existing cell types using a common feature that we observed in hippocampal granule cells: their scalability. This scalability is consistent with the fact that neuronal trees can be described as fractal-like structures in terms of their resolution (or complexity) within the field in which they span rather than by their real dimensions (see [1]). Based on the assumption that the principles of scalability hold true, we applied the following procedure on various dendritic structures: after all dendritic trees were centred around their somata, they were rotated manually such as to maximize the dendritic overlap. This was straightforward for cortical pyramidal cells and hippocampal granule cells where the main axis is obvious. Others, such as hippocampal pyramidal cells which will be discussed briefly further below, did not overlap since their precise branching contours depended greatly on the context and the location within the neuronal circuit. After rotating the cells into a common context, the limits of the spanning fields were measured separately for each region of the neuronal branching structure (apical and basal dendrite for example). In Figure 5A such limits are shown for apical and basal dendrites of layer 2/3, layer 4 and layer 5 pyramidal cells of the developing somatosensory cortex (data from [29]). The coordinates of all nodes belonging to an individual region were scaled to the mean limits of that region within each group of cells. This resulted in size-normalized cells. Surprisingly, the scaling of the different individual region limits did not typically correlate with each other, i.e. a large apical dendrite did not necessarily mean a large basal dendrite. After scaling, the topological points belonging to one specific region could all be lumped together and a bounded density cloud was calculated (exemplary density clouds for the cells as a whole are overlaid in Figure 5A). This procedure can be applied not only to different types of cells but also to different developmental stages of one particular cell type (data from perirhinal cortical pyramidal cells [30]; see Figure 3B). Carrier points were then distributed randomly according to the density distributions of each region one by one and connected by the growth algorithm. The number of carrier points used was increased for each synthetic tree until it matched a target branch point number picked from the distribution observed in the real cells. In pyramidal cells, it was necessary to split the apical dendrite in a tuft region and a lower oblique dendrite region, hinting possibly to a functional or developmental requirement. The tuft was grown first and resampled to 5 µm segments to provide attaching points along the main branch for the lower part of the apical dendrite to grow on. The basal dendrite was grown separately and the resulting cells subsequently subjected to spatial jitter, soma diameter mapping and dendritic diameter tapering (see Figure 5C and “Extended minimum spanning tree” section of Methods as well as Protocol S1 for more details).

**Figure 6:**
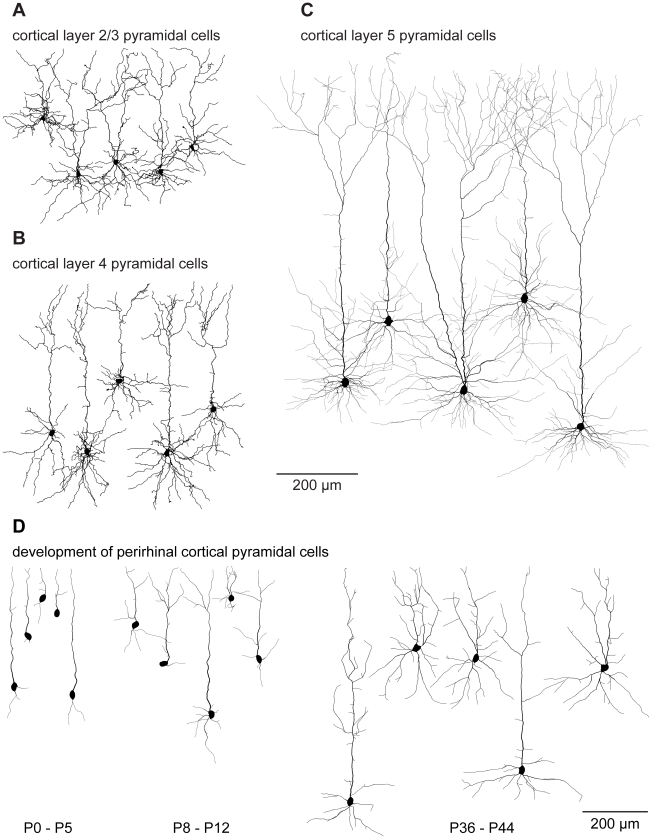
Sample cells grown using the general strategy. (A), (B) and (C) show sample synthetically generated model cells of layer 2/3, layer 4 and layer 5 cortical pyramidal cells respectively, all grown using the general strategy described in Figure 5. In A and B, all dendrites were thickened by 1 µm over all cells for clarity purposes. (D) When data from developing neurons was binned into three groups (P0–5, P8–12, P36–44), synthetically generated cortical pyramidal cells could be generated for the different developmental stages. Vertical or horizontal locations of the cells are purely for layout purposes in all cases.

Using this approach, dendritic morphologies of different pyramidal cells (Figure 6A–C: layer 2/3, layer 4 and layer 5, all *bf* = 0.7) were generated based on the spanning fields of Figure 5A. In this case, a wide range of suitable balancing factors (*bf* = 0.4–0.7) matched the distributions of total cable length and average path lengths in the resulting synthetic morphologies leading to realistic branching statistics (see below). In contrast to obtaining the density profiles for distributing random carrier points by a geometrical construction, these synthetic dendrite morphologies were obtained directly from scaled density plots from the real reconstructions. This increases the parameter space describing the generation of synthetic cells considerably. With this additional restriction, we obtain synthetic cells that are indistinguishable by eye from their real counterparts. The same approach can be used to generate pyramidal cells at different developmental stages (Figure 6D *bf* = 0.7, from the spanning fields of Figure 5B). We note that apart from the different spanning fields shown in Figure 5AB, different diameters and spatial jitter, all pyramidal cell clones were constructed according to the exact same procedure. In conclusion, Cajal's laws impose a general constraint on dendritic branching in all preparations and at all developmental stages we have investigated.

**Figure 7:**
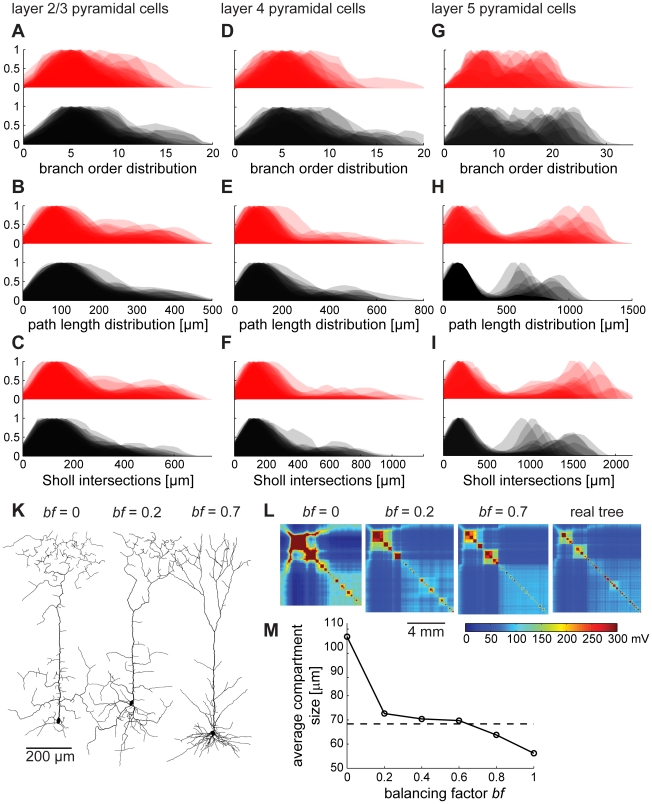
Validating synthetic branching structures of pyramidal cells. While branching statistics of starburst amacrine cells and hippocampal granule cells were moderately homogeneous, pyramidal cells exhibited stronger variations. Balancing factors leading to reasonable branching statistics ranged from 0.4 to 0.7. In the following we compare branching parameter distributions as in Figure 4 for synthetic (black) and original dendrites (red) of layer 2/3 (A–C), layer 4 (D–F) and layer 5 (G–I) pyramidal cells grown with a balancing factor *bf* = 0.7, 0.6 and 0.5 respectively. (K) Representative layer 5 pyramidal cells grown with different balancing factors *bf* = 0, *bf* = 0.2, *bf* = 0.7. (L) Representative electrotonic signatures of these synthetically generated dendrites and of one original layer 5 pyramidal cell for comparison. (M) Simple relationship between electrotonic compartmentalization and balancing factor. Straight line connects averages of each 100 model dendrites at different balancing factor values. Dashed line shows average compartment size of real reconstructions.

Distributions of branching parameters to compare the synthetic pyramidal cells with their real counterparts are shown in Figure 7A–I. As mentioned above, because of a higher variability between pyramidal cells than for example hippocampal granule cells, branching statistics lose their informative value. Note however, that, as observed previously for insect dendrites [20], path length distributions are similar to Sholl intersections for both real and synthetic geometries, which is a direct consequence of minimizing conduction times (since path lengths along the tree are kept to tightly match direct Euclidean distances). Synthetic pyramidal cells grown with a non-optimal balancing factor on the other hand were clearly flawed, as illustrated by the example of layer 5 pyramidal neuron clones (Figure 7K, compare *bf* = 0 and *bf* = 0.2 with more adequate *bf* = 0.7). The electrotonic signature, developed in Figure 1 to compare electrotonic compartmentalization between neuronal trees, revealed the deficiencies of the synthetic dendrites with wrong balancing factor. When the balancing factor was too low, the electrotonic signature exhibited a distorted compartmentalization compared to an original tree (Figure 7L, leftmost). This can be loosely quantified by measuring the average size of an electrotonic compartment in real morphologies compared to synthetic ones with different balancing factors (Figure 7M). This quantity for a compartment size was obtained by averaging dendritic length exceeding 60% of the maximal potential deflection for current injections in all nodes one at a time. Thus, the balancing factor determines the degree of compartmentalization of the neuronal tree. This is expected, since a more stellate-like morphology associated with a higher balancing factor (Figure 2B, right side) should exhibit greater electrotonic segregation in its sub-trees.

### Relationship between the growth process and the network context

**Figure 8:**
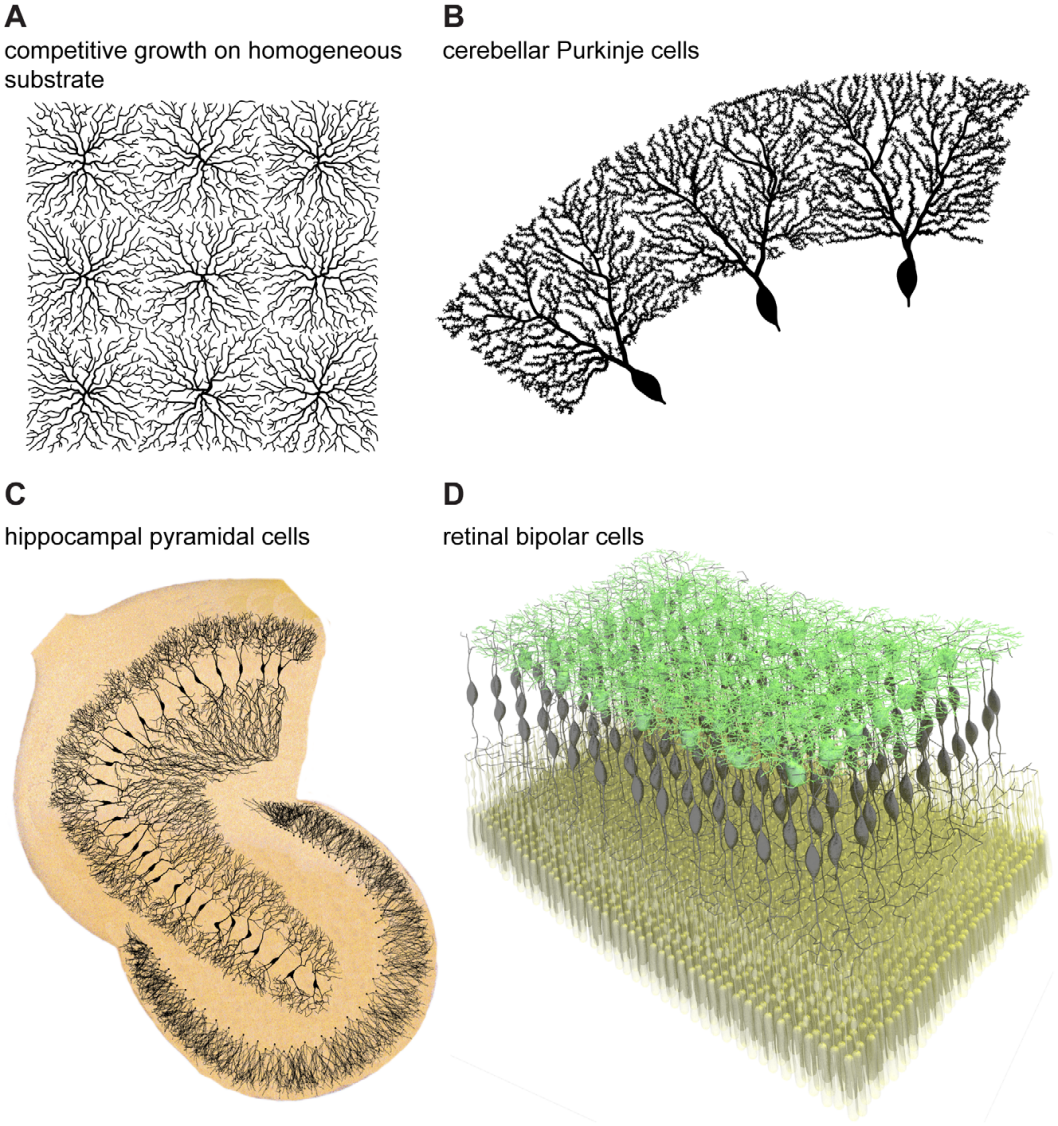
The interactions between neuronal branching and the network context. (A) Nine synthetic neuronal trees grown competitively on a sample square substrate of homogeneously distributed random carrier points: the competitive greedy growth results automatically in tiling of the available space. (B) Three out of 16 neuronal trees grown competitively on random carrier points distributed on a ring: this simulates well the sharp borders of Purkinje cells in the cerebellum. Whether Purkinje cell dendrites actually tile in sagittal planes of the cerebellum remains to be determined. (C) Hippocampal granule cells from Figure 4 were scaled and positioned along the contours of a human dentate gyrus obtained from a sketch by Camillo Golgi [31]. Growing synthetic CA3 hippocampal pyramidal cells competitively with the limits from the template resulted in realistic hippocampal pyramidal cells affected by mutual avoidance. Synthetic dendrites were overlaid on the background of the original sketch. (D) Bipolar cells (black) in the retina were grown competitively to connect an array of photoreceptors (yellow) to an array of starburst amacrine cells (green, obtained using the algorithm in Figure 3). In such a case the full morphology of bipolar cells is determined by the context of the circuitry, after prescribing soma locations of the bipolar cells. For all panels of Figure 8 precise scale bars would depend on the details of the preparations and were therefore omitted.

The local circuitry ultimately determines the context in which neuronal trees grow. There are global boundaries given by the neural tissue such as layers, topography or physical borders. However, competition for inputs between neighbouring neurons also seems to play a major role. Competition is easily implementable in the greedy growth algorithm introduced here because of the iterative nature of the algorithm. This can then be considered as a greedy extension of the growth algorithm and should be applicable in the network context. When grown under competitive conditions in which trees connect to a carrier point one after the other, the immediate consequence is spatial tiling. This can be seen in 2D for example when trees were grown from starting points on a spatial grid in a homogeneous substrate of random carrier points (Figure 8A). In fact, both Cajal's material cost and his conduction time cost independently lead to this type of tiling, which does not happen in the case of random wiring (not shown). Competitive dendrite growth can directly reproduce the sharp borders observed in Purkinje cell dendrites of the cerebellum. Using the geometric approach described in Figure 3 and 4, 16 cells were grown on random carrier points distributed homogeneously in a ring-shaped area in a competitive manner. As was the case for the apical tuft of pyramidal cells, Purkinje cell dendrites required to be grown in two stages: first the thick primary dendrites and then the thinner ones covered in spines (three sample cells are displayed in Figure 8B). The sharp borders of Purkinje cell dendrites could well be reproduced but whether this actually is a result of tiling in sagittal planes of the cerebellum remains to be determined experimentally. Cajal's laws can therefore explain more than just the inner branching rule: tiling between cells can emerge directly from his suggested optimization principles applied at the network level.

As mentioned above, the network context also plays a major role in governing neuronal spanning fields and their density profiles. Arranging the hippocampal granule cells developed in Figure 4 onto the contours of the dentate gyrus should for example fully determine their scaling variability (Figure 8C). Growing CA3 hippocampal pyramidal cells in a context-dependent manner (here in a competitive growth process bounded by the CA3 contours of the hippocampus, from Golgi, see Ref. [31]) might determine the variability in neuronal branching seen in the reconstructions. This is the right place to express a caveat regarding the use of branching statistics to compare dendritic structures. The branching statistics of two synthetic hippocampal pyramidal cells grown on both extremities of the CA3 region will differ entirely because of the different network context. This is the case even though these were grown using the exact same growth rule, they belong to the same cell class and they resemble their real counterparts. The idea that the network context determines a neuron's branching can be followed further: both input and output locations can serve as direct constraints for the cell morphology, as is the case when an array of photoreceptors (Figure 8D, yellow) in the retina connects to an array of starburst amacrine cells (Figure 8D, green obtained as in Figure 3) via a set of bipolar cells. In such a case, the input-output topography of the connection determines the morphology of bipolar cells given that these grow in a competitive manner (Figure 8D, black).

**Figure 9:**
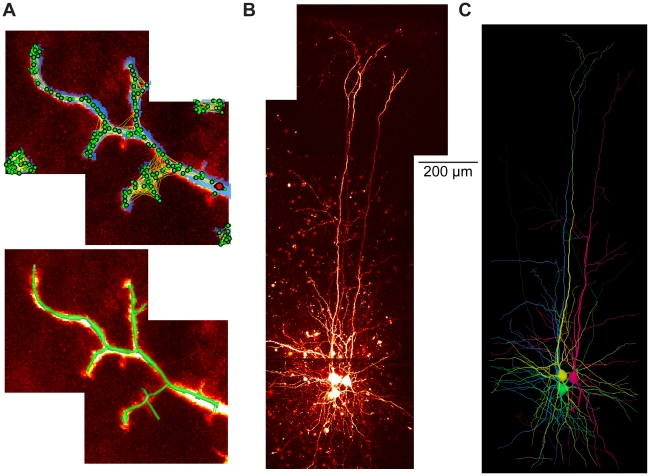
Automated reconstruction of multiple cells using the greedy algorithm. (A) Example of an additional application of the algorithm: automated model-based tree reconstruction from image stacks. Maximum intensity projection of tiled image stacks containing a sample sub-tree of a fluorescently labelled neuronal tree. Blue overlay in top panel corresponds to the output of a non-linear thresholding. The resulting binary matrix is then reduced to single points in space (green dots) via a skeletonization procedure. After a distance graph is obtained which describes the probability of a connection between these points due to the image data the points are used as carrier points for the growth algorithm to obtain the corresponding tree using the distance graph as an additional cost factor. After unlikely branches are removed the underlying tree structure is captured (green tree structure in the lower panel, see text for more detail; note absence of scale bar since this a sample image stack). (B) Maximum intensity projections of tiled 2-photon fluorescent image stacks acquired at 820 nm from primary visual cortex of a p13 JAX transgenic mouse (strain #007677, [38]) expressing GFP in parvalbumin interneurons, of which one is present. Three further layer 5 pyramidal neurons are also imaged; all cells were filled with a fluorescent dye Alexa 594 via whole cell patch-clamp recording. Data courtesy of Kate Buchanan and Jesper Sjöström. (C) Corresponding reconstructions (with the interneuron in green) grown in a competitive manner on the image stacks after manual post-processing.

Finally, as shown previously [20], the growth algorithm can serve as a tool for automatic reconstruction of neuronal trees from tiled image stacks containing fluorescently labelled neurons. Figure 9A displays an example of such a tiled image stack from a small part of an insect interneuron dendrite. In order to obtain the carrier points necessary to grow the tree, the image stack is first subjected to a local threshold (blue overlay) which is then 3D skeletonised (all green dots). The green dots are sparsened (only larger green dots with black surround) and a starting point is chosen (red dot). Apart from the cost functions described above, additional costs can be implemented here to connect the carrier points according to the image stack information (indicated by yellow lines between the dots). Connecting the carrier points results in a tree that can be further processed (Figure 9A, bottom, green tree structure). The procedure can be applied competitively and in an automated or semi-automated way to recover multiple trees such as the three entangled pyramidal cells and one interneuron from a set of tiled image stacks (Figure 9BC). Note that manual post-processing was required to obtain these clean reconstructions. But the simple representation of the cost function in the growth algorithm allows it to be easily extended to a state-of-the-art model-based reconstruction tool.

## Discussion

We present a new framework for understanding dendritic branching in neurons based on the use of graph theory. Our results demonstrate that the laws of conservation of cytoplasm and conduction time formulated by Ramón y Cajal from simple observation represent a fundamental constraint to dendritic branching. Generation of synthetic tree structures using a simple algorithm derived from these constraints creates highly realistic neuronal branching structures across a wide range of neuronal types. Nevertheless, there remains flexibility within these natural constraints. Notably, the weighting of both components, the balancing factor – which determines a neuron's electrotonic compartmentalization – is an adjustable parameter and can differ from one cell type to the next. Within these constraints, neuronal processes can grow and adapt depending on their specific functionality. Most strikingly, the spanning field and density profile are key determinants of nerve cell individuality and depend on the sharp physical boundaries of the tissue and on the network input-output topography. Finally, additions to this rule are required in certain cases such as the suppression of multifurcations and the addition of spatial jitter. These might relate to the developmental bio-mechanistic growth process or specific computational features and we are confident we will find many more in further preparations.

### Relating computation and morphology

The spanning field in which a dendrite grows plays a major role in defining the computational and functional features of axons and dendrites. This is reflected in its importance in the process of accurately reproducing single cell morphologies. Furthermore, to replicate dendrite regions such as the apical tuft of layer 5 pyramidal neurons or the primary dendrites of Purkinje cells, a timed growth process was required in which subparts of a region were grown in a second step. This could indicate a functional constraint governing neuronal outgrowth in these cells. However, two limitations of the greedy algorithm must be considered. Firstly, the growth process does not guarantee a global optimum since it is based on an algorithm which optimizes at the local level, adding carrier points one by one. Secondly, it does not involve volumetric considerations. Both cable diameter when optimizing the amount of material used and space packing issues in conjunction with axons and dendrites of other neurons as well as with glia cells are known to play a role in determining wiring properties in the brain [3]. It is likely that some of these restrictions are responsible for the extra steps necessary in the construction of synthetic neuronal branching structures. These two possible extensions are good starting points for subsequent studies.

### Spanning fields and the network context

We show that spatial tiling as observed in many dendritic structures (Figure 8, [32], [33]) is a direct consequence of Cajal's laws when applied at the network level. Indeed, network structure in general is expected to be determined by the same optimization principles, a feature which Cajal highlighted throughout his work. We have implemented this directly with the example of bipolar cells, whose carrier points were directly obtained from arrays of other existing input and output neurons rather than indirectly from its individual spanning field and its respective density profiles. By optimally arranging input and output locations, the spanning field, the major contributor to shaping the synthetic neuronal trees we have presented here, was strictly constrained by the same wiring and conduction time costs. Defining starting locations (e.g. somata) and synaptic target partners should therefore generally suffice to fully determine the architecture of a network. This is most likely to be a general principle and should be investigated further in future work.

### Methods derived from analyzing neuronal trees as graphs

Based on the formalisms of optimized graphs, we have derived several new ways of representing dendritic structure and function. First, we show that graph resampling and labelling order lead to an objective representation for electrotonic compartmentalization. Simplified models which still faithfully represent the compartmentalization behaviour can be obtained with such a process. Second, taking advantage of their scalability, we derived generalized spanning fields and their density profile descriptions. These representations may be useful for comparing branching structures of different neuronal cell types. We show here how these can be useful for generating synthetic neuronal tree clones. Finally, as mentioned previously [20] after extracting carrier points directly from image stacks, the greedy algorithm is capable of a model-based automated reconstruction of neuronal trees from microscopy data. The simplicity of the algorithm and the fact that the cost factors are arbitrarily adjustable render this method an easily extendable tool. This could be crucial for combining the wide existing set of various approaches [34]–[37] in one process. For example, costs for segment orientation [37] can be integrated into the cost function directly.

### Conclusion

In summary, we find that a simple growth algorithm which optimizes total cable length and the path length from any point to the root in an iterative fashion can generate synthetic dendritic trees that are indistinguishable from their real counterparts for a wide variety of neurons. This represents a direct validation of the fundamental constraints on neuronal circuit organization described originally by Cajal. Furthermore, this approach provides a new framework for understanding dendritic tiling, which is a direct consequence of using this algorithm. The availability of these tools in a comprehensive software package (the TREES toolbox; see Protocol S1) should now allow these principles to be applied to any arbitrary dendritic or axonal architecture, and permit synthetic neurons and neural networks to be generated with high precision.

## Methods

We have developed a software toolbox, the TREES toolbox (deposited at www.treestoolbox.org), written in MATLAB (Mathworks, Natick, MA), with corresponding extensive documentation. All methods used in this paper are based on applications from this toolbox and are only explained briefly here; detailed documentation regarding the toolbox is presented in Supplementary Information (Protocol S1). Dendritic morphologies were obtained from the neuromorpho database (www.neuromorpho.org, see [16]).

### Label sorting

The labelling of the nodes of a tree should be unique in order to, for example, compare the graphs of two trees topologically or electrotonically. In order to obtain such a unique labelling, nodes were first sorted according to their topological depth, chosen here to be the sum of the path length values of all children. Each node was then inserted in that order into a one-dimensional string, one-by-one directly behind its direct parent node. Subsequently, the resulting string of labels was mapped back onto the nodes of the tree. We refer to the Supplementary Information for more details on this subject (Protocol S1).

### Resampling

The direct comparison of two trees along strict criteria also requires a unique distribution of node locations on the graph. We redistributed nodes on a tree structure with equal inter-nodal distances, a process we termed resampling. Each terminal branch was first lengthened by half the sampling distance. Then, starting at the root, extra nodes were positioned at integer multiples of the sampling distance along the path of the tree. All other nodes were then removed while maintaining the connectivity. During this process all segments become shorter or remain the same length; this is because a wriggly path is simplified by a straight line (which is by definition always shorter). In order to preserve the total branching length and the electrotonic properties all segments were then elongated to the given sampling length (Figure 1B, length conservation).

### Electrotonic equivalent

Rearranging the metrics of a tree after sorting the labels leads to generation of its unique electrotonic equivalent tree. To obtain the equivalent tree, its metrics were rearranged according to a circular dendrogram where the angle towards which a segment is directed within a circle around the root corresponds to the value of the label of its target node.

### Electrotonic signature

To obtain the electrotonic signature, the conductance matrix describing the axial conductances along the edges of the graph (following the adjacency matrix structure) and the membrane leak conductances (on the diagonal of the matrix) was simply inverted. The result is a potential matrix (in mV) corresponding to the resulting steady-state potential in one node when 1 nA current was injected in another node, i.e. the current transfer from any node to another. The passive axial and membrane conductances were 100 Ωcm and 2000 Ωcm^2^ for the sample insect dendrite sub-tree in Figure 1 and 100 Ωcm and 20000 Ωcm^2^ for the layer 5 pyramidal cells in Figure 7.

### Extended minimum spanning tree

The branching growth was implemented as a greedy algorithm [23] as in [19], [20]. In some cases (Figure 5C rightmost, and all morphologies in Figures 6, 7 and 8), quadratic diameter decay was mapped on the resulting trees [19] and a soma-like increase of diameters was obtained by applying a cosine function to the diameters in close vicinity of the root (see Protocol S1 for exact methods).

### Automated reconstruction

Two-photon microscopy 3D image stacks containing neurons filled with a fluorescent dye or expressing a fluorescent protein were subject to local brightness level thresholding. After 3D skeletonization and sparsening of the resulting carrier points, these were submitted to the same greedy algorithm (started at a user defined dendrite root location) as used for obtaining synthetic dendrites. In the case of multiple entangled neurons as in Fig. 9BC, manual cleaning was required using the user interface provided by the TREES toolbox. See Supplementary Information for more information including the software implementing these algorithms (Protocol S1).

## Supporting Information

Protocol S1 The TREES toolbox documentation.
(6.97 MB PDF)
