# Supplementary material for: One Rule to Grow Them All: A General Theory of Neuronal Branching and Its Practical Application
Source: PLoS Comput Biol. 2010 Aug 5;6(8):e1000877. doi: 10.1371/journal.pcbi.1000877 (PMC2916857; doi:10.1371/journal.pcbi.1000877)
Supplement: Protocol S1 — The TREES toolbox documentation. (6.97 MB PDF) [file pcbi.1000877.s001.pdf]

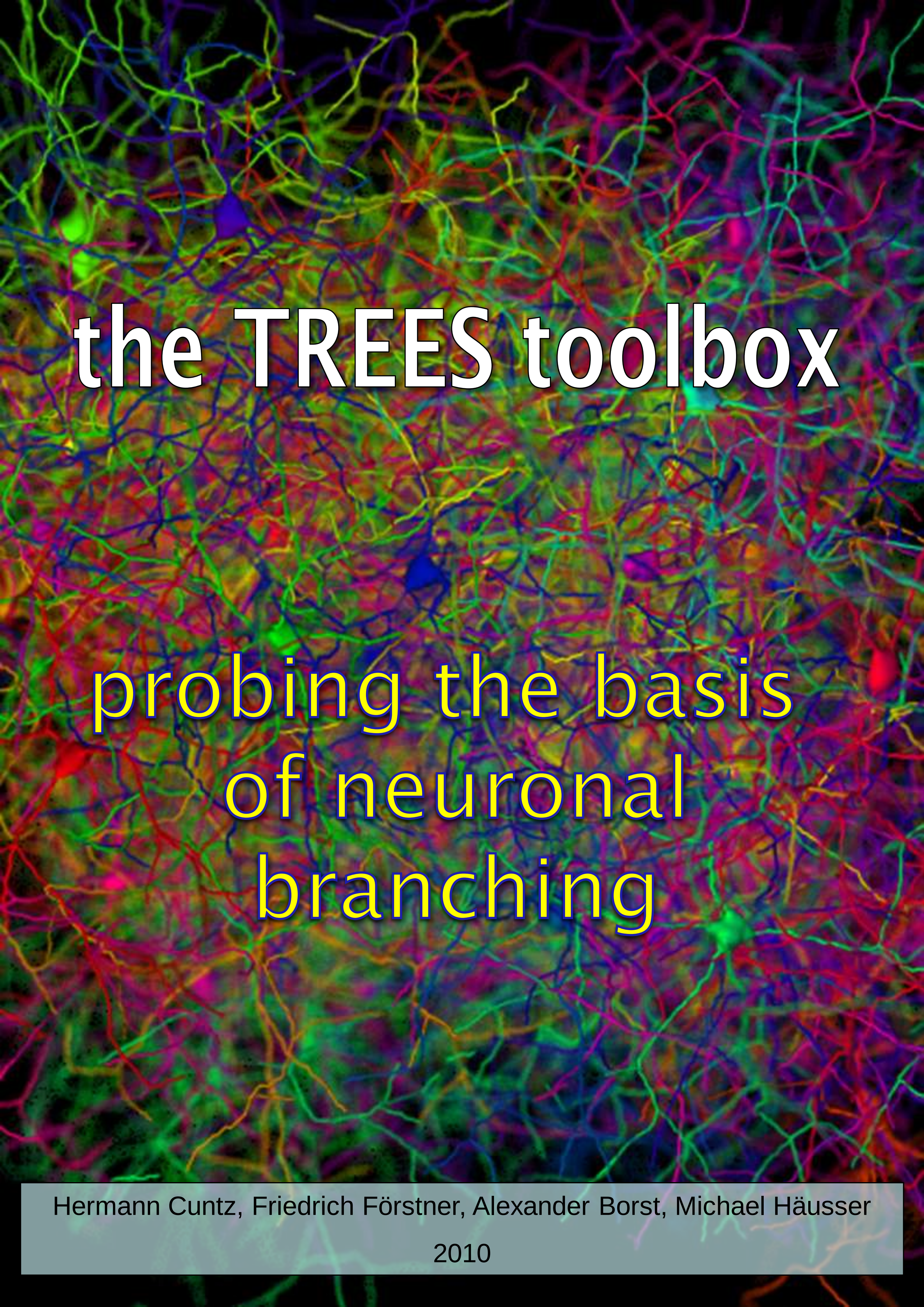

the TREES toolbox

probing the basis of neuronal branching

Hermann Cuntz, Friedrich Förstner, Alexander Borst, Michael Häusser

2010

Foreword

More than a century ago, Ramón y Cajal provided a qualitative description of neuronal branching in all its forms and variants. However, few rigorous and useful formalisms exist even today to describe neuronal branching. In particular, working with functional models consisting of detailed neuronal morphologies reveals this gap. Ways to compare branching structures between different types of neurons or neurons of the same type are a source of growing interest in the neuroscience community, and yet morphological statistics remain mostly unrelated to their functional impact.

With the TREES toolbox we aim to take two important steps:

1. We start with a simple description of neuronal morphology and provide the basic tools to edit, visualize and analyze neuronal trees on the basis of this description.

2. We develop an approach which assumes that neuronal branching can largely be expressed by local optimization of total wiring and conduction distances, and provide tools to automatically reconstruct neuronal branching from microscopy image stacks and for generating artificial branched structures.

This software package is written in Matlab (Mathworks, Natick, MA), the most widely used scientific programming language. We hope that other groups can therefore easily add to the TREES toolbox with their own code for their own specific applications, and the code is therefore freely distributed. When publishing scientific work using this toolbox please cite the current paper:

"One rule to grow them all: A general theory of neuronal branching and its practical application. Cuntz H, Forstner F, Borst A, Häusser M 2010 PLoS Computational Biology".

We encourage users of the toolbox software to recommend the toolbox to their peers, and also to funding and award agencies.

In return for the services we provide with this toolbox, we invite users to incorporate any extensions and/or related code which they develop. Ideally, suggestions for improvements or add-ons to the code should be sent directly as improved pieces of code. The contributor's name will be mentioned in the header of the function when integrated in the toolbox and in the toolbox documentation. For the contribution of a new method to either generate artificial neurons or reconstruct neuronal morphology from image stacks we offer to call the core function "lastnameofcontributor_tree" to acknowledge the author's contribution. We hope that this will provide a further incentive for making contributions to the toolbox.

Hermann Cuntz, Friedrich Forstner, Alexander Borst, Michael Häusser

This work was supported by the Gatsby Charitable Foundation, the Wellcome Trust, the Alexander von Humboldt Foundation, and the Max-Planck Society.

This document is supplementary material Protocol S1 to a manuscript entitled "One rule to grow them all: A general theory of neuronal branching and its practical application" published in PLoS Computational Biology. The software package and updated materials are available at www.treestoolbox.org.

Table of contents

Definitions

a tree is a graph, p. 4
adjacency matrix, p. 5
using graph theory, p. 6-7
BCT formalism, p. 8-9
sorted and equivalent tree p.10
the “topological gene”, p. 11
tree morphing, p. 12
electrotonic signature, p. 13-14
resampling a tree, p. 15
MST rule, p. 16-17
diameter tapering, p. 18
Nx1 vectors, p.19
obtaining some statistics, p.20

First steps

starting with TREES, p. 22
exploration of a tree, p. 23
visual exploration, p. 24
code comments, p. 25

Reference

contents, p. 26
full function list, p.27
general remarks, p. 28
graphtheory, p. 29-50
edit, p. 51-61
metrics, p. 62-79
graphical, p. 80-94
construct, p. 95-113
electrotonics, p. 114-124
IO, p. 125-133

scheme, p. 135
stacks, p. 136-144
sample, p. 145

GUI

starting the GUI, p. 146
the vis_ panel, p. 147-149
the stk_ panel, p. 150-153
the thr_ panel, p. 154-155
the skl_ panel, p. 156-157
the mtr_ panel, p. 158-165
the cat_ panel, p. 166
the ged_ panel, p. 167-170
the slt_ panel, p. 171-172
the ele_ panel, p. 173
the plt_ panel, p. 174-177

Definitions a tree is a graph

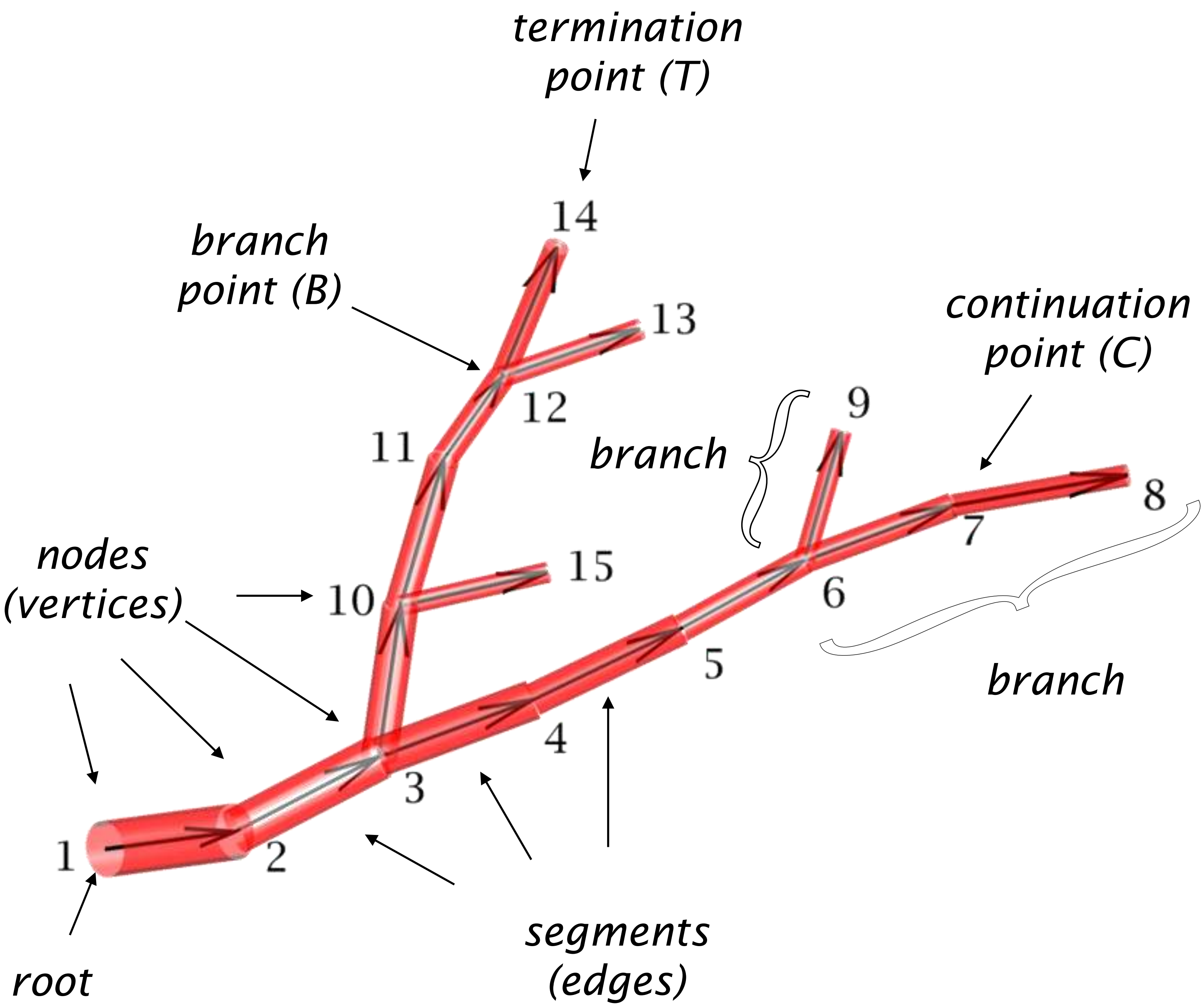

As a graph, a tree is represented by a set of labelled nodes connected by edges. Since most statistics describing a neuron‘s branching relate to the root (e.g. branch order, which increases after each branch point on the way from the tree root to all terminal nodes) it makes sense to attribute a directionality to the edges and to define the root as the node with the index 1. All edges lead away from the root. That defines their directionality uniquely.

Definitions adjacency matrix

The directed adjacency matrix describes how nodes are directionally connected to a graph

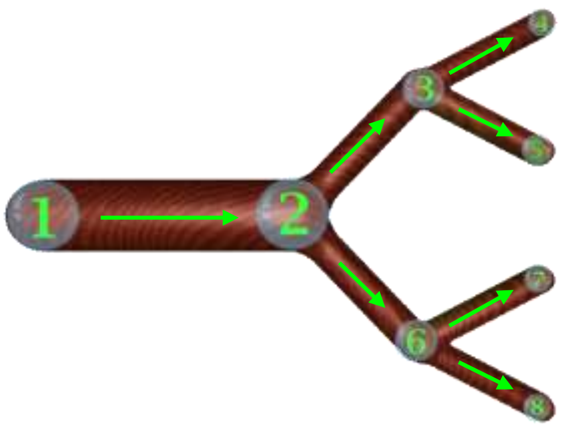

	1	2	3	4	5	6	7	8
1	0	0	0	0	0	0	0	0
2	1	0	0	0	0	0	0	0
3	0	1	0	0	0	0	0	0
4	0	0	1	0	0	0	0	0
5	0	0	1	0	0	0	0	0
6	0	1	0	0	0	0	0	0
7	0	0	0	0	0	1	0	0
8	0	0	0	0	0	1	0	0

adjacency matrix *dA*

When neuronal trees are regarded as graphs, their branching structure can be well described with the corresponding directed adjacency matrix *dA* (see “dA_tree”), a quadratic matrix of size *NxN* where *N* is the number of nodes in the tree. As mentioned earlier, the direction of the edges shows away from the first point, representing the arbitrary starting vertex *S* (= 1), the root of the tree. Note that the widely used .swc format (Cannon RC, Turner DA, Pyapali GK, Wheal HV, 1998, J Neurosci Methods 84: 49-54) for storing neuronal morphology is nothing else than a sparse representation of the adjacency matrix since it simply attributes to all nodes (row index) a parent node (column index).

Not each possible directed adjacency matrix represents a possible neuronal tree, since loops and branching points with more than two child branches are possible, but do not exist in natural dendritic trees. *dA* therefore never contains more than two entries in one column and no entry will lay directly on the diagonal. Also, each node has exactly one parent, apart from the root, which has none. Each row of *dA* therefore contains exactly one entry apart from the first, which contains none.

In order to derive most dendritic branching statistics using the typical descriptions, an algorithmic formulation by recursion is required to “walk” through a tree and collect statistics. Many operations for example on dendritic trees require processing with a stack and can therefore not be written analytically. With repeated matrix multiplication on the directed adjacency matrix as in dA^r the (*i*, *j*)-entry represents the number of distinct *r*-walks from node *i* to node *j* in the graph.

Using simple multiplications of the adjacency matrix allows one to “walk” through a tree

Therefore, the derivation of some elementary branching properties follows directly from the graph representation of the tree. As such, the child nodes of each node *i* can be read out in the non-zero elements directly from *dA* in column *i*. The index of the direct parent node *idpar* to any node *i* (see “idpar_tree”) is simply the *i*-th element of:

0	0	0	0	0	0	0	0		1	=	0	1	2	3	3	2	6	6
1	0	0	0	0	0	0	0		2									
0	1	0	0	0	0	0	0		3									
0	0	1	0	0	0	0	0		4									
0	0	1	0	0	0	0	0	x	5									
0	1	0	0	0	0	0	0		6									
0	0	0	0	0	1	0	0		7									
0	0	0	0	0	1	0	0		8									

$$idpar = dA \times \begin{pmatrix} 1 & 2 & \ldots & N \end{pmatrix}^T$$

Further order *r* parents are simply obtained by applying repeated matrix multiplication (see “ipar_tree”):

$$ipar^r = dA^r \times \begin{pmatrix} 1 & 2 & \ldots & N \end{pmatrix}^T$$

Where r = 0 corresponds to the node itself, r = 1 the parent, r = 2 the grand-parent etc…

Definitions using graph theory (II)

Topological path length and branching order values can be obtained by such matrix multiplications

$$dA = \begin{bmatrix} 0 & 0 & 0 & 0 & 0 & 0 & 0 & 0 \\ 1 & 0 & 0 & 0 & 0 & 0 & 0 & 0 \\ 0 & 1 & 0 & 0 & 0 & 0 & 0 & 0 \\ 0 & 0 & 1 & 0 & 0 & 0 & 0 & 0 \\ 0 & 0 & 1 & 0 & 0 & 0 & 0 & 0 \\ 0 & 1 & 0 & 0 & 0 & 0 & 0 & 0 \\ 0 & 0 & 0 & 0 & 0 & 1 & 0 & 0 \\ 0 & 0 & 0 & 0 & 0 & 1 & 0 & 0 \end{bmatrix} \quad dA_1 = \begin{bmatrix} 0 \\ 1 \\ 0 \\ 0 \\ 0 \\ 0 \\ 0 \\ 0 \end{bmatrix} \quad dA \times dA_1 = \begin{bmatrix} 0 \\ 0 \\ 1 \\ 0 \\ 0 \\ 1 \\ 0 \\ 0 \end{bmatrix} \quad dA^2 \times dA_1 = \begin{bmatrix} 0 \\ 0 \\ 0 \\ 1 \\ 1 \\ 0 \\ 1 \\ 1 \end{bmatrix}$$

Correspondingly, the vector of topological path lengths *PL* (see "PL_tree") from all nodes to the root of the directed graph can be obtained as follows:

$$\vec{PL} = \sum_{r=1}^{N} r \cdot \left(dA^{(r-1)} \times dA_1 \right)$$

where dA_1 is the first column of *dA*.

A similar approach can be used to obtain the vector of branch order values for all elements compared to the root of the graph in position 1. A supporting adjacency matrix *sdA* is required, which is weighted by the number of child nodes of each node:

$$sdA = dA \cdot \left(diag(sum(dA)) \right)$$

By multiplying this matrix, branch points get potentiated, and the branch order *BO* (function "BO_tree") can be extracted by taking the base 2 logarithm:

$$BO = \log_2 \sum_{r=1}^{N} \left(sdA^{(r-1)} \times sdA_1 \right)$$

Where sdA_1 is the first column of *sdA*.

Definitions BCT formalism

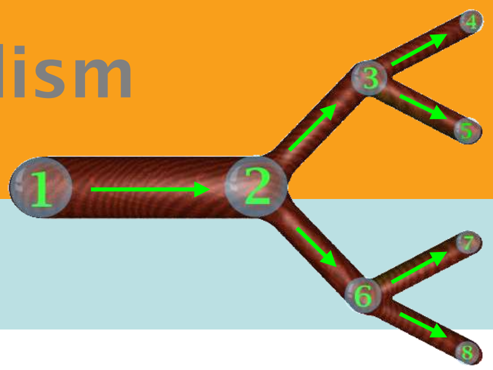

With the BCT form, the topology of a tree can be written as a simple string

	1	2	3	4	5	6	7	8
1	0	0	0	0	0	0	0	0
2	1	0	0	0	0	0	0	0
3	0	1	0	0	0	0	0	0
4	0	0	1	0	0	0	0	0
5	0	0	1	0	0	0	0	0
6	0	1	0	0	0	0	0	0
7	0	0	0	0	0	1	0	0
8	0	0	0	0	0	1	0	0
	1	2	2	0	0	2	0	0
	C	B	B	T	T	B	T	T

BCT formalism

None of the common formats for dendritic trees represent one and the same tree in a unique way. This becomes very clear for the graph representation where all permutations of labels (indices) $i \rightarrow j$ result in the same tree:

$$dA(i,i) \rightarrow dA(j,j)$$

Again, the same is true for the .swc format. A more constrained representation is given by the BCT formalism (which was developed as far as we know by Rocky Nevin and implemented in the compartmental modelling software *NeMoSys, Eeckman FH, Theunissen FE and Miller JP, 1994, Nemosys: a system for realistic single neuron modeling. In Neural Network Simulation Environments, ed. Skrzypek J, pp. 114-135. Boston, Dordrecht, London: Kluwer Academic Publishers*). There, the node labels are sorted hierarchically so that the nodes of a sub-tree remain in sequence and within each sub-tree parent nodes always precede their respective daughter nodes. This was done in our example: the resulting BCT string can then be read out by summing over the columns of *dA*.

Nodes where this sum is 0 (no daughter nodes) are termed “T” for terminal. Nodes where the sum is 1 are termed “C” for continuation. Nodes where the sum is 2 are termed “B” for branch. The resulting string reads “CBBTTBTT”:

“Define a root and start a branch. Continue to next node (C). Open new branch (B_1). Open new branch (B_2). Terminate last branch which is still open (T_2). Terminate previous branch (T_1). Open new branch (B_3). Terminate last opened branch (T_3). Terminate full tree (T_0).”

The adjacency matrix is fully described by the BCT string and additionally the labelling of the nodes is now more restricted. But at each branch point permuting the sub-trees would still result in the exact same underlying tree.

A simple way to arrange the node labels to conform to BCT is to insert each node one by one directly behind its parent node and to re-label the nodes after the whole process (see “sort_tree” function of the TREES toolbox).

An important note here: If the nodes are pre-sorted beforehand (for example lexicographically or by level order and topological path length, see section “sorting a tree”) a perfectly unique representation of the topology can be obtained. Note also: In BCT form, all entries in *dA* are strictly below the diagonal.

The BCT form can be obtained by a simple algorithm

In order to find the directed adjacency matrix from a BCT string (see “BCT_tree”) by maintaining the order of elements (metrics can then be directly transferred) the following algorithmic procedure can be applied:

```
% basic algorithm:
Set dA to square matrix of zeros
Use a stack
For i = 1:N
            if index exists then dA(i,index) = 1
            index = i
            If BCT(i) == ‘0|T’ then index = POP stack
            If BCT(i) == ‘2|B’ then PUSH i to stack
End
```

If an adjacency matrix represents a correct BCT order, a pointer starting with one at the root diminishing by one for each terminal and increasing by one for each branching point should become zero exactly at the end of the string (position N, number of nodes). This can be represented by the cumulative sum C_x:

$$T = \vec{1}^{1\times N} \times dA \qquad\qquad C_i = 1 + \sum_{j=1}^{i} (T_j - 1)$$

$$isBCT = \begin{cases} true & if \quad only \quad C_N = 0 \\ false & \quad else \end{cases}$$

Note that one rough way to obtain all possible BCT strings with N nodes is by setting all numbers from 0 to 3^N-1 into base 3 and verifying whether they are BCT.

Definitions sorted and equivalent tree

Even in BCT order the representation of a tree’s connectivity matrix is not unique. Sorting the labels is a solution

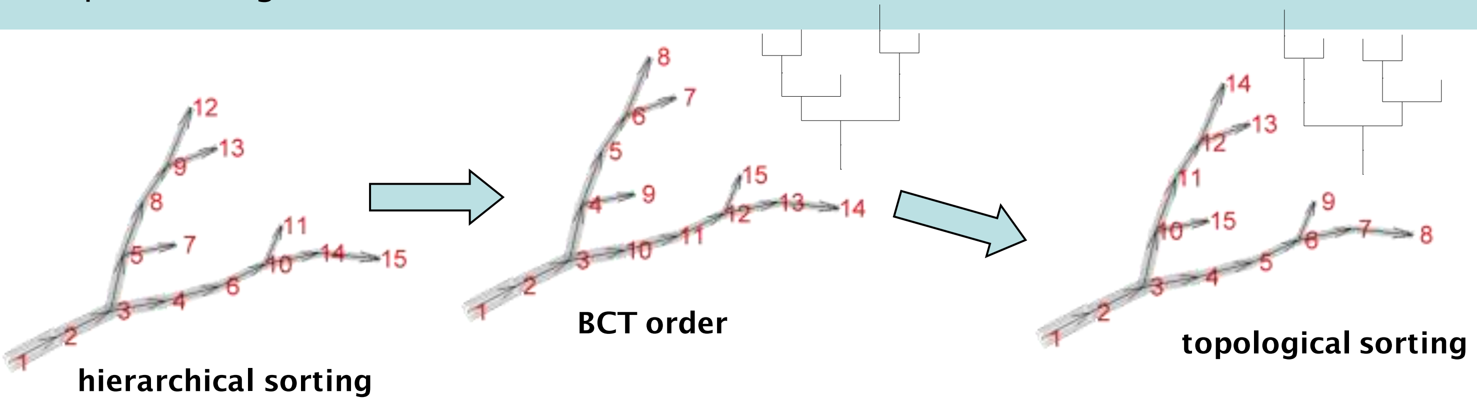

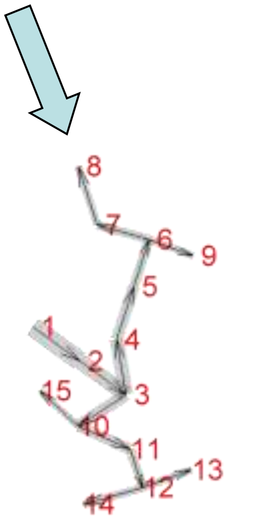

equivalent tree

The labeling of the nodes of a tree should be unique if one wants to for example compare the graphs of two trees topologically or electrotonically. In principle, the labels on the nodes describing a graph can be attributed arbitrarily. As mentioned before, all permutations of labels (indices) $i \rightarrow j$ result in the same tree by rearranging the adjacency matrix and any metric elements attributed to the nodes:

$$dA(i,i) \rightarrow dA(j,j)$$

$$X(i) \rightarrow X(j) \qquad Y(i) \rightarrow Y(j) \qquad Z(i) \rightarrow Z(j) \qquad D(i) \rightarrow D(j)$$

In a **hierarchical sorting**, node label values always increase towards daughter nodes. This can constrain the otherwise arbitrary labelling. As discussed before, labelling can be constrained further in the **BCT order** (see introduction part “BCT formalism”). Within any sub-tree, the labelling is then continuous. A truly unique labelling arises in a **topological sorting** when labels additionally carry a weight according to some topological values such as the topological depth or the number of child nodes. At each branch point for example, the heavier sub-trees can then be labelled first. Rearranging the metrics of a tree based on its topologically sorted label order leads to a unique electrotonic **equivalent tree.**

In order to arrive to such a labelling, nodes are first sorted according to their topological depth. Each node is then inserted in that order into a one dimensional string one by one directly behind its direct parent node. Subsequently, the resulting string of labels is mapped back onto the nodes of the tree.

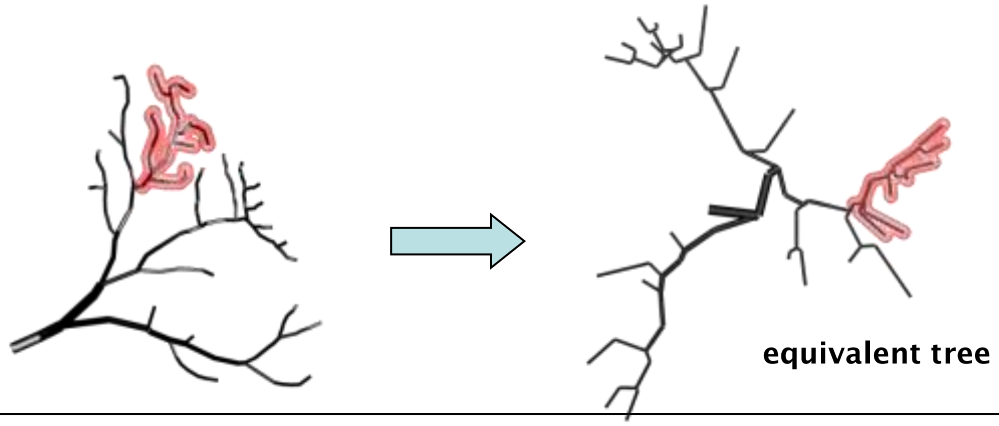

Definitions the “topological gene”

Variants of the BCT string can be used as a “topological gene” description of a tree

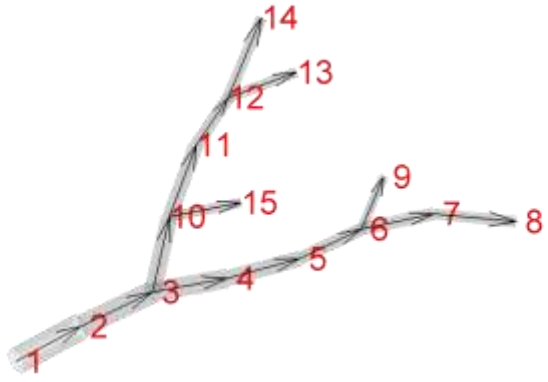

Once the labels of a tree are topologically sorted (see “sort_tree”), a unique representation of the topology is given by the topological “gene” (see “gene_tree”):

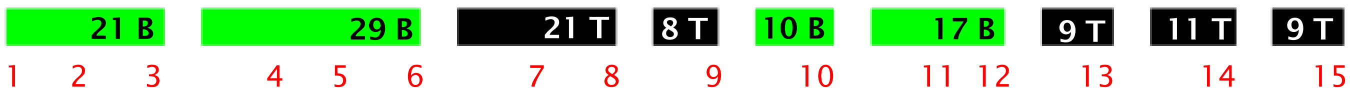

green segments are branches ending with a branch point, black segments are branches ending with a termination point; the order is determined by the topological sorting described on the previous page. Numbers on the “gene” branches (and also their actual length) correspond to the path length along each branch. In this case the node labels are displayed under the “gene” for descriptive purposes. Apart from the diameter mapping, the equivalent tree can be reconstructed solely from this one-dimensional string.

Because of the continuous labelling sub-trees of the original tree are continuous bits within the topological “gene” (see red contours).

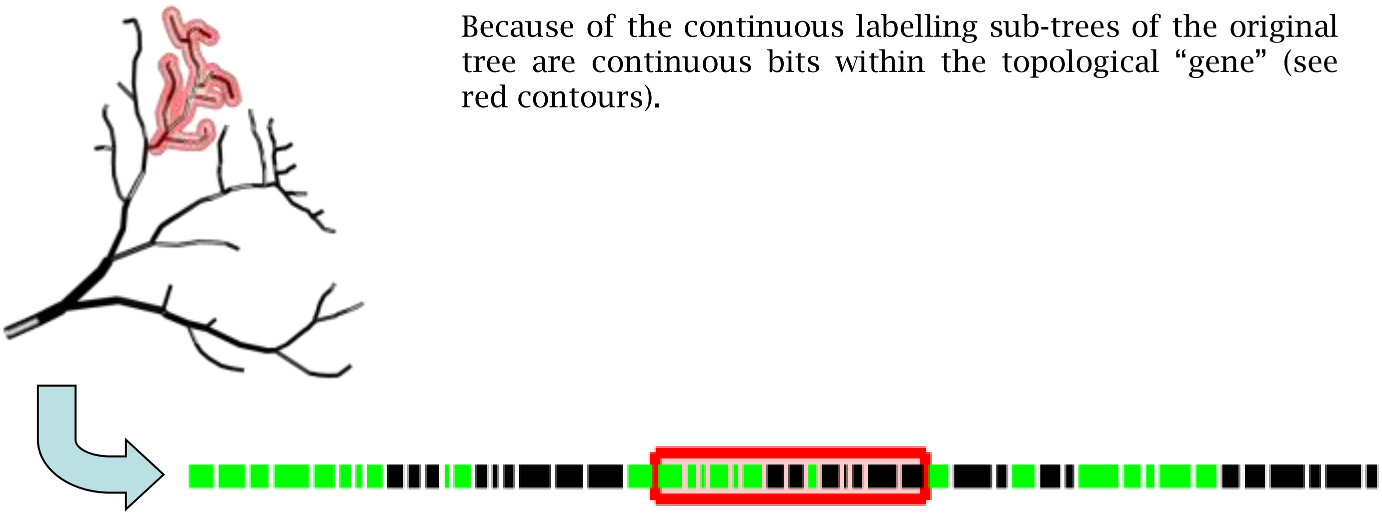

Definitions tree morphing

Morphing a tree is the process of mapping new metric length values on an existing tree, while preserving its topology and local angles

Segev and colleagues derived a new representation of neuronal trees depending on electrotonic measures. The method used for this representation was called the "Morphoelectrotonic Transform" (Zador et al. 1995, J Neurosci 15(3):1669-82).

In fact, the method used in that case is very generalizable. Any *Nx1* vector of length values may be mapped on a tree with *N* nodes. This is done by scaling the length value l_i of all segments to the new segment lengths while conserving the direction of the segment indicated by the direction vector (arrow below). At each step the entire sub-tree needs to be translated accordingly.

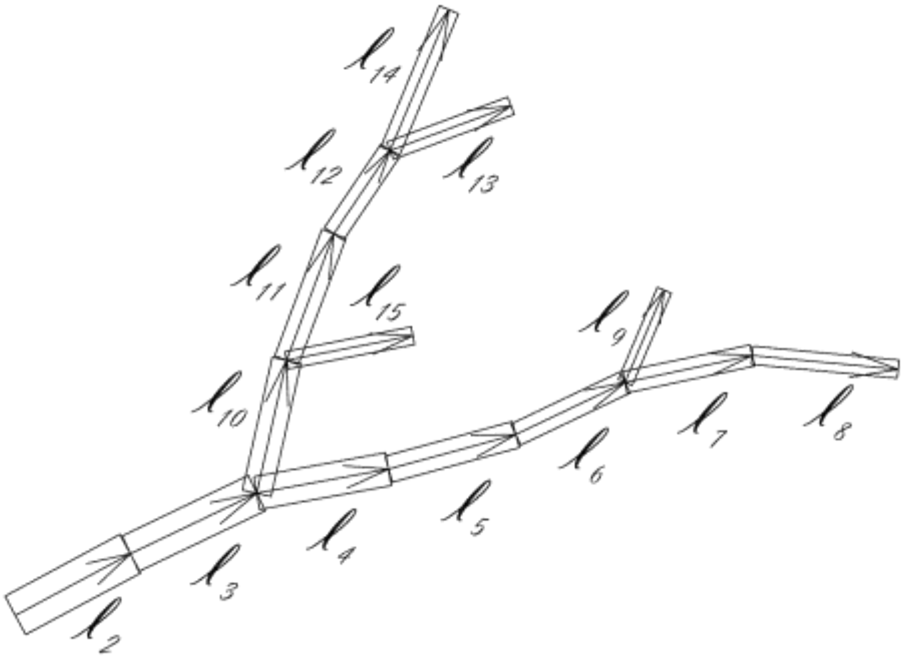

Only in the case of 0-length segments, a direction needs to be picked arbitrarily. A TREES toolbox function (see "morph_tree") performs this type of morphing operation which can have various applications of which the morpho-electrotonic transform is just one .

On the path sum (see "Pvec_tree"), child sum (see "child_tree"), parent daughter ratio (see "ratio_tree"), segment binning (see "bin_tree") are further examples of such "meta-functions" which apply an *Nx1* vector on a tree structure to result in a wide variety of applications.

Definitions Electrotonic signature

Using linear algebra in combination with Kirchhoff's laws of current conservation, the adjacency matrix can be used directly to obtain a full signature of passive steady-state current propagation in a tree

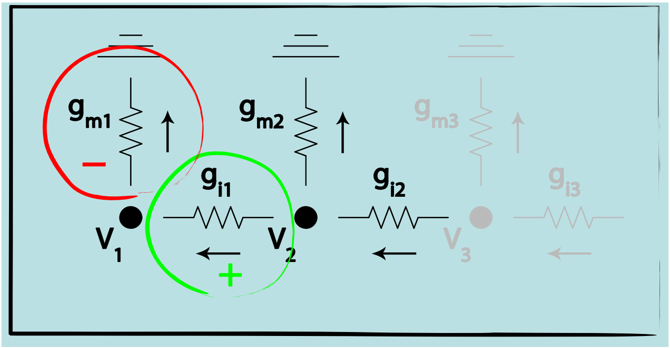

$$g_{i1}(V_1 - V_2) + g_{m1}(V_1 - 0) = 0$$

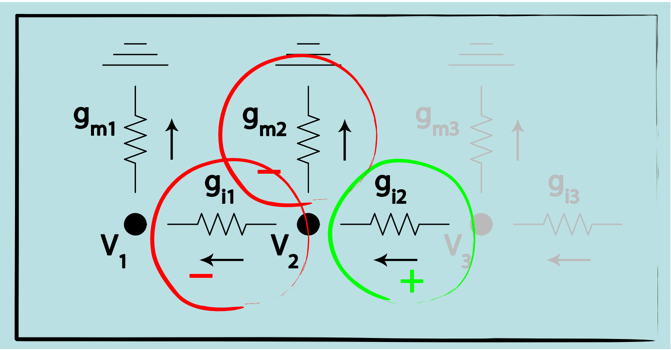

$$g_{i1}(V_1 - V_2) + g_{m1}(V_1 - 0) = 0$$
$$g_{i2}(V_2 - V_3) + g_{i1}(V_2 - V_1) + g_{m2}(V_2 - 0) = 0$$

Combining Kirchhoff's junction law for electrical circuits with simple linear algebra, a matrix can be derived which describes the equivalent circuit of a tree. If we cut the circuit at node 3 in the above example the following matrix appears:

$$\underbrace{\begin{pmatrix} g_{m1} + g_{i1} & -g_{i1} & 0 \\ -g_1 & g_{m2} + g_{i1} + g_{i2} & -g_{i2} \\ 0 & -g_{i2} & g_{m3} + g_{i2} \end{pmatrix}}_{M} \times \begin{pmatrix} V_1 \\ V_2 \\ V_3 \end{pmatrix} = \begin{pmatrix} I_1 \\ I_2 \\ I_3 \end{pmatrix}$$

$$M = G_m D_S + G_a \{ \text{diag} [\text{sum} (AD_l + D_l A^T)] - (AD_l + D_l A^T) \}$$

G_m, G_a: spec. membrane and axial conductances
D_s, D_l: diag. matrices with compartment surfaces and inverse volumes
A: adjacency matrix

Dividing a vector or matrix of input currents I by the conductance matrix M results in potential vectors V (or matrix respectively) according to Ohm's law.

Definitions Electrotonic signature (II)

The electrotonic signature describes the compartmentalization of the neuronal tree

Simply taking the inverse of the conductance matrix *M* (see previous page) results in the steady state electrotonic signature of the tree:

$$V_{SSE} = M^{-1}$$

This electrotonic signature (see "sse_tree") describes well the electrotonic compartmentalization of a neuronal tree. In this case the matrix of input currents *I* is simply the identity matrix. Currents of 1 (nA) are therefore injected one at a time in each node in subsequent columns or rows. The symmetrical square matrix V_{SSE} contains in each column or row the potential distribution in all nodes following the current injection in the corresponding node (i.e. the current transfer). The diagonal therefore contains the local input resistances since there the potential change is measured in each node resulting from current injection into the same node. Red squares correspond to sub-trees with increased electrotonic inter-connectivity. The electrotonic signature therefore follows closely on the adjacency matrix (as can be seen from the relationship between *M* and *dA*).

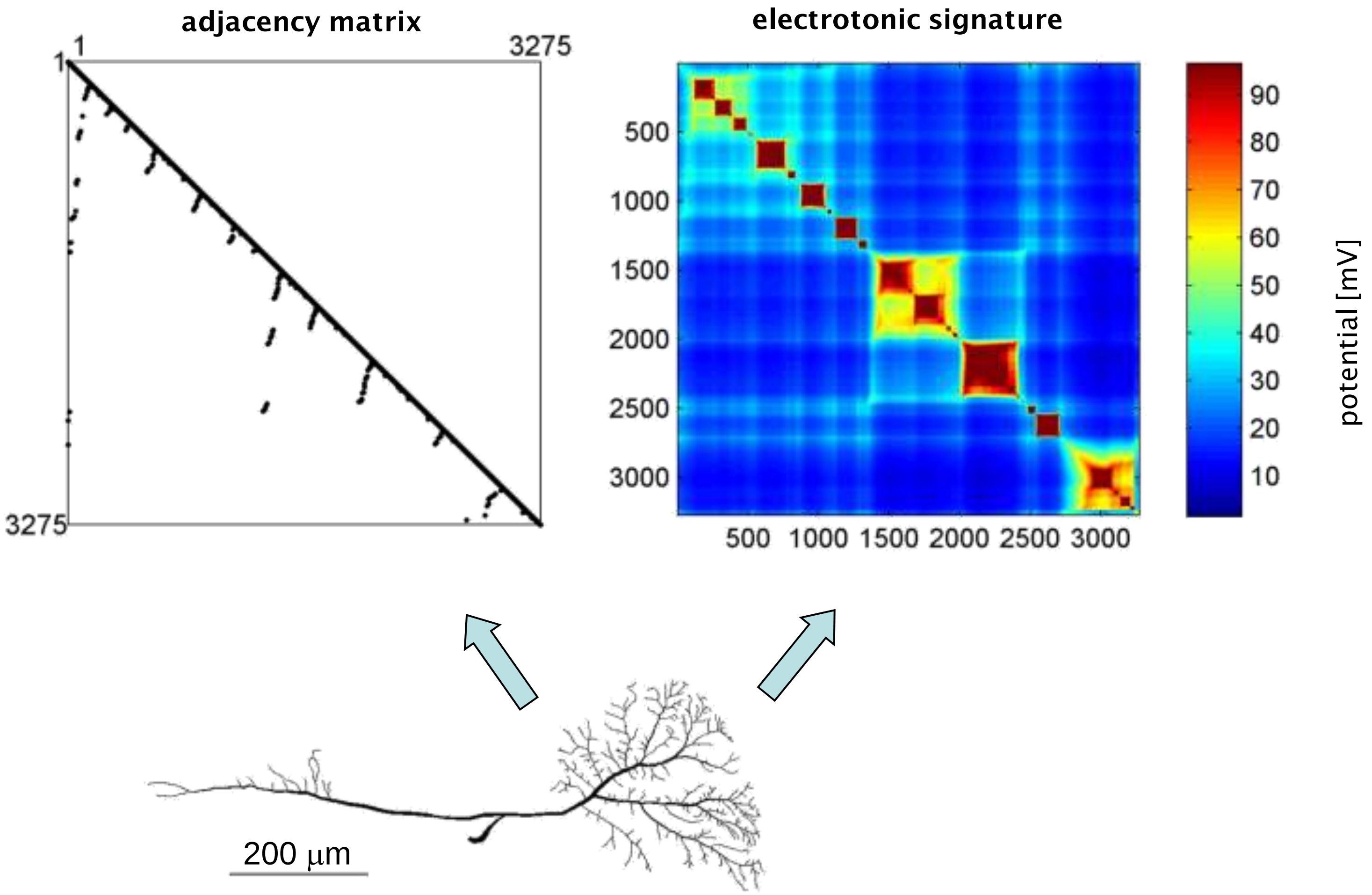

Definitions resampling a tree

By redistributing nodes on a tree structure such that segments are constant length, node locations become unique (not arbitrary) and trees can be simplified.

loss resampling **length conservation** **zig-zag resampling (not implemented yet)**

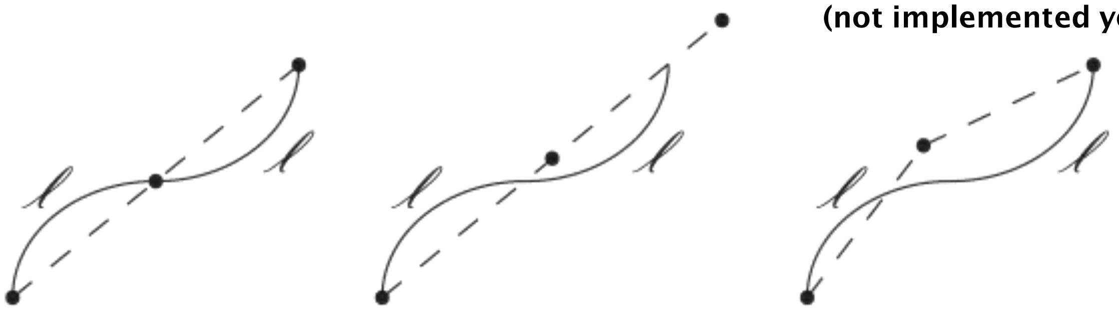

The direct comparison of two trees along strict criteria requires (apart from a unique label distribution) a unique distribution of node locations on the graph. The process of manual reconstruction attributes node locations in an arbitrary manner. However, nodes can be redistributed on the same tree structure assigning homogeneous inter-nodal distances, a process we term resampling (see “resample_tree”). Resampling compromises either the total length (see **loss resampling**) or the shape of the neuronal tree (see **length conservation**) when undersampling. This is because a tortuous path is simplified by a straight line (always shorter). If the length is conserved then the shape of the neuronal tree is altered (the tree spannning field becomes larger, see **length conservation**). A zig-zag implementation of resampling would best remediate this but would also alter the original shape of the tree (and was not implemented).
The resulting electrotonic signature or BCT string will then be entirely independent of the reconstruction procedure. Furthermore, simplified tree structures, which preserve the electrotonic compartmentalization, can be obtained. Computing current flow in a corresponding model will be much faster since the number of nodes is decreased drastically (from 297 to 39 in the example on the far right).

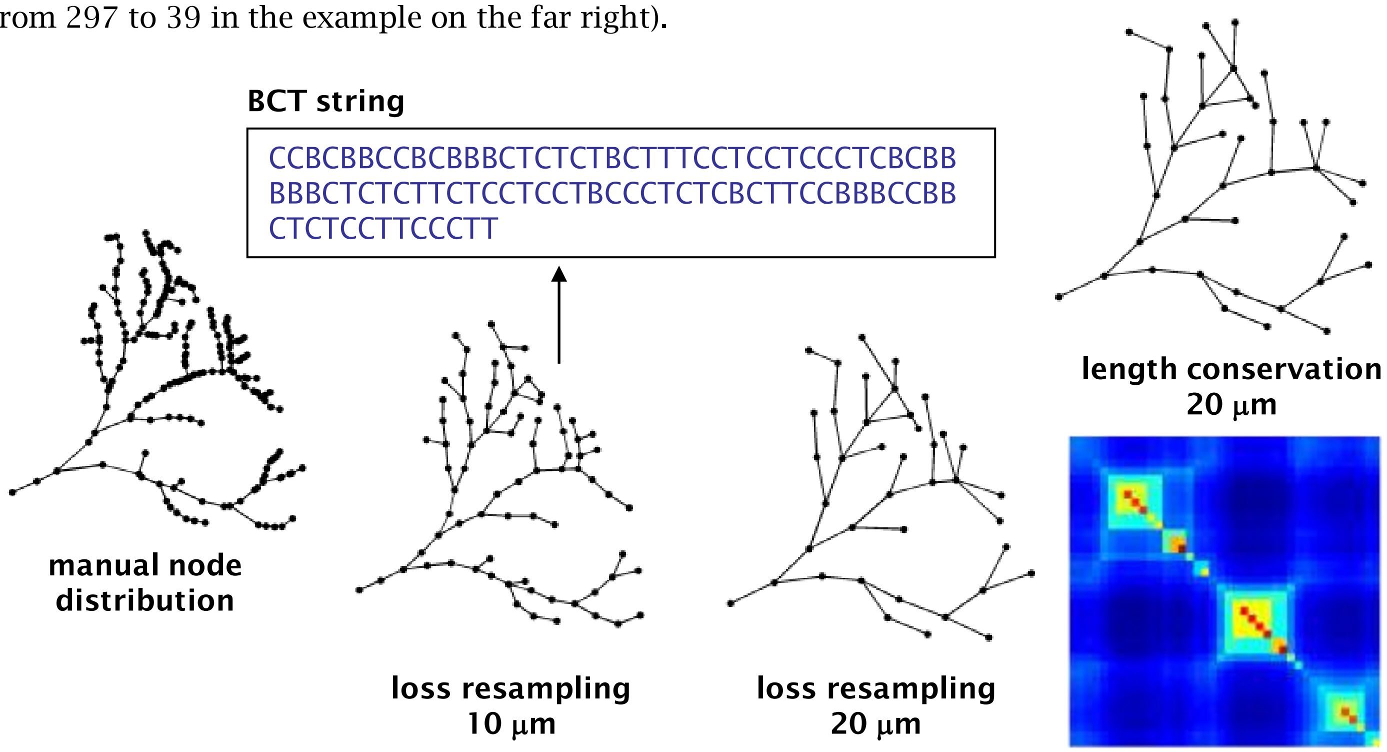

A greedy algorithm can be implemented which optimizes locally total wiring and path length to the root inspired by Cajal's laws of conservation of material and conduction time. This represents an extension to the minimum spanning tree (MST) algorithm.

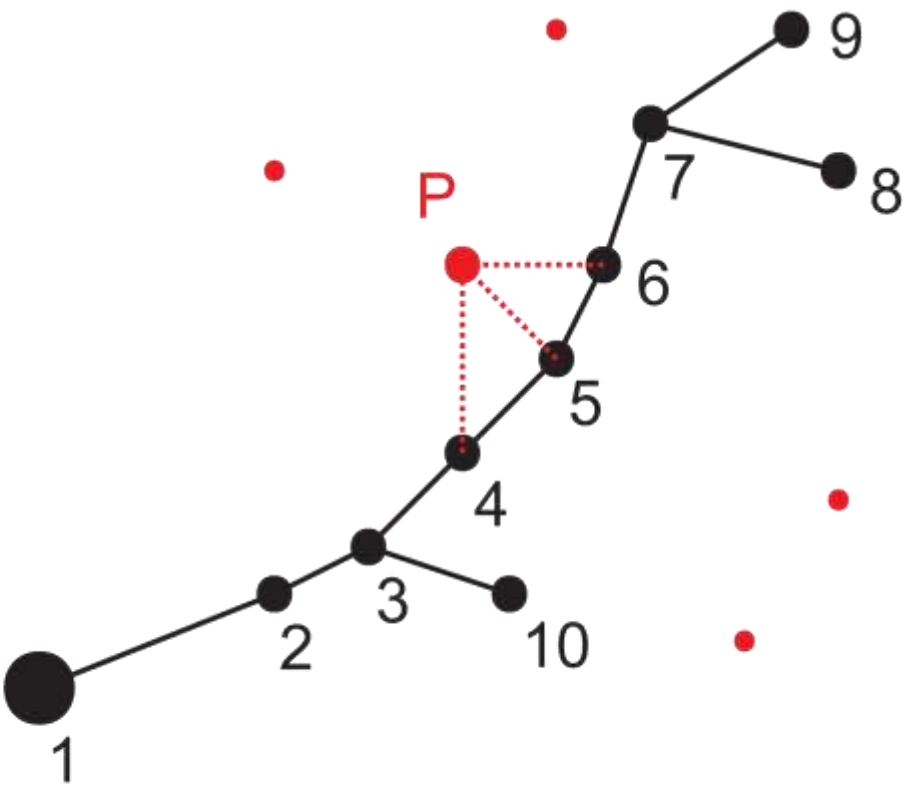

The figure above exemplifies the general approach to obtain a locally optimized graph. In the process, unconnected carrier points (red dots) connect one by one to the nodes of a tree (black dots). At each step, the unconnected carrier point, which is closest to the tree according to some cost function, connects to the node in the tree to which it is closest. The distance cost in this case is composed of two components inspired by Ramón y Cajal's laws of neuronal branching: 1. the wiring cost corresponding to the Euclidean distance to the node in the tree (red dashed lines show three sample segment distances for carrier point P); 2. the conduction time cost, corresponding to the path length from the root (large black node) to the carrier point P. In the example here, even though P is closer to node 5 in Euclidean terms, the additional cost of path length (adding distance between node 4 and node 5) might tip the balance in favour of node 4. A balancing factor *bf* weighs these two cost functions against each other (see "MST_tree").

Definitions MST rule (II)

One parameter, the balancing factor *bf*,
determines the formants of potential trees

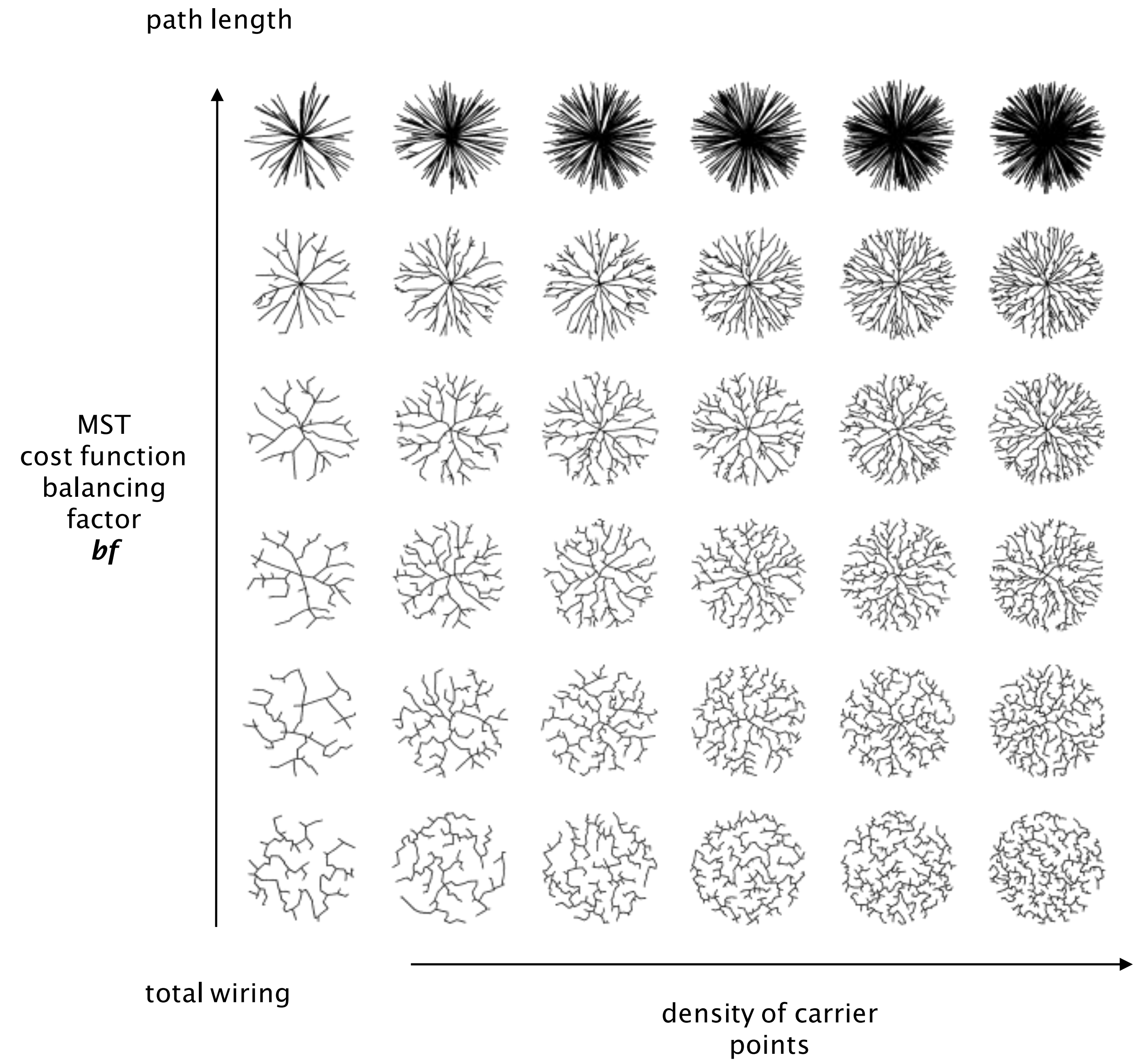

This approach produces realistic neuronal branching structures in all cases. The balancing factor between the two costs determines the electrotonic compartmentalization of the neuronal tree. At one extreme, one finds the pure minimum spanning tree, at the other, the entirely compartmentalized stellate structure, which connects each carrier point directly to the root.

Optimizing diameter values for an equal current transfer to the root (synaptic democracy) results in a quadratic taper. The latter can be mapped on a neuronal tree.

A quadratic diameter taper optimizes current transfer in cables from any point to the root (Cuntz, Borst and Segev 2007, Theor Biol Med Model, 4:21, see right figure). For different cable lengths, different parameter sets can be derived to fit the quadratic equation with distance x:

$y = P_1 x^2 + P_2 x + P_3$.

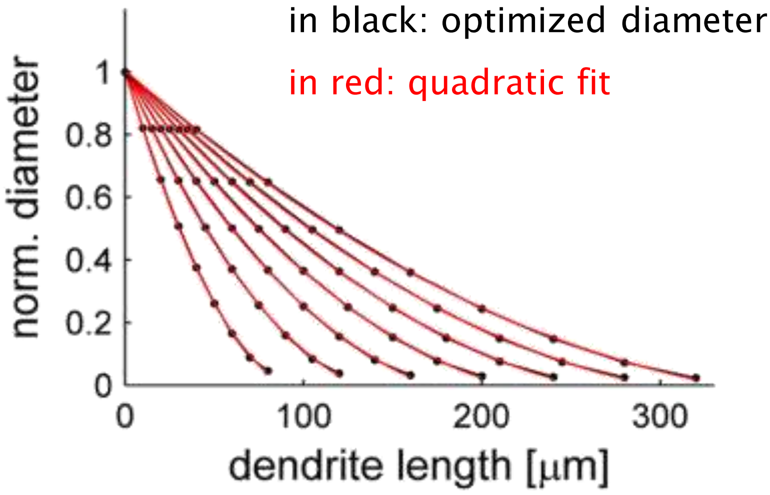

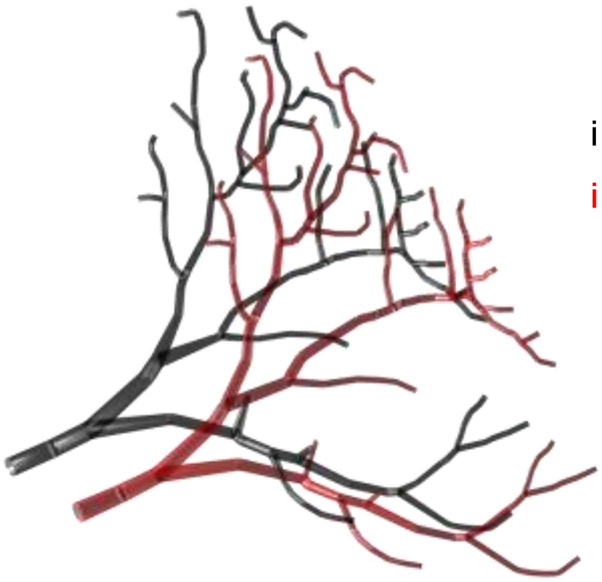

in black: original tree

in red: mapped quadratic diameter taper

Each single triplet P corresponds to the best fit to a segment of a specific cable length l_{dend}. In order to map a quadratic diameter to a full tree, each path from terminal to the root is compared to its closest value in a predetermined set of l_{dend}. Then the quadratic equation parameters P are chosen according to l_{dend}. This is done for all paths from root to terminal points and for each node the diameter is set to the average of all local diameters of all paths leading through that node (see “quaddiameter_tree”). P and l_{dend} depend on the total leak and the minimal diameter: these have to be adjusted by the parameters scale and offset respectively (see “quadfit_tree”). The resulting tree diameter mapping compares well with the original even though it is set merely by two parameters, the scale and offset values.

Definitions *Nx1* vectors

Nx1 vectors are vectors which attribute a value to each node of the tree

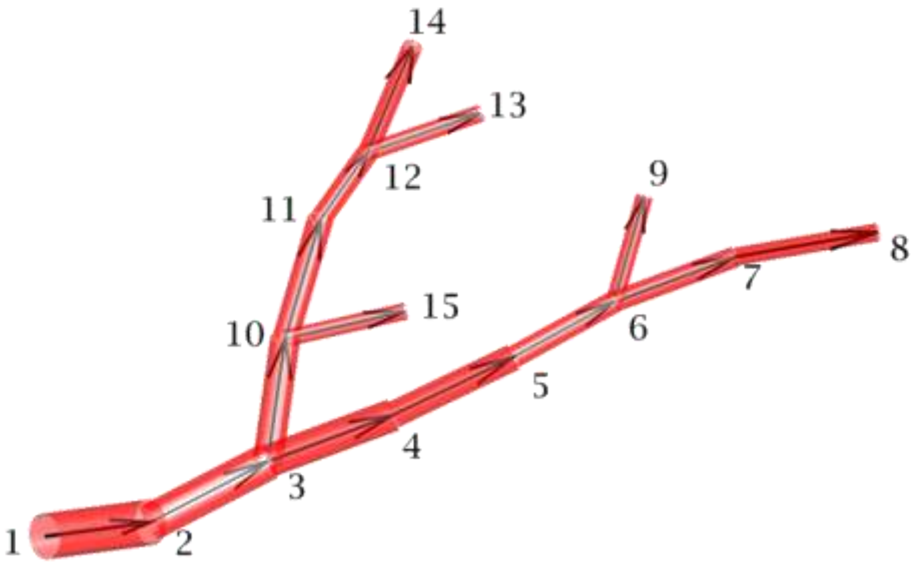

		1 2 3 4 5 6 7 8 9 1 1 1 1 1 1<br>0 1 2 3 4 5
**B_tree**	Branch point 1, other 0	001001000101000'
**C_tree**	Continuation point 1, other 0	110110100010000'
**T_tree**	Termination point 1, other 0	000000011000111'
**typeN_tree**	C, 1: Continuation, B, 2: Branch,<br>T, 0: Termination	112112100212000'<br>CCBCCBCTTBCBTTT'
**PL_tree**	topological path length to the root	012345676345664
**BO_tree**	branch order	000111222122332

Here is a sample overview of the outputs of some TREES toolbox functions whose form is an *Nx1* vector, attributing thereby one value to each node.

Obtaining some statistics

Here are some example statistical measures of neuronal trees and the ways to obtain them using the TREES toolbox

GLOBAL PROPERTIES

width, height, depth and much more..
spanning = gscale_tree ({tree})

total cable length
sum (len_tree (tree))

total membrane surface
sum (surf_tree (tree))

terminal density
M = gdens_tree (tree, sr, …
T_tree (tree))

number of branches
sum (B_tree (tree))*2+1

number of tips (related measure…)
sum (T_tree (tree))

HULL/AREA PROPERTIES

surface
[a b c area] = vhull_tree (tree,…
[],[],[],[],'-2d'); sum (area)

volume
[a b c volume] = vhull_tree (tree);
sum (volume)

IN GENERAL

stats_tree and gscale_tree can be helpful

COMBINATIONS

Be creative…

BRANCH STATISTICS/DISTRIBUTIONS

diameter
tree.D

segment membrane surfaces
surf_tree (tree)

segment volumes
vol_tree (tree)

branch order
BO_tree (tree)

euclidian distance
eucl_tree (tree)

path distance
Pvec_tree (tree)

terminal diameters
tree.D (T_tree (tree))

sholl analysis
sholl_tree (tree)

RATIOS

branch asymmetry
e.g. asym_tree (tree)

tapering
e.g. ratio_tree (tree, tree.D)

distance to root path/Euclidian
Pvec_tree (tree) / eucl_tree (tree)

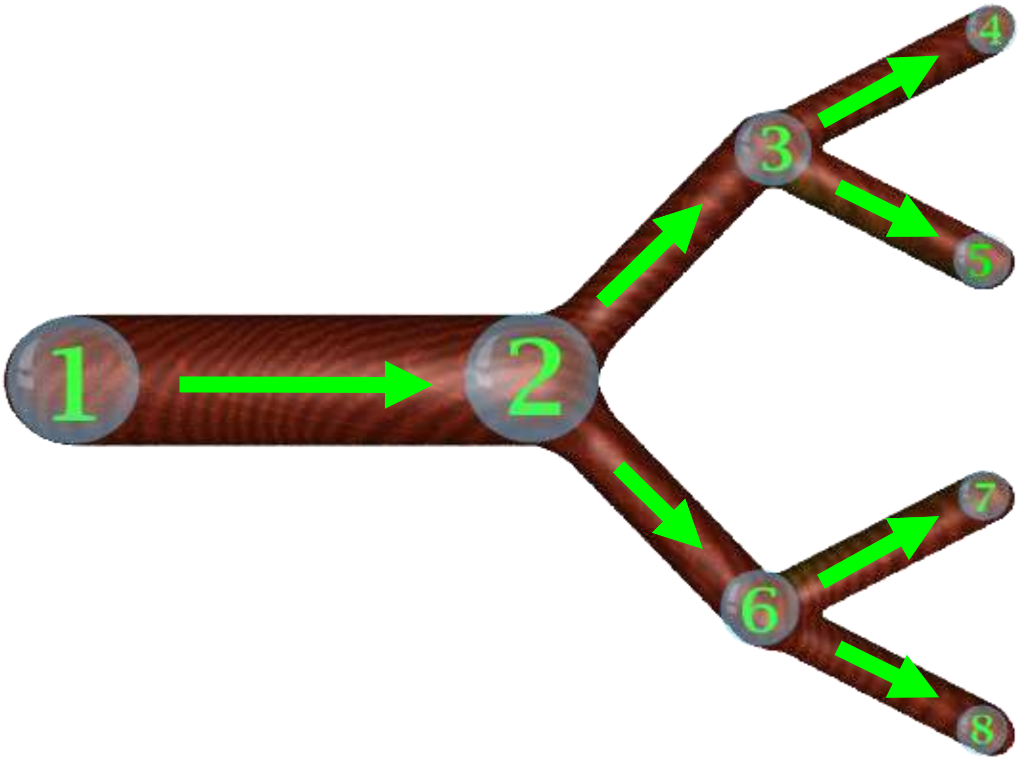
1
2
3
4
5
6
7
8

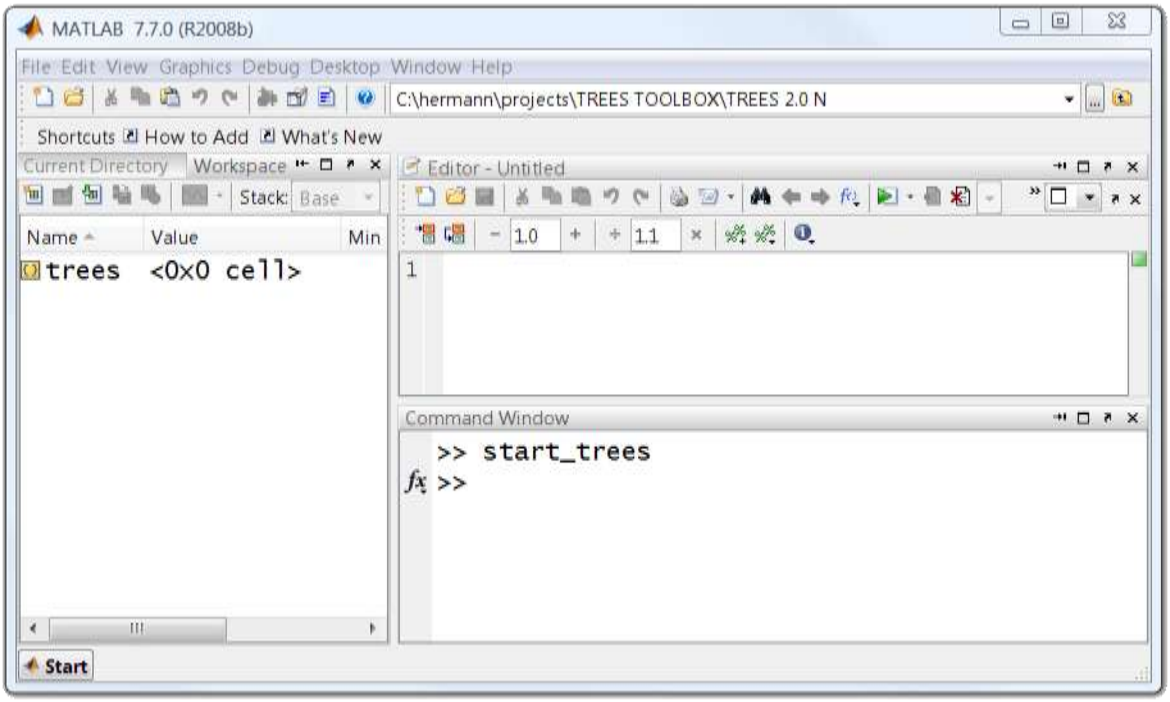

Simply unzip the TREES package obtained from www.treestoolbox.org unto your computer and change directory to its parent folder after opening Matlab. Set the path and create a global empty cell array called „trees“ by typing „start_trees“ in the command window.

By default, most functions append new trees to this cell array „trees“. Try out:

```
>> load_tree
```

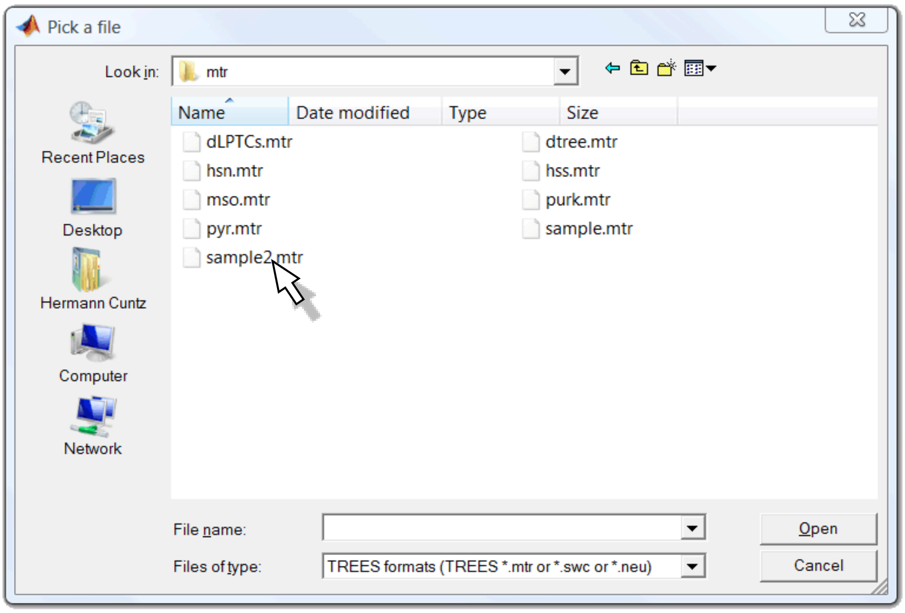

which opens a fileselect. You can find some sample trees in „.\sample\mtr\“. We will start by loading the tree called „sample2.mtr“.

The tree was appended to the cell array „**trees**“:

```
>> trees

trees =

    [1x1 struct]
```

as a structure with the following organization:

```
>> trees{1}

ans =

        dA: [15x15 double]
         X: [15x1 double]
         Y: [15x1 double]
         Z: [15x1 double]
         R: [15x1 double]
         D: [15x1 double]
    rnames: {'1'  'dendrite'}
        Ri: 100
        Gm: 5.0000e-004
        Cm: 1
      name: 'tree1'
```

```
>> trees{1}.dA

ans =

   (2,1)        1
   (3,2)        1
   (4,3)        1
  (10,3)        1
   (5,4)        1
   (6,5)        1
   (7,6)        1
   (9,6)        1
   (8,7)        1
  (11,10)       1
  (15,10)       1
  (12,11)       1
  (13,12)       1
  (14,12)       1
```

```
>> trees{1}.D

ans =

    4.2506
    3.4969
    2.8540
    2.4839
    2.0405
    1.6907
    1.6019
    1.3737
    1.2672
    2.2141
    1.9202
    1.6343
    1.3279
    1.4731
    1.2903
```

It contains the *NxN* adjacency matrix „***dA***“ in sparse form which describes the edges between the *N* nodes of the graph. A few *Nx1* vectors attribute individual values to all nodes (e.g. „***X***“, „***Y***“, „***Z***“ coordinates; „***R***“ region index; „***D***“ diameter values). A cell array of strings „***rnames***“ attributes a name to each region. A few single values describe homogenously distributed features, here the passive electrotonic parameters. A string „***name***“ attributes a name to the given tree.

First steps   visual exploration

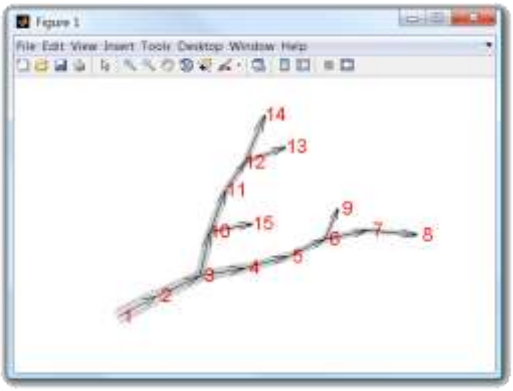

Many ways were implemented to explore the tree visually, for example:

>> xplore_tree; axis off;

of course this is equivalent to „xplore_tree (1)“ or „xplore_tree (trees{1})“or „xplore_tree ([])“.

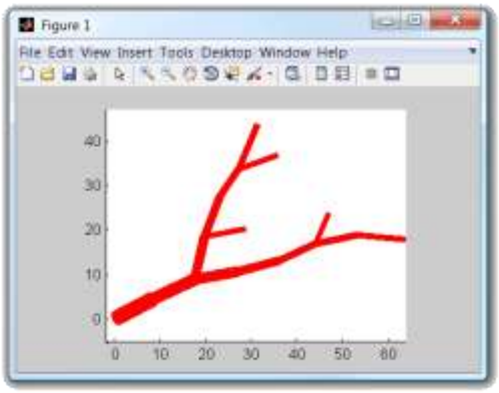

But the most common function will be:

>> plot_tree (1, [1 0 0]);

which takes a color as a first argument, here RGB: red.

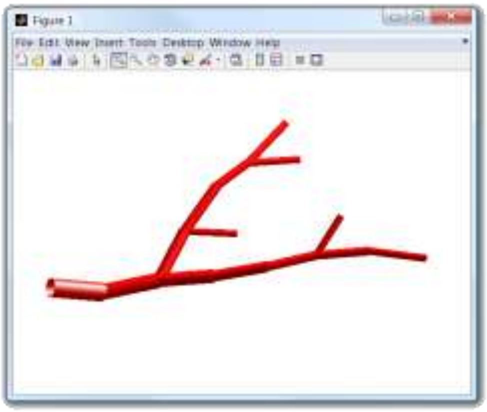

This is all in 3D of course. This becomes clearer when adding light (turning on opengl) and adopting another view:

>> shine; axis off; view (15,55)

Importantly, the color can also be an *Nx1* vector which is then mapped to the color of the tree:

```
>> plot_tree (1, BO_tree (1));

>> colorbar
```

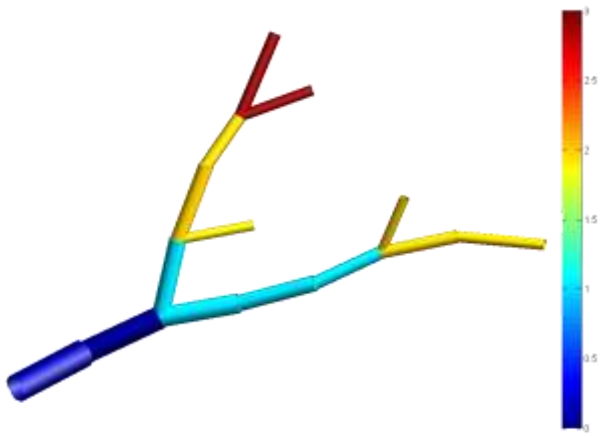

In order to get more information about a function check out the reference (in the end of this manual) or type “help function-name”, for example here we might want to know what “BO_tree” does:

```
>>  help BO_tree

  BO_TREE   Branch order values in a tree.
  (trees package)

  BO = BO_tree (intree, options)
  ------------------------------

  returns the branch order of all nodes referring to the first node as the
  root of the tree. This value starts at 0 and increases after every branch
  point.

  Input
  -----
  - intree::integer:index of tree in trees or structured tree
  - options::string: {DEFAULT: ''}
      '-s' : show

  Output
  ------
  BO::Nx1 vector: vector of branching order values

  Example
  -------
  BO_tree (sample2_tree, '-s')

  See also PL_tree LO_tree
  Uses ver_tree typeN_tree dA
```

The reference contents

graphtheory basic topological readout

edit edit topology of a tree

metrics readout or change tree metrics

graphical visual output and various hulls

construct generation of artificial trees

electrotonics calculate current flow in a tree

IO export and import in various formats

scheme non-TREES related dependencies

stacks handling of image stacks

sample sample trees and image stacks

GUI user interface for tree reconstruction

For more information on each function please type „help functionname" in the matlab command or have a look at the code directly. Usually the code is very simple and commented.

press here to get to the function list

graph theory
asym_tree
B_tree
bin_tree
BO_tree
C_tree
child_tree
dissect_tree
dist_tree
gene_tree
idpar_tree
ipar_tree
LO_tree
PL_tree
Pvec_tree
ratio_tree
redirect_tree
rindex_tree
sort_tree
sub_tree
T_tree
typeN_tree

edit
cat_tree
delete_tree
elim0_tree
elimt_tree
insert_tree
insertp_tree
recon_tree
repair_tree
resample_tree
root_tree

metrics
angleB_tree
cvol_tree
cyl_tree
dstats_tree
eucl_tree
flatten_tree
flip_tree
len_tree
morph_tree
rot_tree
scale_tree
sholl_tree
surf_tree
stats_tree
tran_tree
vol_tree
zcorr_tree

graphical
chull_tree
dA_tree
dendrogram_tree
gdens_tree
hull_tree
lego_tree
plot_tree
plotsect_tree
pointer_tree
spread_tree
vhull_tree
vtext_tree
xdend_tree
xplore_tree

construct
allBCTs_tree
BCT_tree
clean_tree
clone_tree
cplotter
cpoints
gscale_tree
in_c
isBCT_tree
jitter_tree
MST_tree
quaddiameter_tree
quadfit_tree
rpoints_tree
smooth_tree
smoothbranch
soma_tree
spines_tree

electrotonics
elen_tree
gi_tree
gm_tree
lambda_tree
loop_tree
M_tree
sse_tree
ssecat_tree
syn_tree
syncat_tree

IO
load_tree
neurolucida_tree
neuron_tree
pov_tree
save_tree
swc_tree
ver_tree
x3d_tree
neu_tree

scheme
deg2rad
eucdist
gauss
rad2deg
rotation_matrix
roundshow
scalebar
Shine
tprint
gifmaker

stacks
fitD_stack
imload_stack
load_stack
loaddir_stack
loadtifs_stack
save_stack
show_stack
skel_stack

samples
GUI

The suffix „_tree“ is usually appended to indicate that a function belongs directly to the TREES toolbox. An input tree ***intree*** is generally the first argument which is passed on (this proved to be more comfortable in most cases). This first argument ***intree*** can be a tree structure or an index (single value) to the global cell array ***trees***. If the first input is omitted (or „[]“) the last entry in the ***trees*** array is used.

In general, omitting an input argument by typing in the empty vector „[]“ or by simply sending out too few arguments to a function will result in replacing the input arguments by default values. These default values are indicated precisely in the headers of each function (simply typing „help function_name“ will retrieve the header) and in the code. For most functions only a subset of the full tree definition is used (e.g. only the diameter values, only the *X* and *Y* coordinates or only the topology). In those cases the functions will not complain if the tree is not complete but the required fields are existent in the tree structure. Missing fields might even be replaced: the „plot_tree“ function for example will attribute sensible fake metrics if real ones are missing.

In most cases the last input argument to a TREES function is the ***options*** string. This string contains concatenated flags starting with „-“. Examples of typical options are:

‚-s‘	show the result, this is mostly for demo purposes
‚-m‘	demonstration movie in very few cases
‚-w‘	waitbar to indicate the progress of long-lasting calculations
‚-e‘	echo changes made to the tree

Note that if ***options*** is left empty (‚‘) default options will be used rather than all flags off. To be sure that all flags are off set ***options*** string to ‚none‘. Note also that when demo flags are on, features of a tree as well as other TREES toolbox functions might be required which are not required when the flags are off.

Meta-functions are generalized functions whose input of an *Nx1* vector can vary their application greatly (see introductary explanation of morphing a tree for one such example).

Examples are not necessarily useful but try to also exemplify typically more unintuitive applications. Output values and resulting plots are formatted and do not always correspond to the correct output (e.g. rounded values) of the Matlab function.

graphtheory

functions relating to the tree as a graph

asym_tree	branch point asymmetry
B_tree	branch points
bin_tree	binning nodes
BO_tree	branch order values
C_tree	continuation points of tree
child_tree	add up child node values
dissect_tree	groups nodes belonging to same branch
dist_tree	nodes at a path distance away from root
gene_tree	string describing tree topology
idpar_tree	index to direct parent node
ipar_tree	path to root: parent indices
LO_tree	level order values
PL_tree	topological path length
Pvec_tree	cumulative summation along paths
ratio_tree	parent to daughter ratio
redirect_tree	set root to new point and redirect graph
rindex_tree	region specific indexation
sort_tree	relabel nodes after sorting
sub_tree	child nodes forming a subtree
T_tree	termination points
typeN_tree	number of daughters to each node (BCT)

asym_tree branch point asymmetry

asym = asym_tree (intree, v, options)

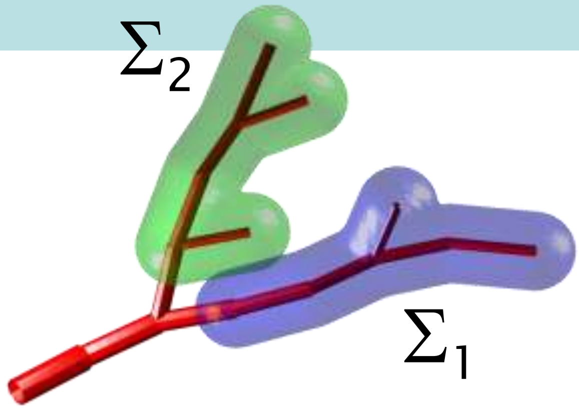

At each branch point of tree ***intree***, node values of *Nx1* vector ***v*** get summed up in each sub-tree to Σ_1 and Σ_2. ***asym***, an *Nx1* vector, contains the ratio of Σ_1 / (Σ_1 + Σ_2) for $\Sigma_1 < \Sigma_2$ at branch points (but NaN otherwise).

By default, number of terminal child nodes are compared in both sub-trees at a branch point: ***v*** is a vector of 1 when termination point and 0 else (see „T_tree“). For the example branch point on the right (at node 3), Σ_1=2 terminals are divided by $\Sigma_1 + \Sigma_2$ = 2+3 terminals (= 0.4).

intree must be BCT!!

Example:

>> asym_tree (sample2_tree, T_tree (sample2_tree))‘

[NaN, NaN, 0.4, NaN, NaN, 0.5, NaN, NaN, NaN, 0.3333, NaN, 0.5, NaN, NaN, NaN]

See demo movie with option ‘-m‘

B_tree branch points

B = B_tree (intree, options)

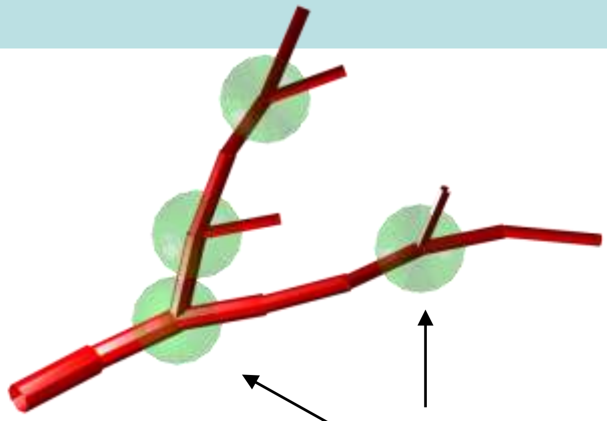

Returns for tree ***intree*** an *Nx1* vector ***B*** which is one if a given node is a branch point (more than 1 daughter node) and zero else.

Example:

```
>> B = B_tree (sample2_tree)'

[0, 0, 1, 0, 0, 1, 0, 0, 0, 1, 0, 1, 0, 0, 0]

>> sum (B)
```
⇨ number of branch points

```
4

>> find (B)
```
⇨ indices of branch points

```
[3, 6, 10, 12]
```

bin_tree binning nodes

[bi, bins, bh] = bin_tree (intree, v, bins, options)

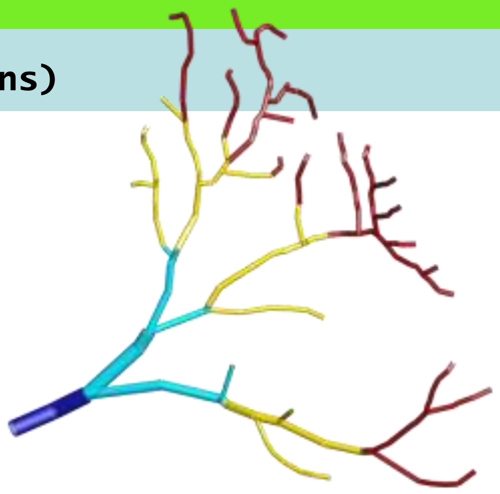

Subdivides nodes of tree ***intree*** into bins ***bins*** (number, by default 10, or exact binning values) according to *Nx1* vector ***v***. By default, ***v*** is the euclidean distance to the root (see "eucl_tree").

bi outputs an *Nx1* vector with affiliation of each node to used bins ***bins***. ***bh*** counts up the number of occurrences in each bin.

Examples:

>> [bi bins bh] = bin_tree (sample_tree, [], 4)

>> bar (bh)

plotting a histogram results in a rudimentary sholl analysis plot

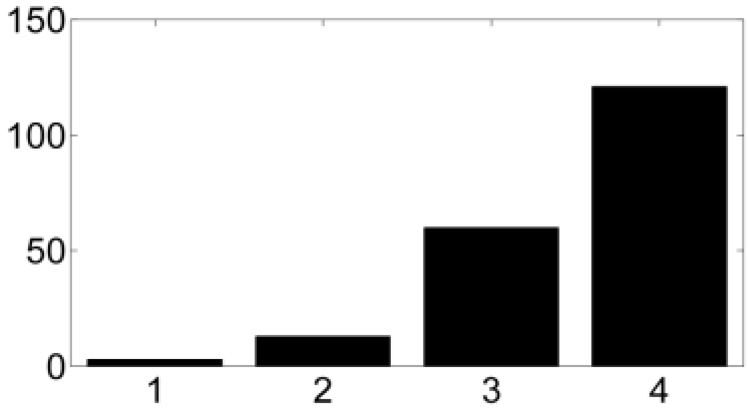

meta-function

BO_tree branch order values

BO = BO_tree (intree, options)

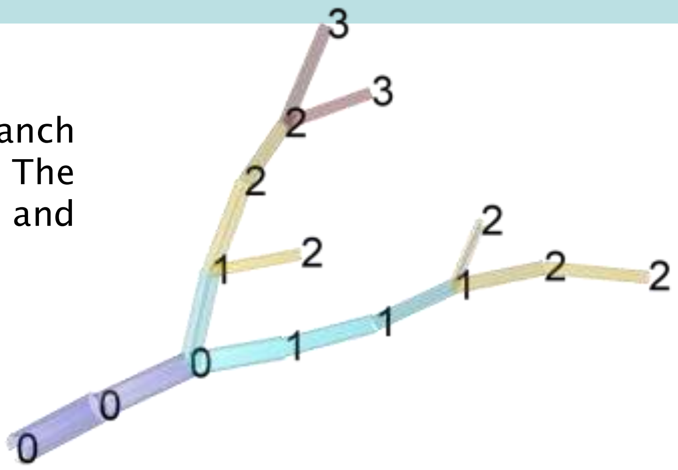

Returns an *Nx1* vector ***BO*** attributing a branch order value to each node of tree ***intree***. The branch order starts at *0* at the root of the tree and increases after every branch point.

Examples:

```
>> BO = BO_tree (sample2_tree)'

[0, 0, 0, 1, 1, 1, 2, 2, 2, 1, 2, 2, 3, 3, 2]

>> max (BO)
```

⇨ Maximum branch order

```
3
```

```
>> BO2 = BO_tree (sample_tree);

>> hist (BO2);
```

⇨ Plot a histogram of branch orders for all nodes

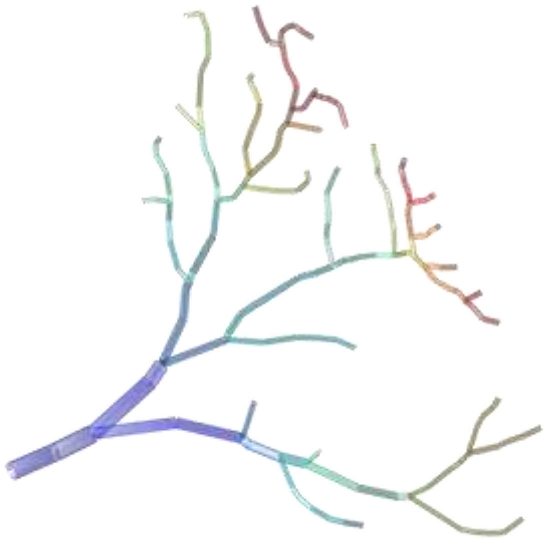

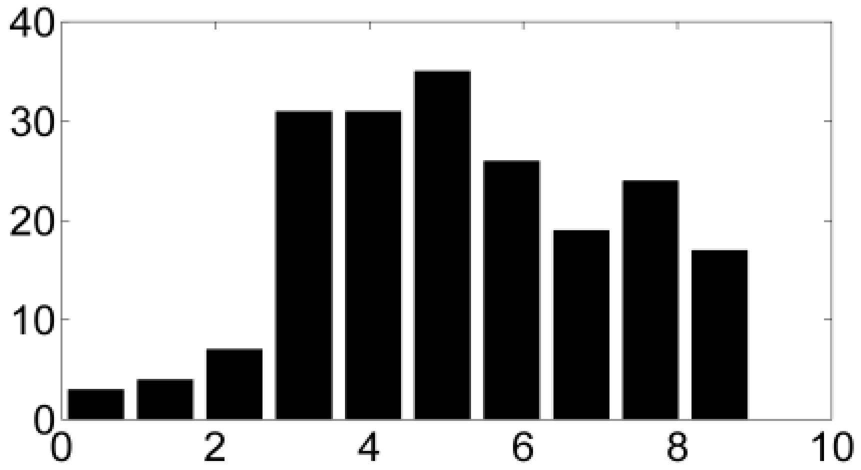

C_tree continuation points

```
C = C_tree (intree, options)
```

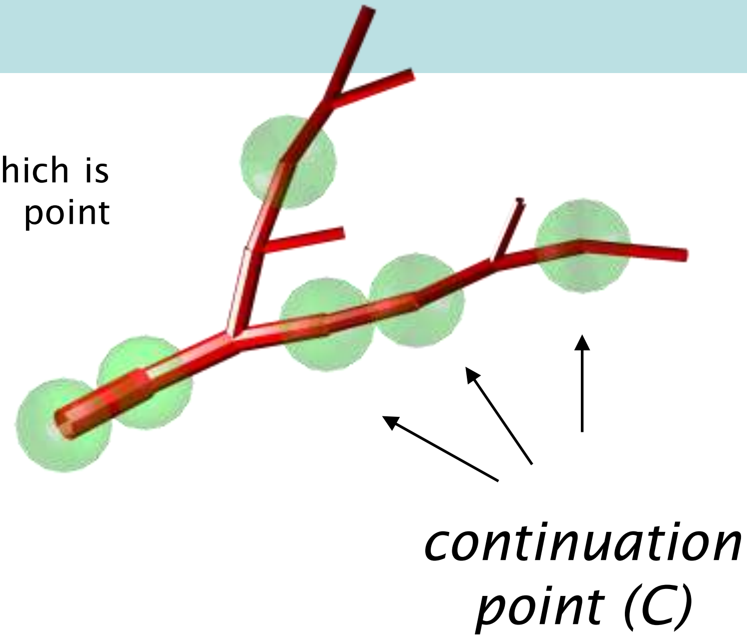

Returns for tree ***intree*** an *Nx1* vector ***C*** which is one if a given node is a continuation point (exactly 1 daughter node) and zero else.

Example:

```
>> C = C_tree (sample2_tree)'
[1, 1, 0, 1, 1, 0, 1, 0, 0, 0, 1, 0, 0, 0, 0]
>> sum (C)
```
⇨ number of continuation points

```
6
>> find (C)
```
⇨ indices of continuation points

```
[1, 2, 4, 5, 7, 11]
```

child_tree add up child node values

child = child_tree (intree, v, options)

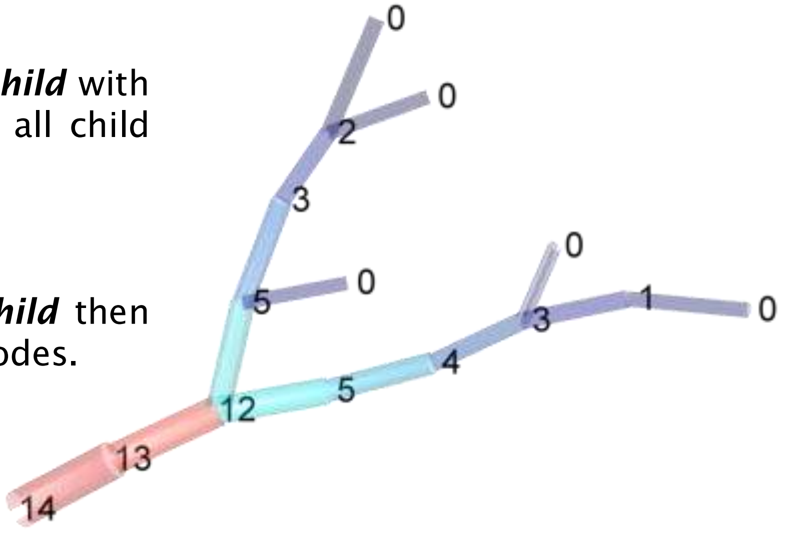

Returns for tree ***intree*** an *Nx1* vector ***child*** with accumulated values of *Nx1* vector ***v*** of all child nodes excluding the node itself.

By default ***v*** is a vector of all ones: ***child*** then simply counts up the number of child nodes.

Examples:

```
>> child = child_tree (sample2_tree)'

[14, 13, 12, 5, 4, 3, 1, 0, 0, 5, 3, 2, 0, 0, 0]
```

or just the number of termination child nodes:

```
>> Tchild = child_tree (sample2_tree, T_tree (sample2_tree))'

[5, 5, 5, 2, 2, 2, 1, 0, 0, 3, 2, 2, 0, 0, 0]
```

meta-function

dissect_tree group nodes to branches

```
[sect vec] = dissect_tree (intree, options)
```

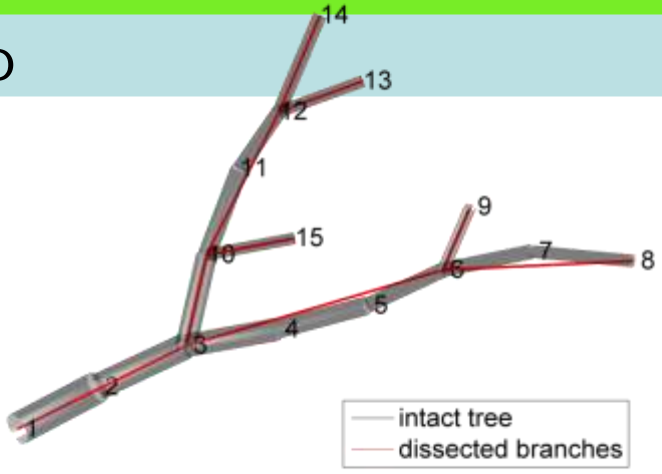

Groups segments of tree ***intree*** together when they belong to the same branch. Can be used as sections in NEURON-like compartmental modelling (see "neuron_tree"). Branches are delimited by either branching or termination points or region-defined borders.

Output matrix ***sect*** of size *nx2* where *n* is the number of branches contains starting and end nodes of each branch. *Nx2* matrix ***vec*** attributes a branch to each node and a fractional path length along this branch.

(Note that in the example below, nodes 14 and 15 form a separate region called "1", which reflects in the NEURON code below.)

Example:

```
>> [sect vec] = dissect_tree (sample2_tree);

>> sect'
```

1	3	6	6	3	10	12	12	10	starting nodes
3	6	8	9	10	12	13	14	15	ending nodes

```
>> vec'
```

1	1	1	2	2	2	3	3	4	5	6	6	7	8	9	segment index
0	.5	1	.35	.7	1	.47	1	1	1	.58	1	1	1	1	fractional path length

corresponding NEURON connectivity:

```
connect tree_dendrite[1](0),tree_dendrite[0](1)
connect tree_dendrite[2](0),tree_dendrite[1](1)
connect tree_dendrite[3](0),tree_dendrite[1](1)
connect tree_dendrite[4](0),tree_dendrite[0](1)
connect tree_dendrite[5](0),tree_dendrite[4](1)
connect tree_dendrite[6](0),tree_dendrite[5](1)
connect tree_1[0](0),tree_dendrite[5](1)
connect tree_1[1](0),tree_dendrite[4](1)
```

dist_tree nodes at distance from root

```
dist = dist_tree (intree, l, options)
```

Returns a binary *Nx1* vector ***dist*** indicating the nodes of tree ***intree***, whose segments cross path distance ***l*** from the root. If ***l*** is a vector of length *n*, ***dist*** becomes an *Nxn* matrix.

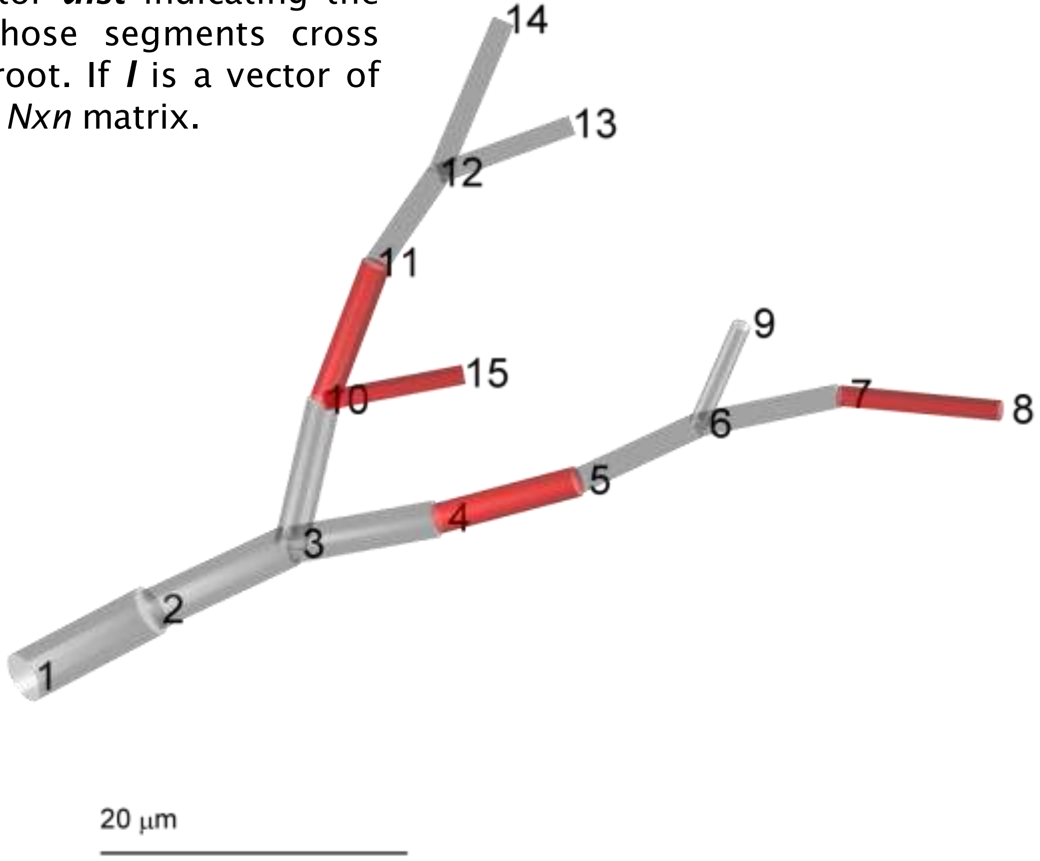

Example:

```
>> dist_tree (sample2_tree, [40 60])'
[0  0  0  0  1  0  0  0  0  0  1  0  0  0  1
 0  0  0  0  0  0  0  1  0  0  0  0  0  0  0]
```

⇨ nodes crossing 40μm
⇨ nodes crossing 60μm

gene_tree "topological gene"

genes = gene_tree (intrees, options)

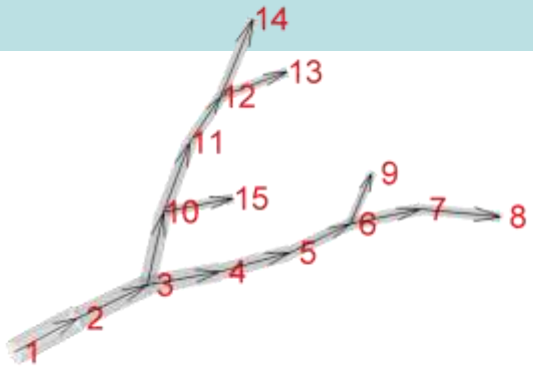

Returns for a cell array of cell arrays of trees ***intrees***, a cell array of cell arrays of topological genes ***genes*** (for each tree one). The two-depth of the input/output arrays allows the comparison between different groups of neuronal trees. The topological gene (see introduction section "topological gene") returns for a sorted labelling of a tree (see "sort_tree") for all branches (delimited by topological points) the metric length and the ending point type (termination or branch).

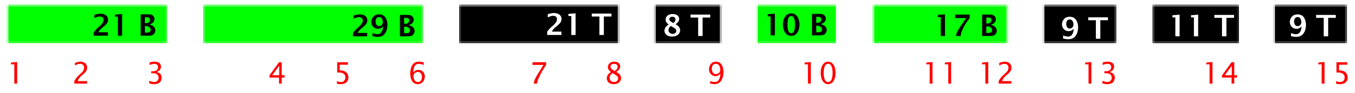

Examples:

```
>> gene = gene_tree({{sample2_tree}}); gene{1}'
```

```
[21 29 21 8 10 17 9 11 9;
  2  2  0 0  2  2 0  0 0]
```

branch length values in μm
branch ending topological point descriptor (0: terminal, 2: branch)

```
>> dLPTCs = load_tree ('dLPTCs.mtr');
```

load groups of tangential cell reconstructions

```
>> genes = gene_tree (dLPTCs, '-s');
```

(this might take some time, 1 min)

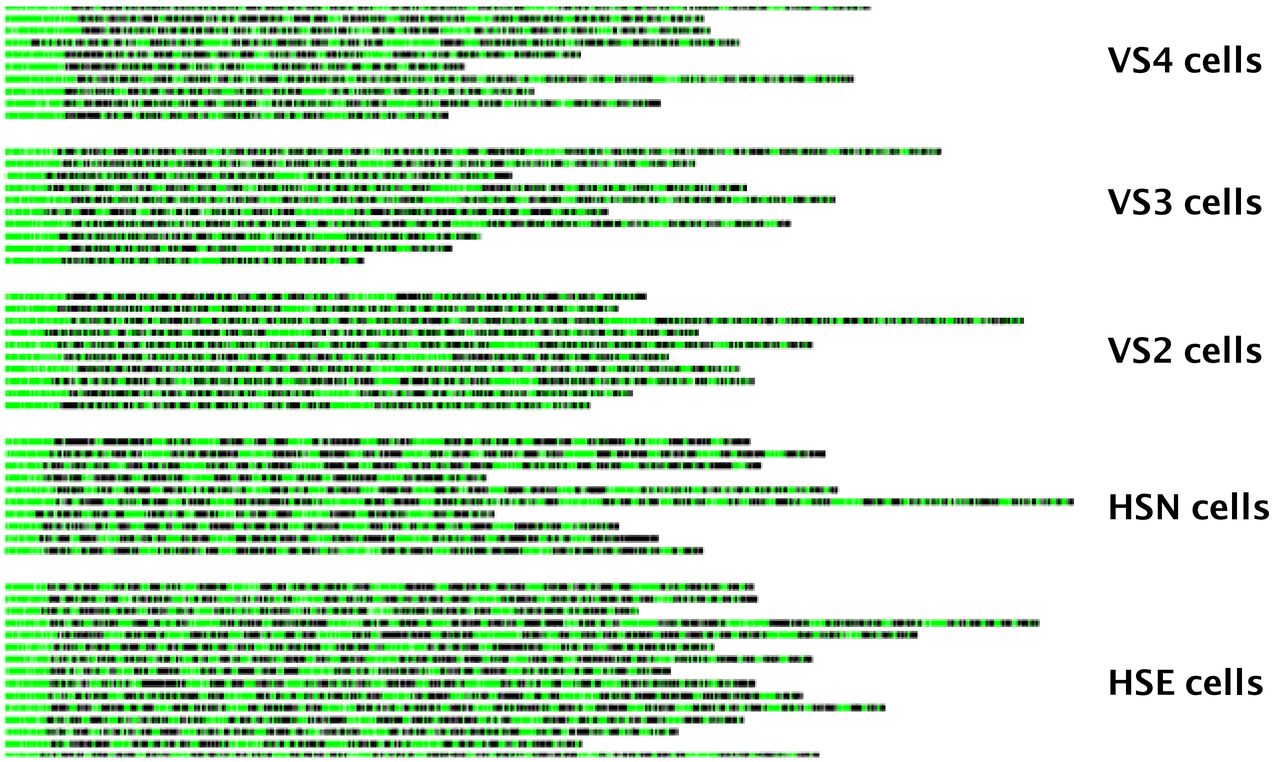

idpar_tree index to direct parent node

idpar = idpar_tree (intree, options)

Returns for tree ***intree*** an *Nx1* vector ***idpar*** containing the direct parent indices for each node. Parent node of root is “1” by default. Use ‘-0’ option to set root parent to “0”.

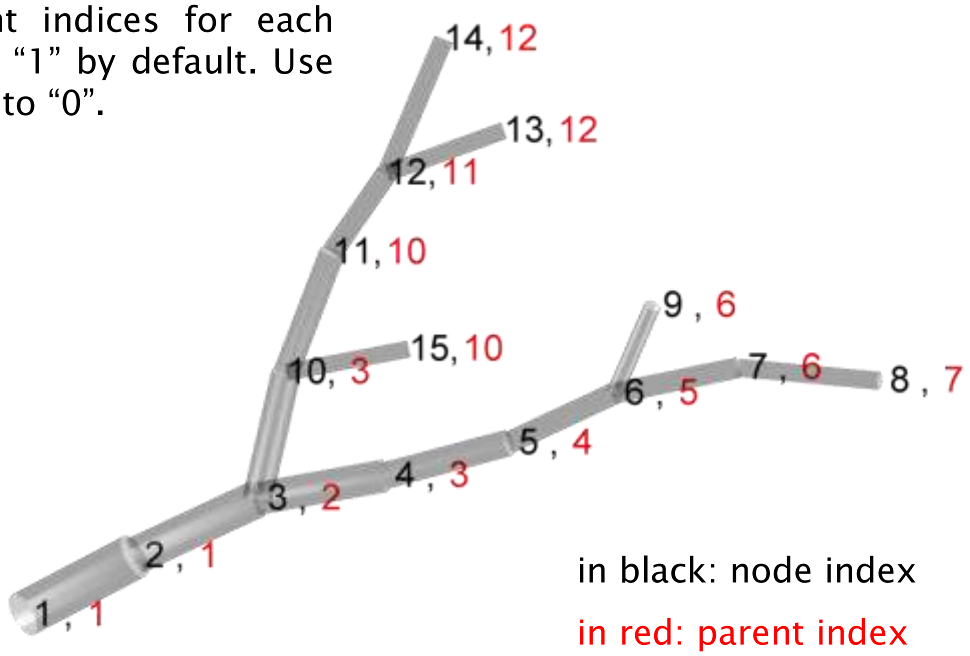

Example:

```
>> idpar_tree (sample2_tree)'

[1, 1, 2, 3, 4, 5, 6, 7, 6, 3, 10, 11, 12, 12, 10]
```

ipar_tree path to root: parent indices

```
ipar = ipar_tree (intree, options)
```

Returns for tree ***intree*** a matrix ***ipar*** of indices to the parent of individual nodes following the path against the direction of the adjacency matrix towards the root of the tree. This function is crucial to many other functions based on graph theory in the TREES package.

Examples:

```
>> ipar_tree (sample2_tree)
```

1	0	0	0	0	0	0	0	0
2	1	0	0	0	0	0	0	0
3	2	1	0	0	0	0	0	0
4	3	2	1	0	0	0	0	0
5	4	3	2	1	0	0	0	0
6	5	4	3	2	1	0	0	0
7	6	5	4	3	2	1	0	0
8	7	6	5	4	3	2	1	0
9	6	5	4	3	2	1	0	0
10	3	2	1	0	0	0	0	0
11	10	3	2	1	0	0	0	0
12	11	10	3	2	1	0	0	0
13	12	11	10	3	2	1	0	0
14	12	11	10	3	2	1	0	0
15	10	3	2	1	0	0	0	0

path of root

path of node #2, etc...

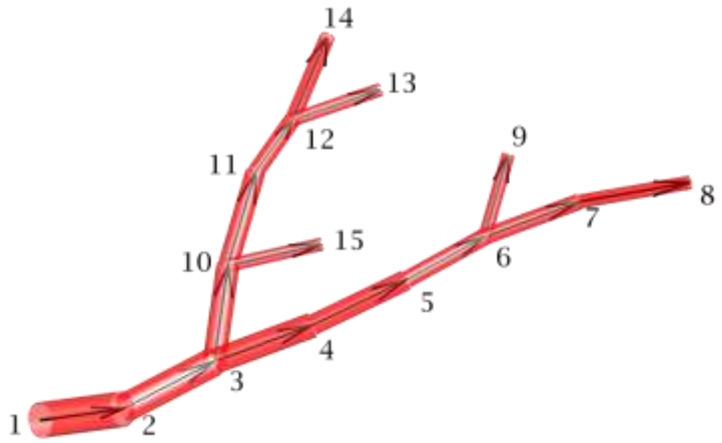


```
>> ipar_tree (sample_tree, '-s')
```

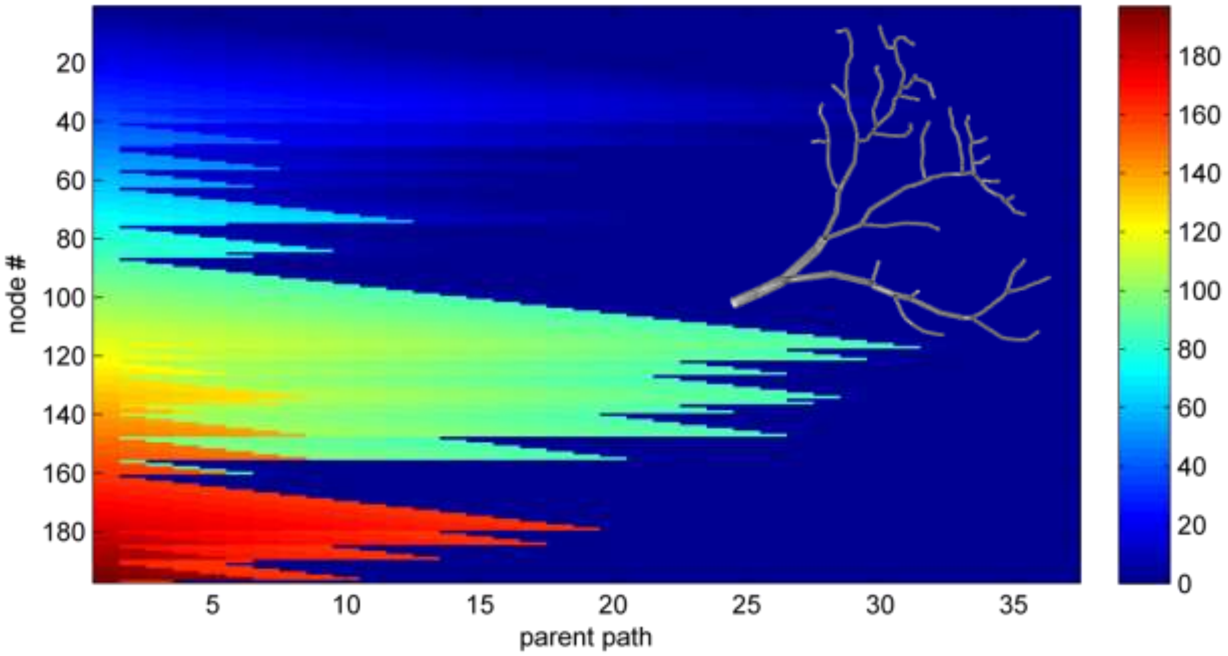

LO_tree level order values

LO = LO_tree (intree, options)

Returns for tree ***intree*** the *Nx1* vector ***LO*** with summed topological path distance (see "PL_tree") of all child branches to the root. We call the function level order and it is useful to classify rooted trees into isomorphic classes, i.e to sort the node labels (see introduction section "sorted and equivalent tree").

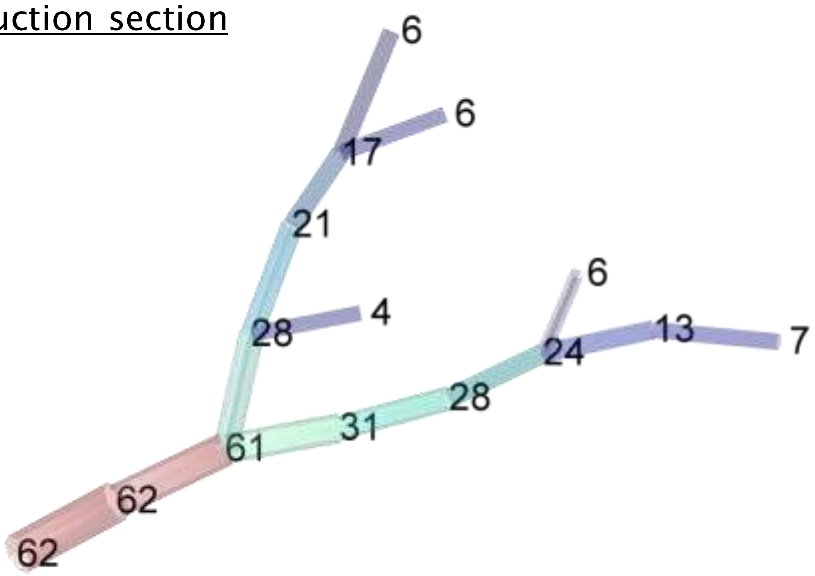

Example:

```
>> LO_tree (sample2_tree)'

[62  62  61  31  28  24  13   7   6  28  21  17   6   6  4]
```

PL_tree topological path length

PL = PL_tree (intree, options)

Returns for tree ***intree*** an *Nx1* vector ***PL*** with topological path length to the root for all nodes.

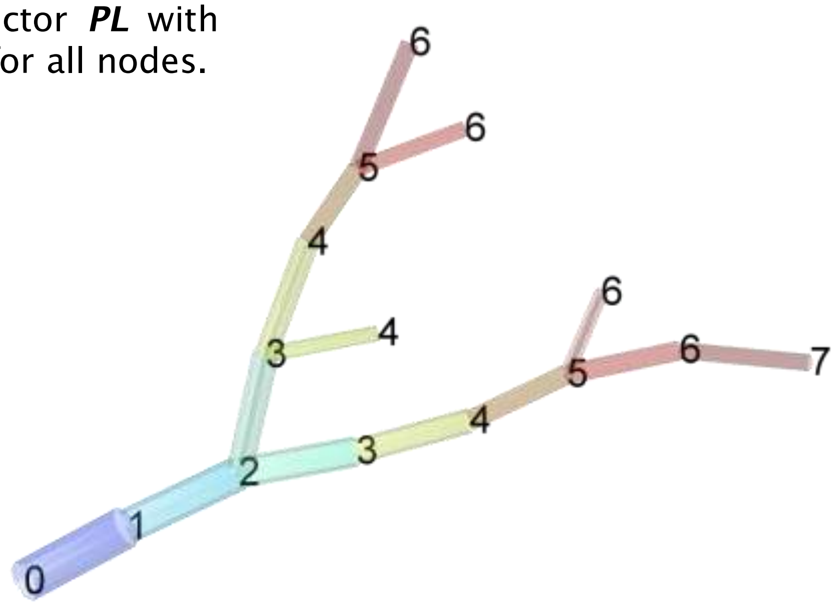

Example:

```
>> PL_tree (sample2_tree)'

[0  1  2  3  4  5  6  7  6  3  4  5  6  6  4]
```

Pvec_tree cumulative summation along paths

Pvec = Pvec_tree (intree, v, options)

Returns for tree ***intree*** an *Nx1* vector ***Pvec*** which accumulates values of *Nx1* vector ***v*** along the path from each node to the root.

By default, „Pvec_tree“ sums up the length values of the segments in the tree: ***v*** is then *len* the vector of segment lengths (see „len_tree“). ***Pvec*** then corresponds to the metric path length to the root [in µm].

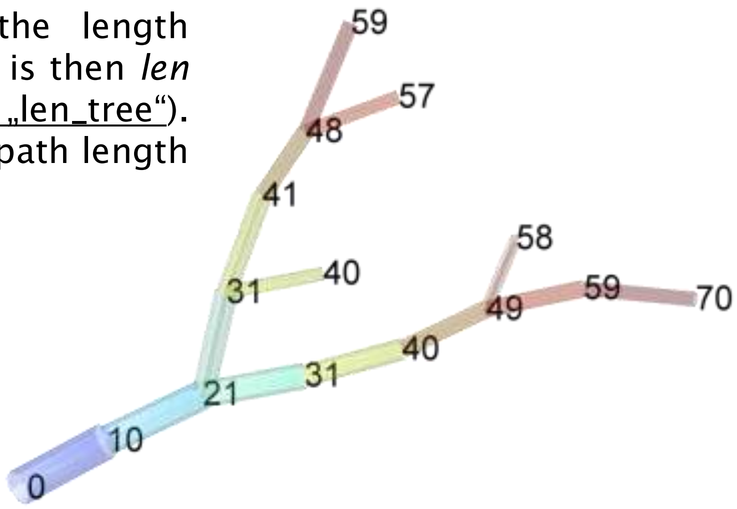

Examples:

```
>> Pvec_tree (sample2_tree)'

[0   10.4   20.6   30.5   40.5   49.3   58.9   69.8   57.6   30.8
41   48.2   57.5   59.2   40.1]
```

when ***v*** contains just ones the output is 1+*PL* the topological path length:

```
>> Pvec_tree (sample2_tree, ones (N, 1))'

[1  2  3  4  5  6  7  8  7  4  5  6  7  7  5]
```

when ***v*** contains the branch points the output is an alternative formulation of the branch order which increases at the branch point itself:

```
>> Pvec_tree (sample2_tree, B_tree (sample2_tree))'

[0  0  1  1  1  2  2  2  2  2  2  3  3  3  2]
```

meta-function

ratio_tree parent to daughter ratio

ratio = ratio_tree (intree, v, options)

Returns for tree ***intree*** an *Nx1* vector ***ratio*** which takes the ratios of values of *Nx1* vector ***v*** at the node itself and its direct parent *idpar*.

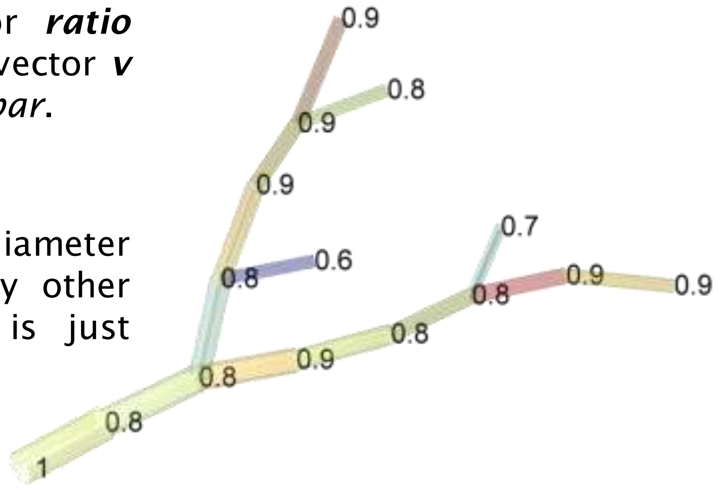

By default, „ratio_tree" compares diameter values: ***v*** is then just *D*. However, any other values can be chosen here. ***ratio*** is just ***v***/***v***(*idpar*).

Example:

```
>> ratio_tree (sample2_tree)'

[1  0.82  0.82  0.87  0.82  0.83  0.95  0.86  0.75  0.78
0.87  0.85  0.81  0.9  0.58]
```

meta-function

redirect_tree set root to new node

```
[tree, order] = redirect_tree (intree, istart, options)
```

Sets the root to a different node. This changes in tree ***intree*** the direction of the adjacency matrix so that arrows show away from node ***istart*** (which becomes the first element).

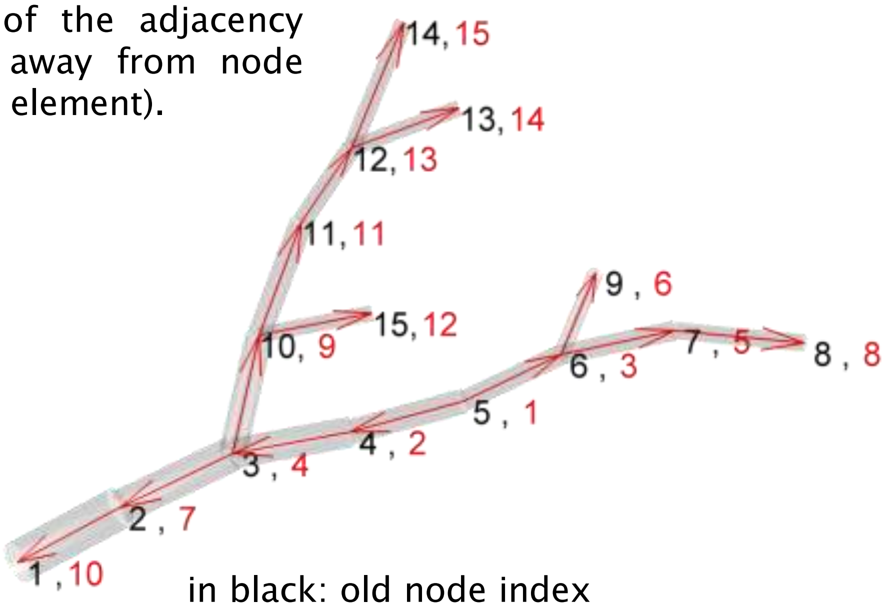

in black: old node index

in red: new node index, after redirect

Example:

```
>> redirect_tree (sample2_tree, 5, '-s');
```

when redirecting on a branch point a trifurcation occurs !!

rindex_tree region specific indexation

rindex = rindex_tree (intree, options)

Returns for tree ***intree*** an *Nx1* vector ***rindex*** attributing to each node a region specific index increasing for each region individually in order of appearance within that region.

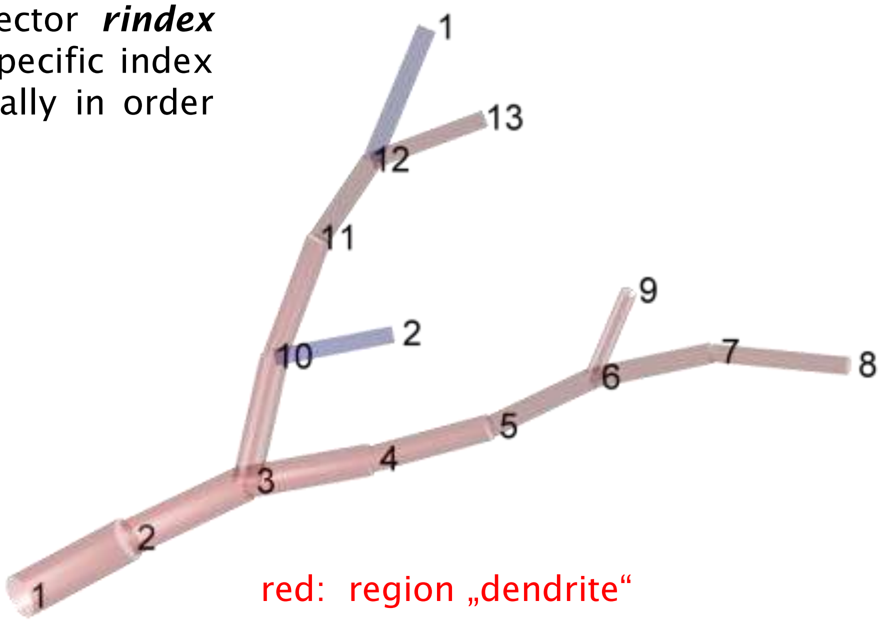

red: region „dendrite“

blue: region „1“

Example:

```
>> rindex = rindex_tree (sample2_tree)’

[1, 2, 3, 4, 5, 6, 7, 8, 9, 10, 11, 12, 13, 1, 2]
```

sort_tree relabel nodes to BCT order

[tree, order] = sort_tree (intree, options)

Sorts the labels (indices) of nodes of tree ***intree*** to conform to BCT, an order in which elements are arranged according to their hierarchy keeping the sub-tree structure intact (<u>see introduction section “sorted and equivalent trees”</u>). Many isomorphic BCT order structures exist, this one is created by switching the location of each node one at a time to the right neighbour position of their parent node. For a unique sorting use '-LO' or ‘-LEX' options.

'-LO' orders the indices using path length and level order. This results in a relatively unique equivalence relation.

'-LEX‘ orders the BCT elements lexicographically. This makes less sense but results in a purely unique equivalence relation.

"sort_tree" affects all vectors of form *Nx1* attributed to the tree accordingly..

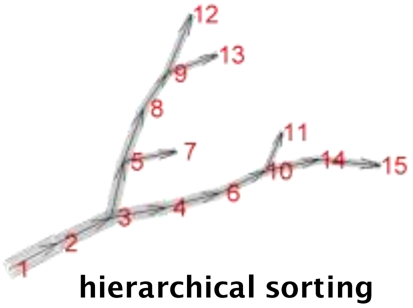

hierarchical sorting

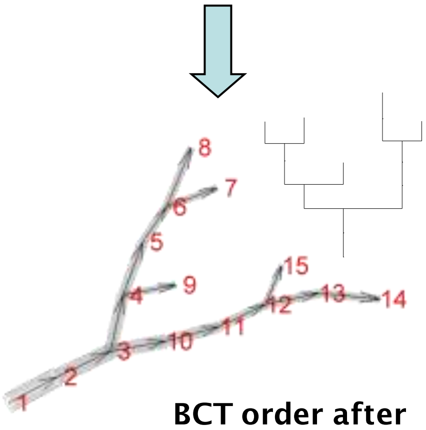

BCT order after sorting with “sort_tree”

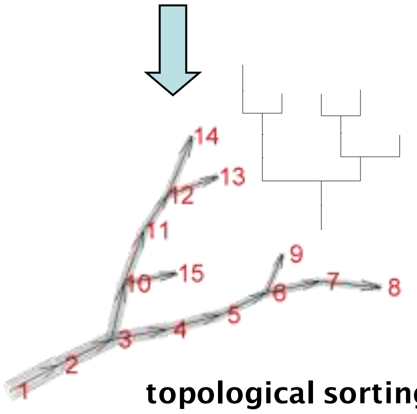

topological sorting

“sort_tree” with option “-LO”

Example:
after redirecting the tree from a different root (<u>see “redirect_tree”</u>) the nodes are scrambled. Try out:

```
>> rtree = redirect_tree (sample2_tree, 5);

>> sort_tree (rtree, ‘-s’);
```

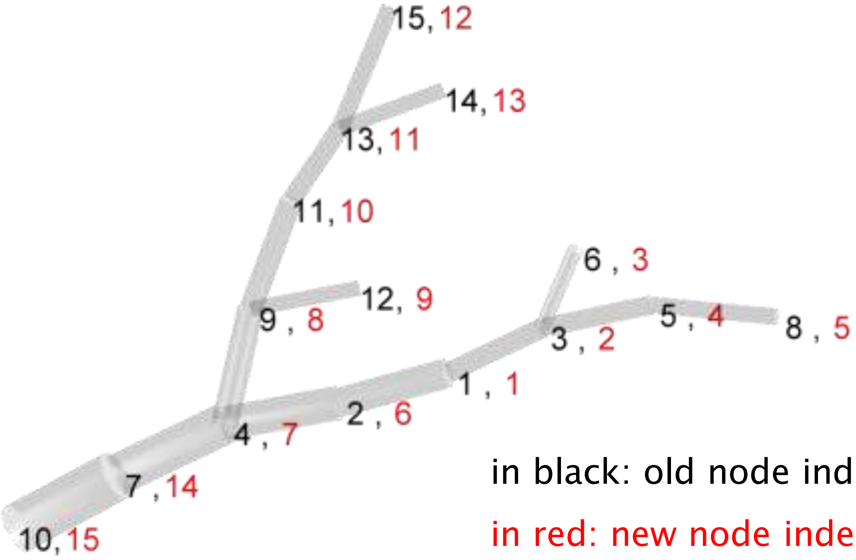

in black: old node index

in red: new node index, after sorting

sub_tree child nodes forming sub-tree

```
[sub subtree] = sub_tree (intree, inode, options)
```

Returns for tree ***intree***, an *Nx1* vector ***sub***, where the elements corresponding to a sub-tree defined by its starting node ***inode*** are “1”, and all other elements are “0”. An optional output ***subtree*** is a structure containing the tree structure corresponding to the sub-tree.

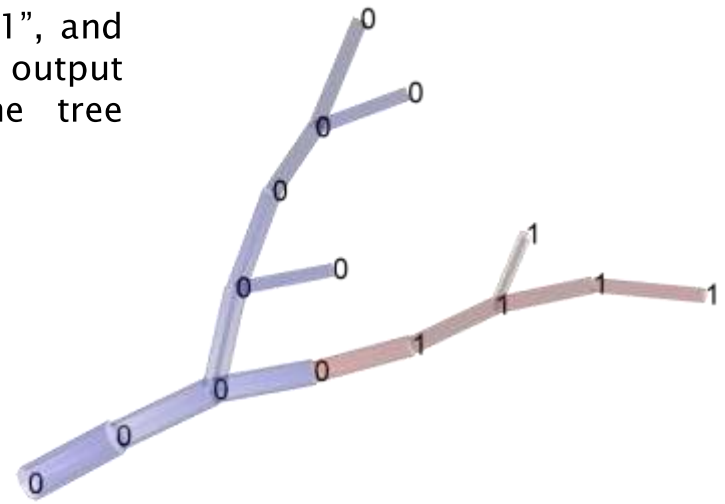

Example:

```
>> [sub subtree] = sub_tree (sample2_tree, 5)

[0, 0, 0, 0, 1, 1, 1, 1, 1, 0, 0, 0, 0, 0, 0]’
```

```
>> sum (sub)
```
⇨ number of child nodes of node #5

```
5
```

```
>> find (sub)
```
⇨ indices of nodes in the sub-tree

```
[5, 6, 7, 8, 9]
```

```
>> subtree
```
⇨ tree structure corresponding to sub-tree

```
dA: [5x5 double]
 X: [5x1 double]
 Y: [5x1 double]
 etc..
```

```
>> resttree = delete_tree (sample2_tree, find(sub))
```

```
dA: [10x10 double]
 X: [10x1 double]
 Y: [10x1 double]
 etc..
```
⇨ original tree structure without the sub-tree

T_tree termination points

T = T_tree (intree, options)

Returns for tree ***intree*** an *Nx1* vector ***T*** which is one if a given node is a termination point (exactly 0 daughter nodes) and zero else.

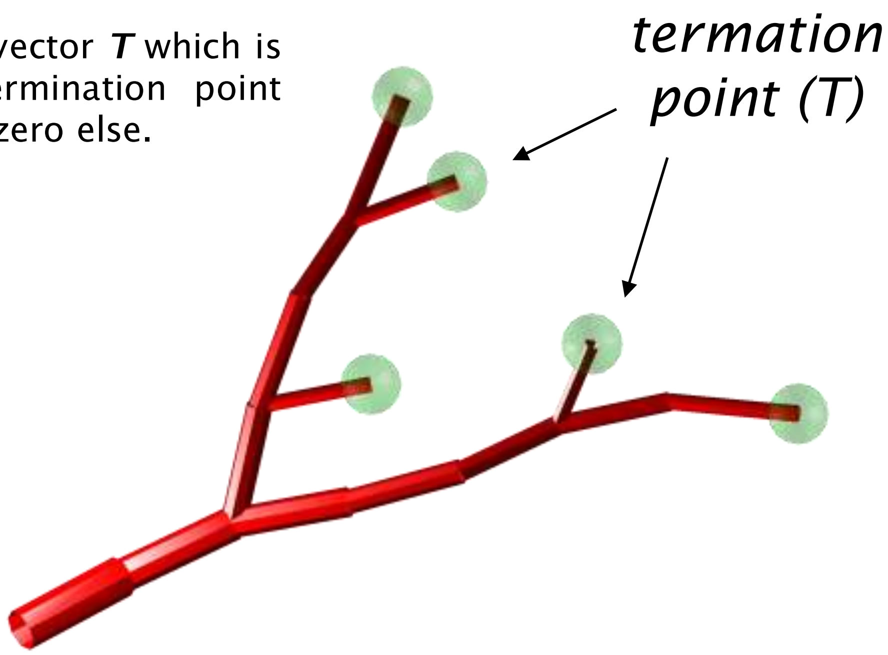

Example:

```
>> T = T_tree (sample2_tree)'

[0, 0, 0, 0, 0, 0, 0, 1, 1, 0, 0, 0, 1, 1, 1]
```

>> sum (T) ⇨ number of termination points

5

>> find (T) ⇨ indices of termination points

[8, 9, 13, 14, 15]

typeN_tree number of daughters (BCT)

typeN = typeN_tree (intree, options)

Returns for tree ***intree*** an *Nx1* vector ***typeN*** which attributes to each node either 0: termination, 1: continuation, 2: branch point. If the tree is sorted (see "sort_tree"), this is the BCT string description of the topology of the tree (see introduction section "BCT formalism"). This function simply takes the vertical sum over the adjacency matrix with 2 as an upper bound.

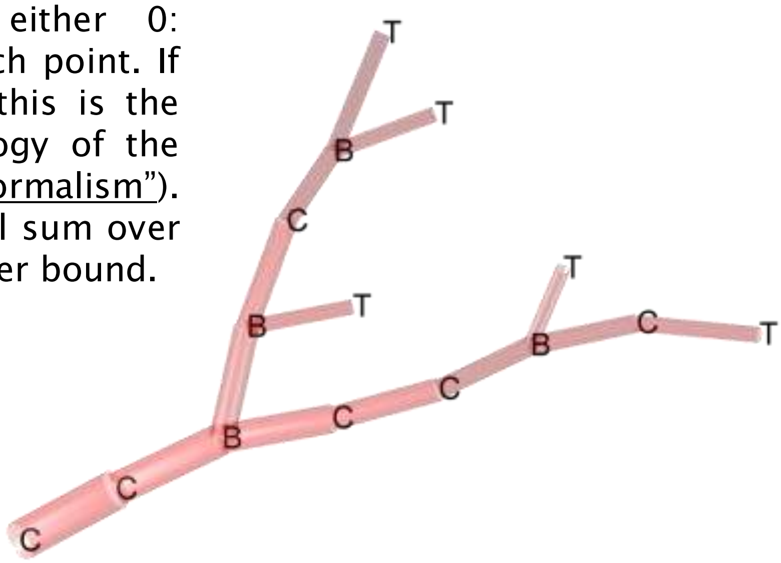

Examples:

>> typeN = typeN_tree (sample2_tree)'

[1, 1, 2, 1, 1, 2, 1, 0, 0, 2, 1, 2, 0, 0, 0]

>> typeN = typeN_tree (sample2_tree, '-bct')'

CCBCCBCTTBCBTTT ⇨ BCT string

edit

functions to edit the topology of a tree

**cat_tree**	concatenates two trees
**delete_tree**	delete a set of nodes
**elim0_tree**	eliminates zero-length segments
**elimt_tree**	replace multifurcations with bifurcations
**insert_tree**	insert a number of points into a tree
**insertp_tree**	insert nodes along a path in a tree
**recon_tree**	reconnect sub-trees to new parent nodes
**repair_tree**	restore full BCT conformity
**resample_tree**	redistributes nodes on tree
**root_tree**	add tiny segment at tree root

cat_tree concatenates two trees

tree = cat_tree (intree1, intree2, inode1, inode2, options)

Concatenates two trees ***intree2*** onto ***intree1*** at respective nodes ***inode2*** and ***inode1*** within the branching structure. Sorts the indices topologically (<u>see "sort_tree"</u> with option '-LO'). Fields are preferably taken from ***intree1***, all vectors (*X*, *Y*, *Z*, *D* etc...) must exist in both trees if they exist in one tree and are concatenated as well. Region fields *R* and *rnames* are updated.

By default, ***intree2*** is connected at its root to the closest node of ***intree1***.

Examples:
Move the sample tree (see „tran_tree“) and concatenate it with itself

```
>> ttree = tran_tree (sample2_tree, [55 25 0]);

>> cattree = cat_tree (sample2_tree, ttree);
```

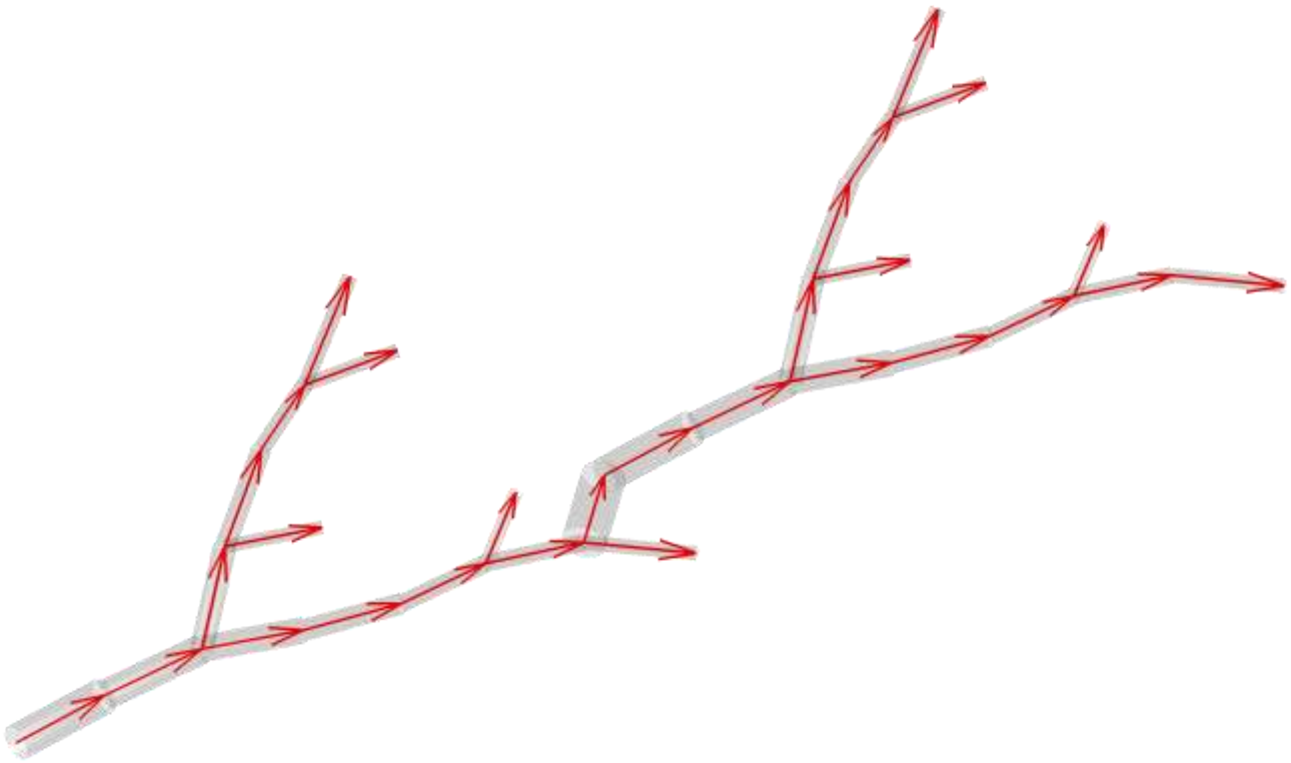

delete_tree deletes a set of nodes

tree = delete_tree (intree, inodes, options)

Deletes in tree ***intree*** a set of nodes defined by index ***inodes***. Trifurcation occurs when deleting any branch points following directly other branch points. Region numbers are changed and region name array is trimmed.

Example:

```
>> delete_tree (sample2_tree, [5 10 12 13]);
```

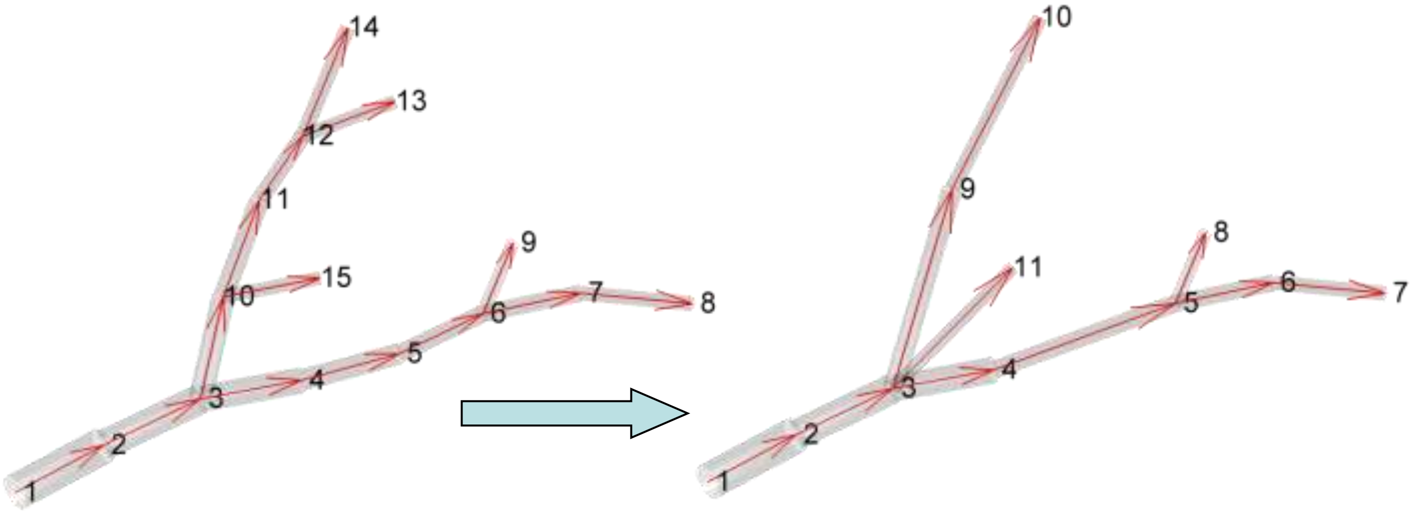

Alters the morphology of course!
Root deletion can lead to unexpected results!

elim0_tree eliminates 0-length segments

```
tree = elim0_tree (intree, options)
```

Deletes 0-length segments (except first segment of course) in tree ***intree***. Updates regions.

Example:
After setting coordinates of node 5 to those of node 4, eliminate resulting 0-length segment.

```
>> tree = sample2_tree;

>> tree.X(5) = tree.X(4); tree.Y(5) = tree.Y(4); tree.Z(5) = tree.Z(4);

>> elim0_tree (tree)
```

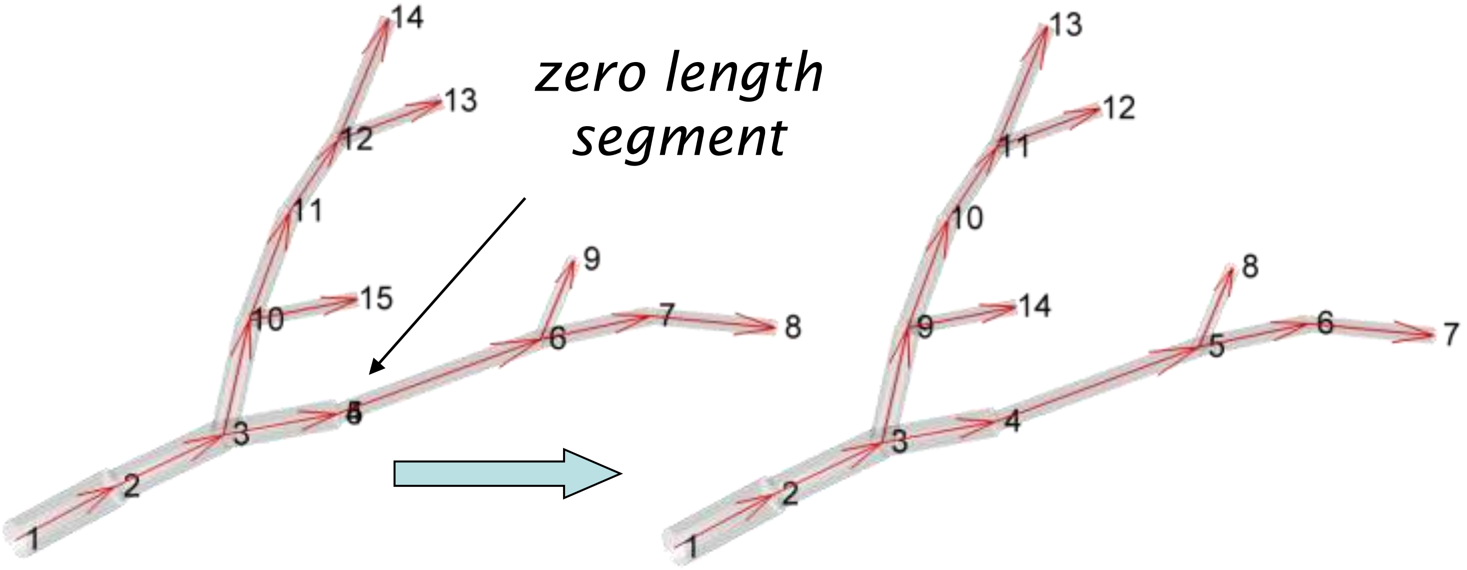

elimt_tree reduce multi- to bifurcations

```
tree = elimt_tree (intree, options)
```

Eliminates trifurcation or multifurcations present in the adjacency matrix of tree ***intree*** by adding tiny (x-deflected) compartments.

Example:
A trifurcation occurs for example when a tree is redirected to a branch point (see “redirect_tree”).

```
>> tree = redirect_tree (sample2_tree, 3);

>> elimt_tree (tree);
```

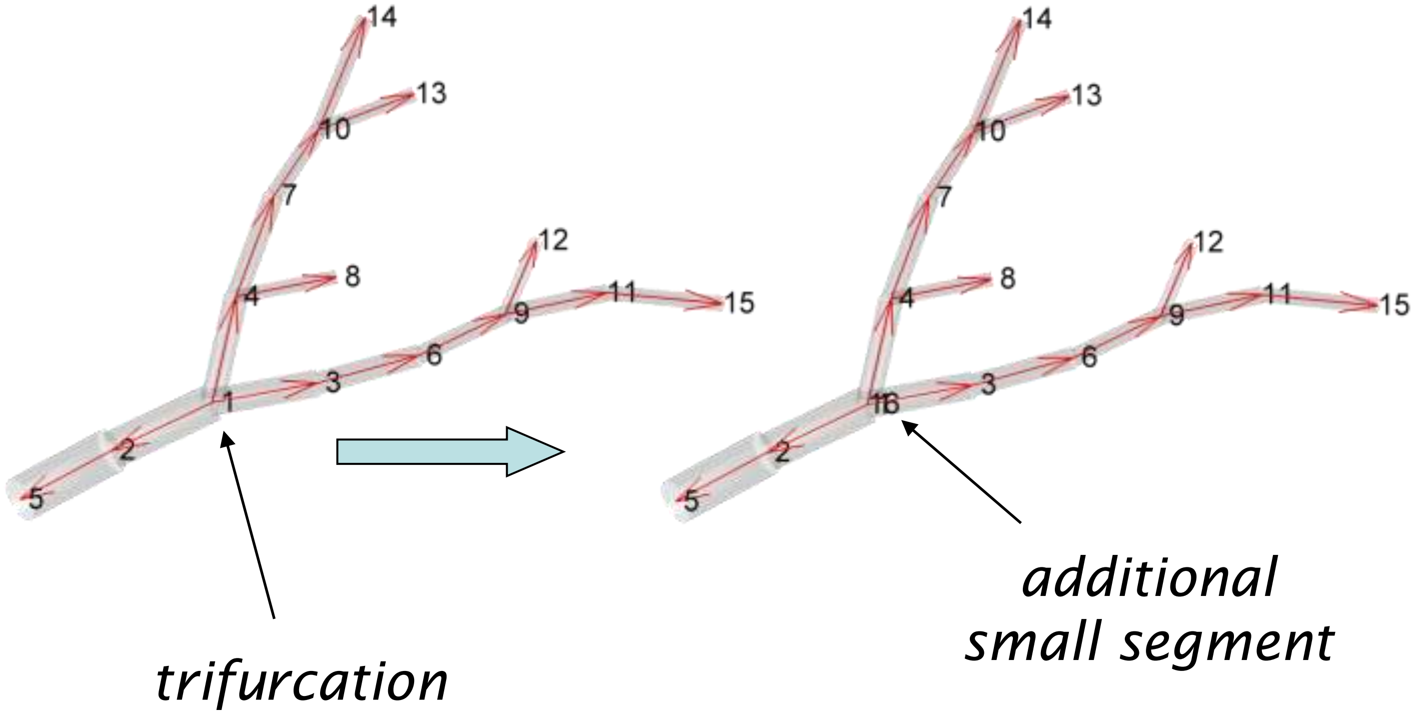

Alters the morphology slightly!

insert_tree insert new points

tree = insert_tree (intree, swc, options)

Inserts a set of points defined by a matrix ***swc*** in SWC format ([*inode R X Y Z D idpar*]) into a tree ***intree***.

Example:

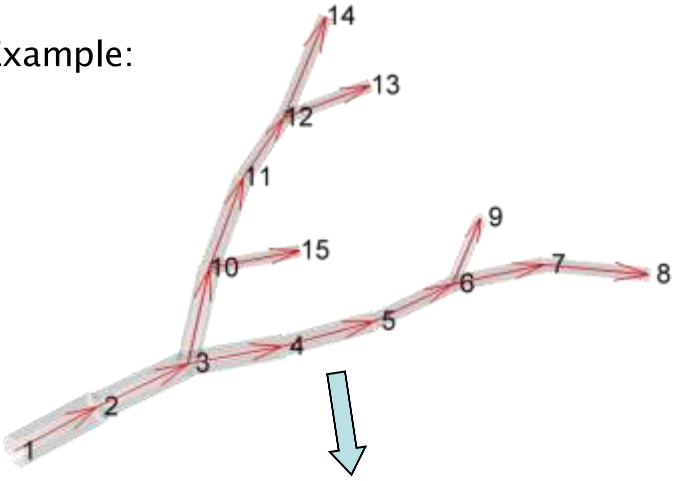

node index
region index
x-coordinate
y-coordinate
z-coordinate
diameter

```
>> tree = insert_tree (sample2_tree,[1 1 45 9 0 1 5]);
```

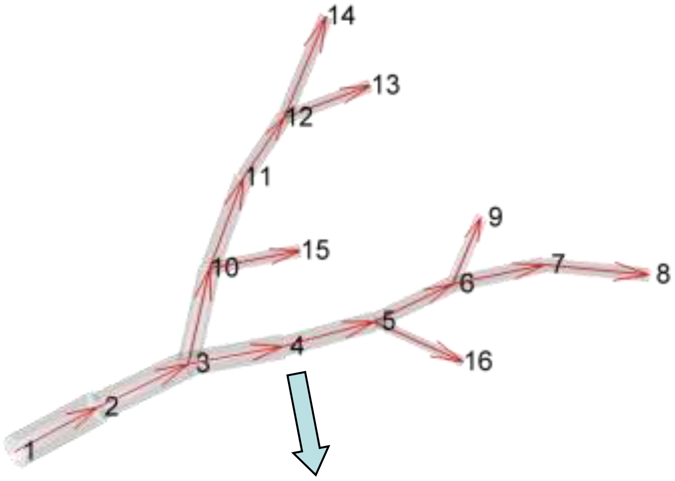

parent node index

```
>> tree = insert_tree (tree,[1 1 55 10 0 1 16]);
```

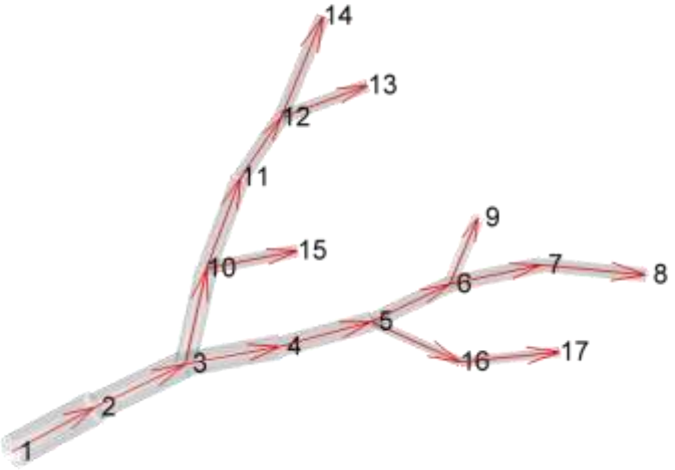

Alters the morphology of course!

these two commands are equivalent to:

```
>> tree = insert_tree (sample2_tree,[1 1 45 9 0 1 5;2 1 55 10 0 1 16]);
```

insertp_tree insert nodes along a path

`[tree indx] = insertp_tree (intree, inode, plens, options)`

Inserts nodes into tree ***intree*** at path-lengths ***plens*** on the path from the root to node ***inode***. All *Nx1* vectors are interpolated linearly but regions are taken from child nodes.

Example:
add nodes every 10 μm along path of node #14 starting at 5 μm until path length 100 μm

```
>> insertp_tree (sample2_tree, 14, 5:10:100)
```

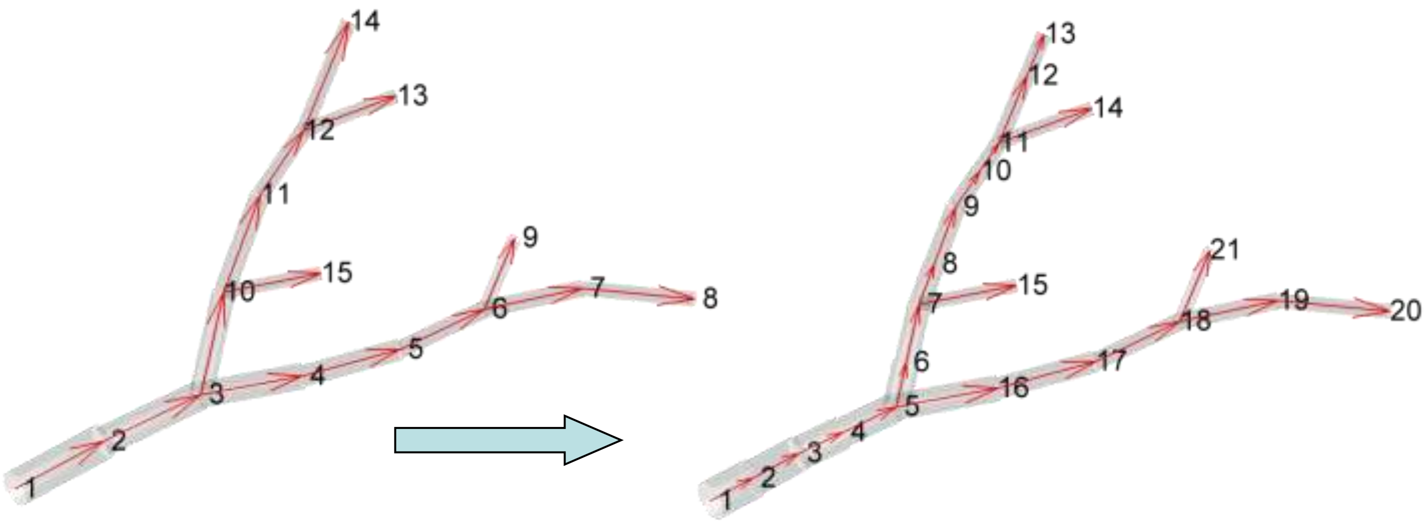

Alters the morphology slightly
and the topology strongly
of course!

recon_tree reconnects sub-trees

tree = recon_tree (intree, ichilds, ipars, options)

Reconnects within tree ***intree*** a set of sub-trees, given by node indices ***ichilds*** to new respective parent nodes defined by indices ***ipars***.

By default the sub-trees are moved directly to their new parent nodes (option “-h”). Without options this can be avoided (see below).

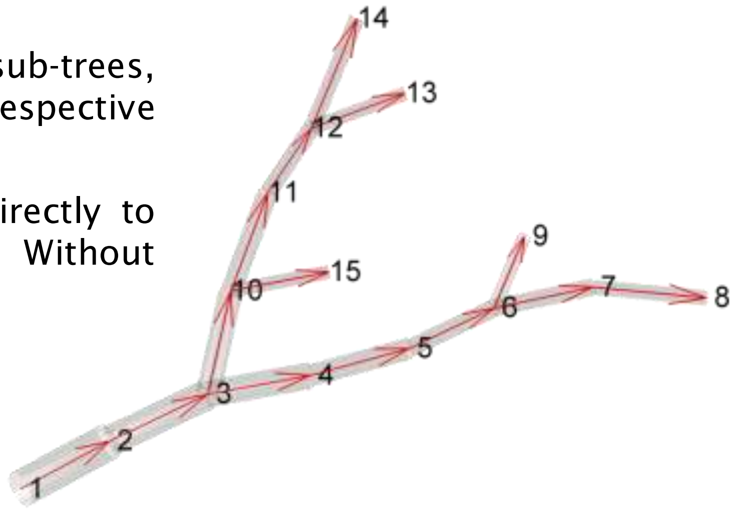

Example:

```
>> recon_tree (sample2_tree, [12 7], [15 9])
```

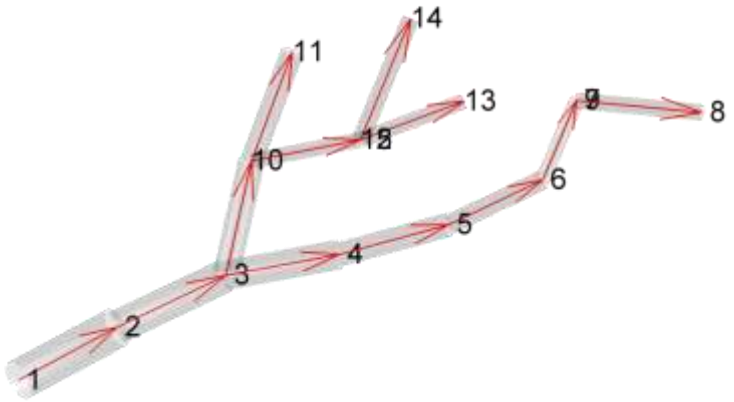


```
>> recon_tree (sample2_tree, [12 7], [15 9], 'none')
```

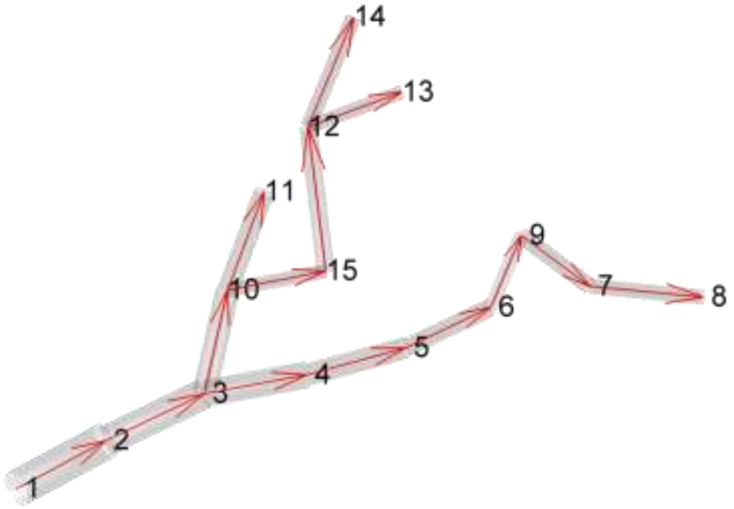

Alters the morphology of course!

repair_tree restore full BCT conformity

tree = repair_tree (intree, options)

Repairs tree ***intree***. This means removing trifurcations by adding small segments (see "elimt_tree"), removing 0-length compartments (see "elim0_tree"), and sorting the indices topologically (see "sort_tree" with "-LO" option). Applying this function is crucial for many other functions in this toolbox, which assume for example BCT-conformity. Importing tree (e.g. with "load_tree") automatically calls this function.

Example:

```
>> repair_tree (tree)
```

May alter the morphology slightly!

resample_tree redistributes nodes

`tree = resample_tree (intree, sr, options)`

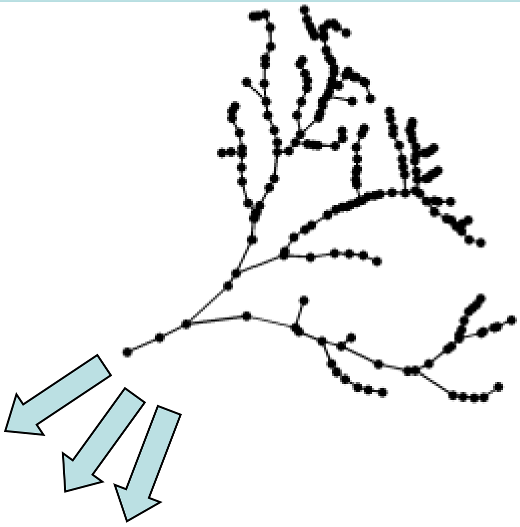

Resamples a tree ***intree*** to equidistant nodes of distance ***sr*** in µm. See introduction section “resampling a tree” for the details on the abstraction principles.

Example:

```
>> resample_tree (sample_tree, 10)
```

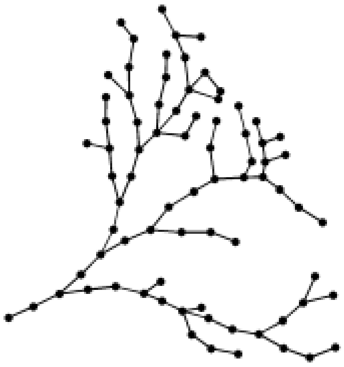

10 µm resampling

```
>> resample_tree (sample_tree, 20)
```

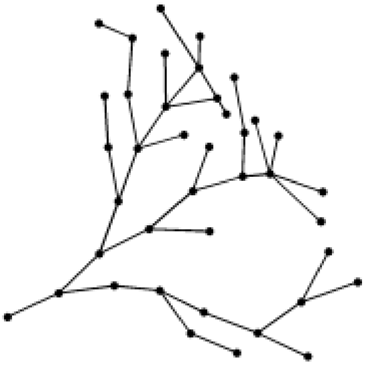

20 µm resampling

```
>> resample_tree (sample_tree, 20, '-l')
```

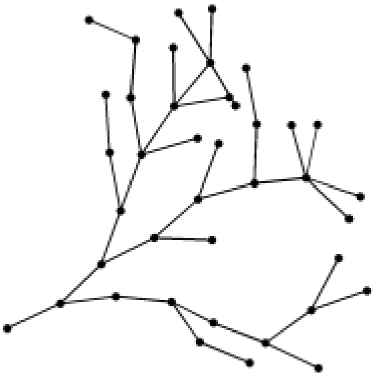

20 µm resampling with length conservation

This does alter the morphology!

root_tree add tiny segment at tree root

tree = root_tree (intree, options)

Roots a tree ***intree*** by adding a tiny segment in the root. This is rather an internal function.

Example:

```
>> root_tree (sample2_tree)
```

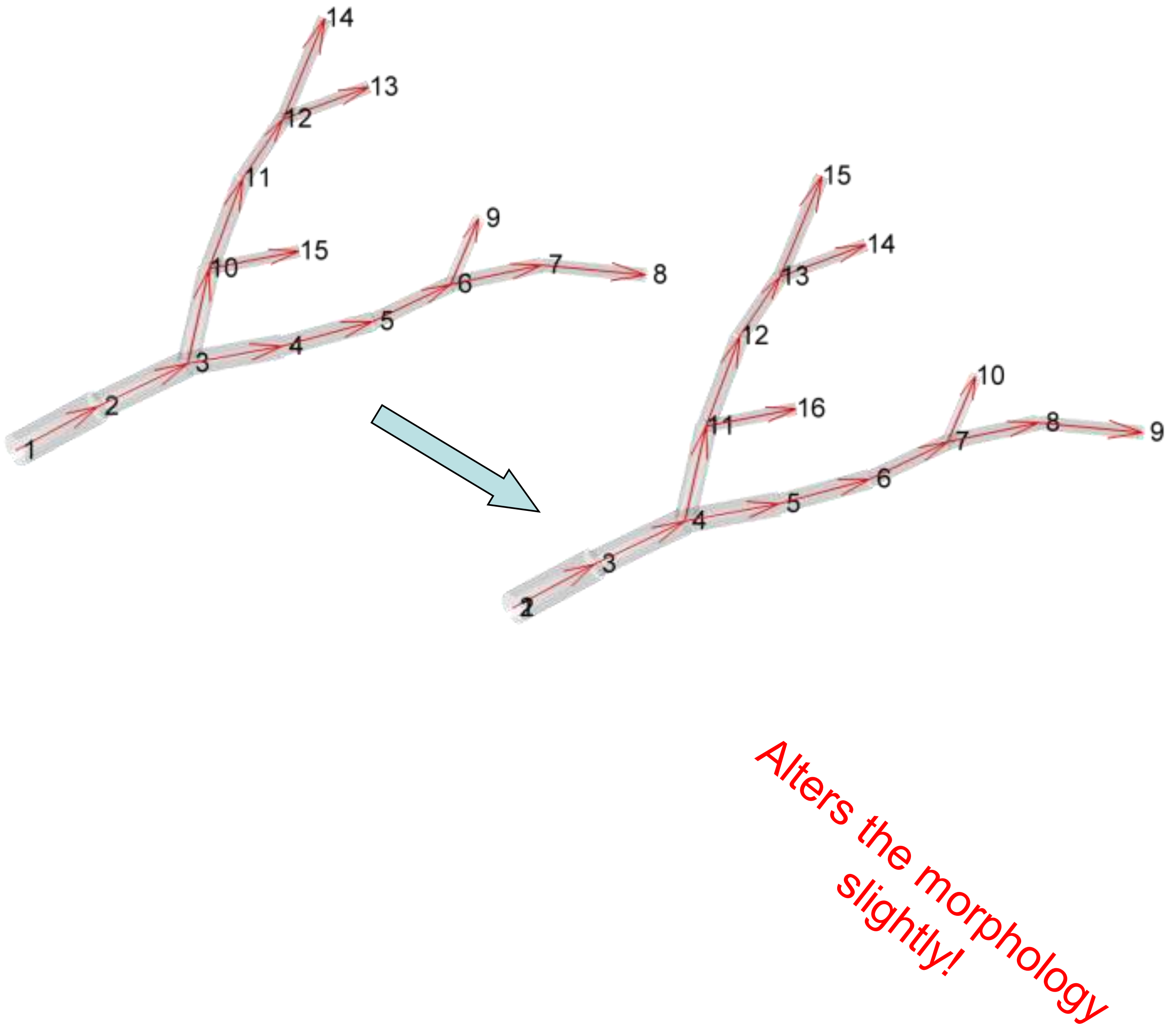

metrics

functions to obtain or alter metrics from a tree

**angleB_tree**	angle values at branch points
**cvol_tree**	continuous volume of segments
**cyl_tree**	cylinder coordinates of segments
**dstats_tree**	display tree statistics from “stats_tree”
**eucl_tree**	Euclidean distances within a tree
**flatten_tree**	flattens a tree onto XY-plane
**flip_tree**	flips a tree around one axis
**len_tree**	length values of tree segments
**morph_tree**	alter metrics preserving angles and topology
**rot_tree**	rotate a tree
**scale_tree**	scale a tree
**sholl_tree**	real sholl analysis
**stats_tree**	collects a number of tree statistics
**surf_tree**	surface values of tree segments
**tran_tree**	move a tree
**vol_tree**	volume values of tree segments
**zcorr_tree**	corrects neurolucida z-artifacts

angleB_tree branch point angles

angleB = angleB_tree (intree, options)

At each branch point of tree ***intree***, angle values corresponding to the branching angle within the branching plane are returned. *Nx1* vector ***angleB*** is *NaN* at nodes which are not branch points. Cone angle calculation is not yet implemented.

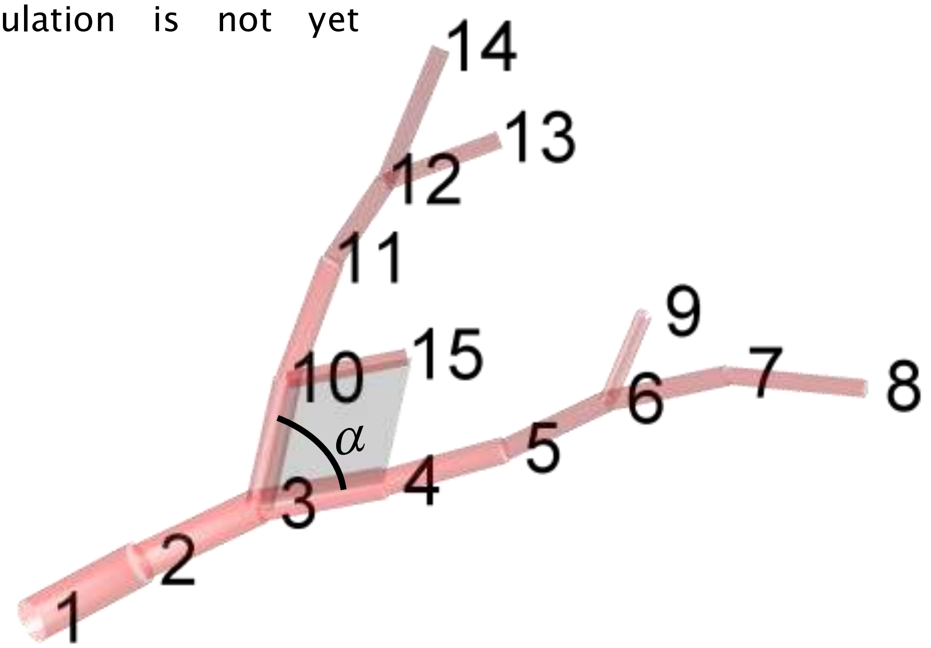

Example:

>> angleB_tree (sample2_tree)'

[NaN, NaN, 1.1, NaN, NaN, 0.9, NaN, NaN, NaN, 1, NaN, 0.8, NaN, NaN, NaN]

see demo movie with option '-m'

intree must be BCT!!
No trifurcations!!

cvol_tree segment's continuous volume

```
cvol = cvol_tree (intree, options)
```

Returns an *Nx1* vector ***cvol*** of continuous volumes for all segments [in 1/μm] in tree ***intree***. This is used by electrotonic calculations in relation to the specific axial resistance [ohm cm] (see "sse_tree“).

$4l/(\pi D^2)$ cylinder-based

$12l/(\pi(D_1^2 + D_1 D_2 + D_2^2))$ frustum-based

Example:

```
>> cvol_tree (sample2_tree, '-s')
```

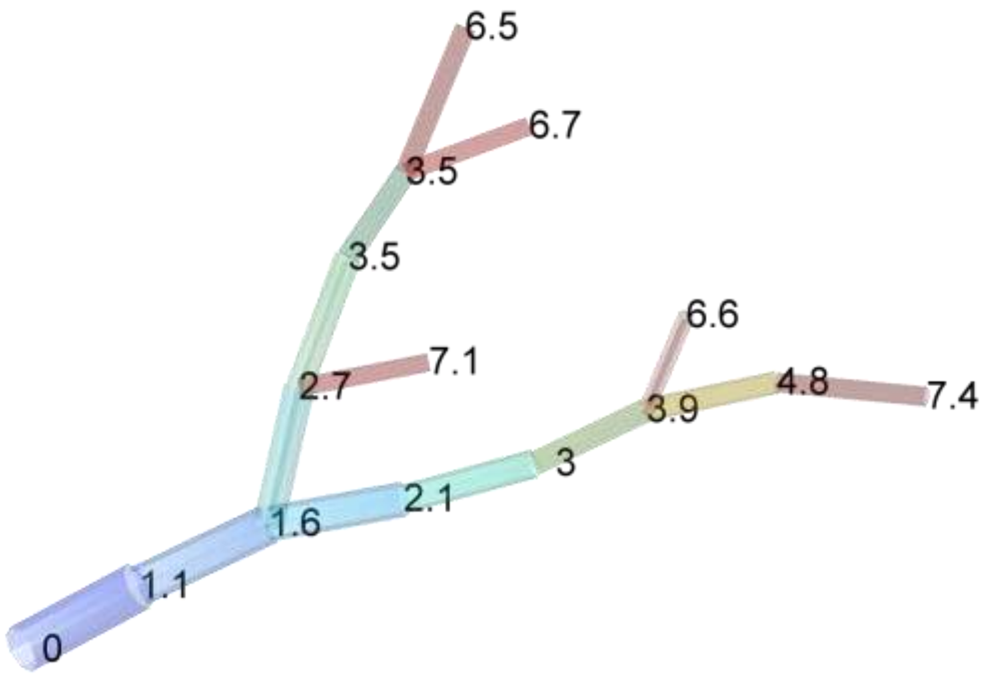

cyl_tree segment cylinder coordinates

[X1, X2, Y1, Y2 ,Z1, Z2] = cyl_tree (intree, options) *or*

M = cyl_tree (intree, options)

Uses the adjacency matrix to obtain the starting and ending points of the individual segments of tree ***intree***. Option "-dA" writes the coordinates at the position of the corresponding segment in the adjacency matrix. Outputs ***X1***, ***X2***, ***Y1***, ***Y2***, ***Z1*** and ***Z2*** are *Nx1* vectors, ***M*** is the concatenated *Nx6* matrix.

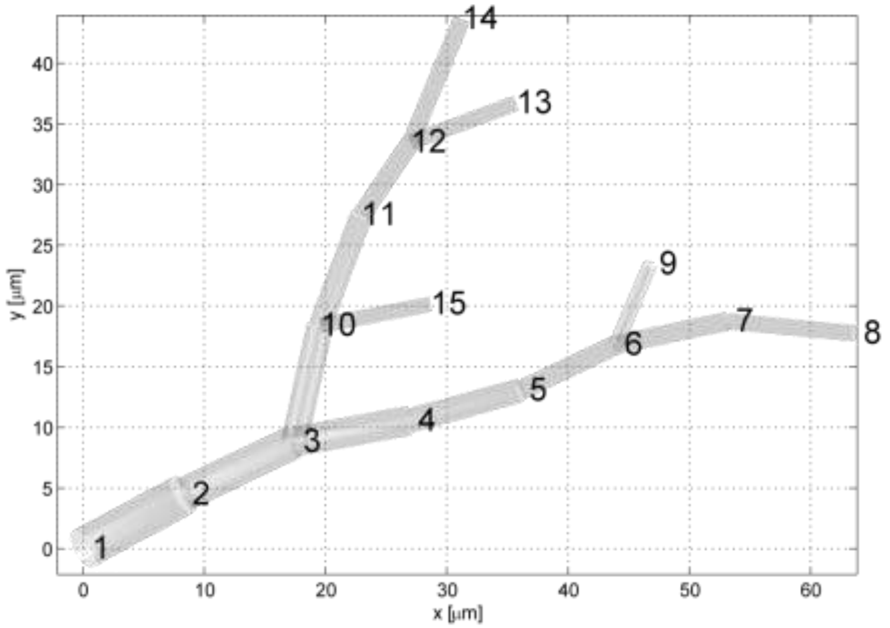

Example:
The 2D coordinates of all segments defined by the respective nodes in the tree

```
>> M = cyl_tree (sample2_tree, '-2d')
```

2D option prevents output of z-coordinates

**0**	**0**	**0**	**0**
**0**	**8**	**0**	**4**
**8**	**17**	**4**	**9**
**17**	**27**	**9**	**11**
**27**	**36**	**11**	**13**
**36**	**44**	**13**	**17**
**44**	**53**	**17**	**19**
**53**	**64**	**19**	**18**
**44**	**47**	**17**	**23**
**17**	**20**	**9**	**18**
**20**	**23**	**18**	**27**
**23**	**27**	**27**	**33**
**27**	**36**	**33**	**37**
**27**	**31**	**33**	**44**
**20**	**29**	**18**	**20**
X1	*X2*	*Y1*	*Y2*

dstats_tree display statistics

HP = dstats_tree (stats, vcolor, options)

Displays some statistics of sets of trees. ***stats*** can be a structure obtained from "stats_tree" or can be read out from an ".sts" file. ***vcolor*** is a *numx3* matrix attributing to each of all *num* groups of trees an RGB 3-tupel colour. Options are showing only global statistics '-g', only distributions '-d' and smoothen the distributions '-c'.

Example:

```
>> dLPTCs = load_tree ('dLPTCs.mtr');

>> dstats_tree (stats_tree (dLPTCs));
```

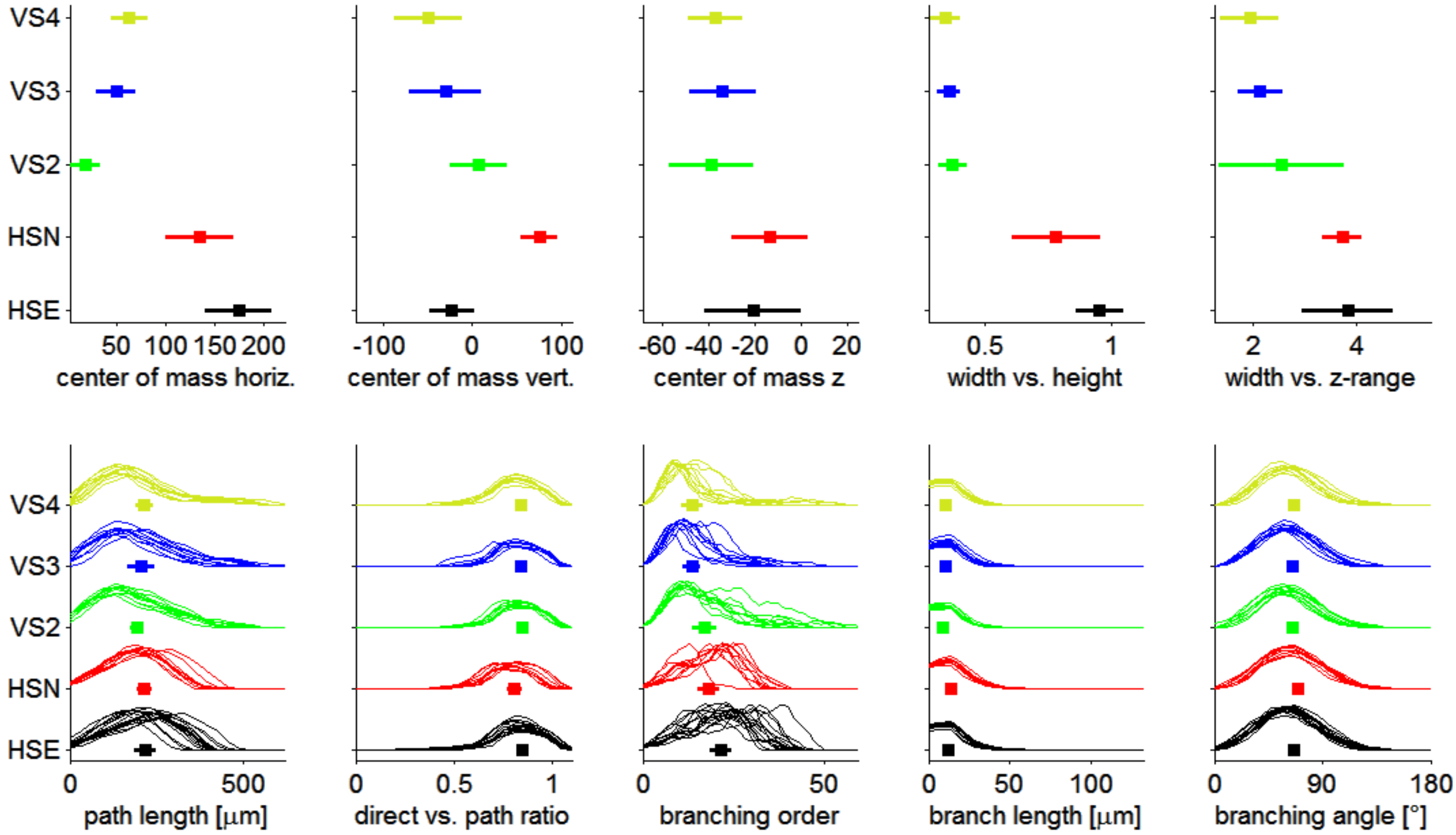

eucl_tree euclidean distances

`eucl = eucl_tree (intree, inode, options)`

Returns *Nx1* vector ***eucl*** containing the Euclidean (as the bird flies) distance [in μm] between all points on the tree ***intree*** and the root (by default) or any other node defined by its index ***inode***. ***inode*** can also be a 3-tupel of *XYZ* coordinates.

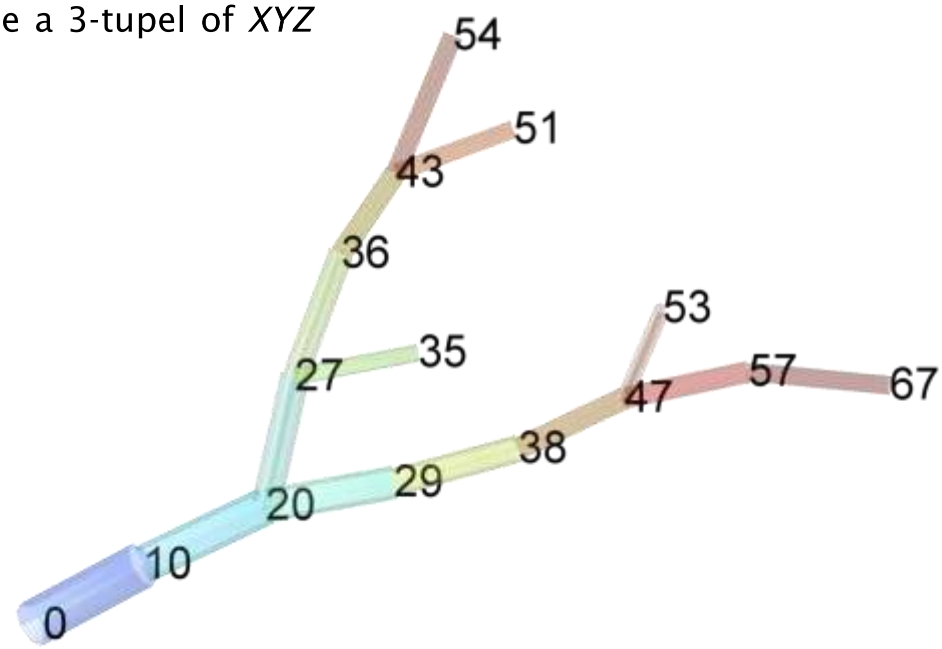

Example:

```
>> eucl_tree (sample2_tree)'

[0, 10, 20, 29, 38, 47, 57, 67, 53, 27, 36, 43, 51, 54, 35]
```

flatten_tree flattens tree onto XY plane

`tree = flatten_tree (intree, options)`

Flattens tree ***intree*** into the *XY* plane by conserving the lengths of the individual compartments. (similar to "morph_tree" but not similar enough to make one function)

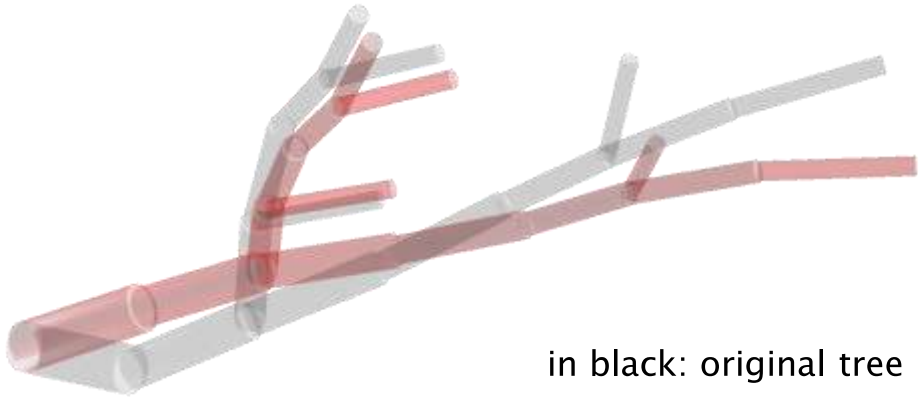

in black: original tree

in red: flattened tree

Example:

```
>> flatten_tree (sample2_tree, '-s -m')
```

See demo movie with option '-m'

flip_tree flips tree around one axis

```
tree = flip_tree (intree, DIM, options)
```

Flips coordinates of tree ***intree*** around dimension ***DIM*** (1: x-axis, 2: y-axis, 3:z-axis).

Example:

```
>> flip_tree (sample2_tree, 1)
```

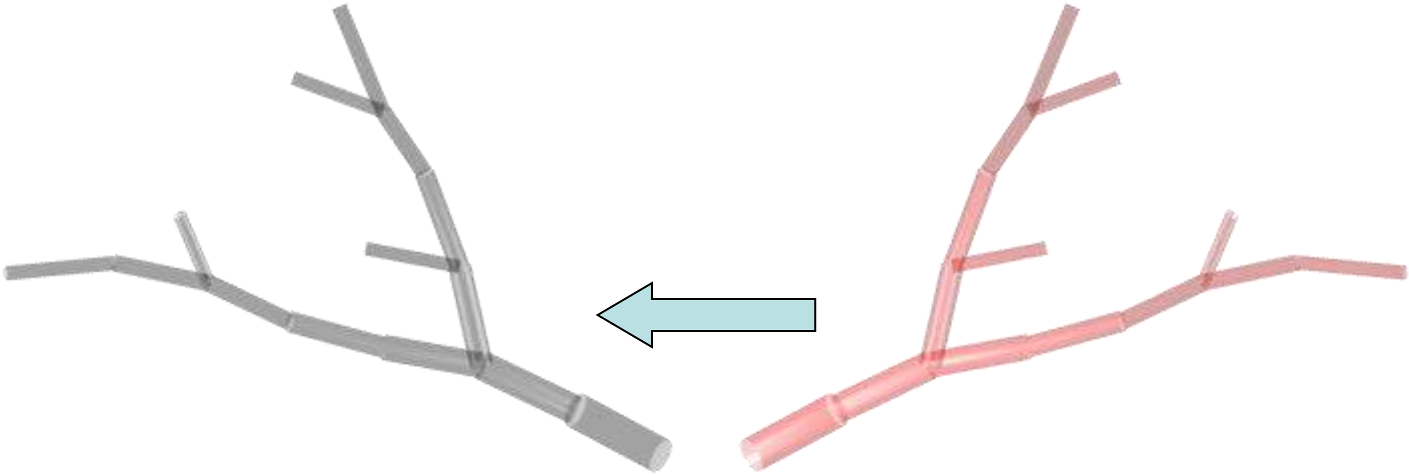

in red: original tree

in black: flipped tree

len_tree length values of tree segments

```
len = len_tree (intree, options)
```

Returns *Nx1* vector ***len***, the lengths of all segments [in μm] in tree ***intree*** using the *X*, *Y* and *Z* coordinates and the adjacency matrix.

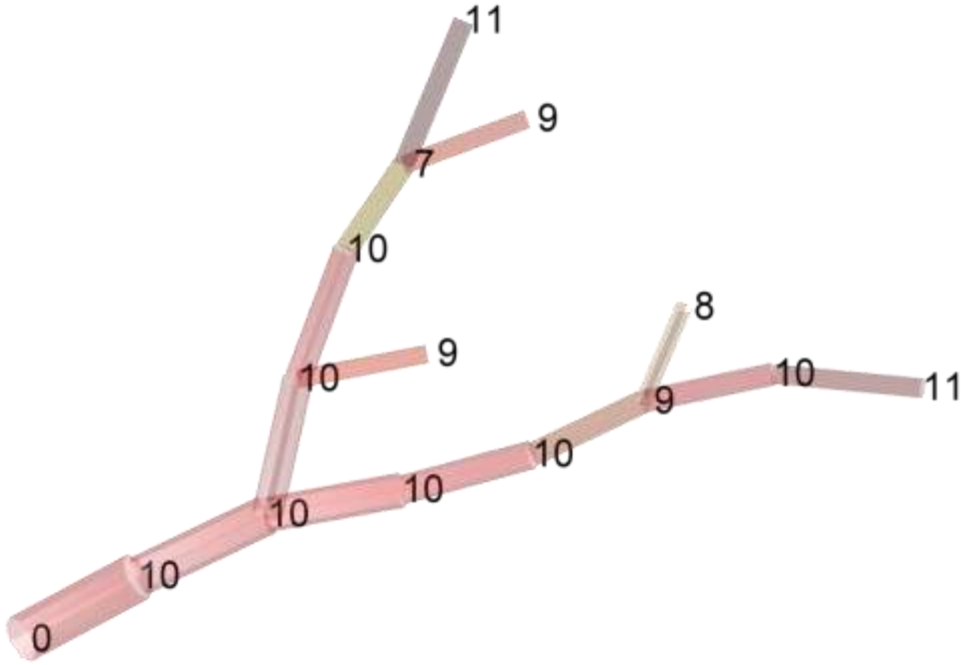

Example:

```
>> len_tree (sample2_tree)'

[0, 10, 10, 10, 10, 9, 10, 11, 8, 10, 10, 7, 9, 11, 9]
```

morph_tree

maps new length values onto segments

tree = morph_tree (intree, v, options)

Morphs the metrics of tree ***intree*** without changing angles or topology (see introduction section “morphing a tree”). Attributes length values from *Nx1* vector ***v*** to the individual segments but keeps the branching structure otherwise intact. This can result in overlap between previously non-overlapping segments or extreme sparseness depending on ***intree*** and ***v***. This function provides universal application to all possible morpho-electrotonic transforms and much more. If the original lengths of segments are backed up in a vector *len*, the original tree can simply be recovered by:

originaltree = morph_tree (morphedtree, *len*);

However of course, 0-length segments cannot be regrown.

Example:
map new (random) lengths on the topology, conserving the angles

```
>> morph_tree (sample2_tree, randn(15,1)*5+10)
```

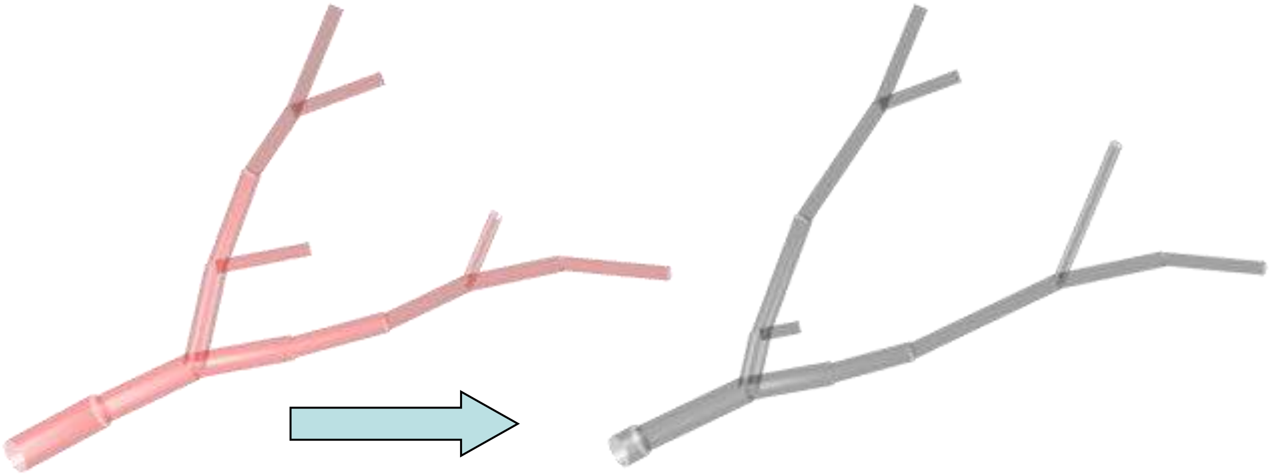

in red: original tree

in black: morphed tree

meta-function

see demo movie with option ‘-m‘

rot_tree rotate a tree

tree = rot_tree (intree, DEG, options)

Rotates tree ***intree*** by multiplying each point in space with a simple 3x3 (3D) or 2x2 (2D) rotation-matrix. Rotation along principal components is also possible (option '-pc2d' or '-pc3d'). Then first pc is in x, second pc is in y, third pc is in z. ***intree*** should be first centred with "tran_tree" except if rotation around a different point is required.

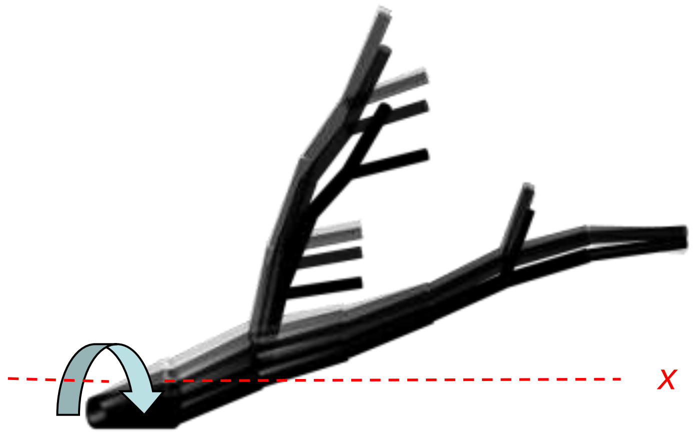

Example:

>> rot_tree (sample2_tree, [x 0 0])

Example:

>> rot_tree (sample2_tree, [0 y 0])

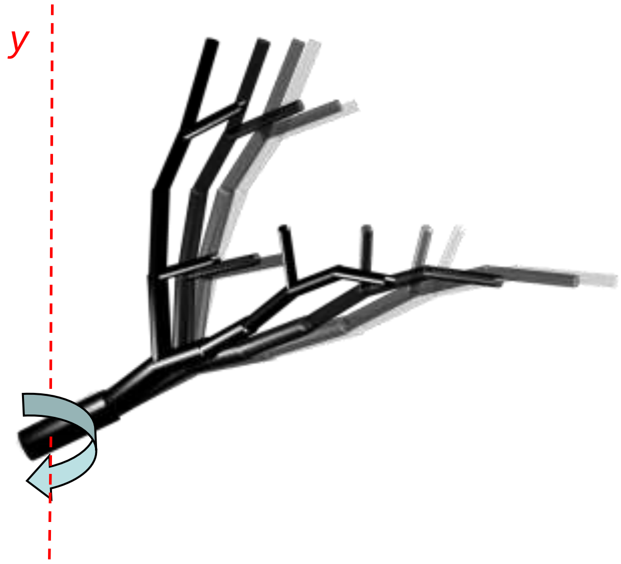

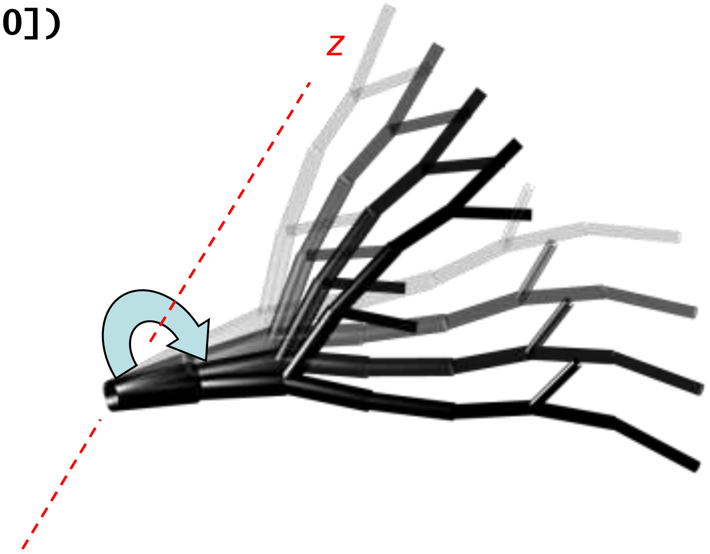

Example:

>> rot_tree (sample2_tree, [0 0 z])

scale_tree scales a tree

```
tree = scale_tree (intree, fac, options)
```

Scales the entire tree ***intree*** by factor ***fac***. If ***fac*** is a 3-tupel, the scaling factor can be different for *X*, *Y* and *Z*. With equal scaling, diameter is scaled by default. Option ‘-d’ can prevent this.

Example:

```
>> scale_tree (sample2_tree, [x 1 1])
```

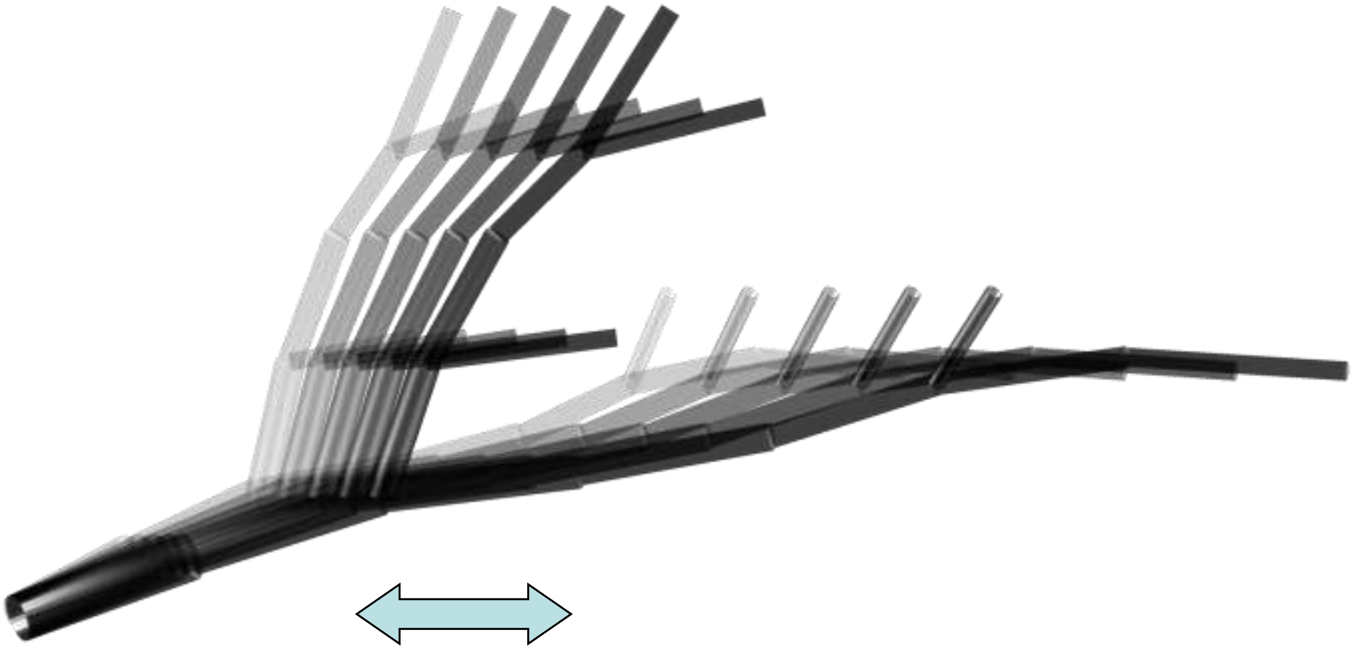

sholl_tree real Sholl analysis

[s, dd, sd, XP, YP, ZP, iD] = sholl_tree (intree, dd, options)

Calculates a Sholl analysis on tree ***intree*** counting the number of intersections of the tree with concentric spheres of increasing diameter values given by ***dd***. ***dd*** can also simply be a single value, the diameter increasing step, by default 50 μm. Outputs are ***s***, number of intersections at diameters ***dd***. A segment can intersect a circle or sphere twice, these double intersections are counted in ***sd***. ***XP***, ***YP*** and ***ZP*** are the coordinates of the intersection points. ***iD*** is the index of these points in ***dd***. Diameter 0 μm is 1 intersection by definition but typically 4 points are still output into *XP*.

Equations for intersection between line segments and spheres are from Paul Bourke 1992:

http://local.wasp.uwa.edu.au/~pbourke/geometry/sphereline/

Example:

```
>> sholl_tree (sample2_tree, 20, '-s')
```

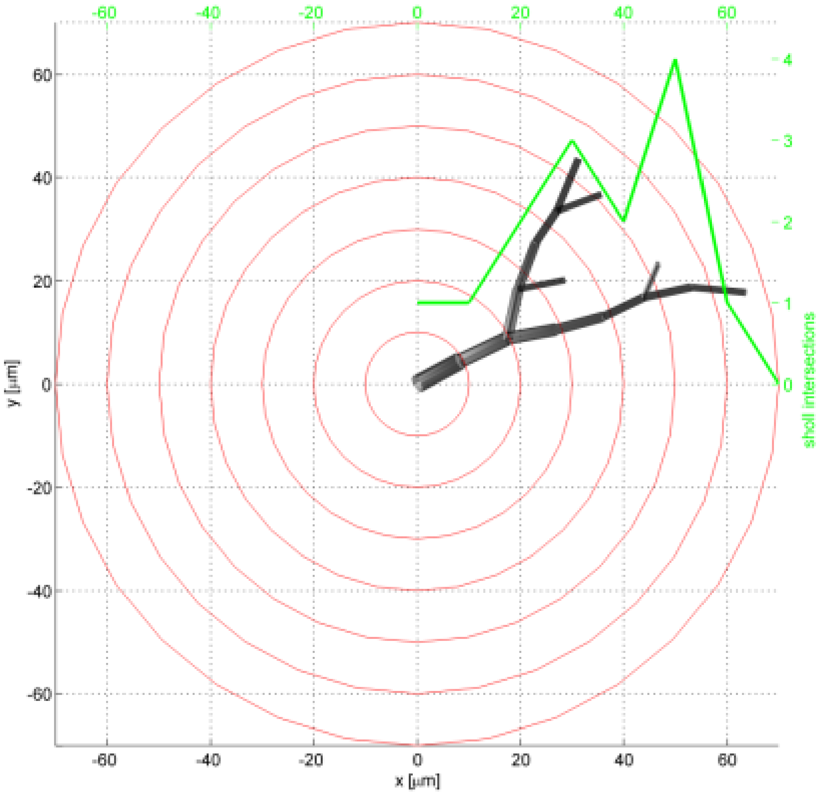

The calculation happens in 3D:

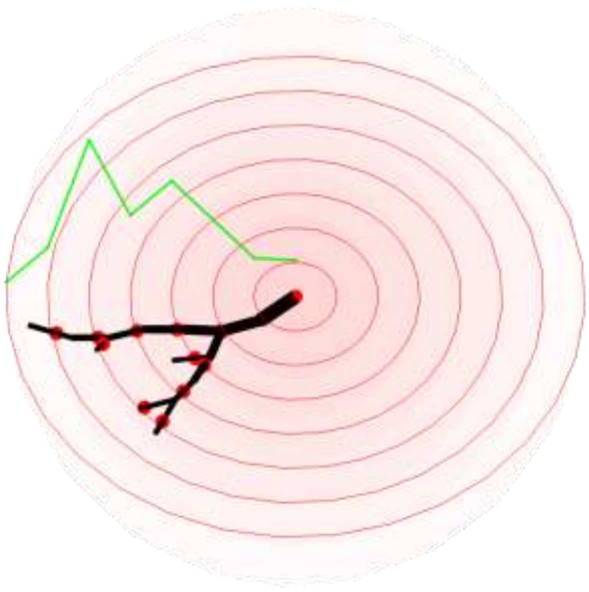

in red: concentric circles/spheres

red dots: intersections

green: Sholl intersection count

in black: tree

stats_tree collect trees statistics

[stats name path] = stats_tree (intrees, s, name, options)

Collects typical statistics on trees ***intrees***. Input trees can be organized as:

.single tree

.one group of trees: {$tree_1$, $tree_2$,... $tree_n$}

.many groups of trees: {{$tree_{i1}$,...,$tree_{in}$},{$tree_{j1}$,...,$tree_{jm}$},...}

With ***s*** a cell array of names defining the individual groups of trees can be passed on, option '-f' saves statistics to file with name ***name***. option '-x' avoids time consuming statistics (use that!!) and option '-s' shows the result (see "dstats_tree"). This function will shortly be greatly enhanced.

Example:

```
>> dLPTCs = load_tree ('dLPTCs.mtr')

{1x15 cell} {1x10 cell} {1x10 cell} {1x10 cell} {1x10 cell}

>> stats_tree (dLPTCs,{'HSE','HSN','VS2','VS3','VS4'},[],'-w -x')

    gstats: [1x5 struct]
    dstats: [1x5 struct]
         s: {'HSE'  'HSN'  'VS2'  'VS3'  'VS4'}
       dim: 3
```

>> stats.gstats (global stats)

1x5 struct array with fields:
- **len**, total cable length
- **max_plen**, maximum path length
- **bpoints**, number of branch points
- **mpeucl**, mean path/Euclidean distance
- **maxbo**, maximum branch order
- **mangleB**, mean branch angle
- **mblen**, mean branch length
- **mplen**, mean path length
- **mbo**, mean branch order
- **wh**, field height/width
- **wz**, field depth/width
- **chullx**, center of mass x
- **chully**, center of mass y
- **chullz**, center of mass z

>> stats.dstats (distributions)

1x5 struct array with fields:
- **BO**, branch order distribution
- **Plen**, path length distribution
- **peucl**, path/Euclidean distance dist.
- **angleB**, branch angle distribution
- **blen**, branch lengths distribution

surf_tree segment surface values

surf = surf_tree (intree, options)

Returns *Nx1* vector ***surf***, the surfaces [in μm²] of all segments of tree ***intree*** from the *X*, *Y*, *Z* and *D* coordinates and the adjacency matrix.

$\pi D l$ cylinder-based

$$\frac{\pi (D_1 + D_2)}{2} \sqrt{l^2 + \frac{(D_1 + D_2)^2}{4}}$$

frustum-based

Example:

```
>> surf_tree (sample2_tree)'

[0, 114, 91, 78, 64, 47, 48, 47, 33, 71, 61, 37, 39, 51, 38]
```

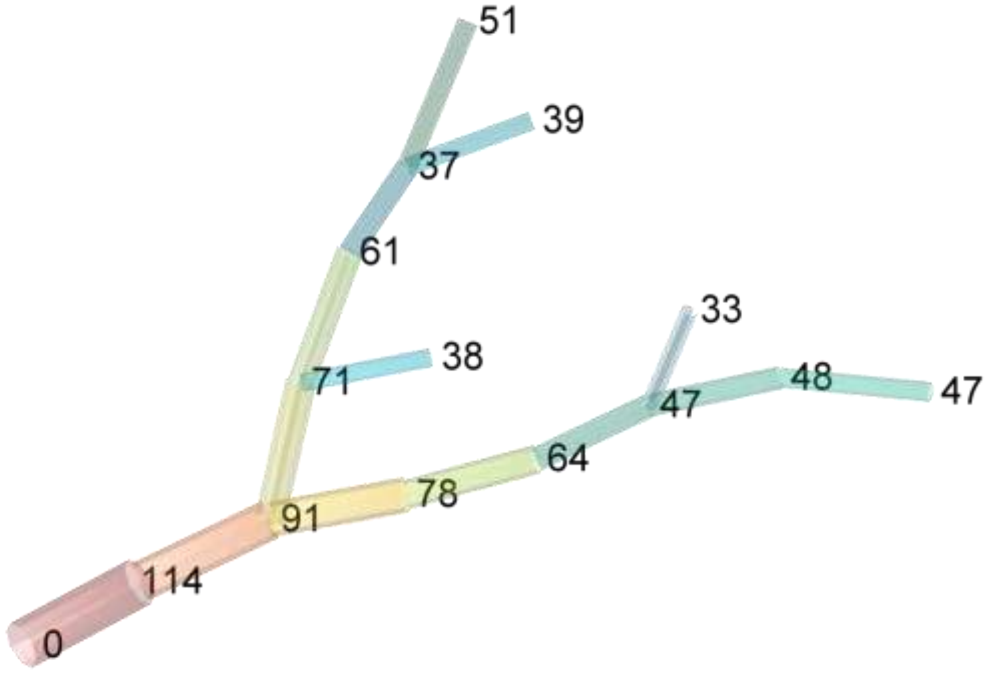

tran_tree move a tree

tree = tran_tree (intree, DD, options)

Moves tree ***intree*** according to translation coordinates given in XYZ 3-tupel ***DD***. By default centers tree by setting root to XYZ coordinates (0,0,0).

Example:

```
>> tran_tree (sample2_tree, [x 0 0])
```

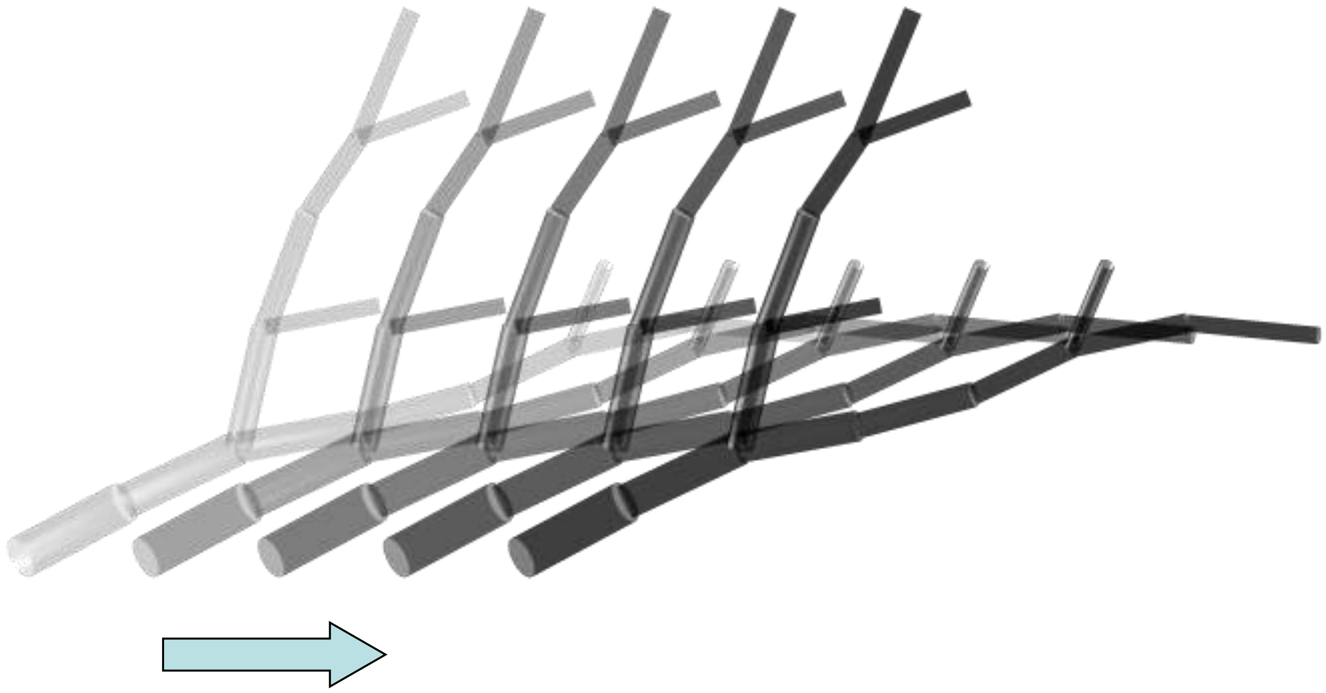

vol_tree segment volume values

vol = vol_tree (intree, options)

Returns *Nx1* vector ***vol***, the volumes [in μm³] of all segments of tree ***intree*** from the *X*, *Y*, *Z* and *D* coordinates and the adjacency matrix.

$$\frac{\pi D^2 l}{4}$$ cylinder-based

$$\frac{\pi\left(D_1^2 + D_1 D_2 + D_2^2\right)}{12}$$ frustum-based

Example:

```
>> vol_tree (sample2_tree)'
```

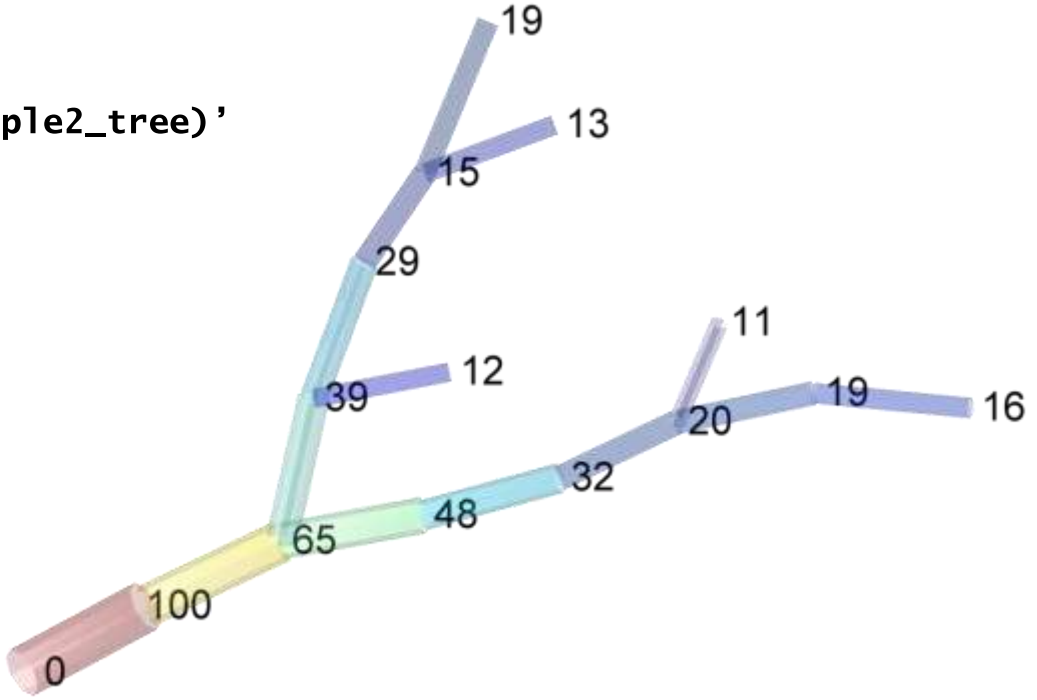

zcorr_tree corrects jumps in z

`[tree idZ] = zcorr_tree (intree, tZ, options)`

While reconstructing neuronal trees sudden shifts in the z-axis may occur. This function automatically corrects these effects in a given input tree ***intree***. Any jump in the z-axis > ***tZ***, a threshold difference in z, is subtracted from the entire sub-tree. Before applying this function, check that this is really the adequate way to process these jumps in z.

Example:
After introducing a fake jump in the z values of the sub-tree of node #5, „zcorr_tree" is applied

```
>> tree = sample2_tree; isub = find (sub_tree (tree, 5));

>> tree.Z(isub) = tree.Z(isub) - 20;

>> zcorr_tree (sample2_tree, 15)
```

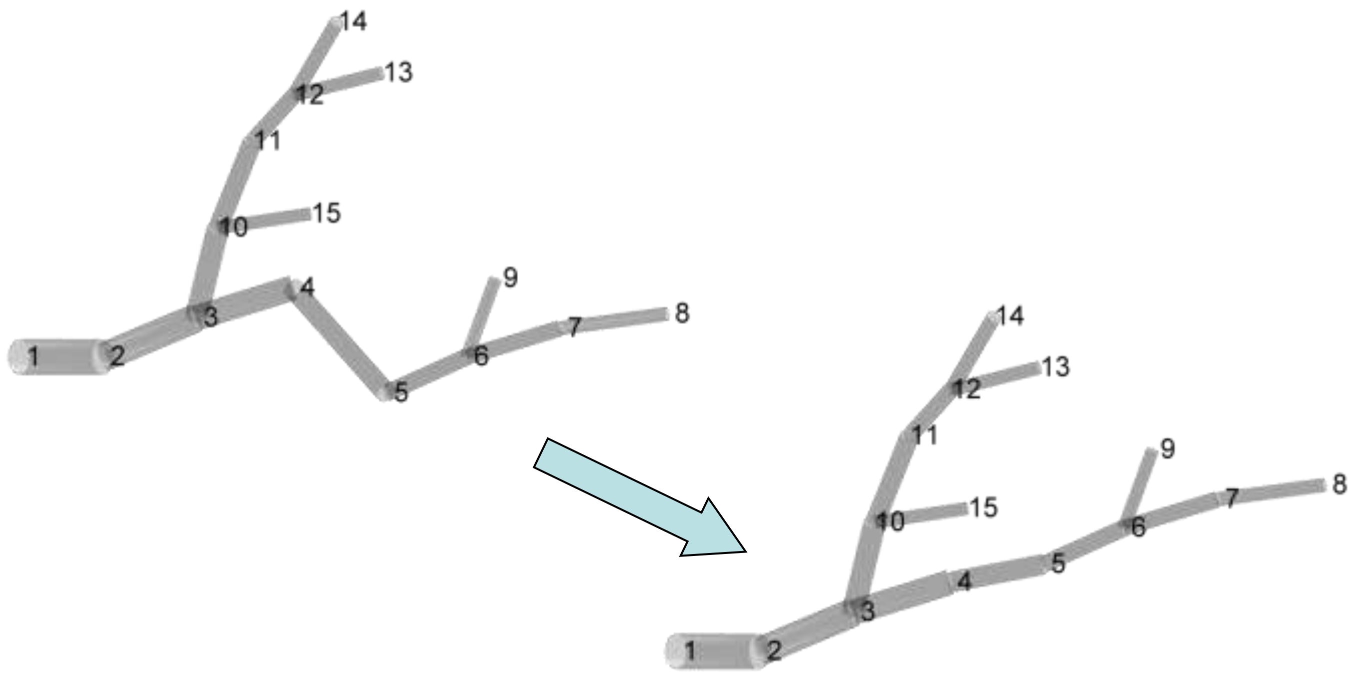

graphical

function for visual output and various hulls

**chull_tree**	convex hull around whole or part of tree
**dA_tree**	plots the adjacency matrix
**dendrogram_tree**	plots a dendrogram
**gdens_tree**	density matrix of nodes in tree
**hull_tree**	iso-distance surface or line around tree
**lego_tree**	density plot as lego pieces
**plot_tree**	plot a tree
**plotsect_tree**	plot a selected path
**pointer_tree**	spheres or electrodes at selected nodes
**spread_tree**	display trees separately
**vhull_tree**	voronoi based subdivision
**vtext_tree**	write text at node locations
**xdend_tree**	x-coordinates of dendrogram
**xplore_tree**	exploration plots

chull_tree convex hulls

```
[HP hull] = chull_tree (intree, ipart, color, DD, alpha, options)
```

Plots a convex hull around nodes with index ***ipart*** of tree ***intree*** (***intree*** can also simply be an *Nx3* matrix of XYZ points). Hull patch is offset by XYZ 3-tupel ***DD*** and coloured with RGB 3-tupel ***color***. ***alpha*** sets the transparency of the patch (by default .2). Option '-2d' restricts the hull patch to two dimensions. ***HP*** is the handle to the graphical object. Set ***options*** to 'none' to avoid graphical output. Output ***hull*** is a structure containing in ***hull.XY(Z)*** the coordinates and in ***hull.ch*** the indices to the convex hull (see Matlab function "convhull").

If the tree is 100% flat 3D convex hull doesn't work. If selected nodes are two draws a straight line. If selected nodes are one plots a point.

Example 1:
convex hull around sub-tree of node #11

```
>> chull_tree (sample_tree, find (sub_tree (sample_tree,11)),...
          [0 1 0])
```

color : green

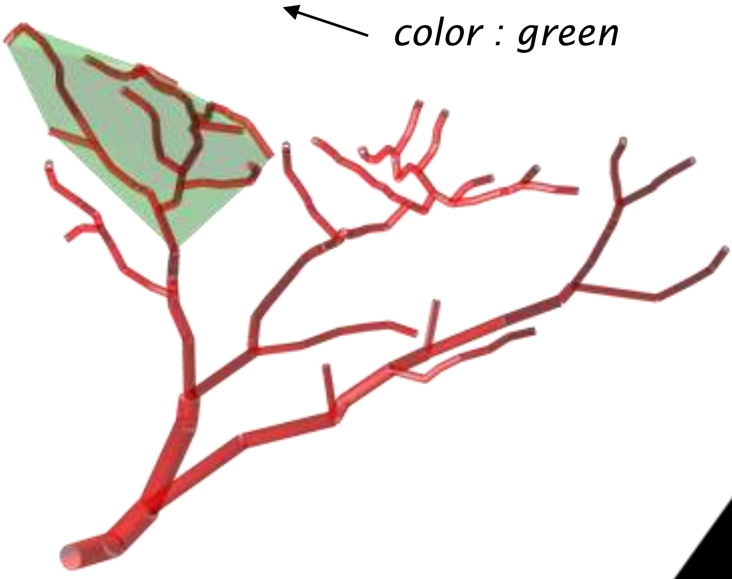

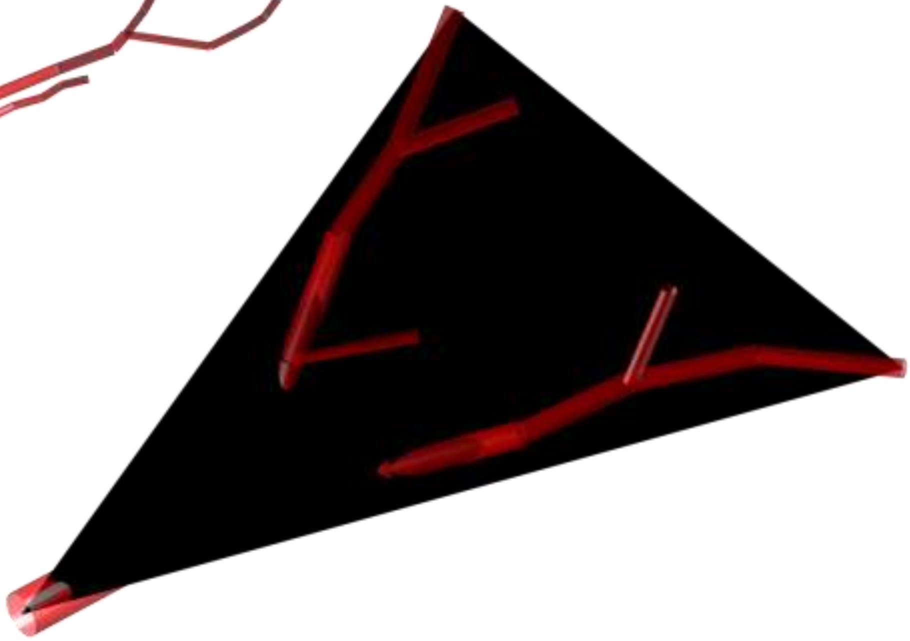

Example 2:
2D convex hull around entire tree

opaque

```
>> chull_tree (sample2_tree, [], [], [], 1, '-2d')
```

dA_tree plots adjacency matrix

```
HP = dA_tree (intree, color, DD, xyscale, options)
```

Displays the adjacency matrix of tree ***intree*** in the colour defined by RGB 3-tupel ***color***, displaced by XY/Z 2/3-tupel ***DD*** and scaled by scaling factor ***xyscale***. Fills in an *NxN* square with 1s (where there is a connection between two nodes, against 0s elsewhere) if *N*<50 and with black dots if tree is bigger. ***HP*** is the handle to the graphical object.

Example 1:
adjacency plot of a small tree

```
>> dA_tree (sample2_tree)
```

	1	2	3	4	5	6	7	8	9	10	11	12	13	14	15
1	0	0	0	0	0	0	0	0	0	0	0	0	0	0	0
2	1	0	0	0	0	0	0	0	0	0	0	0	0	0	0
3	0	1	0	0	0	0	0	0	0	0	0	0	0	0	0
4	0	0	1	0	0	0	0	0	0	0	0	0	0	0	0
5	0	0	0	1	0	0	0	0	0	0	0	0	0	0	0
6	0	0	0	0	1	0	0	0	0	0	0	0	0	0	0
7	0	0	0	0	0	1	0	0	0	0	0	0	0	0	0
8	0	0	0	0	0	0	1	0	0	0	0	0	0	0	0
9	0	0	0	0	0	1	0	0	0	0	0	0	0	0	0
10	0	0	1	0	0	0	0	0	0	0	0	0	0	0	0
11	0	0	0	0	0	0	0	0	0	1	0	0	0	0	0
12	0	0	0	0	0	0	0	0	0	0	1	0	0	0	0
13	0	0	0	0	0	0	0	0	0	0	0	1	0	0	0
14	0	0	0	0	0	0	0	0	0	0	0	1	0	0	0
15	0	0	0	0	0	0	0	0	0	1	0	0	0	0	0

Example 2:
adjacency plot of a larger tree

```
>> dA_tree (sample_tree)
```

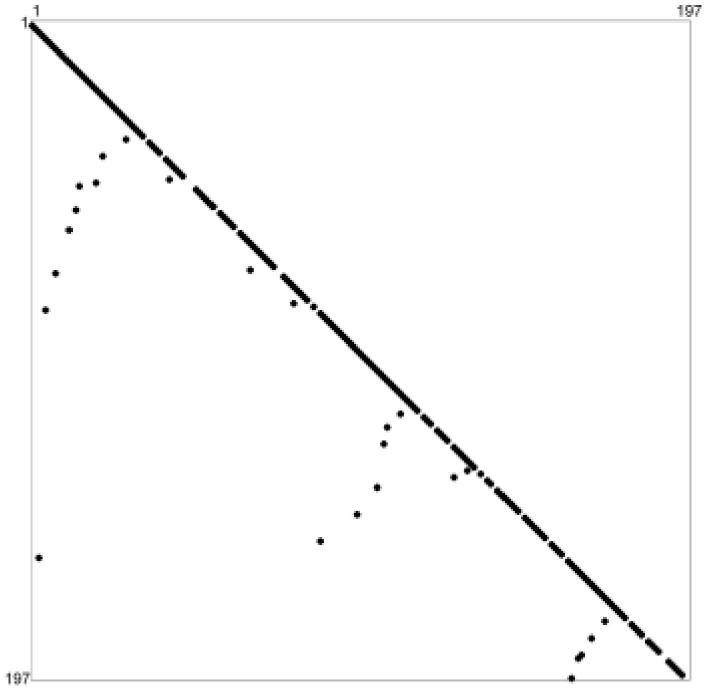

dendrogram_tree plots a dendrogram

HP = dendrogram_tree (intree, diam, yvec, color, DD, wscale, options)

tree must conform to BCT
consider applying “repair_tree” first

Plots a dendrogram of the tree ***intree*** (must conform to BCT). ***HP*** is the handle to the graphical object. *Nx1* vector ***yvec*** simply assigns a y-value to each node (metric path length by default, see “Pvec_tree”), while *Nx1* vector ***diam*** attributes a diameter (single value = constant diameter). The dendrogram is offset by XYZ 3-tupel ***DD*** and coloured with RGB 3-tupel ***color***. Single value ***wscale*** determines the spacing between two terminals.

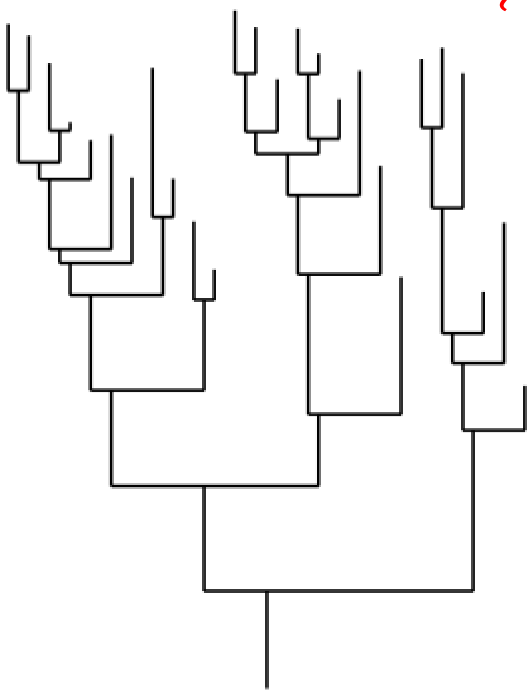

Example 1:
simple line dendrogram

```
>> dendrogram_tree (sample_tree)
```

Example 2:
“topological” dendrogram, plotting topological path length on the y-axis

```
>> PL = PL_tree (sample2_tree);
>> dendrogram_tree (sample2_tree, PL/20, 10*PL, PL, [], [], '-p -v')
```

diameter
y-axis
color
all depend on the topological path length

plot as patches but without horizontal lines

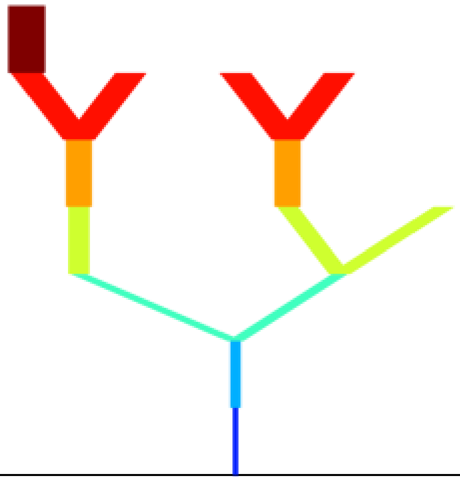

gdens_tree density matrix of tree

```
[M dX dY dZ HP] = gdens_tree (intree, sr, ipart, options)
```

Calculates a density matrix of the nodes in the tree ***intree***. Uses Matlab function “isosurface” to display the resulting gradient and increases opacity with density. ***sr*** determines the bin size for the matrix. ***ipart***, a set of indices into ***intree***, allows to use only a subset of nodes, for example only the topological points. Outputs are the density matrix ***M*** and ***dX***, ***dY*** and ***dZ*** are the *X*-, *Y*- and *Z*-dimension labels of ***M***. ***HP*** is the handle to the graphical object. Set ***options*** to ‘none’ to avoid graphical output.

Example:
Density of topological points (only termination and branch points)

```
>> iBT = B_tree(sample_tree)|T_tree(sample_tree)

>> gdens_tree (sample_tree, 20, iBT)
```

20 μm bins

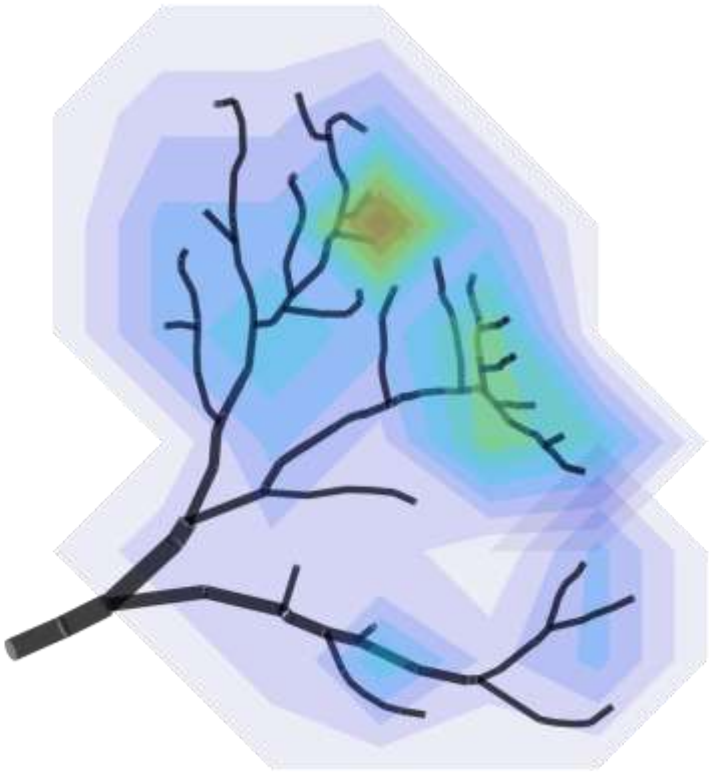

hull_tree isodistance surfaces/lines

[c M HP] = hull_tree (intree, thr, bx, by, bz, options)

Calculates a space-filling 3D isosurface around the tree ***intree*** with a threshold distance of ***thr*** [in μm]. In order to do this it creates a grid defined by the vectors ***bx***, ***by*** and ***bz*** and calculates the closest point of the tree to any of the points on the grid. Higher resolution requires more computer power but results in higher accuracy of contour. Note that for smaller threshold distances ***thr***, a better spatial resolution is required! Outputs are ***c***, a structure containing the polygon point coordinates, and the distance matrix ***M***. In the 2D case (with option '-2d'), ***c*** is a contour (see Matlab function "contourc"). ***HP*** is the handle to the graphical output object. Reduce the resulting patch resolution if necessary with:

reducepatch (***HP***, *ratio*)

Example:

```
>> hull_tree (sample2_tree, 5)
```

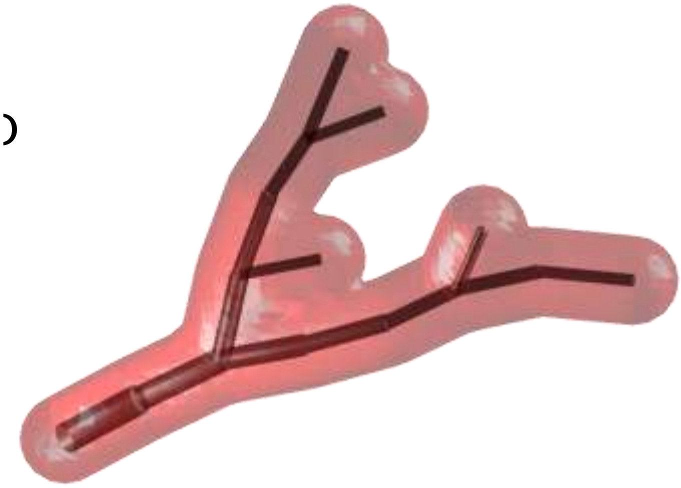

5 μm isodistance

or in 2D:

```
>> hull_tree (sample2_tree, 5, [], [], [], '-s -2d')
```

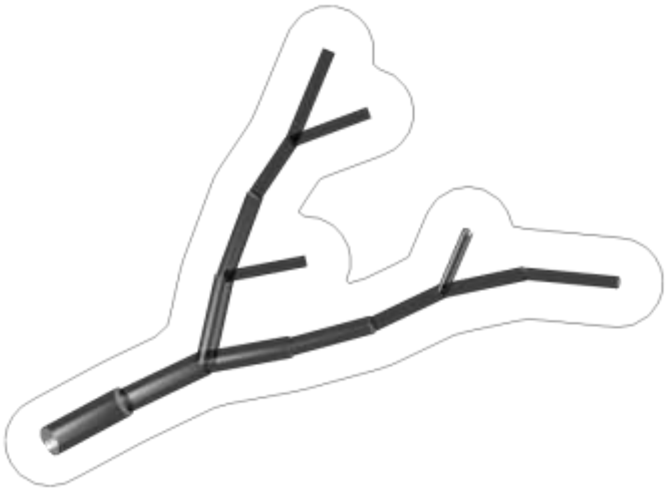

lego_tree lego density plot

`[HP, M] = lego_tree (intree, sr, thr, options)`

Uses "gdens_tree" to plot the bins of a density matrix ***M*** of points in a tree ***intree***. ***sr*** sets the resolution (size of the bins) and ***thr*** a threshold for the transparency. Opacity and colours increase with density. The individual bins are shaped like lego pieces.

Example:

```
>> tree = resample_tree (sample_tree, 1);

>> lego_tree (tree, 5)
```

without the resampling, most bins along the tree will be empty since no nodes exist there

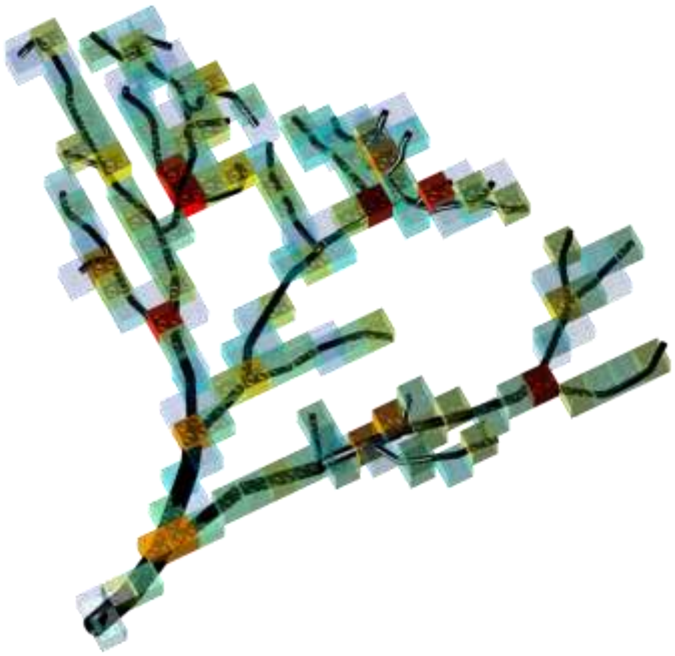

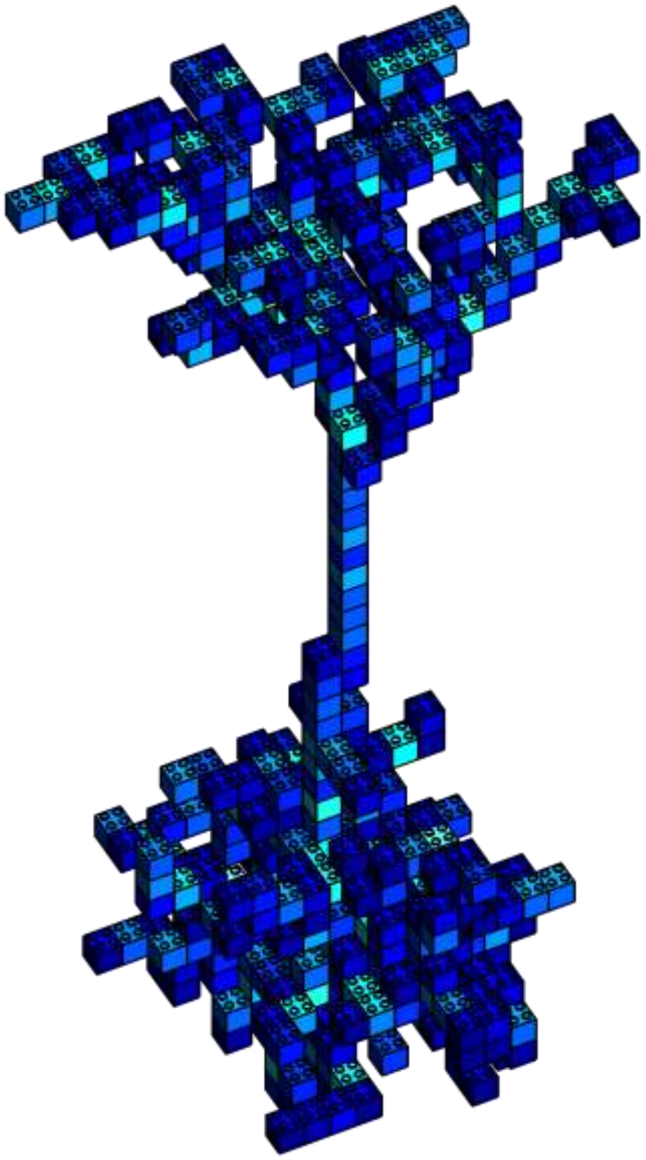

plot_tree plots a tree

HP = plot_tree (intree, color, DD, ipart, res, options)

Plots a directed graph contained in tree structure ***intree***. Many settings allow to play with the output results. Colour handling is different on line plots than on patchy '-b' or '-p'. Even if metrics are nonexistent “plot_tree” will plot its best guess for a reasonable tree (see "xdend_tree"). Line plots are always slower than any patch display. Plot is offset by XYZ 3-tupel ***DD*** and coloured with RGB 3-tupel (or *Nx1* vector, a colour value per node) ***color***. ***ipart*** is an optional index for a sub-set of nodes whose segements are to be plotted. ***res*** determines the resolution of the cylinders, with 8 points as a default. ***options*** are ‘-2l’ or ‘-3l’ for 2D or 3D line plots, ‘-2q’ or ‘-3q’ for 2D or 3D arrow plots (quiver); see comments in “plot_tree” for more details. ***HP*** is the handle to the graphical object.

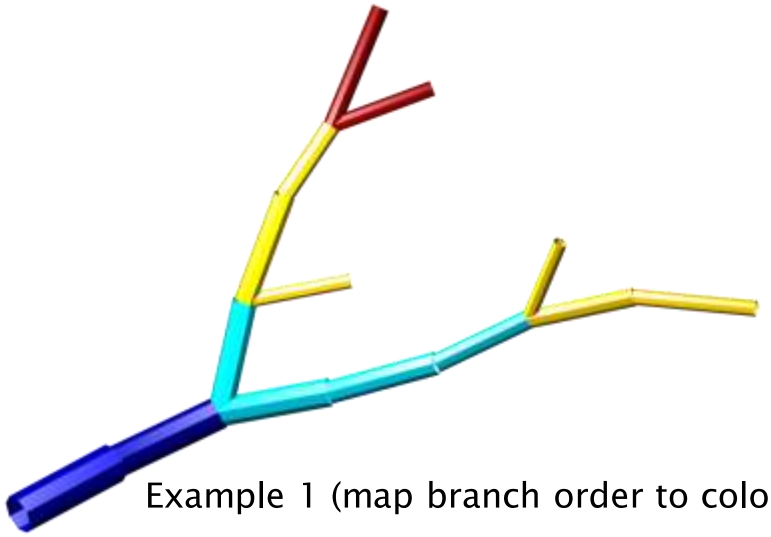

Example 1 (map branch order to colour):

```
>> plot_tree (sample2_tree,
BO_tree (sample2_tree))
```

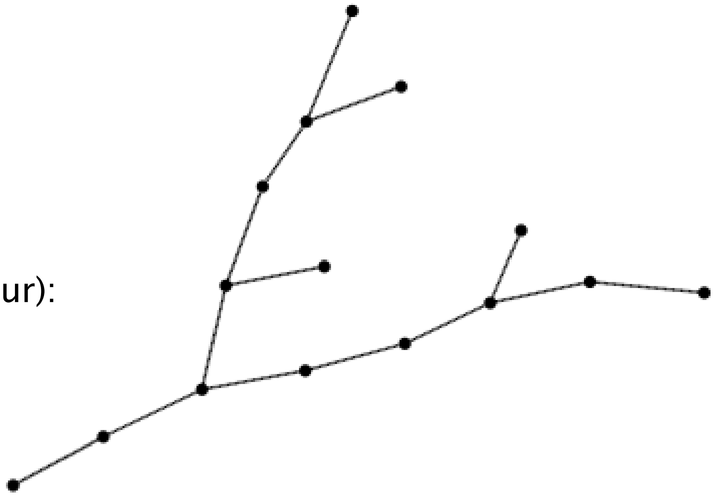

Example 2 (as a line):

```
>> HP = plot_tree (sample2_tree,
[], [], [], [], '-3l');
>> set (HP, 'marker', '.');
```

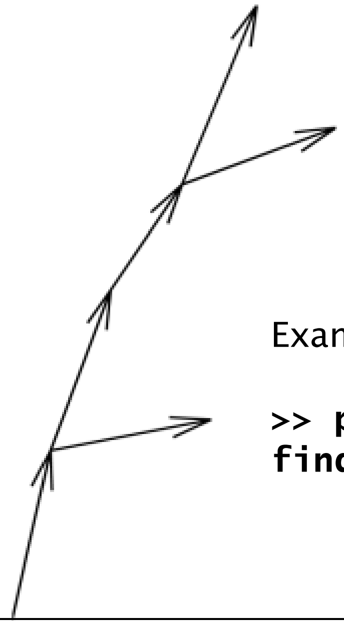

Example 3 (quiver plot of sub-tree of node #10):

```
>> plot_tree (sample2_tree, [], [],
find (sub_tree (sample2_tree, 10)), [], '-3q');
```

written partly by Friedrich Forstner 2008

plotsect_tree plots selected path

`[HP, indy] = plotsect_tree (intree, sect, color, DD, options, ipar)`

Draws a line through a section (or path) out of a tree ***intree***. The section must be a directed path away from the root as obtained by "dissect_tree" for example (or "ipar_tree"). Plot is offset by XYZ 3-tupel ***DD*** and coloured with RGB 3-tupel ***color***. Providing parental paths matrix ***ipar*** (see "ipar_tree") speeds up the plotting significantly. ***HP*** is the handle to the graphical object. ***indy*** outputs the nodes on the path.

Example:

```
>> [HP indy] = plotsect_tree (sample2_tree, [1 13], [1 0 0]);
```

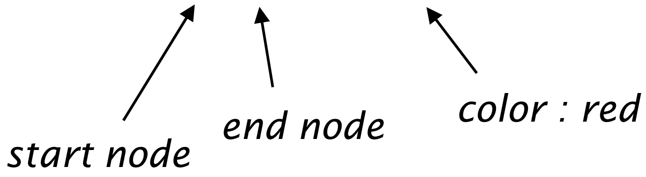


```
>> indy

[13, 12, 11, 10, 3, 2, 1]

>> set (HP, 'marker', '.');
```

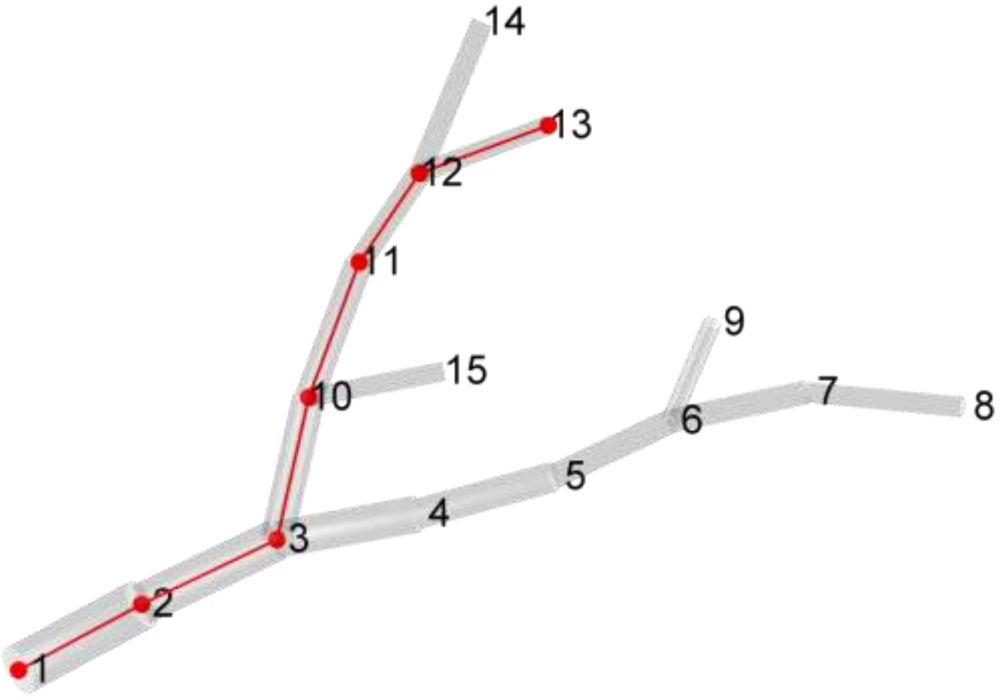

pointer_tree draws pointers (electrodes)

HP = pointer_tree (intree, inodes, llen, color, DD, options)

Draws pointers away at random positive deflections of length ~***llen*** from nodes ***inodes*** in tree ***intree***. Pointer are offset by XYZ 3-tupel ***DD*** and coloured with RGB 3-tupel ***color***. By default these are simple spheres. With options '-l' or '-v' the pointers look a bit like thin or thick electrodes respectively. ***HP*** is the handle to the graphical object.

Example:

green spheres around nodes #8, #5 and #2:

```
>> HP = pointer_tree (tree, [8 5 2], [], [0,1,0]);
```

red "patch" electrode on node #4

```
>> HP = pointer_tree (tree, 14,     100, [1 0 0], [], '-v');
```

blue transparent "sharp" electrode on node #15

```
>> HP = pointer_tree (tree, 15,      50, [0 0 1], [], '-l');
>> set (HP, 'facealpha',.1);
```

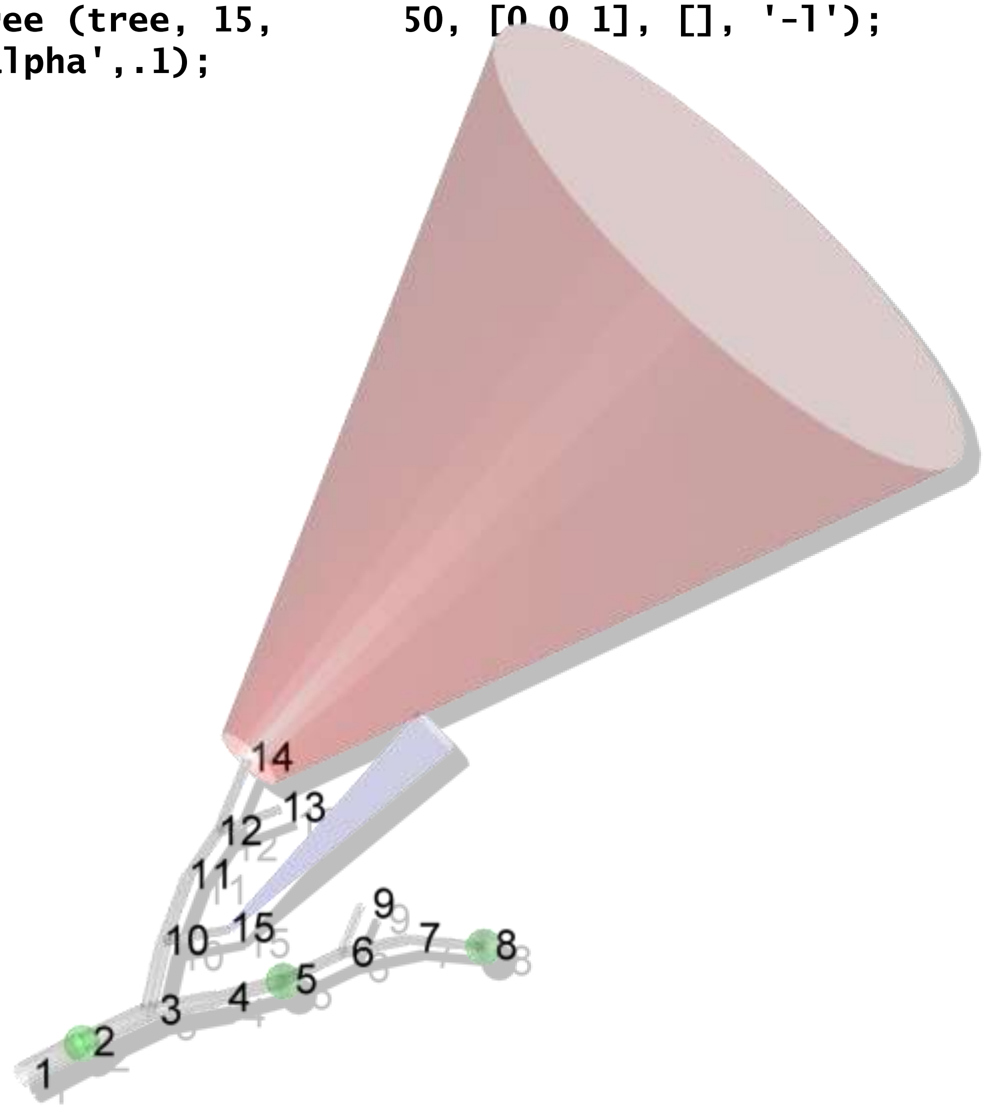

spread_tree spreads out trees

[DD trees] = spread_tree (intrees, dX, dY, options)

Creates a cell array ***DD*** of same organization as 2(or 1)-depth cell array ***intrees*** with *X*-, *Y*- and *Z*-coordinates to display trees spread over the XY-surface of a figure. ***DD*** is then an input to most functions in the "graphical" folder of the TREES toolbox (see "plot_tree" for example). If nesting level is 2 deep, trees are separated in groups additionally. ***dX*** and ***dY*** determine the minimal distance in *X* and *Y* respectively between two trees. ***trees*** output contains all trees in ***intrees*** translated according to ***DD***.

Example:

```
>> dLPTCs = load_tree ('dLPTCs.mtr');

>> spread_tree (dLPTCs{1})
```

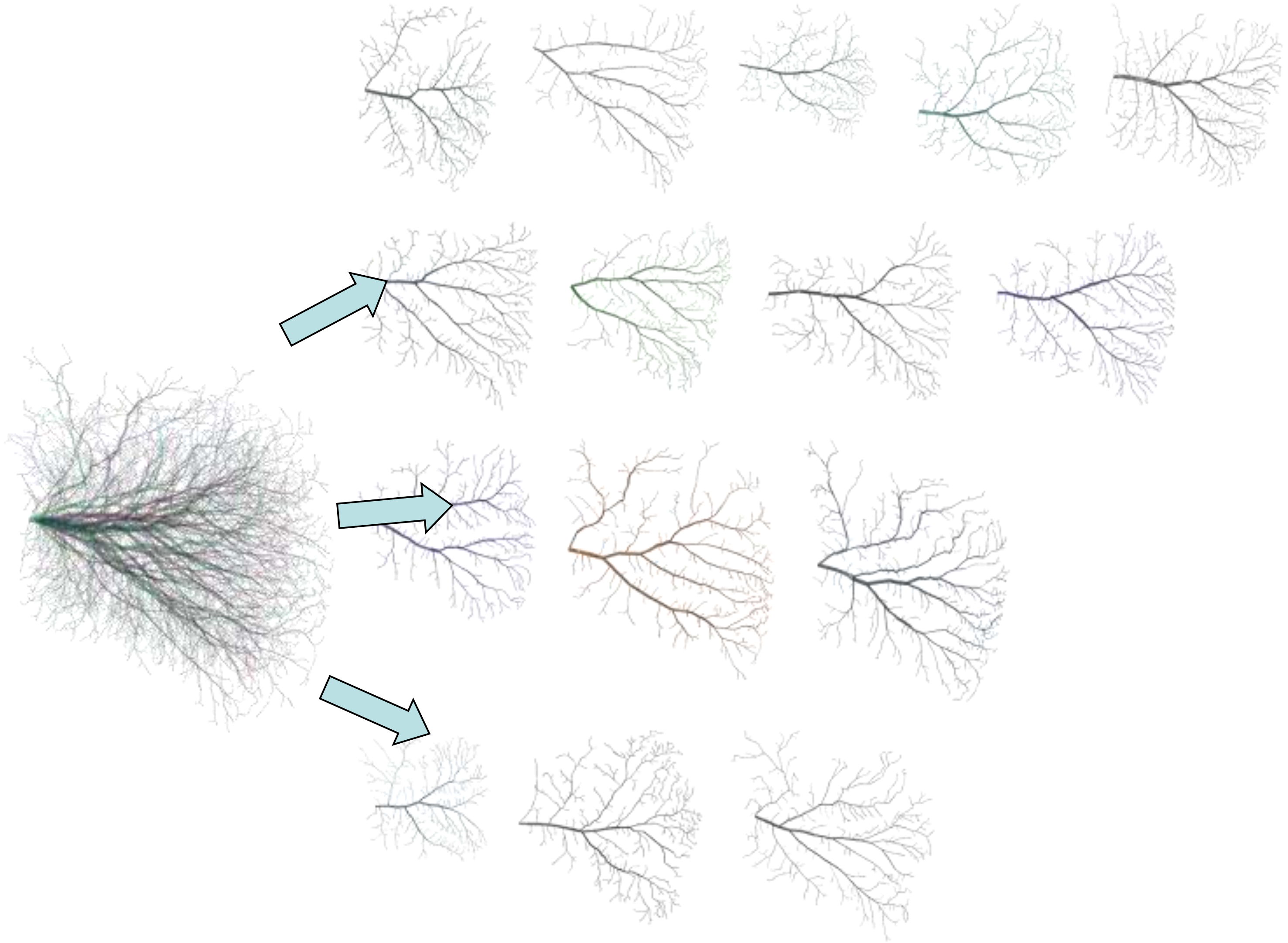

vhull_tree voronoi based subdivision

```
[HP VO KK vol] = vhull_tree (intree, v, points, ipart, DD, options)
```

Subdivides a tree ***intree*** in convex polygons using the voronoi-algorithm. Returns one patch around each node. Patches can be coloured with *Nx1* vector ***v***. Boundary voronoi patches would go ad infinitum. Therefore a set of boundary points prevents this, by default these are calculated according to the isosurface from "hull_tree". vhull plot is offset by XYZ 3-tupel ***DD*** and ***ipart*** is an optional index for using a sub-set of nodes only. ***HP*** is the handle to the graphical object. ***VO*** and ***KK*** are coordinates and convex hull indices of each individual polygon. ***vol*** outputs an *Nx1* vector of volume values (or surface values if 2D) for the output polygons.

Example 1:

```
>> vhull_tree (sample2_tree, BO_tree (sample2_tree))
```

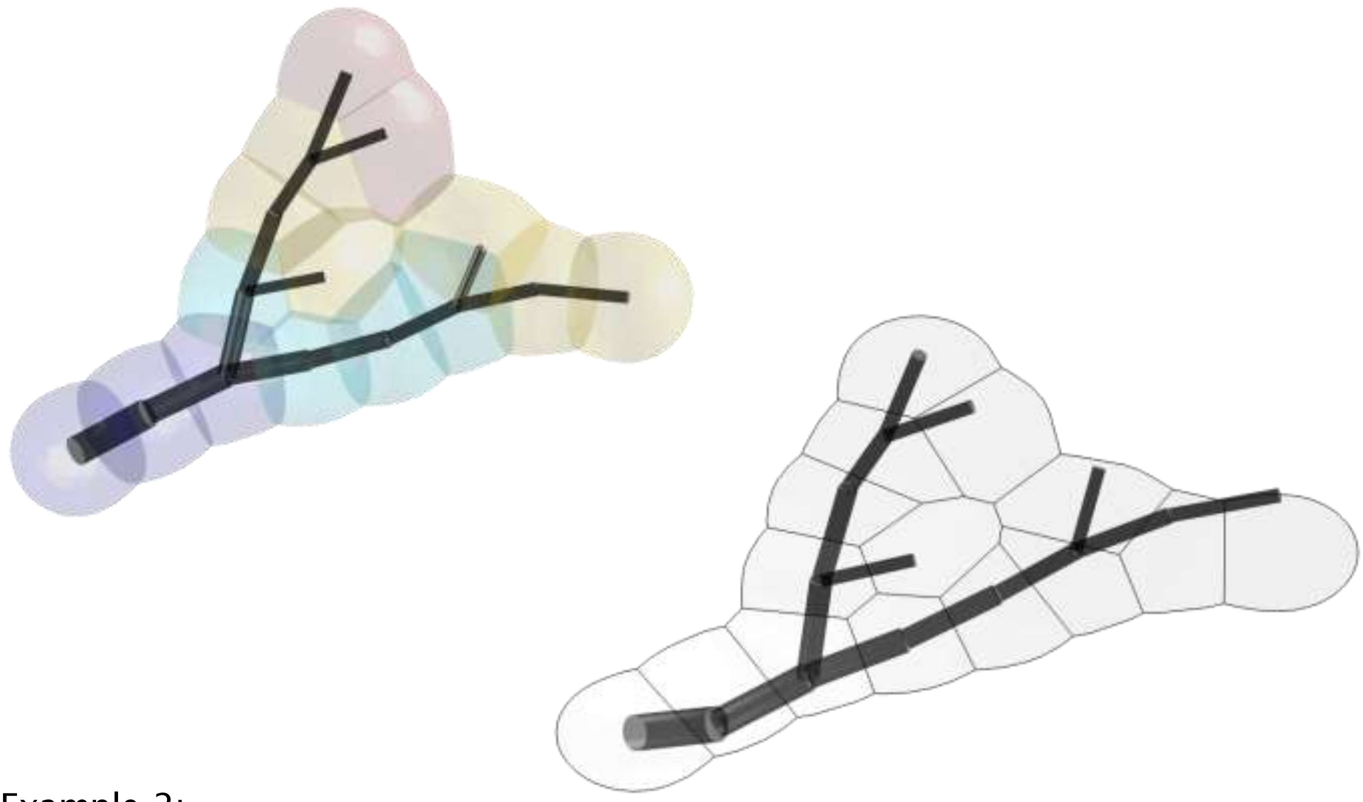

Example 2:
First get the points for the 2D boundary at 15 μm isodistance then 2D vhull

```
>> c = hull_tree (sample2_tree, 15, [], [], [], '-2d');

>> [Xt Yt] = cpoints (c);

>> HP = vhull_tree (tree, [], [Xt Yt], [], [], '-2d -s');
```

vtext_tree write text at node locations

```
HP = vtext_tree (intree, v, color, DD, crange, ipart, options)
```

Displays text numbers or text in the *Nx1* vector ***v*** at the coordinates of the nodes of tree ***intree***. By default ***v*** contains the numbers 1 to *N*, a way to display node indices. Plot is offset by XYZ 3-tupel ***DD*** and coloured with RGB 3-tupel (or *Nx1* vector, a colour value per node) ***color***. ***ipart*** is an optional index for a sub-set of nodes to be plotted. ***crange*** restricts the colour limits. ***HP*** is the handle to the text object.

Example:

```
>> HP = vtext_tree (sample2_tree, ('hello!!!!')', eucl_tree (sample2_tree), [], [], 1:9)
```

each new line is attributed to a new node

selected nodes

colour according to Euclidean distance

```
>> set (HP, 'fontname', 'times new roman');
```

output can be modified as any other text handle

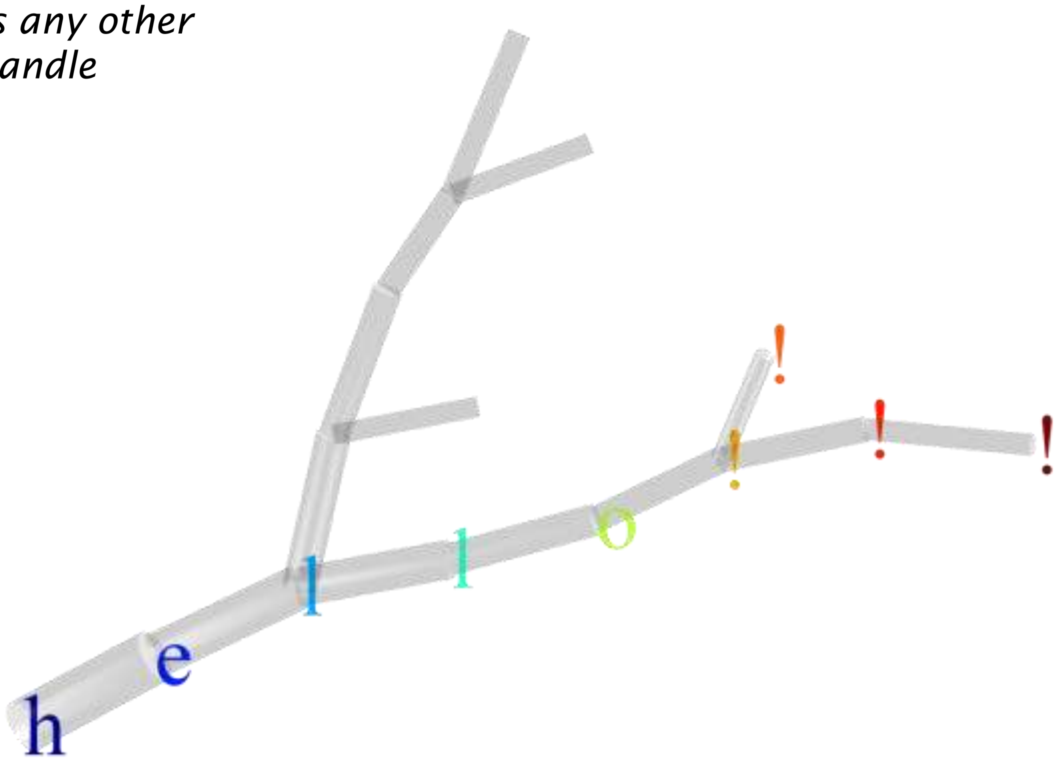

xdend_tree dendrogram x-coordinates

[xdend tree] = xdend_tree (intree, options)

Returns an *Nx1* vector ***xdend*** of x-values useful for constructing the dendrogram of tree ***intree***. Each element's x-value is set in the middle of the labelled terminal children (maximum index + minimum index)/2. Optional output is a correlate (equivalent) tree to ***intree*** with same branch lengths and topology but with standard and sorted metrics. Branch overlap is also avoided if possible. ***intree*** must be conform to BCT format. If unsure just apply "repair_tree" beforehand.

Example:

```
>> xdend_tree (sample2_tree)'

[3, 3, 3, 1.5, 1.5, 1.5, 1, 1, 2, 4, 3.5, 3.5, 3, 4, 5]
```

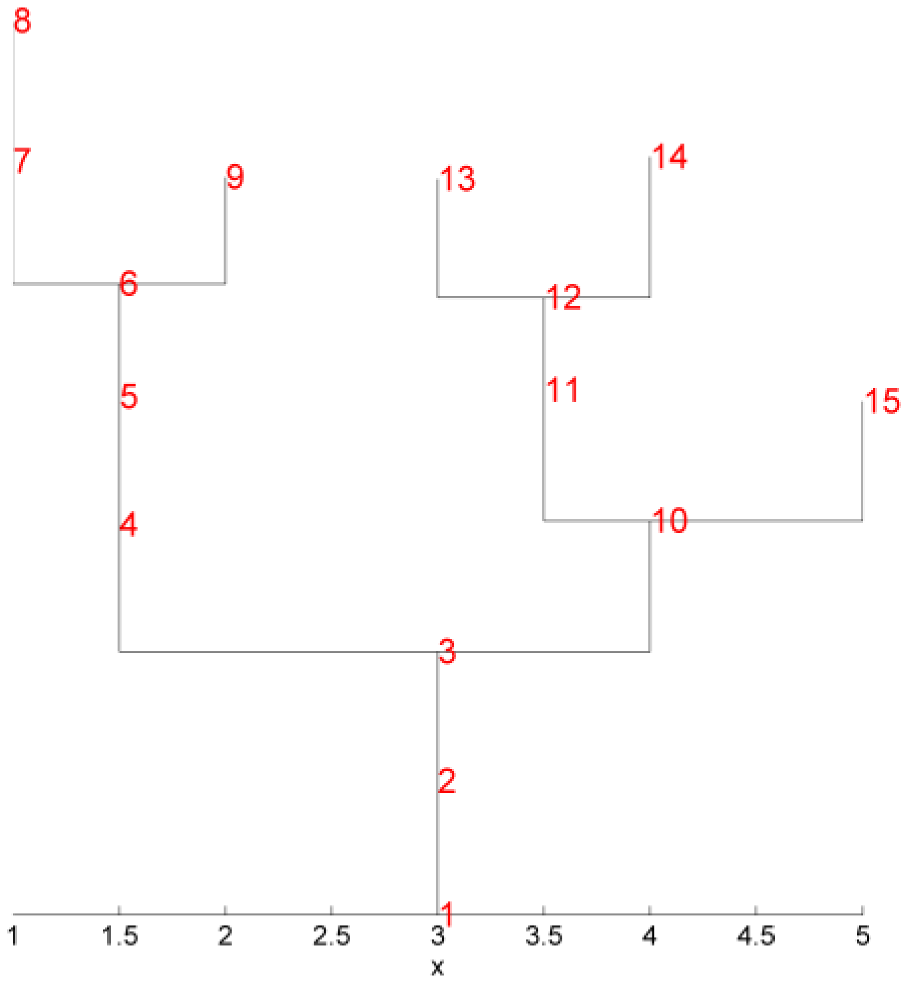

xplore_tree tree exploration plots

[HT HP] = xplore_tree (intree, options, color, DD)

Plots different representative exploration plots for a tree ***intree***. Three options exist (see below). When it makes sense, plot is offset by XYZ 3-tupel ***DD*** and coloured with RGB 3-tupel (or *Nx1* vector, a colour value per node) ***color*** as in "plot_tree". ***HT*** contains handles to font objects while ***HP*** contains the handles to the graphical objects.

Example 1:
graph representation of the tree

>> xplore_tree (sample2_tree)

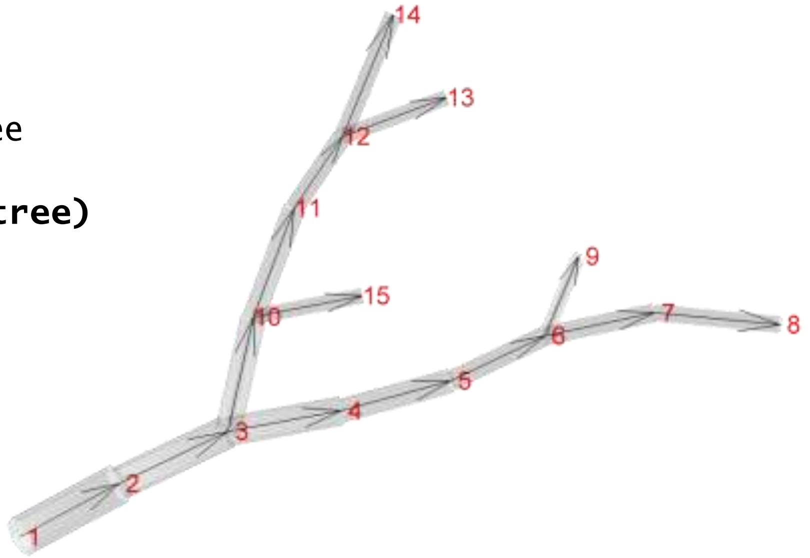

Example 2:
regions of a tree are highlighted

>> xplore_tree (sample2_tree, '-2')

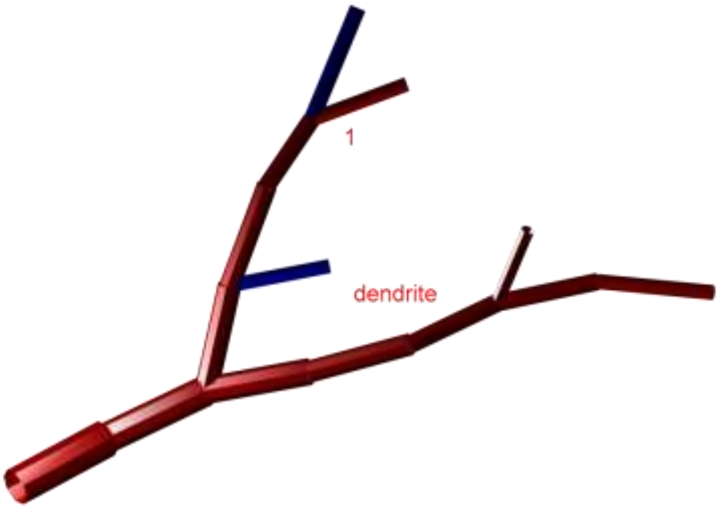

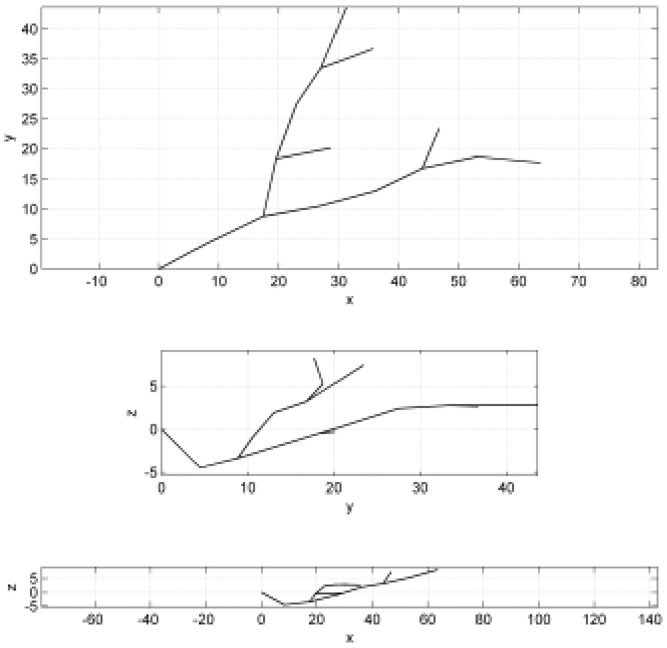

Example 3:
tree plotted in the three planes xy yz xz

>> xplore_tree (sample2_tree, '-3')

construct

functions to generate artificial trees

allBCTs_tree	outputs all possible trees with N nodes
BCT_tree	creates a tree from BCT string
clean_tree	deletes improbable nodes
clone_tree	clones a tree type using the MST constructor
cplotter	plots a contour
cpoints	returns points on a contour
gscale_tree	trees spanning field scaling
in_c	applies inpolygon on contour
isBCT_tree	checks if tree is conform to BCT
jitter_tree	adds noise to node coordinates
MST_tree	minimum spanning tree based constructor
quaddiameter_tree	maps quadratic diameter tapering on tree
quadfit_tree	fits quadratic diameter taper to tree
rpoints_tree	weighted rand distribution of points in hull
smooth_tree	smoothens node coordinates on long paths
smoothbranch	smoothens node coordinates on branch
soma_tree	adds thick diameters around root
spines_tree	add spines

allBCTs_tree all trees with *N* nodes

```
[BCTs BCTtrees] = allBCTs_tree (N, options)
```

Outputs in ***BCTs*** all possible non-isomorphic BCT strings with ***N*** nodes (see introduction section “BCT formalism”). On demand, cell array of trees ***BCTtrees*** is calculated whose trees correspond to the BCT strings using sensible metrics. This uses the equivalent tree method from “BCT_tree”.

Example:

```
>> allBCTs_tree (6, '-s -w')
```

all trees with 6 nodes

```
[1  1  1  1  1  0
 1  1  1  2  0  0
 1  1  2  1  0  0
 1  2  1  0  1  0
 1  2  1  1  0  0
 1  2  2  0  0  0
 2  1  1  0  1  0
 2  1  1  1  0  0
 2  1  2  0  0  0
 2  2  0  0  1  0
 2  2  1  0  0  0]
```

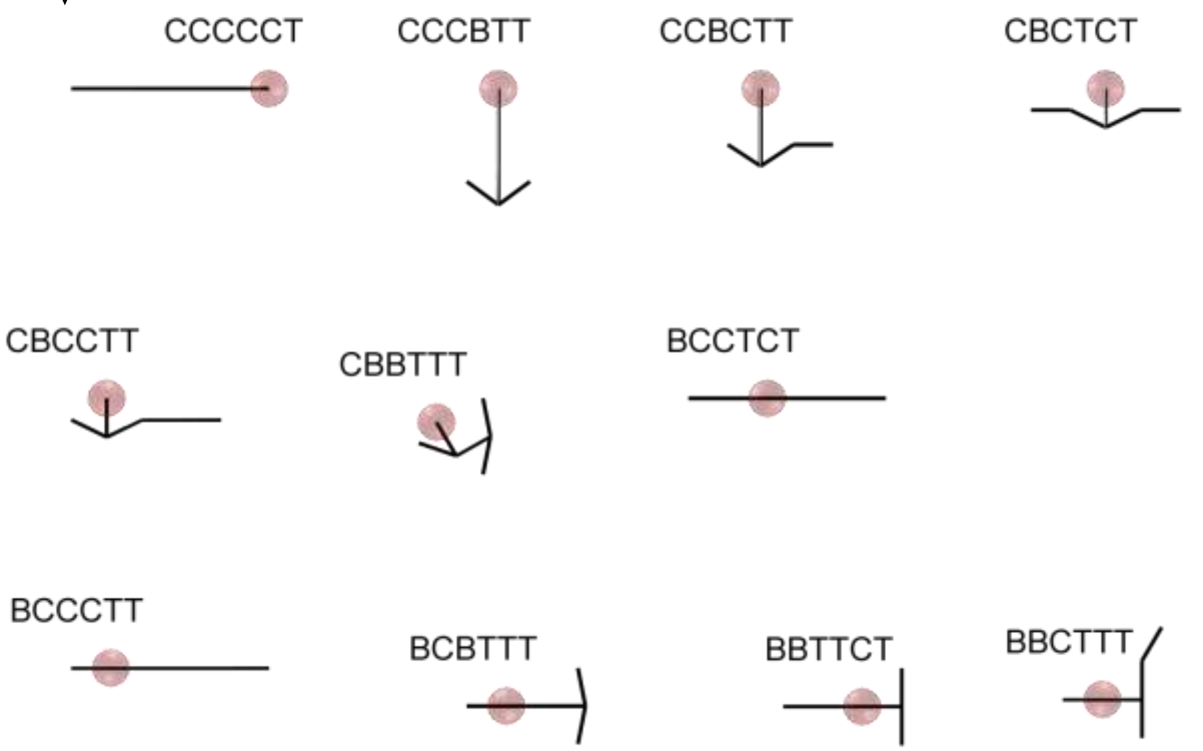

BCT_tree tree from BCT string

```
tree = BCT_tree (BCT, options)
```

Finds the directed adjacency matrix from ***BCT*** a horizontal vector (0: terminal, 1: continuation, 2: branch). The algorithm uses a stack (see introduction section on "BCT formalism"). Proposes artificial metrics according to a circular dendrogram (see "xdend_tree", but used as angles). This can be seen as an equivalent tree (electrotonically) to any real tree whose BCT string is known.

Examples:

```
>> BCT_tree ([1 2 1 0 2 0 0])
```

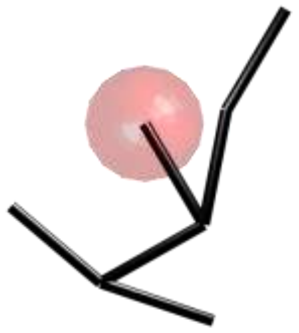

```
>> dA = BCT_tree ([1 2 1 0 2 0 0], '-dA')
```

	1	2	3	4	5	6	7
1	0	0	0	0	0	0	0
2	1	0	0	0	0	0	0
3	0	1	0	0	0	0	0
4	0	0	1	0	0	0	0
5	0	1	0	0	0	0	0
6	0	0	0	0	1	0	0
7	0	0	0	0	1	0	0

if options string contains 'dA' only the adjacency matrix is calculated.

clean_tree deletes improbable nodes

```
tree = clean_tree (intree, radius, options)
```

Cleans tree ***intree*** of improbable nodes (after e.g. automated reconstruction or artificial generation of a tree structure). Termination points in close vicinity of other nodes on a different branch will be deleted and very short terminal branches as well. The "close vicinity" depends on the radius of the "other node“ and on the input parameter ***radius***. Consecutive calls of this function can be useful.

Example:

```
>> clean_tree (sample2_tree, 20);
```

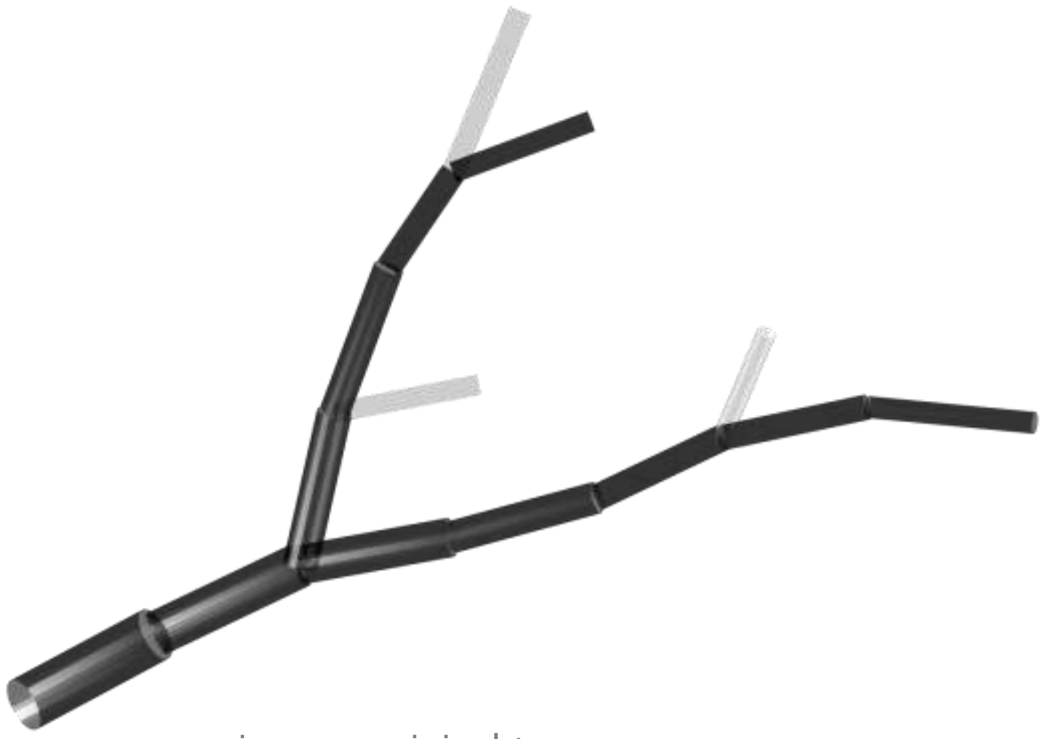

in gray: original tree

in black: cleaned tree

clone_tree MST-based cloning

```
trees = clone_tree (intrees, num, bf, options)
```

Creates as set of ***num*** trees ***trees*** similar to an input set of trees ***intrees*** by distributing points randomly in the spanning fields of the average ***intrees***, scaling them within the variance of ***intrees*** and connecting them with “MST_tree”, the minimum spanning tree constructor. “MST_tree” requires the balancing factor ***bf*** between minimization of path length and total wire length.

Cloning VS4 cells
Examples:

```
>> dLPTCs = load_tree ('dLPTCs.mtr');

>> trees = clone_tree (dLPTCs{5})
```

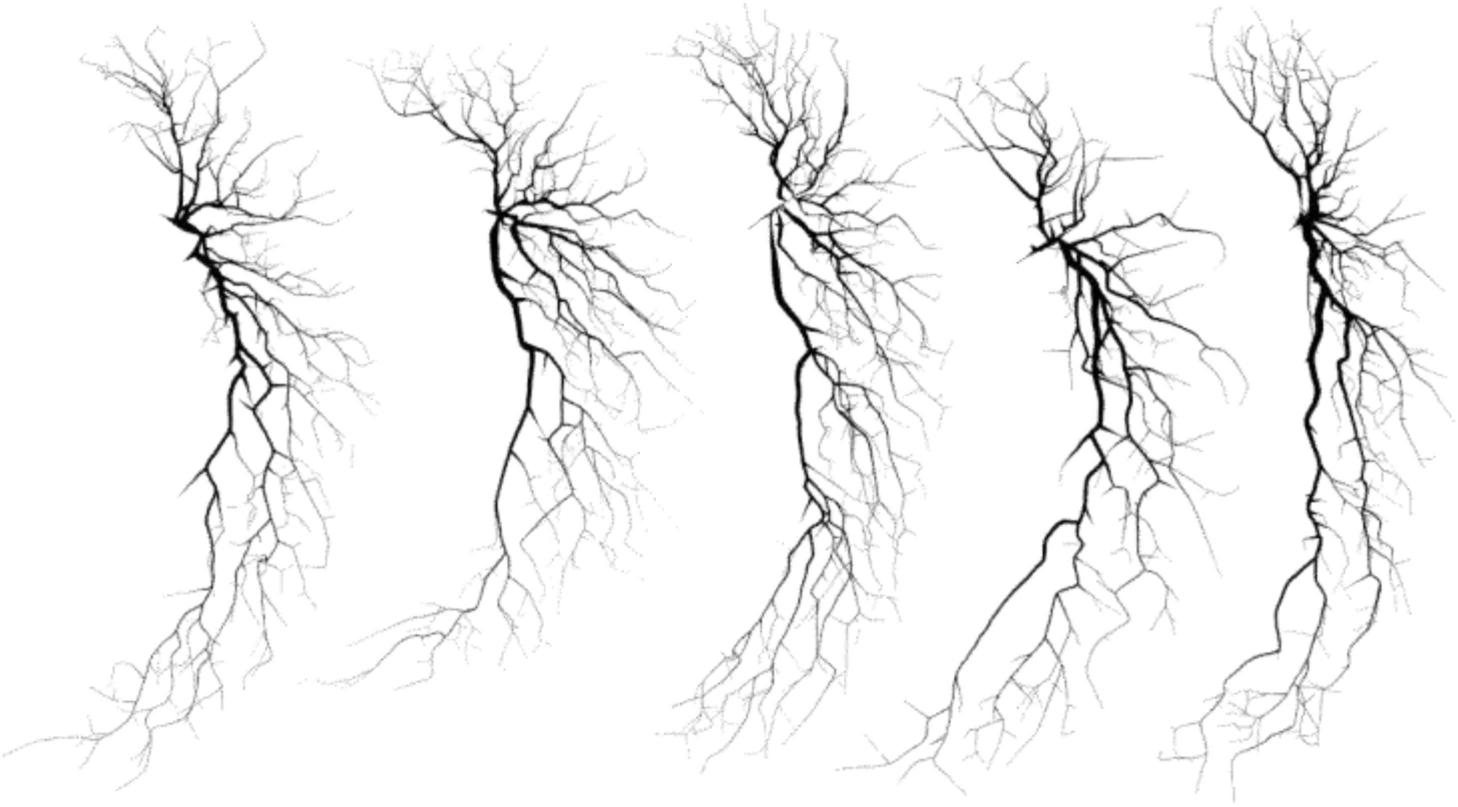

This is a good opportunity to mention that specific cloning should be adapted to the individual properties of the trees to be cloned. In this case much better results can be obtained (just out of reasons of computation) by using the fact that the trees are flat or by fitting the amount of jitter or taper in more sophisticated ways (not implemented in clone_tree for speed reasons). clone_tree uses „gscale_tree“ to fit these parameters, a very primitive but computationally efficient way to obtain them.

cplotter plots a contour

HP = cplotter (c, color, DD)

Plots a 2D contour ***c*** obtained e.g. from "contourc" (see Matlab function). A contour ***c*** is defined by:

```
c = [contour1          x1 x2 x3 ... contour2          x1 x2 x3 ...;
    #number_of_pairs  y1 y2 y3 ... #number_of_pairs y1 y2 y3 ...]'
```

"hull_tree" can for example produce such a contour and this can be used to describe the spanning field of a neuronal tree in the construction process. The contour plot is offset by XYZ 3-tupel ***DD*** and coloured with RGB 3-tupel ***color***. ***HP*** is the handle to the graphical object.

Examples:

```
>> for ward = 1:10,
>>    c = hull_tree (sample2_tree, ward, [], [], [], '-2d');
>>    HP = cplotter (c, rand (1, 3), 2*[ward ward ward])
>> end
```

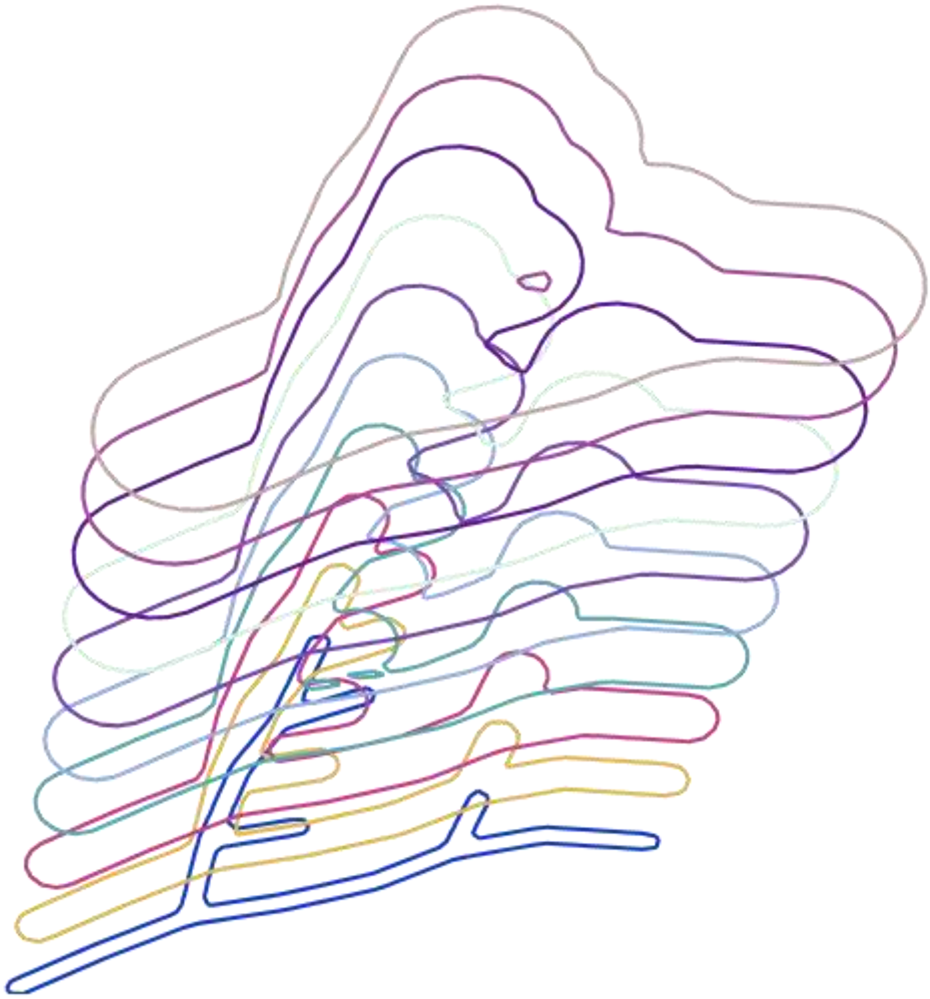

cpoints returns points on a contour

[X, Y] = cpoints (c)

Returns the point coordinates x and y from a contour ***c*** (see Matlab function "contourc") into vectors ***X*** and ***Y***. A contour is defined by:

```
c = [contour1            x1 x2 x3 ... contour2            x1 x2 x3 ...;
    #number_of_pairs     y1 y2 y3 ... #number_of_pairs    y1 y2 y3 ...]'
```

"hull_tree" can for example produce such a contour and this can be used to describe the spanning field of a neuronal tree in the construction process.

Example:

```
>> [X, Y] = cpoints (c); plot (X, Y, 'k.');
```

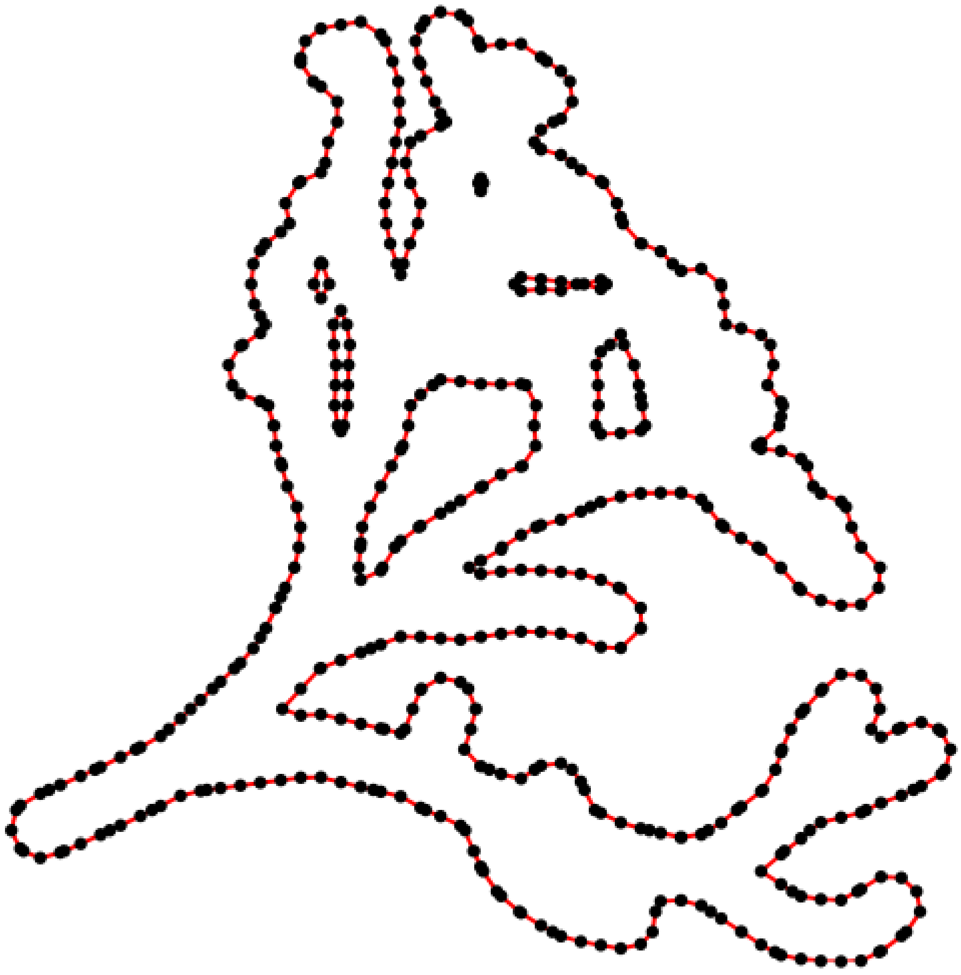

gscale_tree spanning field scaling

```
[spanning ctrees] = gscale_tree (intrees, options)
```

Extracts region by region features from a group of trees ***intrees*** which are sufficient to constrain the artificial generation of trees similar to the original group. Is based on the assumption that the density of topological points on the trees are more or less scalable. The result is a structure ***spanning*** with some info about the spanning fields of the individual regions throughout the trees. ***ctrees*** contains the scaled trees.

Example:

```
>> dLPTCs = load_tree ('dLPTCs.mtr');

>> [spanning ctrees] = gscale_tree (dLPTCs{1})
```

each info per region and tree

```
 regions: {'axon'  'dendrite'}
   xlims: {[15x2 double]  [15x2 double]}
   ylims: {[15x2 double]  [15x2 double]}
   zlims: {[15x2 double]  [15x2 double]}
   xmass: {[15x1 double]  [15x1 double]}
   ymass: {[15x1 double]  [15x1 double]}
   zmass: {[15x1 double]  [15x1 double]}
      iR: {{1x15 cell}  {1x15 cell}}
     nBT: {{1x15 cell}  {1x15 cell}}
 wrigles: [15x2 double]
  mxdiff: [2x1 double]
stdxdiff: [2x1 double]
  mydiff: [2x1 double]
stdydiff: [2x1 double]
  mzdiff: [2x1 double]
stdzdiff: [2x1 double]
       X: {{1x15 cell}  {1x15 cell}}
       Y: {{1x15 cell}  {1x15 cell}}
       Z: {{1x15 cell}  {1x15 cell}}
   qdiam: {[15x2 double]  [15x2 double]}
    mnBT: [2x1 double]
  stdnBT: [2x1 double]
```

- xlims, ylims, zlims: *region limits*
- xmass, ymass, zmass: *center of mass*
- iR: *Index to nodes belonging to each region*
- nBT: *# branch and termination points*
- wrigles: *parameters for jittering*
- mxdiff … stdzdiff: *Mean and standard deviation of region range*
- X, Y, Z: *all node locations*
- qdiam: *Quadratic tapering parameters*
- mnBT, stdnBT: *mean and standard deviation of # branch and termination points*

in_c checks if points are in contour

[IN ON] = in_c (X, Y, c, dx, dy)

If *c* is a contour obtained from a single isoline contourc (see Matlab function "contourc"), this checks if points with coordinates ***X*** and ***Y*** are located ***IN*** the largest contour or ***ON*** the outer boundaries of the largest contour. A contour is defined by:

```
c = [contour1          x1 x2 x3 ... contour2          x1 x2 x3 ...;
     #number_of_pairs  y1 y2 y3 ... #number_of_pairs y1 y2 y3 ...]‘
```

"hull_tree" can for example produce such a contour and this can be used to describe the spanning field of a neuronal tree in the construction process.

Example:

```
>> X = rand (10000,1)*150-20; Y = rand (10000,1)*150-30;

>> inc = in_c (X, Y, c); cplotter (c, [1 0 0]);

>> plot (X(inc), Y(inc), 'k.'); plot (X(~inc), Y(~inc), 'r.');
```

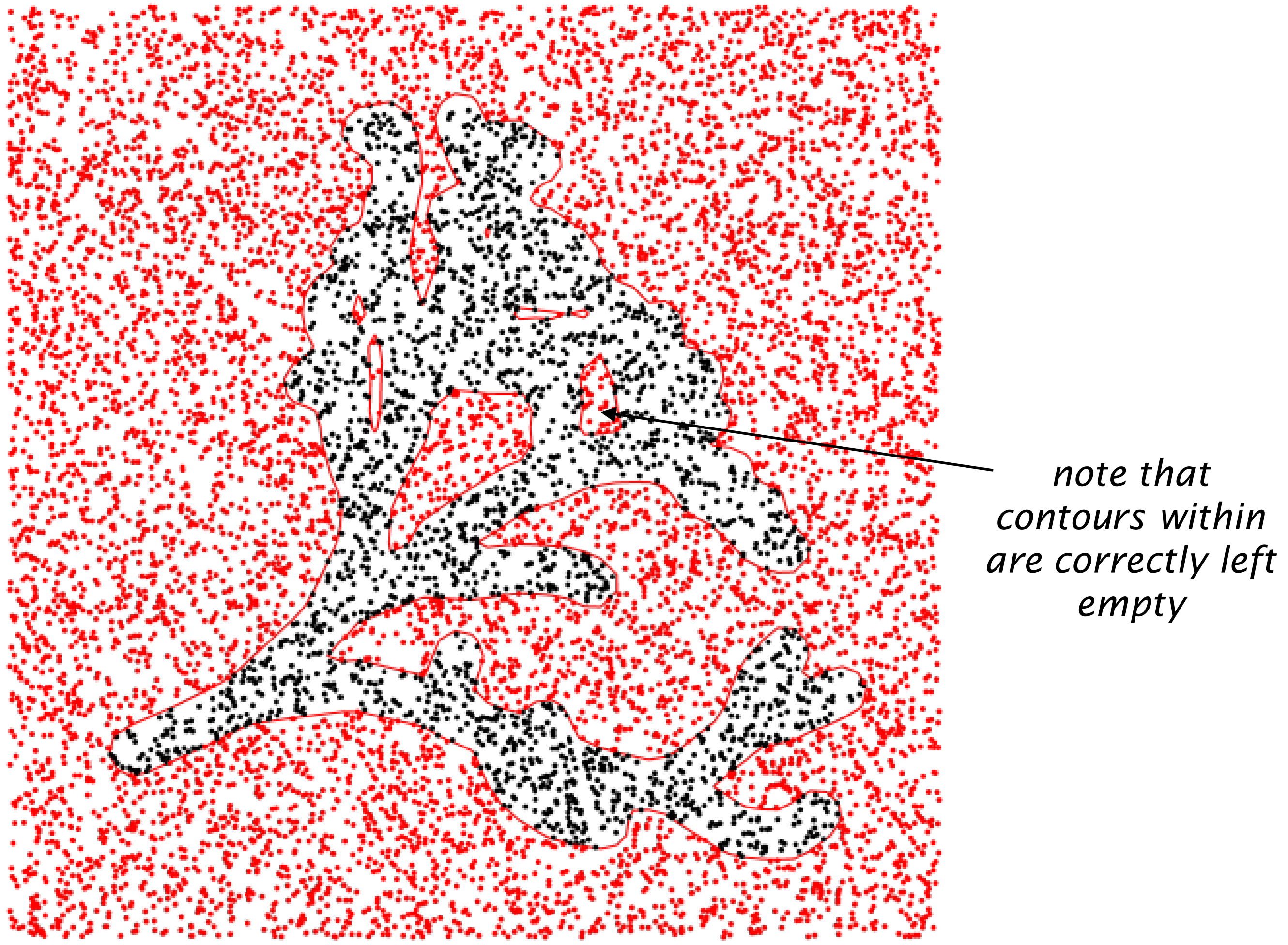

isBCT_tree check if tree conforms to BCT

```
isBCT = isBCT_tree (intree)
```

Checks if tree ***intree*** (or a BCT vector of terminals (0), continuations (1) and branches (2)) is conform to BCT order (see introduction section "BCT formalism").

Examples:

```
>> isBCT_tree ([1 2 1 0 2 0 0])

1

>> isBCT_tree ([1 1 1 1])

0

>> isBCT_tree (sample2_tree)

1
```

this branch does not terminate

No trifurcations allowed!!

jitter_tree jitters node coordinates

```
tree = jitter_tree (intree, stde, lambda, options)
```

Adds spatial noise to the coordinates of the nodes of tree ***intree***. The amplitude of the spatial noise is such that its standard deviation is ***stde*** and it is filtered with length constant ***lambda*** (not in µm but in nodes on the path...).

Examples:
change the amplitude of the spatial noise (stde)

```
>> rtree = resample_tree (sample2_tree, 1);
```

resampling is absolutely required for a homogeneous spatial noise distribution

```
>> jitter_tree (rtree), stde, 10);
```

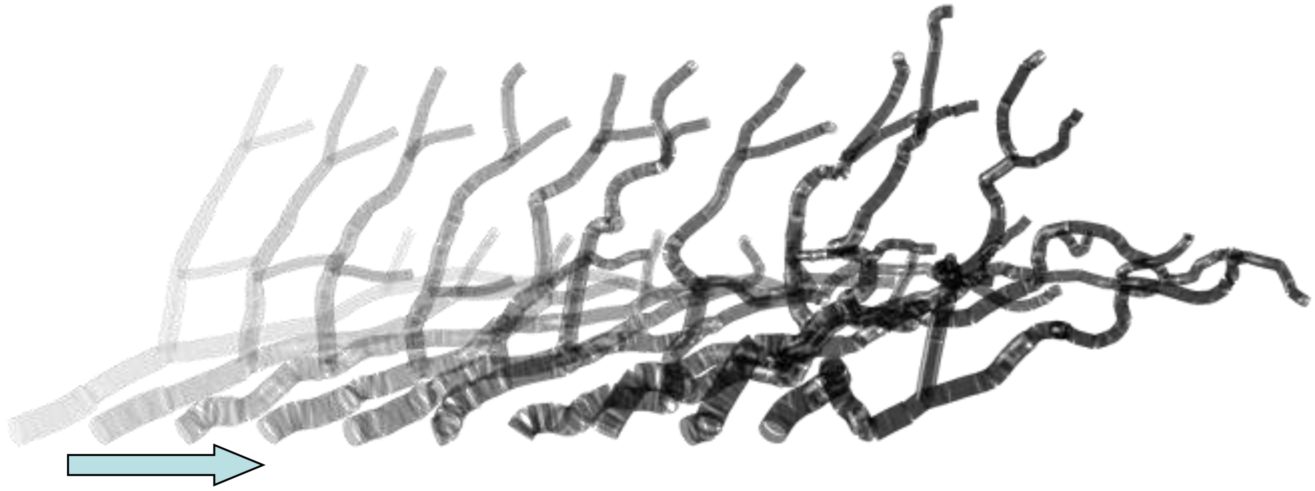

change the length constant of the spatial noise (lambda)

```
>> jitter_tree (rtree, .35, lambda);
```

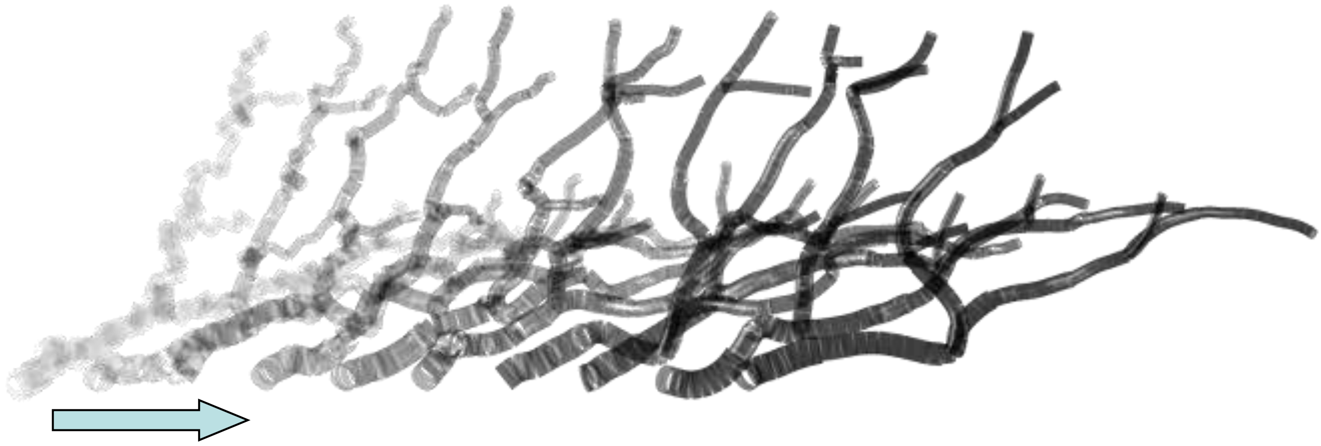

MST_tree minimum spanning tree based constructor

[tree indx] = MST_tree (msttrees, X, Y, Z, bf, thr, mplen, DIST, options)

Connects points defined by coordinates ***X***, ***Y*** and ***Z*** in a competitive manner to starting trees ***msttrees*** (alternatively: starting positions as index into ***X***, ***Y*** and ***Z***) using a greedy algorithm which minimizes locally the total amount of wiring and the path length to the root (with balancing factor ***bf***). A threshold connection distance ***thr*** and a maximal path length in the tree ***mplen*** constrain the resulting tree size. A sparse distance matrix ***DIST*** between nodes is added to the cost function. Don't forget to include input tree nodes into the distance matrix ***DIST***! Option '-b' forbids trifurcations during the process, option '-t' outputs timelapse trees, see option '-s' for a movie.

For speed and memory considerations an area of close vicinity is drawn around each tree as it grows.

Example:
Connect hundred randomly distributed points

```
>> X = rand(100,1)*100; Y = rand(100,1)*100; Z = zeros(100,1);

>> tree = MST_tree (1, [50;X], [50;Y], [0;Z], .5, 50, [], [], 'none');
```

first XYZ value is starting node of tree

first point is (50,50,0)

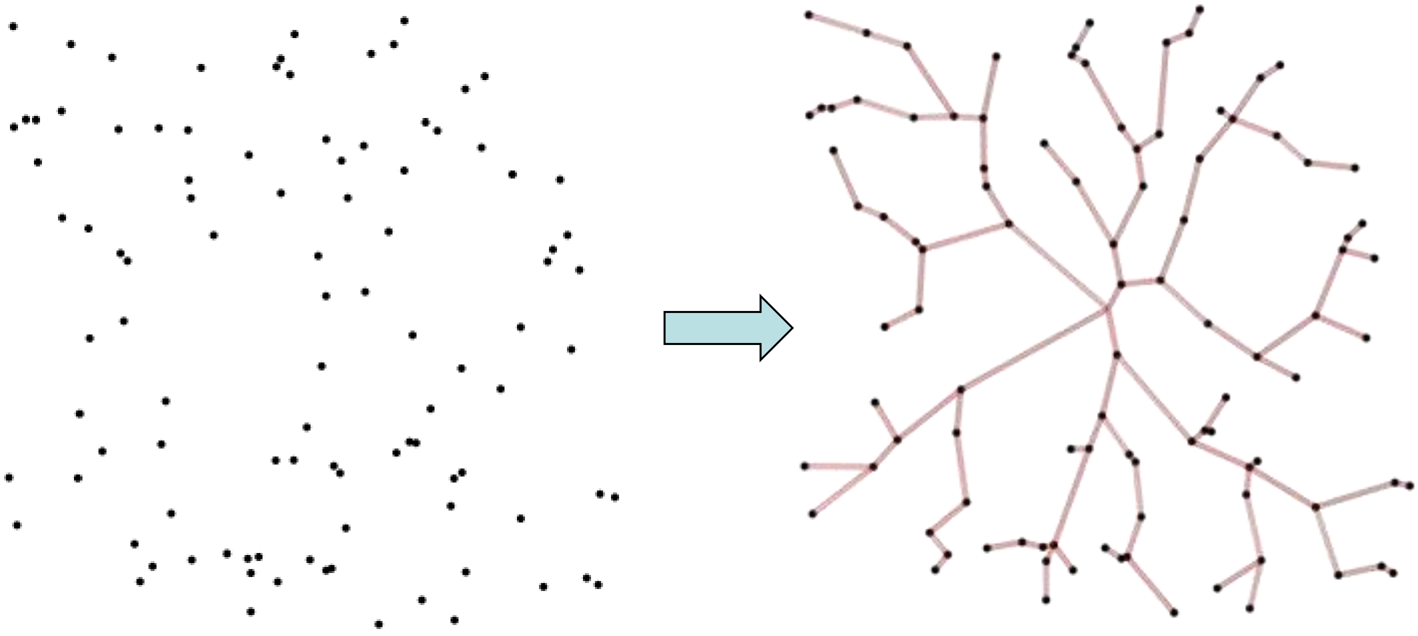

quaddiameter_tree

quadratic diameter tapering

```
tree = quaddiameter_tree (intree, scale, offset, options, P, ldend)
```

Maps quadratic diameter tapering on a given tree structure ***intree***. ***P*** and ***ldend*** are derived in (*Cuntz, Borst and Segev 2007, Theor Biol Med Model, 4:21*). ***P*** is an *numx3* matrix containing the parameters to put in the quadratic equation y = P(1)x^2 + P(2)x + P(3). Each single triplet corresponds to the best fit to a segment of length ***ldend*** (*numx1*) vector. When the quadratic diameter is added, the path from each terminal to the root is compared to its closest in ***ldend***. Then the quadratic equation is chosen according to the index in ***ldend***. This is done for all paths from root to terminal point and for each node the diameter is an average of all local diameters of all paths leading through that node. Choosing parameters (***P*** and ***ldend***) by hand here is tempting but very hard. ***P*** and ***ldend*** depend on the total leak and the minimal diameter: these have to be adjust by the parameters ***scale*** and ***offset*** respectively (see "quadfit_tree").

Examples:
change the scaling of the quadratic tapering (scale)

```
>> rtree = resample_tree (sample2_tree, 1);

>> quaddiameter_tree (rtree, scale, 1)
```

resampling is better for a homogeneous quadratic diameter tapering

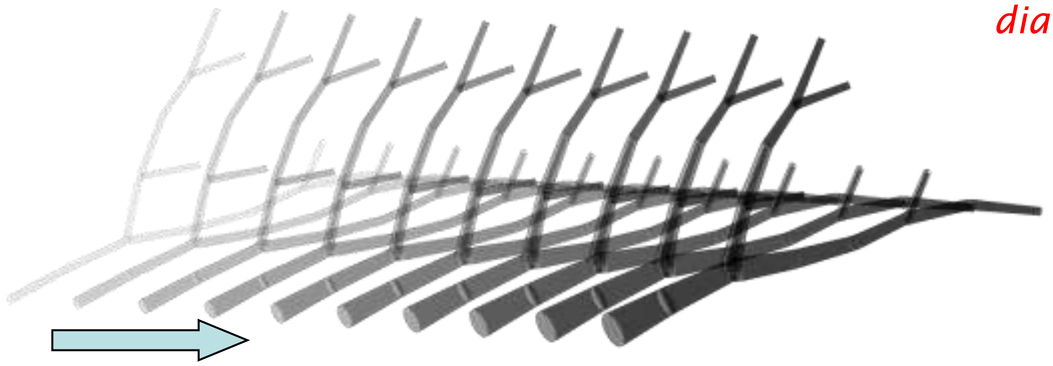

add an offset diameter value to the quadratically tapering diameter (offset)

```
>> quaddiameter_tree (rtree, .4, offset)
```

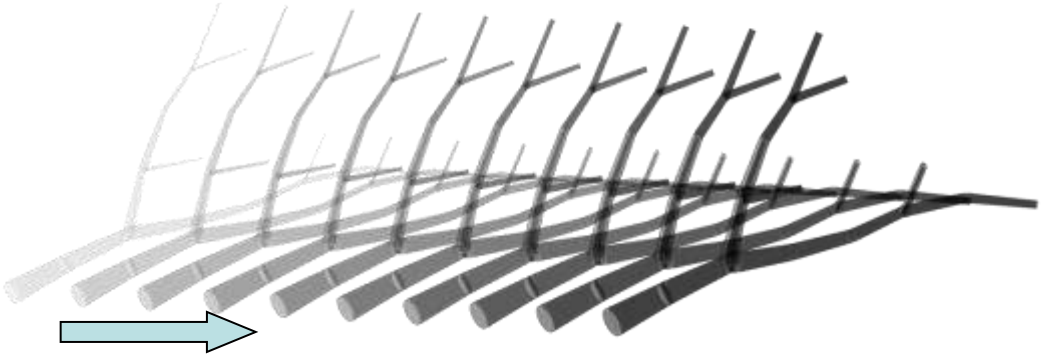

quadfit_tree fit quadratic diameter taper

[P0 tree] = quadfit_tree (intree, options)

To a given tree ***intree*** with diameter values, this fits a quadratic tapering using "quaddiameter_tree" to best fit the original diameters. The output is a scaling and an offset value in ***P0*** for direct input to "quaddiameter_tree". See below how well two parameters simply describe the dendritic quadratic tapering nearly perfectly.

Examples:

```
>> tree = resample_tree (sample2_tree, 1, '-d');

>> [P0 qtree] = quadfit_tree (tree); P1

0.3250    1.2880
```

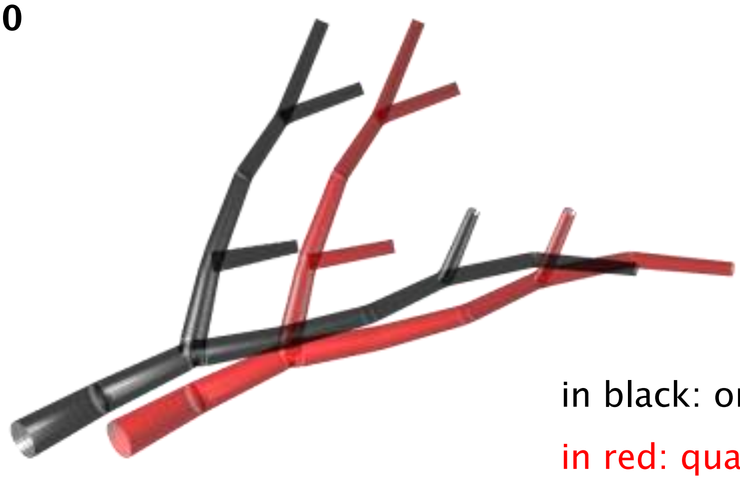

in black: original tree

in red: quadfit diameters

```
>> tree = resample_tree (sample_tree, 1, '-d');

>> [P0 qtree] = quadfit_tree (tree); P1

0.2736    1.0336
```

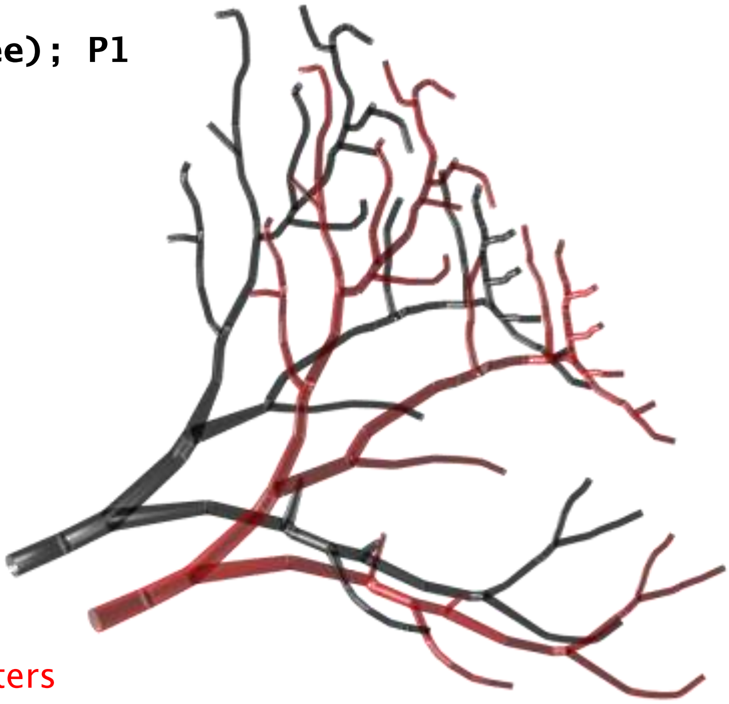

in black: original tree

in red: quadfit diameters

rpoints_tree distribute points within hull

[X, Y, Z, HP] = rpoints_tree (M, N, c, x, y, z, thr, options)

Distributes ***N*** random points in accordance with the density matrix ***M***. Only points within the sharp boundaries of a 2d contour ***c*** are selected (see below). Note that the number of resulting points is therefore typically smaller than ***N***. The boundary can be further reduced by a distance ***thr***, minimal distance that a point needs to be away from any point on the contour. This makes particularly sense if the contour was obtained using "hull_tree" in 2D. The contour (see "contourc") is defined by:

```
c = [contour1         x1 x2 x3 ... contour2         x1 x2 x3 ...;
    #number_of_pairs   y1  y2  y3  ...  #number_of_pairs  y1  y2  y3
...]‘
```

see “hull_tree” to check out how such a contour can be produced.

Examples:
As an example, let‘s get some points which are similarly distributed as an underlying hsn cell:

```
>> tree = hsn_tree;

>> [M dX dY dZ] = gdens_tree (tree, 20, find(B_tree (tree) | T_tree (tree)),'-s');

>> c = hull_tree (tree, 20, [], [], [], '-2d');

>>[X Y Z] = rpoints_tree (M, 1290, [], dX, dY, dZ, 5);
```

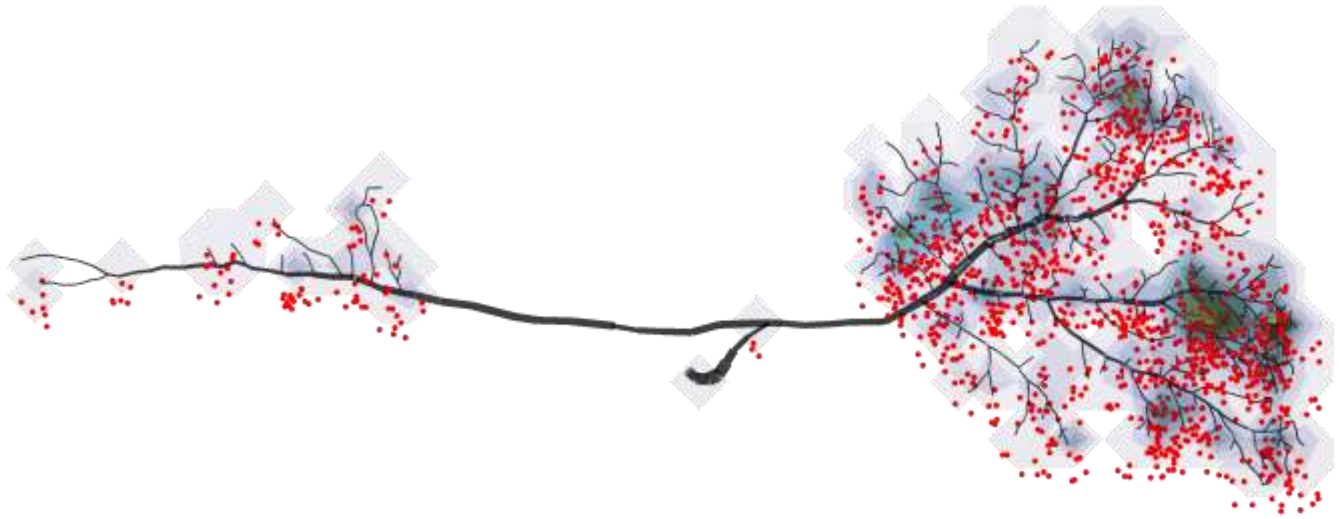

smooth_tree smoothens long branches

```
tree = smooth_tree (intree, pwchild, p, n, options)
```

Smoothens a tree ***intree*** along its longest paths. This changes (shortens) the total length of the branch significantly. First finds the heavier sub-branches (thresholded by .5 to 1 value ***pwchild***) and puts them together to longest paths. Then a smoothing step is applied on the branches individually using ***p***, proportion smoothing (0 to 1, default .9), and ***n***, number of iterations (default 10). "smooth_tree" calls "smoothbranch" but this sub-function can be replaced by any other one of a similar type.

Examples:
change proportion of smoothing (p)

```
>> rtree = resample_tree (sample2_tree, 1);

>> smooth_tree (rtree, .5, p, 5)
```

resampling is better for a homogeneous smoothing

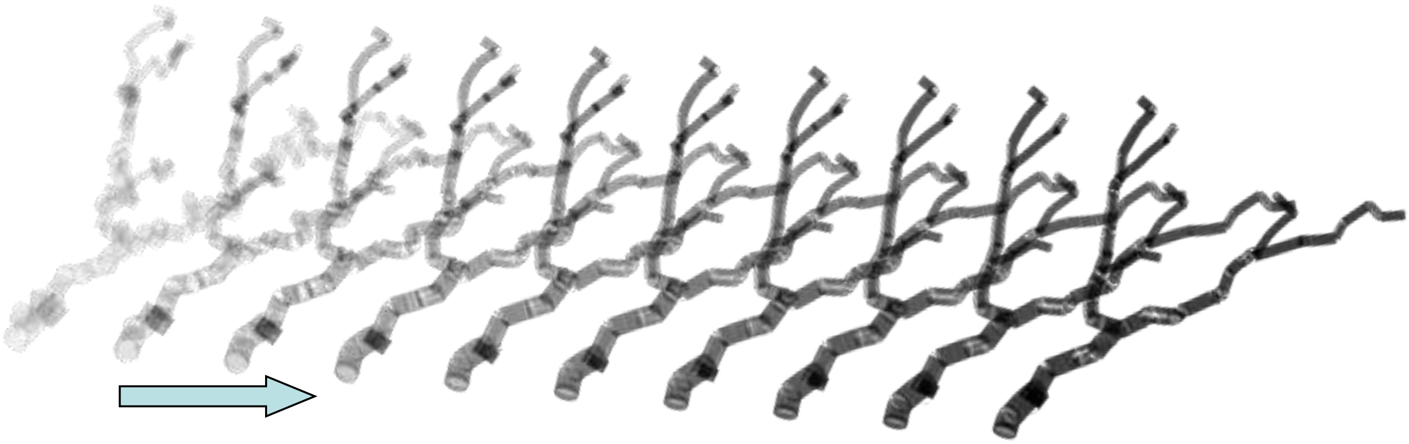

change number of iterations (n)

```
>> smooth_tree (rtree, .5, .9, n)
```

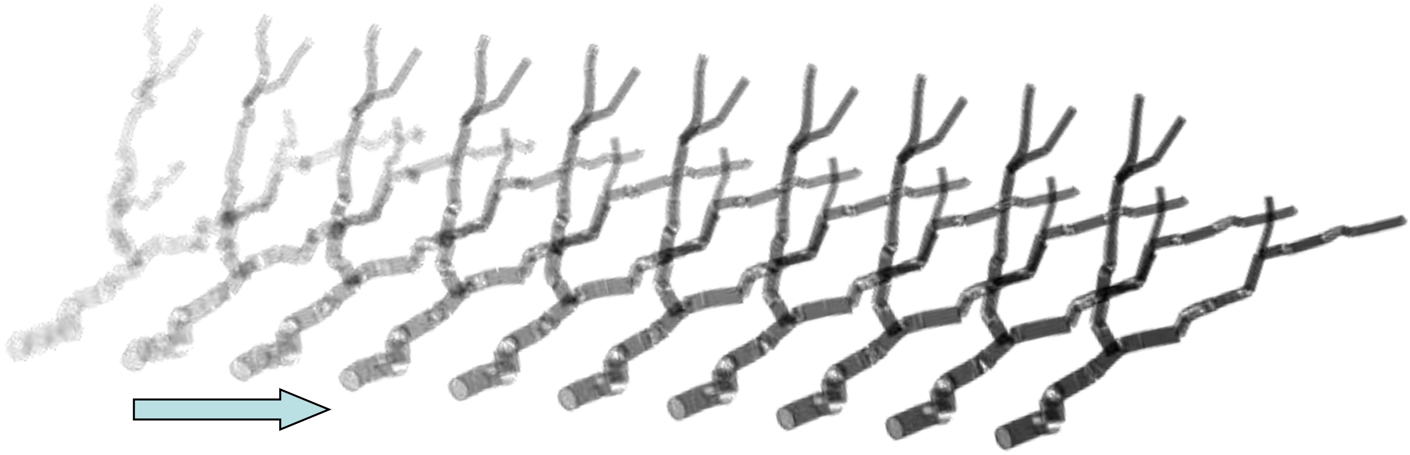

smoothbranch

smoothens points along one path

[Xs, Ys, Zs] = smoothbranch (X, Y, Z, p, n)

Smoothens a branch given by consecutive 3D coordinates ***X***, ***Y*** and ***Z***, in usage by "smooth_tree". This changes (shortens) the total length of the branch significantly. The amount of smoothing is parameterized by ***p***, proportion smoothing (0 to 1, default .9), and ***n***, number of iterations (default 10). *Method: in each triplet of consecutive points, approach the middle point toward the center of the triangle formed by the three points. (this is rather arbitrary and can be improved)*

Example:
increased smoothing (*x*: black → white) on nine different sample branches

>> smoothbranch (X, Y, Z, (*x*-1)/10, *x*)

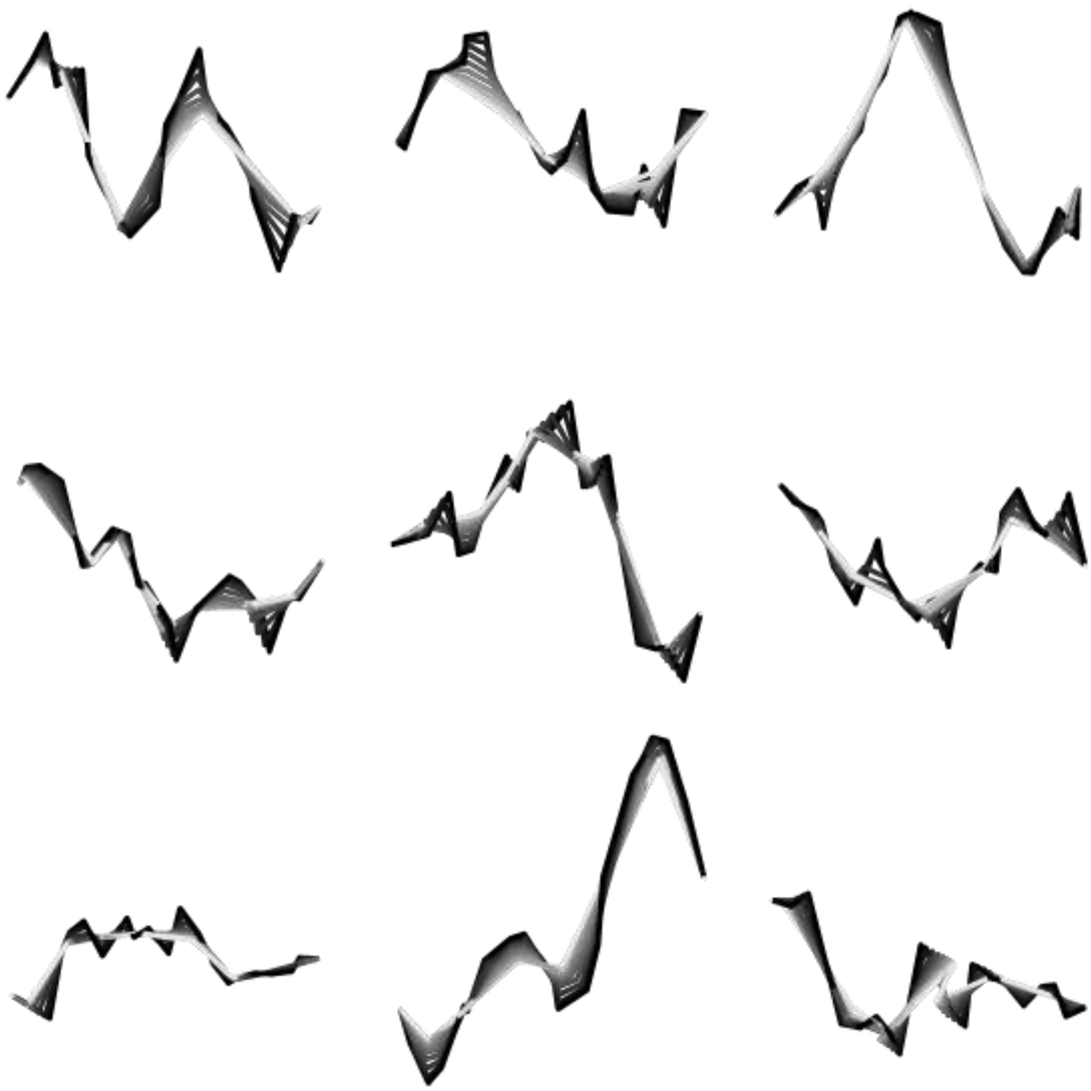

soma_tree adds a soma

tree = soma_tree (intree, mD, l, options)

Changes the diameter in tree ***intree*** in all locations smaller than path length *x*=***l***/2 [in µm] away from the root. Diameters become a sort of circular (cosine) soma shape of maximal diameter ***mD***:

$$D(x) = \frac{mD}{4}\left(\cos\left(\frac{2\pi x}{l}\right)+1\right)$$

If the original diameter at that location in the tree is larger, it remains unchanged!

Examples:
change the maximum diameter (***mD***); by default ***l*** is just 1.5x ***mD***

```
>> rtree = resample_tree (sample2_tree, 1);

>> soma_tree (rtree, mD)
```

resampling is better for a homogeneous result

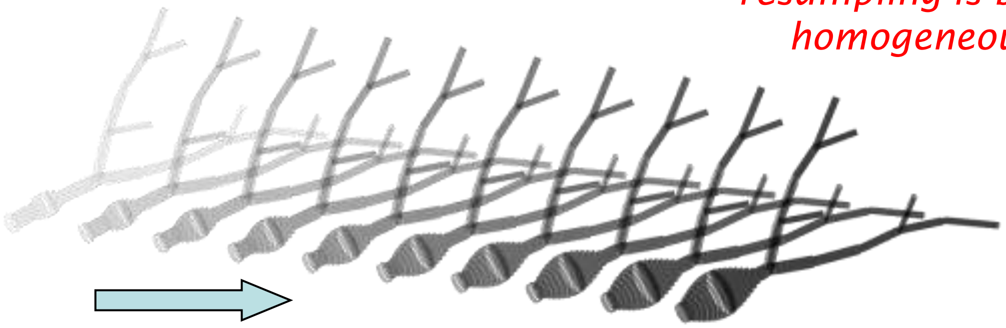

change the maximum path length value (***l***)

```
>> soma_tree (rtree, 20, l)
```

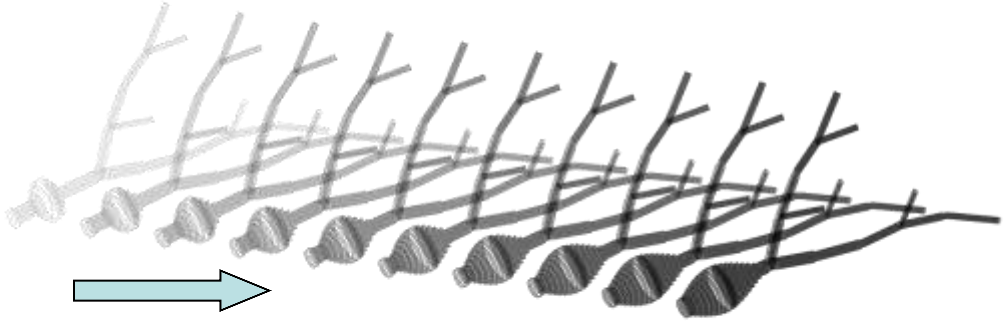

spines_tree add spines

```
tree = spines_tree (intree, XYZ, dneck, dhead, mlneck, stdlneck, ipart, options)
```

Attaches cylinders with diameter ***dhead*** to closest node on an existing tree ***intree***, introducing a neck with diameter ***dneck***. If XYZ coordinates ***XYZ*** are not defined, the spines are attached to a randomly picked node with distance ***mlneck***+-***stdlneck***. ***XYZ*** parameter becomes the number of spines (default: 100). If region with name "spines" exists then nodes are appended to that region otherwise new nodes are attributed to new region named "spines“. ***ipart*** is an optional index for attaching spines only to a sub-set of nodes.

resampling allows for more “attaching” points on the tree

Example:

```
>> rtree = resample_tree (sample2_tree, .2);

>> spines_tree (rtree, 200, [], [], [], [], find(rtree.D<3))
```

200 random spines

only attached to nodes where diameter is smaller than three

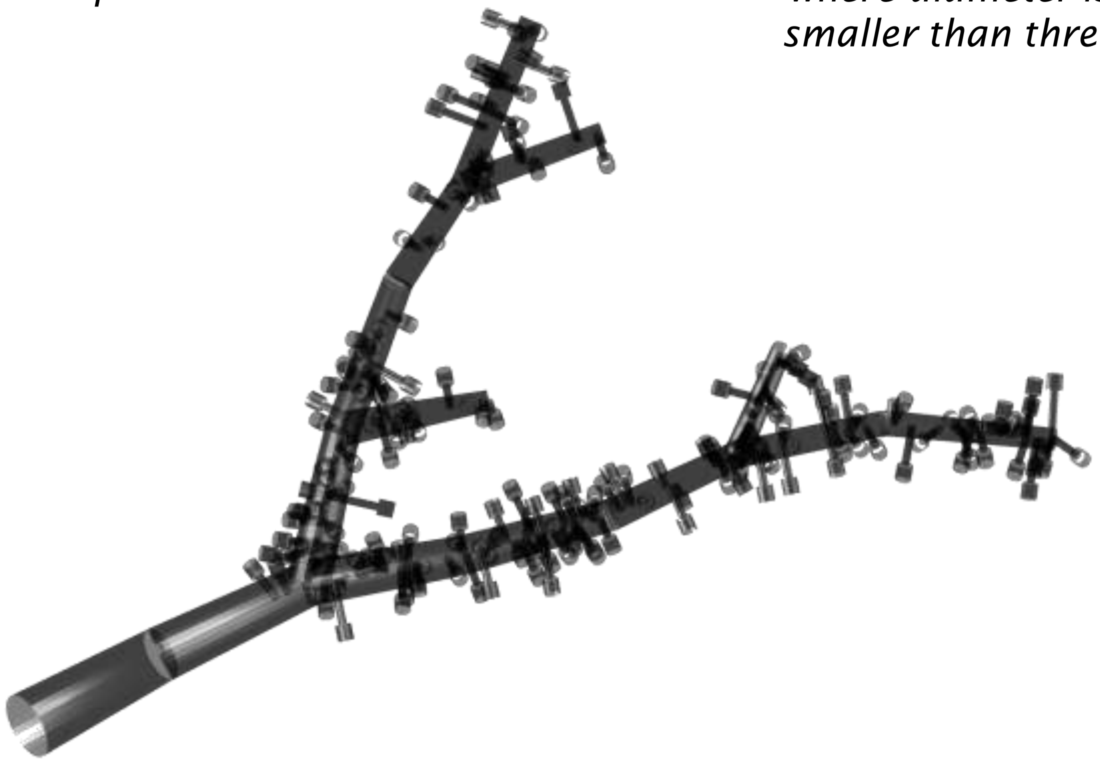

electrotonics

functions to calculate current flow in a tree

**elen_tree**	local electrotonic lengths
**gi_tree**	local axial conductances
**gm_tree**	local membrane conductances
**lambda_tree**	local length constants
**loop_tree**	conductance matrix including loops
**M_tree**	conductance matrix of tree equivalent circuit
**sse_tree**	steady-state electrotonic signature
**ssecat_tree**	sse signature including gap junctions
**syn_tree**	sse signature including synapses
**syncat_tree**	sse signature incl. electrical and input synapses

For these functions, the tree structure must contain passive electrotonic properties:

tree.Ri	axial resistance [in ohm cm]
tree.Gm	membrane conductance [in S/cm^2]
tree.Cm	membrane capacitance (unused)

elen_tree local electrotonic lengths

elen = elen_tree (intree, options)

Returns for tree ***intree***, *Nx1* vector ***elen*** the electrotonic length of all segments (length/lambda, see "lambda_tree").

Example:

```
>> elen_tree (sample2_tree)
```

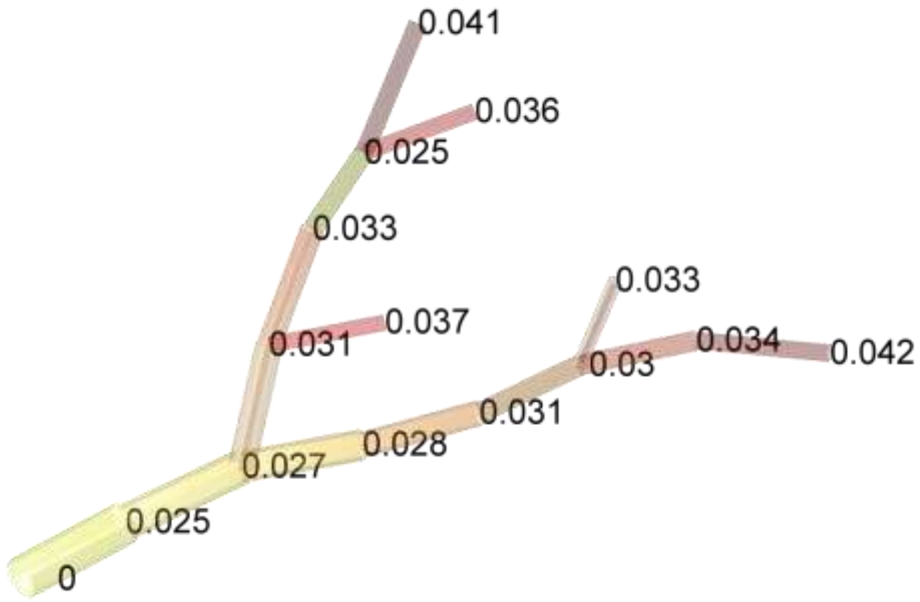

gi_tree local axial conductances

gi = gi_tree (intree, options)

Returns for tree ***intree***, *Nx1* vector ***gi*** the local axial conductances of all segments [in Siemens].

Example:

```
>> gi_tree (sample2_tree)
```

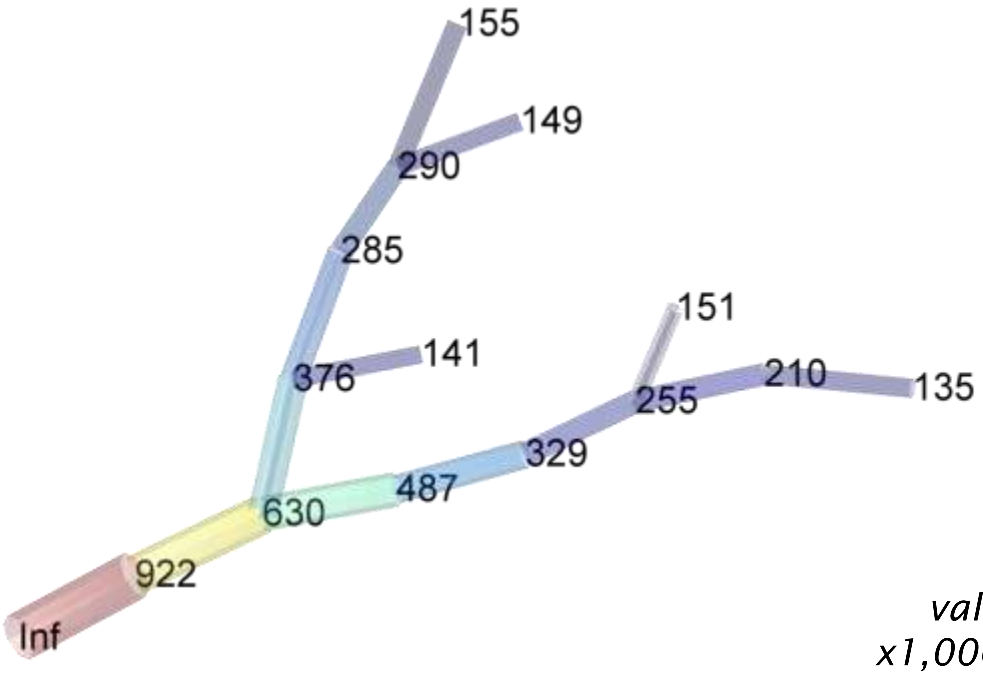

values are x1,000,000,000

gm_tree local membrane conductances

gm = gm_tree (intree, options)

Returns for tree ***intree*** , *Nx1* vector ***gm*** the local membrane conductance of all segments [in Siemens].

Example:

```
>> gm_tree (sample2_tree)
```

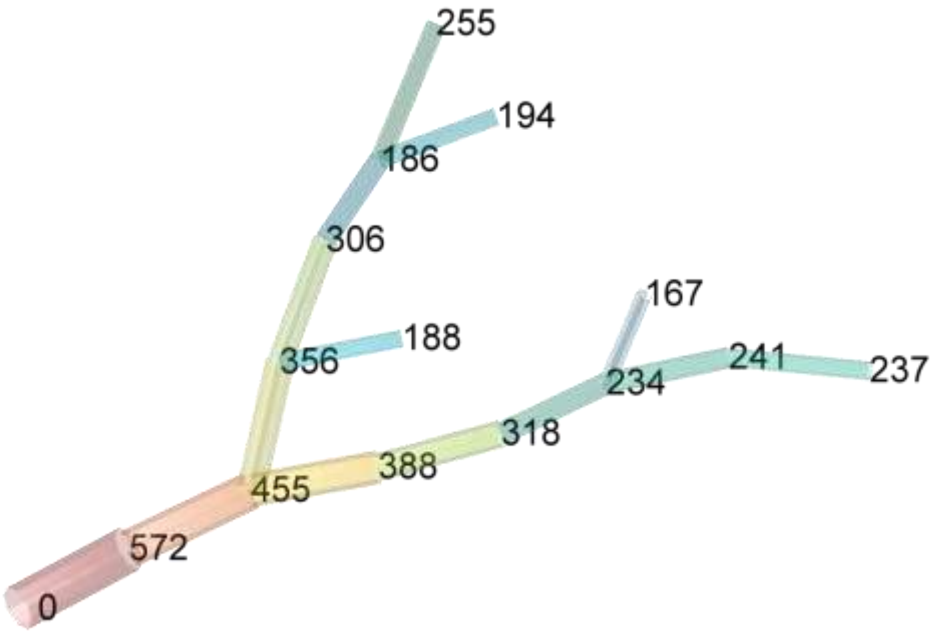

values are x1,000,000,000,000

lambda_tree local length constants

`lambda = lambda_tree (intree, options)`

Returns *Nx1* vector ***lambda*** the local length constant [in cm] of all segments in tree ***intree***.

Example:

`>> lambda_tree (sample2_tree)`

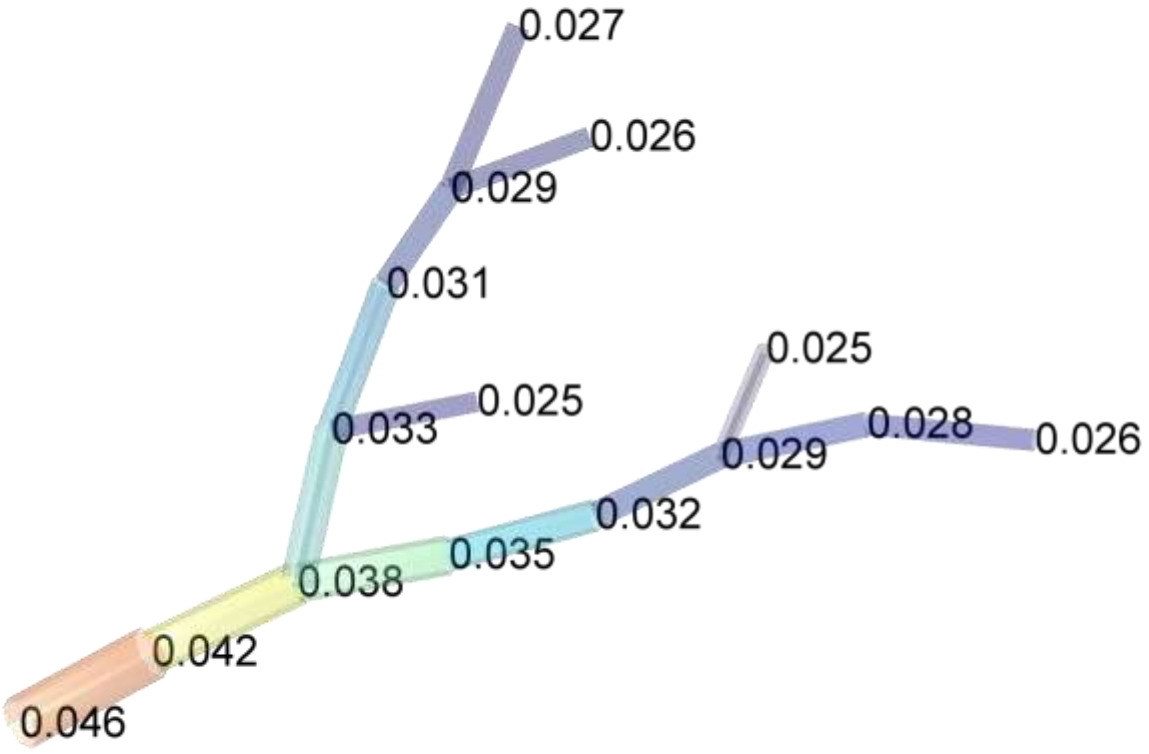

loop_tree conductance matrix incl. loops

M = loop_tree (intree, inodes1, inodes2, gelsyn, options)

Creates loops in the neuronal conductance matrix of tree ***intree***. Since conventional trees cannot be used (they are not supposed to have loops), a conductance matrix ***M*** is calculated directly extending on ***M*** from "M_tree". Set of *num* nodes ***inodes1*** are connected to the set of *num* nodes ***inodes2*** with gap junction conductances given by *numx1* vector ***gelsyn***.

Examples:
connect node #1 with node #13

```
>> M = loop_tree (sample2_tree, 1, 13, [],'-s')
```

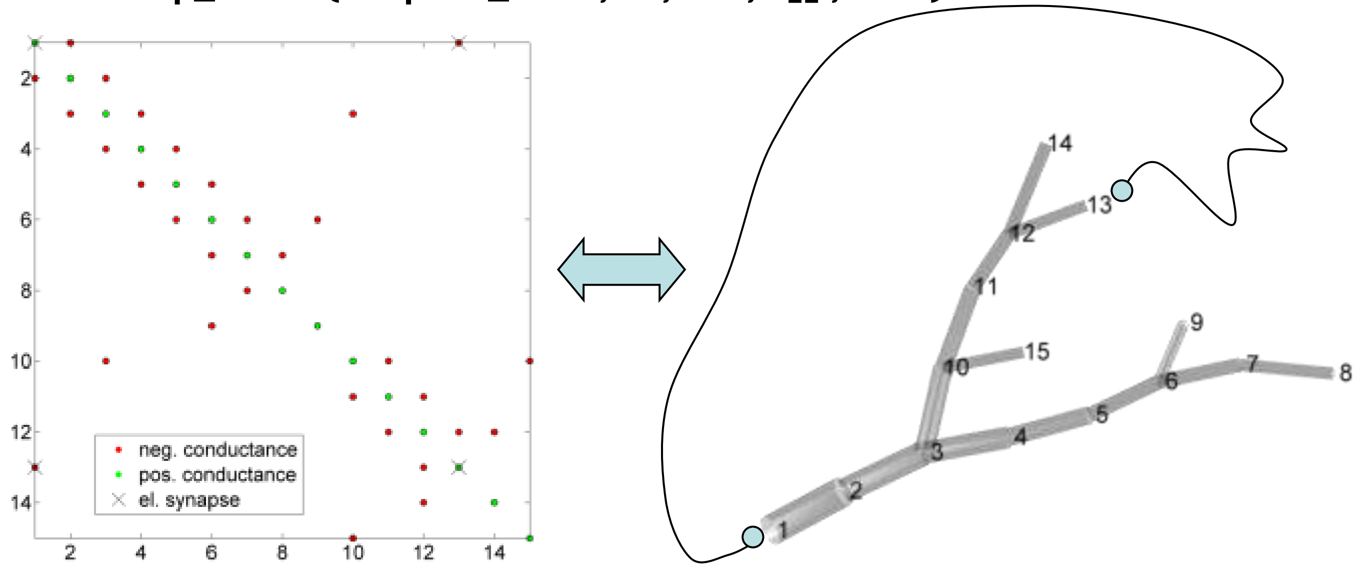

inverse of the loop conductance matrix gives the electrotonic signature (just as "sse_tree"):

```
>> inv (M)
```

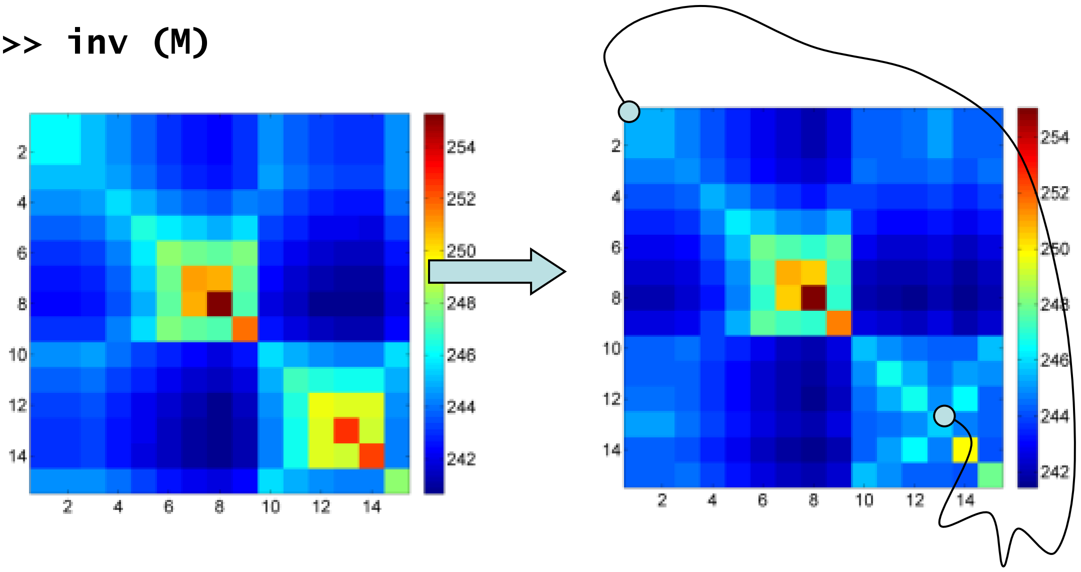

M_tree conductance matrix

```
M = M_tree (intree, options)
```

Calculates the matrix containing the conductances in the equivalent circuit of the neuron in the tree structure ***intree***. To be used in "sse_tree" and other electrotonic analysis of trees. See introduction section "electrotonic signature" for more details:

$$M = G_m D_S + G_a \{ \text{diag} [\text{sum} (A D_l + D_l A^T)] - (A D_l + D_l A^T) \}$$

G_m, G_a:	spec. membrane and axial conductances (inverse of R_i)
D_s, D_l:	diag. matrices with compartment surfaces and inverse volumes
A:	non-directed adjacency matrix (= $dA + dA'$)

Example:

```
>> M = M_tree (sample2_tree, '-s');
```

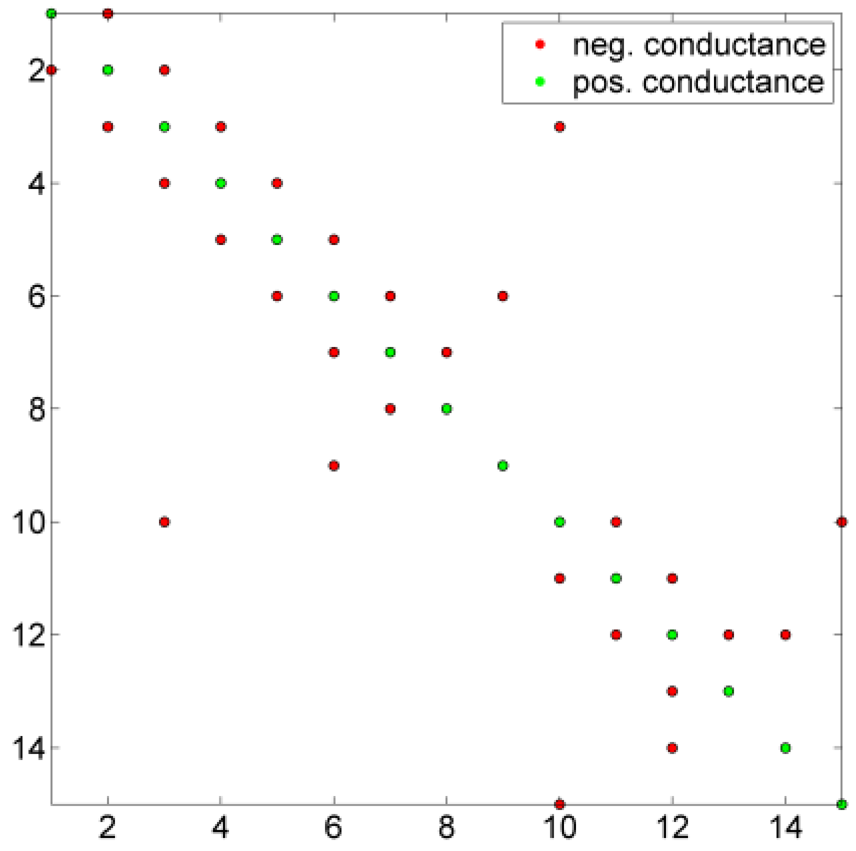

sse_tree electrotonic signature

sse = sse_tree (intree, I, options)

Calculates the steady state electrotonic (***sse***) matrix describing the electrotonic properties of the neuron in the tree structure ***intree***. Each column *j* is the potential distribution in all nodes during injection of current into node *j*. The diagonal contains therefore the local input resistances of each node. ***sse***, *NxN* matrix, is therefore symmetric. If input current ***I*** is not identity matrix then columns in ***sse*** correspond to potential distributions in separate experiments corresponding to the input current distribution in that column. Note that *NxN* ***sse*** is obtained by inverse matrix calculation and therefore goes very quickly but takes memory. In special cases it is advisable to split calls in several input matrices ***I***.

Example 1:
calculate the full *NxN* ***sse*** matrix

```
>> sse = sse_tree (sample2_tree)
```

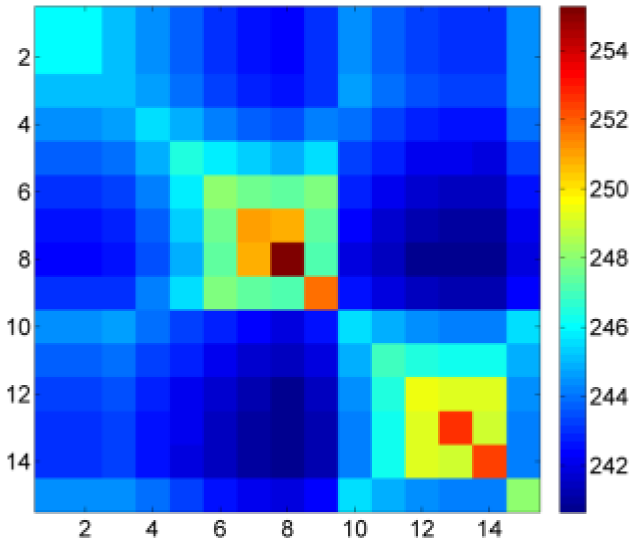

Example 2:
inject current only in node #13

```
>> sse = sse_tree (sample_tree, 13)
```

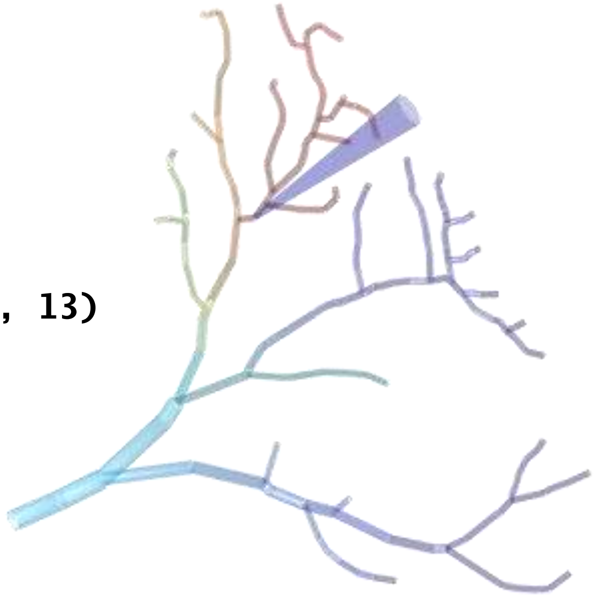

ssecat_tree sse signature incl. gap junctions

sse = ssecat_tree (intrees, inodes1, inodes2, gelsyn, I, options)

Concatenates trees in cell array of trees ***intrees*** with electrical synapses and calculates the steady state electrotonic matrix (***sse***, see “sse_tree”). Indices of connected nodes (***inodes1*** to ***inodes2***) accumulative along trees. ***gelsyn*** assigns conductance values to each gap junction. ***I*** is as in “sse_tree”.

Example:
we reproduce a simplified dendritic network similar as in (*Cuntz H, Haag J, Borst A 2003 PNAS 100(19):11082-5). Connect all nodes from one tree to another one and inject a current in the dendrite of one tree:*

```
>> tree = hsn_tree;

>> sse = ssecat_tree ({tree tree}, (1:1290)', (1291:2580)', .01, 15, 'none');
```

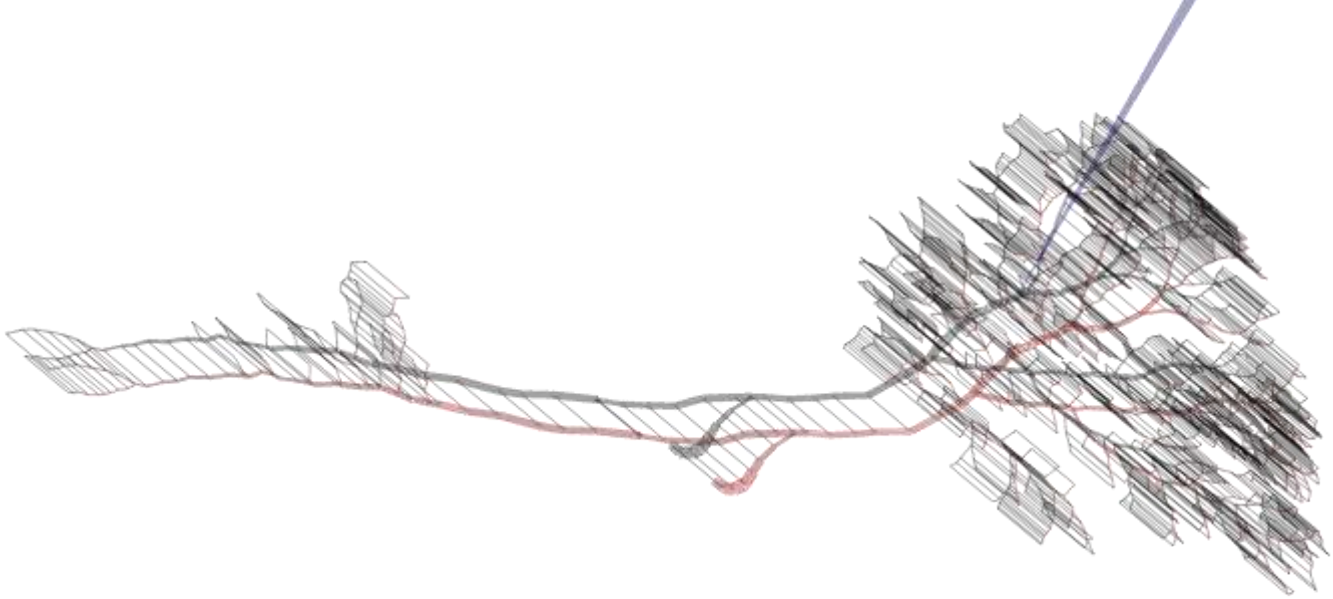

The resulting potential spread is smaller in the tree where the current was injected, than in the neighbour: the dendritic network leads to spatial blurring, as is used in some fly interneurons to process motion-based images:

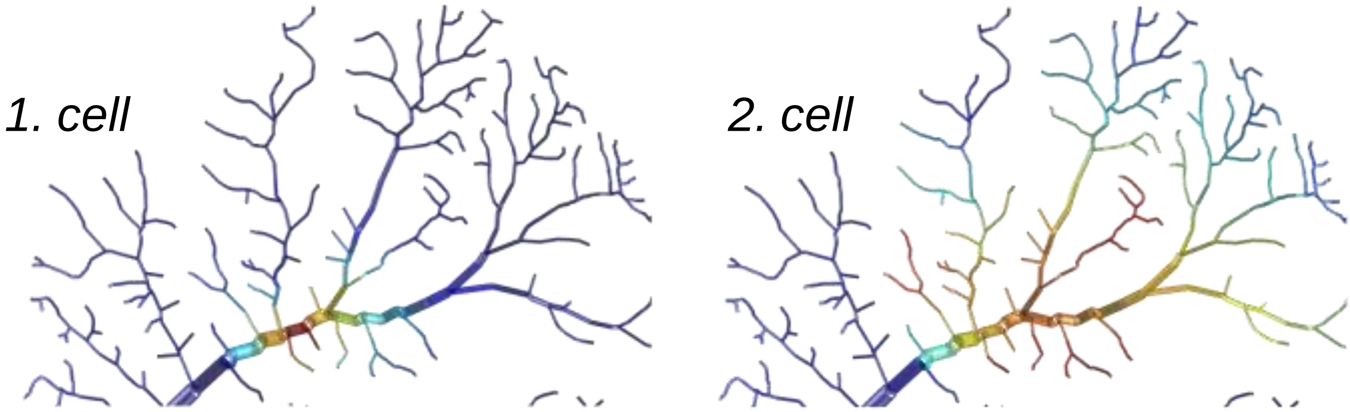

syn_tree synaptic electrotonic signature

syn = syn_tree (intree, ge, Ee, gi, Ei, I, options)

Calculates the steady state potentials matrix ***syn*** resulting from a current injection I (see “sse_tree”) and synaptic inputs defined by *Nx1* vectors ***ge*** and ***gi***, conductance values for each node (or alternatively: node location of unit conductances), and reversal potential values ***Ee*** and ***Ei*** into tree ***intree***.

Examples:
Impact of on-path location of inhibition, compare:

```
>> sse = syn_tree (sample_tree, 100, 95, [], [])
```

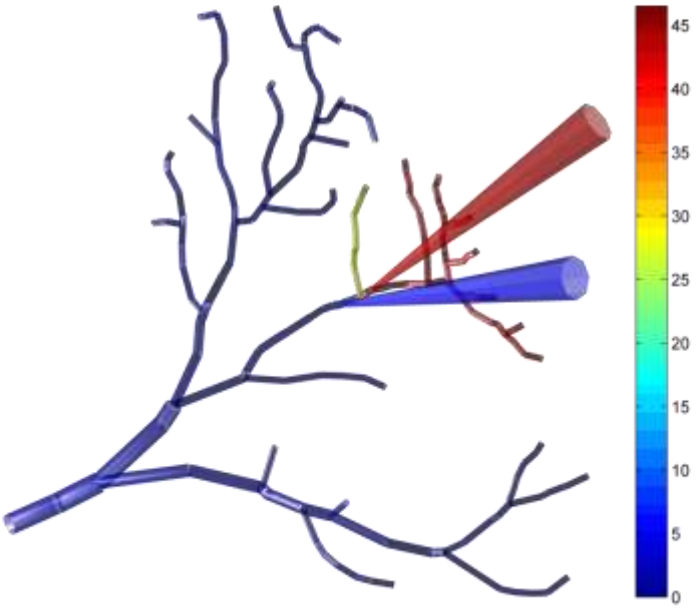

inhibition (blue) on the path between excitation (red) and root

```
>> sse = syn_tree (tree, 100, 105, [], [])
```

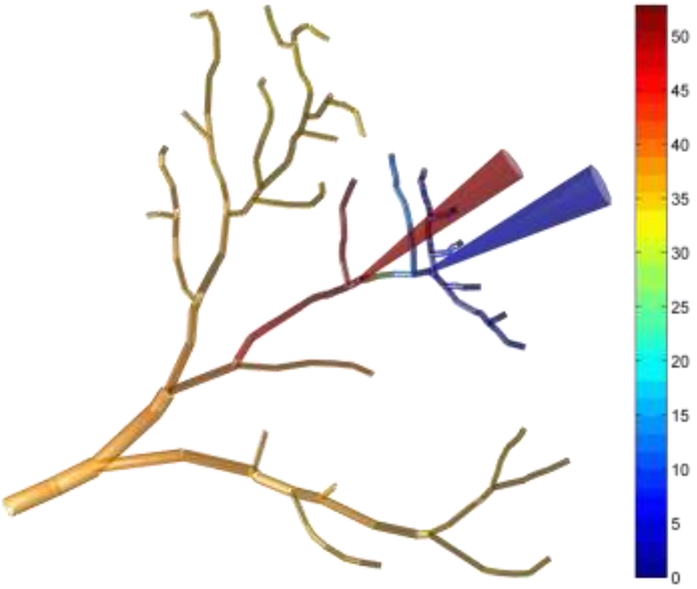

inhibition (blue) just on the wrong side

syncat_tree synaptic sse incl. gap junctions

```
syn = syncat_tree (intrees, inodes1, inodes2, gelsyn, ge, Ee, gi, Ei, ...
                   I, options)
```

Concatenates trees in cell array of trees ***intrees*** with electrical synapses and calculates the steady state electrotonic matrix just as does "ssecat_tree". Additionally, synaptic inputs (as in "syn_tree") can be defined by conductance values ***ge*** and ***gi*** and by reversal potentials ***Ee*** and ***Ei***.

Examples:

be creative...

IO functions

input and output functions

**load_tree**	loads a tree from swc/neu/TREES formats
**neurolucida_tree**	loads a tree from neurolucida ASCII format
**neuron_tree**	export tree as NEURON file
**pov_tree**	POV-Ray rendering
**save_tree**	save tree or many trees in TREES format
**swc_tree**	export tree as SWC file
**ver_tree**	verifies integrity of a tree
**x3d_tree**	export tree as X3D format
**neu_tree**	export tree in NEURON to read in TREES

load_tree loads a tree from swc/neu/mtr formats

[tree, name, path] = load_tree (name, options)

Loads the metrics and the corresponding directed adjacency matrix of tree from file ***name*** to create the tree structure. Input files can be in TREES internal .mtr format (which is just a matlab workspace file), in .swc format (see “swc_tree”) or in an export format .neu for trees created in the software NEURON package (http://www.neuron.yale.edu/neuron/) with the function “neu_tree” provided in the TREES toolbox. Make sure to realize that most imported trees are originally encoded as connected frusta instead of cylinders whereas the TREES toolbox assumes that they are cylinders. This can be changed by adding the field “frustum=1” to the tree structure.

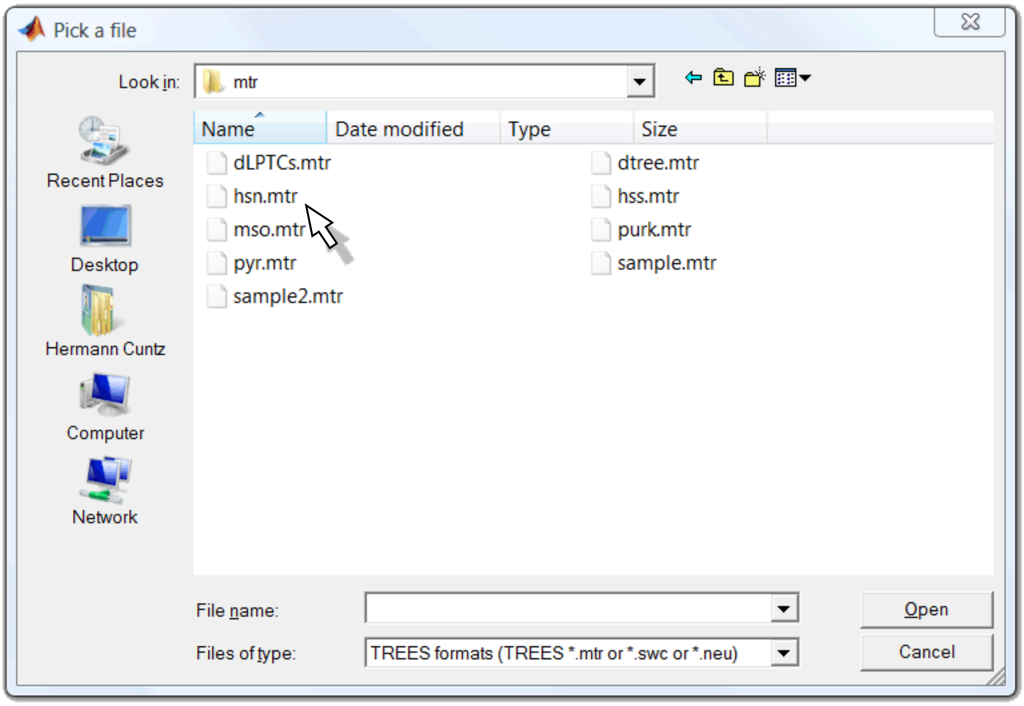

Examples:

```
>> load_tree

trees{1}

ans =

        dA: [15x15 double]
         X: [15x1 double]
         Y: [15x1 double]
         Z: [15x1 double]
         R: [15x1 double]
         D: [15x1 double]
    rnames: {'1'  'dendrite'}
        Ri: 100
        Gm: 5.0000e-004
        Cm: 1
      name: 'tree1'
```

neurolucida_tree loads a tree ASCII format

[tree, coords, contours, name, path] = neurolucida_tree (name, options)

Loads the metrics and the corresponding directed adjacency matrix to create a tree directly from an ASCII neurolucida description file called ***name***. NOTE! For example to infer the cylinder-representation of the soma we chose arbitrary algorithms similar but not equal to the NEURON neurolucida import. Sub-trees are attributed to somata by who-is-closest. This function can be much further optimized or just rewritten. The TREES toolbox function however has additional features to the NEURON neurolucida import. For example spines are imported (as cylinders with region named "spines"). Furthermore, imported markers can be added as spines via "spines_tree".

Examples:

```
>> neurolucida_tree
```

neuron_tree export tree as NEURON file

[name path] = neuron_tree (intree, name, res, options)

Saves a complete tree ***intree*** into the file called ***name*** in the section based NEURON (http://www.neuron.yale.edu/neuron/) .hoc format. ***res*** determines the resolution at which NEURON samples a segment (*nseg*). Alternatively, the tree can also be stored as .nrn file in which each segment from the tree graph becomes an independent section in NEURON. Option '-e' incorporates electrotonic properties if existent, '->' starts NEURON immediately if it is installed and the TREES toolbox runs in windows.

Examples:
Current injection in section dendrite[5] and spatial potential distribution.

>> neuron_tree (sample2_tree, [], [], '-s -e ->')

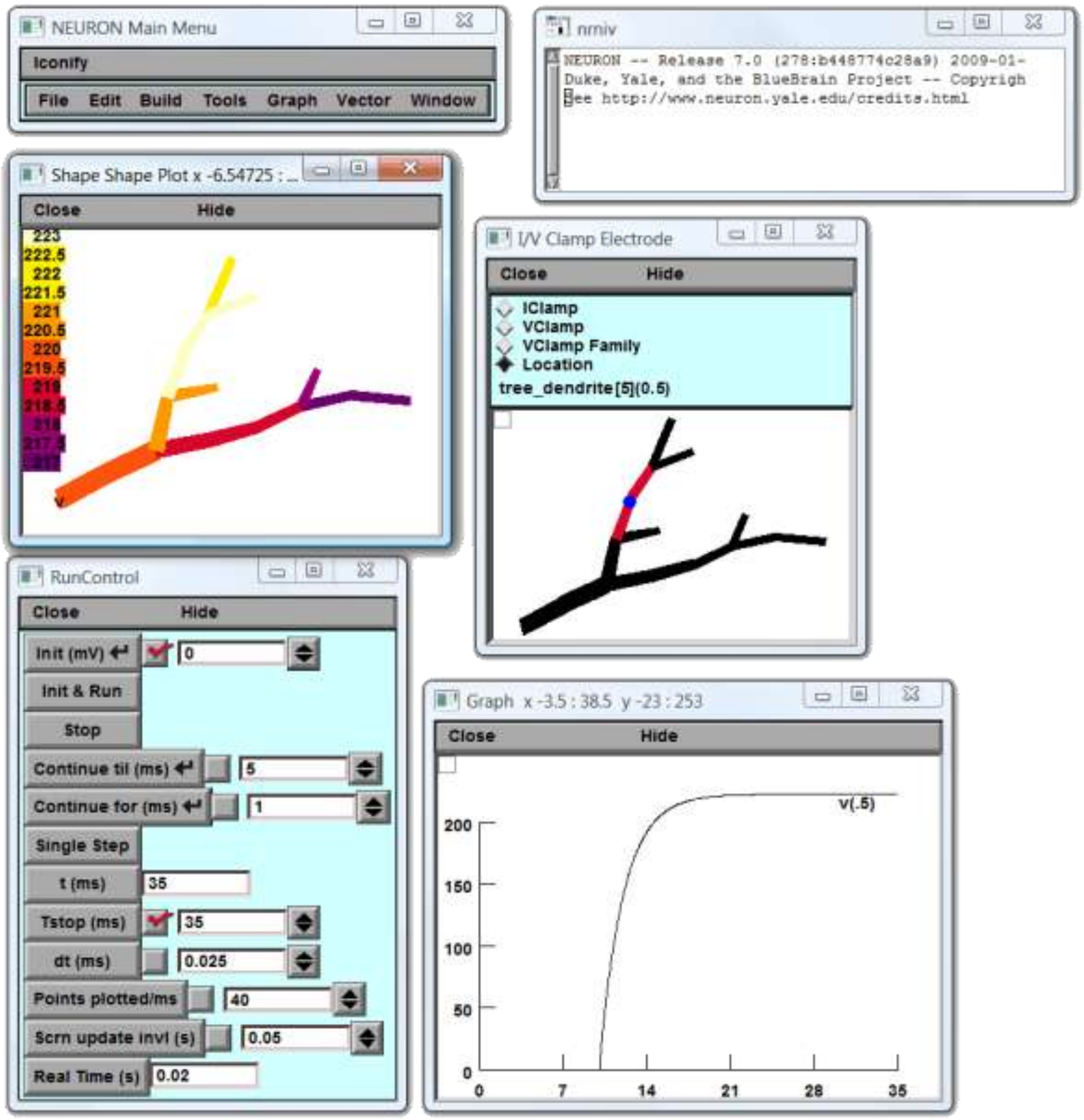

pov_tree POV-Ray rendering

[name path] = pov_tree (intree, name, v, options)

Writes POV-ray (http://www.povray.org/) files using the anatomy-data contained in ***intree***. ***intree*** can be a single tree structure or a cell array of trees or just *XYZD* coordinates of points (plotted as spheres). With ***v***, an *Nx1* vector or cell array of vectors (corresponding to ***intree***), values for each node can be mapped to the colours of the segments. options involve different styles and for example option '-c' for brainbow random colours and '-v' option which conserves the viewpoint of the active Matlab figure. '->' starts POV-Ray immediately if it is installed and the TREES toolbox runs in windows.

```
>> pov_tree (sample2_tree, [], [], '-b -s1 -w ->')
```

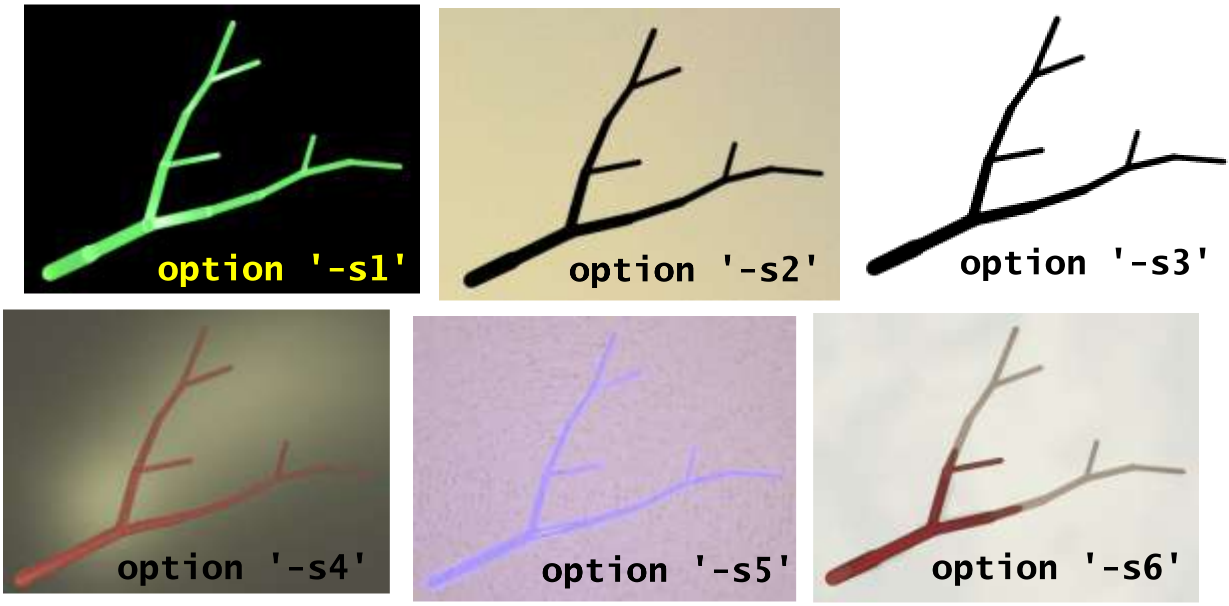

mapping of colors works as in „plot_tree“:

```
>> pov_tree (sample2_tree, [],
eucl_tree (sample2_tree),
 '-b -s1 -w ->')
```

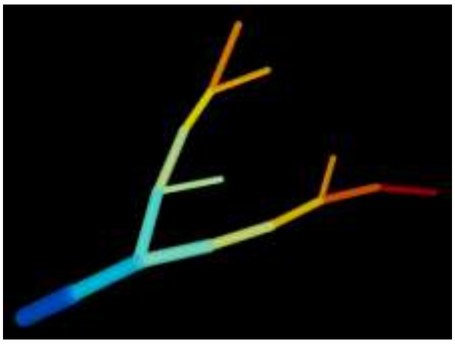

brainbow colour mapping on a group of trees:

```
>> dLPTCs = load_tree ('dLPTCs.mtr')

>> pov_tree (dLPTCs,[],[],'-b -c -s1
->')
```

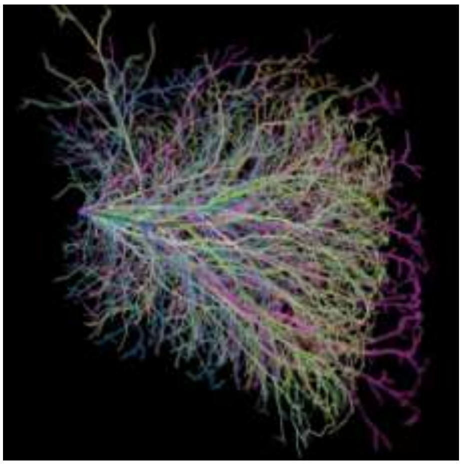

save_tree save tree/trees into a file

[name path] = save_tree (intree, name)

Saves tree ***intree*** into a TREES internal .mtr format using a Matlab type workspace file. ***intree*** can be a structured tree or a cell array of structured tree or a cell array of cell array of structured trees (2-depth). This type of file can be read directly by the GUI (see below) and the 2-depth in that case allows to arrange in groups of trees.

Examples:

```
>> save_tree (sample2_tree)
```

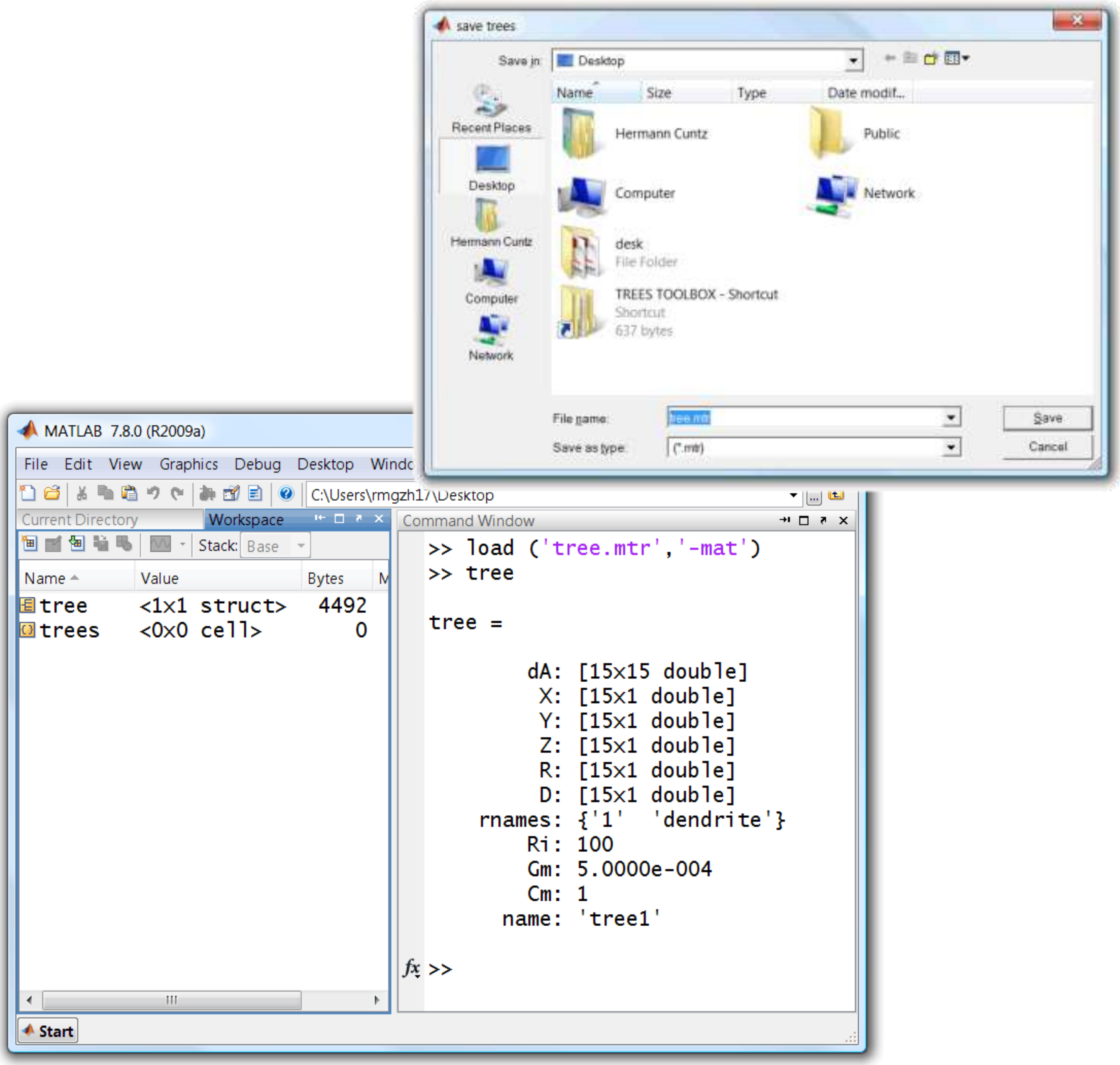

swc_tree export to ".swc" format

`[name path] = swc_tree (intree, name)`

Exports tree ***intree*** to the .swc format, a matrix with 7 columns:

[*inode R X Y Z D/2 idpar*]

node index *inode* is usually from *1..N* and *idpar* is the direct parent index. The root has an *idpar* of -1. Fills in region index *R* if *R* is missing.

Examples:

```
>> swc_tree (sample2_tree)
```

produces:

```
# TREES toolbox tree - tree
# written by an automatic procedure "swc_tree" part of
# the TREES package in MATLAB
# copyright 2009 Hermann Cuntz
#
# inode R X Y Z D/2 idpar
1  2   0.00000000   0.00000000   0.00000000   2.12528117 -1
2  2   8.29304961   4.44755127  -4.46045613   1.74844749  1
3  2  17.40876415   8.80206655  -3.39415938   1.42701764  2
4  2  26.90050540  10.54155965  -0.95626440   1.24193528  3
5  2  36.06519913  13.03275911   1.94061621   1.02025571  4
6  2  43.93899105  16.79337615   3.22598031   0.84536559  5
7  2  53.10549509  18.73244431   5.26125456   0.80092718  6
8  2  63.62347087  17.73353041   8.25362875   0.68685660  7
9  2  46.75945383  23.43321561   7.46333851   0.63360263  6
10 2  19.55373994  18.37988646  -0.46949875   1.10702919  3
11 2  22.96179913  27.48763086   2.41729942   0.96010238 10
12 2  27.01621437  33.48111427   2.79183599   0.81713800 11
13 2  35.71264128  36.71289453   2.66823593   0.66393903 12
14 1  31.24690854  43.64653220   2.81017550   0.73654865 12
15 1  28.66148433  20.14267570  -0.28834723   0.64516327 10
```

ver_tree verifies the integrity of a tree

ver_tree (intree)

Verifies the integrity of a tree ***intree*** and creates warnings that precede common errors. Is called by basically every single TREES package function. Could therefore be used for something else. This is rather an internal function.

Examples:

```
>> ver_tree (sample2_tree)
```

no output, tree is ok...

x3d_tree exports tree as .x3d

[name path] = x3d_tree (intree, name, color, DD, ipart, options)

Exports a tree ***intree*** as a set of cylinders in the .x3d html format. A viewer is necessary to use these files. Blender (http://www.blender.org/) and BS Contact (http://www.bitmanagement.com/) can for example load .x3d files. As opposed to POV-Ray (see "pov_tree", http://www.povray.org/), these programs can only deal with polygons. As such, the output trees are sets of polygons. Object is offset by XYZ 3-tupel ***DD*** and coloured with RGB 3-tupel ***color*** (or *Nx1* vector, attributing one value per node). ***ipart***, a set of indices into ***intree***, allows to use only a subset of nodes, for example only a sub-tree. If a viewer is installed and TREES runs on windows Matlab can call the viewer directly with the option '->'.

Examples:

```
>> x3d_tree (sample_tree, [], [1 0 0],[],[],'-o ->')
```

red (arrow to [1 0 0])

add spheres at node locations (arrow to -o)

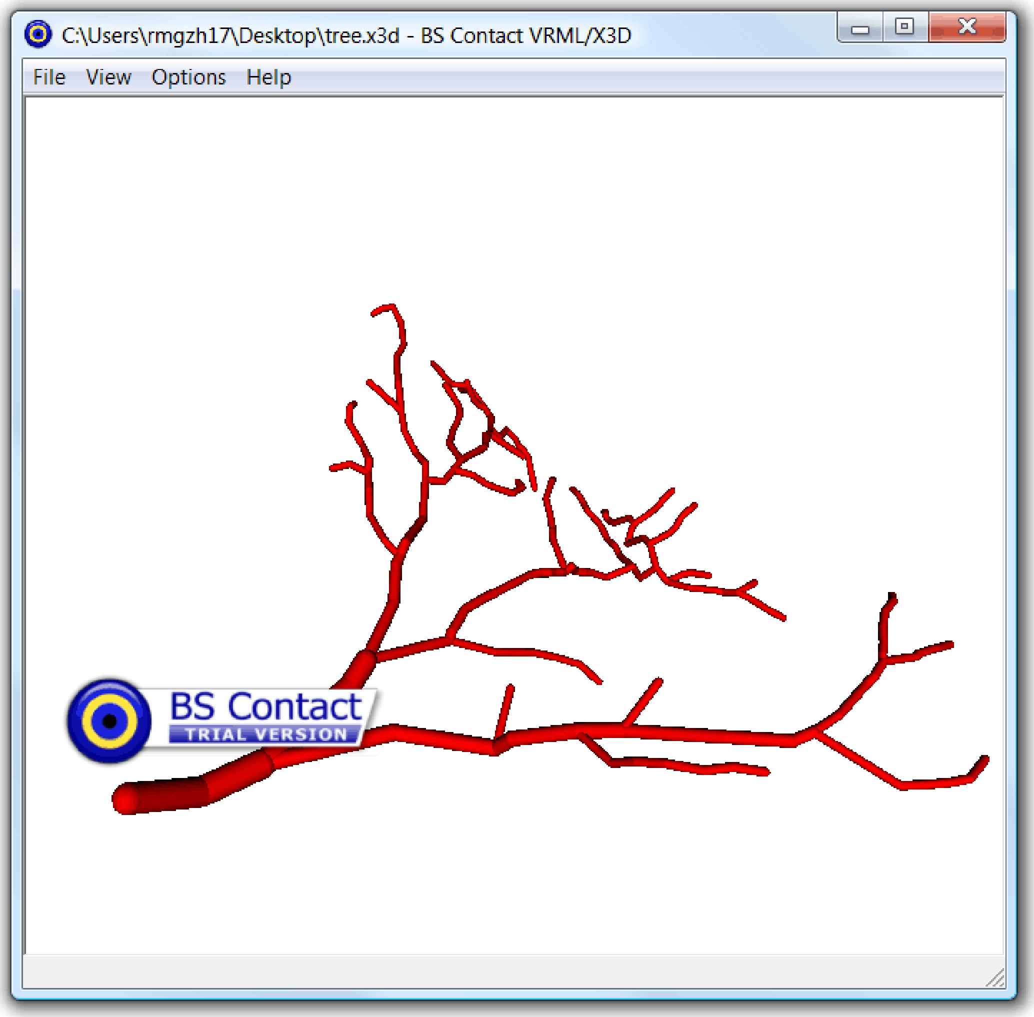

written by Friedrich Forstner 2008

neu_tree export from NEURON to TREES

neu_tree (name)

This is a NEURON .hoc file which exports a tree into a .neu format which the TREES toolbox can read using “load_tree”. Works only for basic trees and sometimes scrambles the graph (use “cgui_tree” for editing). Make sure to realize that most imported trees are originally encoded as connected frusta instead of cylinders whereas the TREES toolbox assumes that they are cylinders. This can be changed by adding the field “frustum=1” to the tree structure.

code in NEURON hoc
After having loaded one single cell in the NEURON environment (make sure that the file neu_tree.hoc is in the current directory).
Examples:

```
>> load_file("neu_tree.hoc")

>> neu_tree(“tree.neu”)
```

„load_tree“ in the Matlab environment is then able to load the .neu file.

scheme

non-TREES related dependencies

deg2rad (x) — **transposes from degrees to radians**

result = mod((x/360)*2*pi,2*pi)

eucdist (X1,X2,Y1,Y2) — **2D Euclidean distances btw. 2 sets of points**

Calculates a distance matrix M between two sets of points described by their x and y coordinates.

gauss (x,mu,sigma) — **gauss function output**

(1/(sigma*(sqrt(2*pi))))*exp(-((x-mu).^2)./(2*sigma*sigma))

rad2deg (x) — **transposes from radians to degrees**

result = mod((x/(2*pi))*360,360)

rotation_matrix (degx,degy,degz,hand) — **calculates rotation matrix for given angles**

treats the different rotations in order x then y then z. In other words it's the rotation_matrix R = Rz*Ry*Rx. Degrees of rotation are given in radians.

roundshow — **a 3D round show of a plot**

a 3D round show, simply changes the view in regular intervals.

scalebar (unit,pos) — **add a scalebar to a plot**

shine — **add some effects on current axis**

polishes the graphical output. By default simply adds a camera light (therefore the name "shine"), which typically sets the figure renderer to opengl as a side effect.

tprint — **simplified printing**

prints the current figure to a file.

gifmaker — **make a movie with transparent background**

appends frames one by one to a movie.

stacks

functions to deal with image stacks

**fitD_stack**	get cylinder diameter values from stack
**imload_stack**	load single image into a 3D matrix
**load_stack**	load TREES .stk file into stack structure
**loaddir_stack**	load image sequence into stack structure
**loadtifs_stack**	load multi-image .tif into stack structure
**save_stack**	save TREES .stk file
**show_stack**	show maximum intensity projections
**skel_stack**	skeletonize image stack

a data stack is a structure as follows:

**stack.M**	*cell-array of 3D-matrices*	n tiled image stacks containing fluorescent image
**stack.sM**	*cell-array of strings, 1xn*	names of individual stacks
**stack.coord**	*matrix nx3*	x,y,z coordinates of starting points of each
**stack.voxel**	*vector 1x3*	xyz size of a voxel

fitD_stack get cylinder diameter values

D = fitD_stack (intree, stack, maxR, options)

Tries to derive diameter values for tree ***intree*** based on the underlying image stack ***stack***. ***maxR*** determines a threshold maximum radius for a segment in the tree (should be much larger than the maximum radius, but of course not too large...).

Examples:
Here applied on a sample tree reconstructed from a sample stack:

```
>> D = fitD_stack (tree, stack, 50)
```

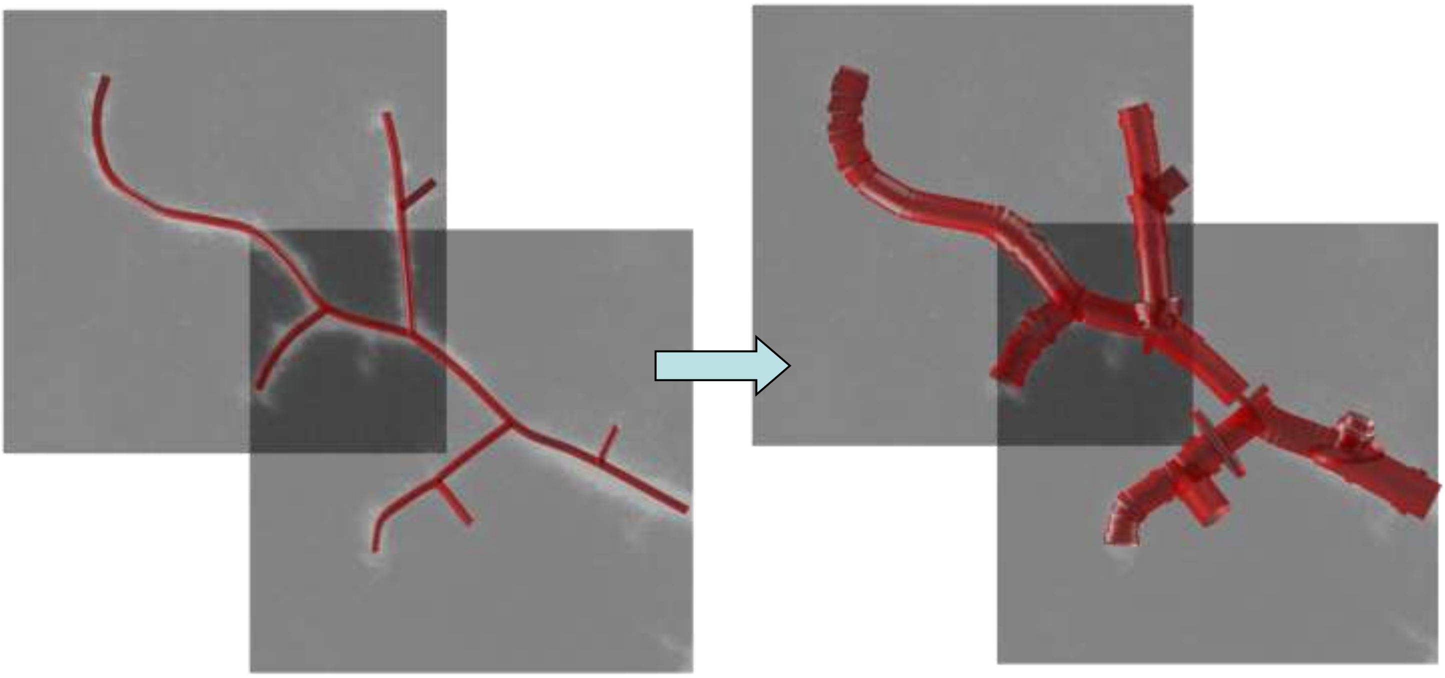

written by Friedrich Forstner 2008

imload_stack load single image

```
[stack name path] = imload_stack (name, options)
```

Loads a single image from file with filename ***name*** into stack ***stack***. If ***name*** is omitted a user interface for file selection is opened.

Examples:

```
>> imload_stack ([], '-s');
```

load_stack load TREES .stk file

```
[stack name path] = load_stack (name, options)
```

Loads a stack structure ***stack*** from a file with filename ***name***. If ***name*** is omitted a user interface for file selection is opened.

Examples:

```
>> stack = load_stack
```

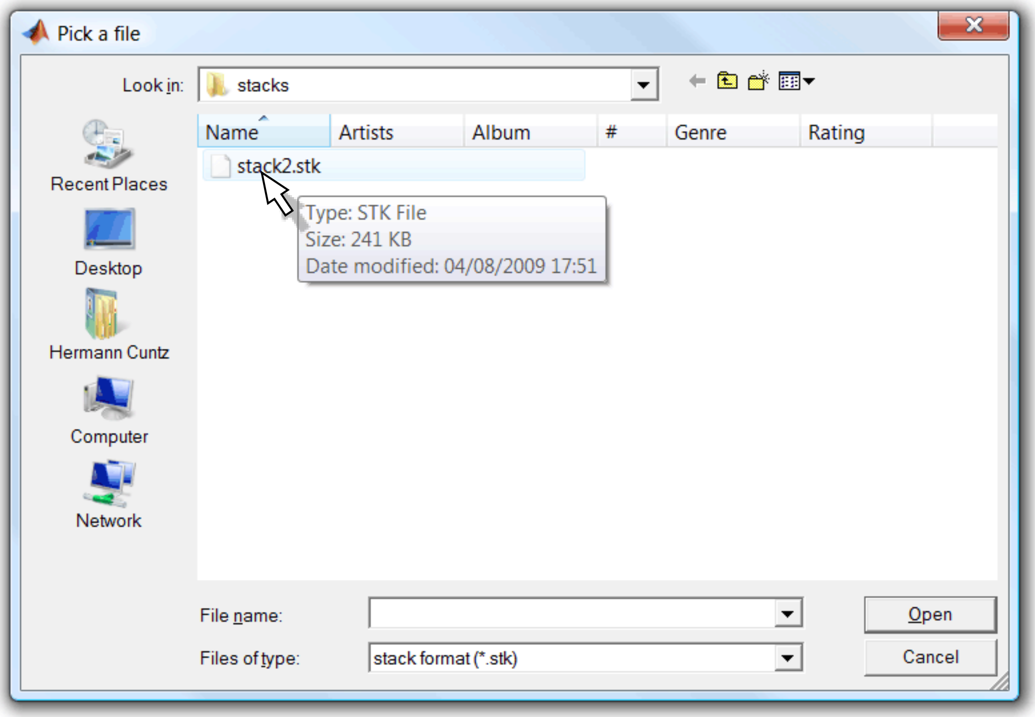


```
stack =

       M: {[100x100x19 uint8]  [100x100x19 uint8]}
      sM: {'HSN_2006-09-28-1'}
   coord: [2x3 double]
   voxel: [1 1 1]
```

loaddir_stack load image sequence from folder

```
[stack path] = loaddir_stack (path, options)
```

Loads all images from a directory defined by ***path*** to an image stack ***stack***. Images must have the same size but there is no error handling.

Examples:

```
>> loaddir_stack
```

loadtifs_stack load multi-image .tif

```
[stack name path] = loadtifs_stack (name, options)
```

Loads an image stack from a .tif file with filename ***name*** into a stack structure ***stack***.

Examples:

```
>> loadtifs_stack
```

save_stack save image stack into file

name = save_stack (stack, name, options)

Save images from a stack ***stack*** into a matlab type file of name ***name*** with extension .stk.

Examples:

```
>> save_stack
```

show_stack

show stack max intensity projections

```
HP = show_stack (stack, options)
```

Show the maximum intensity projection of a stack ***stack*** on a 3D patch.

Examples:

```
>> stack = load_stack ('sample.stk'); show_stack (stack)
```

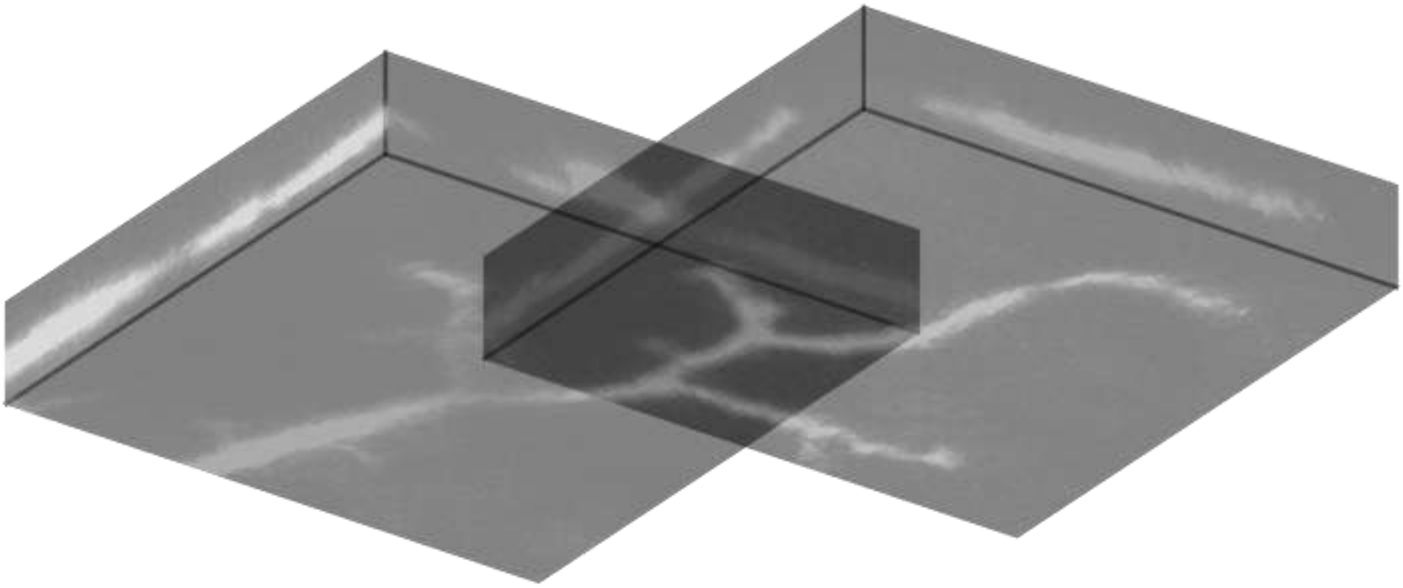

skel_stack skeletonize a 3D matrix

```
[i1 i2 i3] = skel_stack (iM, thr, options)
```

Extracts carrier points from a brightness level containing matrix ***iM***. ***thr*** sets a threshold to binarize ***iM***. ***i1***, ***i2*** and ***i3*** output the *Y*, *X* and *Z* values respectively. See the algorithm described by Palagyi and Kuba. Very nice piece of code, hopefully correctly interpreted from their paper. It involves numerous permutation of indices and logical operators on a 26-neighbourhood.

Examples:
skeletonize first matrix in stack:

```
>> [X Y Z] = skel_stack (stack.M{1}, 100);

>> hold on; show_stack (stack); plot3 (Y, X, Z, 'r.');
```

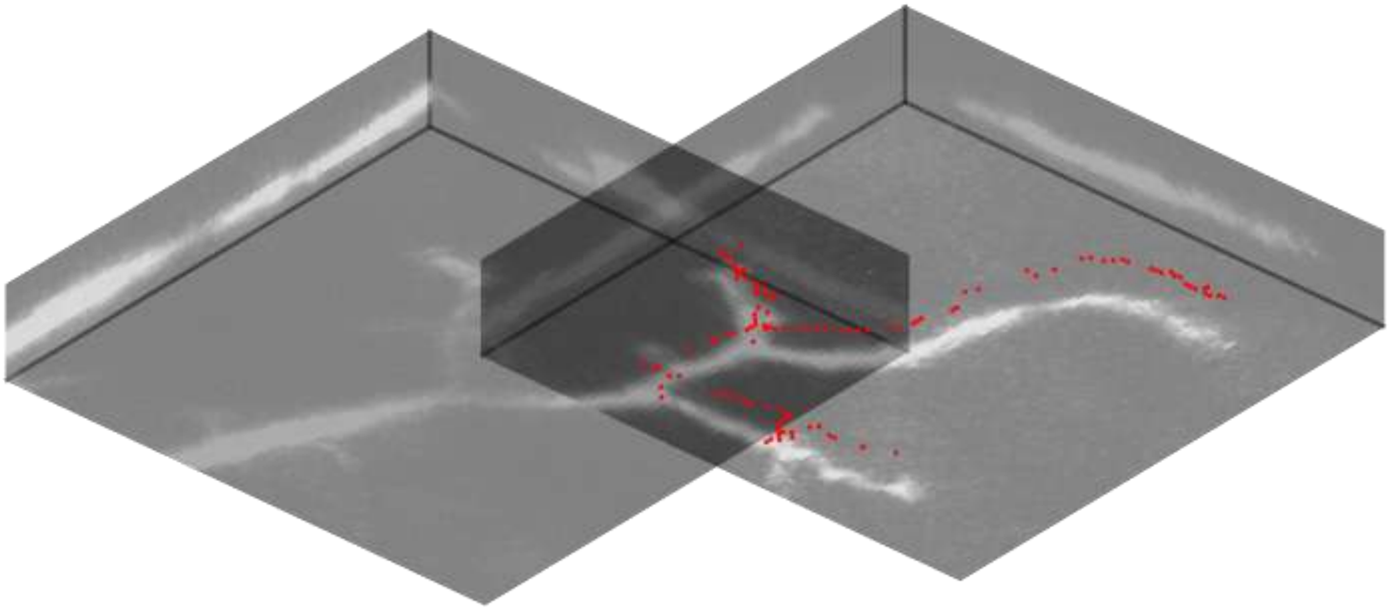

sample

sample data structures to test TREES toolbox

dLPTCs.mtr set of flattened dendritic trees of Lobula Plate Tangential Cells (LPTCs) of the fly

from Cuntz, Forstner, Haag, Borst 2008, PLoS Comp Biol 4: e1000251.

hsn.mtr one sample LPTC of the fly (HSN cell)

from Cuntz, Forstner, Haag, Borst 2008, PLoS Comp Biol 4: e1000251.

hss.mtr one sample LPTC of the fly (HSS cell)

from Borst and Haag 1996, J Comput Neurosci 3(4):313-36.

sample.mtr large sample sub-tree of an LPTC

sample2.mtr small arbitrary sub-tree

sample.stk small arbitrary stack

25HSS.swc SWC file of HSS cell above

from Borst and Haag 1996, J Comput Neurosci 3(4):313-36.

twop9purks.asc two Purkinje cells in neurolucida format

From Watt , Cuntz, Mori, Nusser, Sjöström, Häusser 2009, Nat Neurosci 12: 463-473.

sample.tw1 sample GUI workspace corresponding to the reconstruction of sample.stk

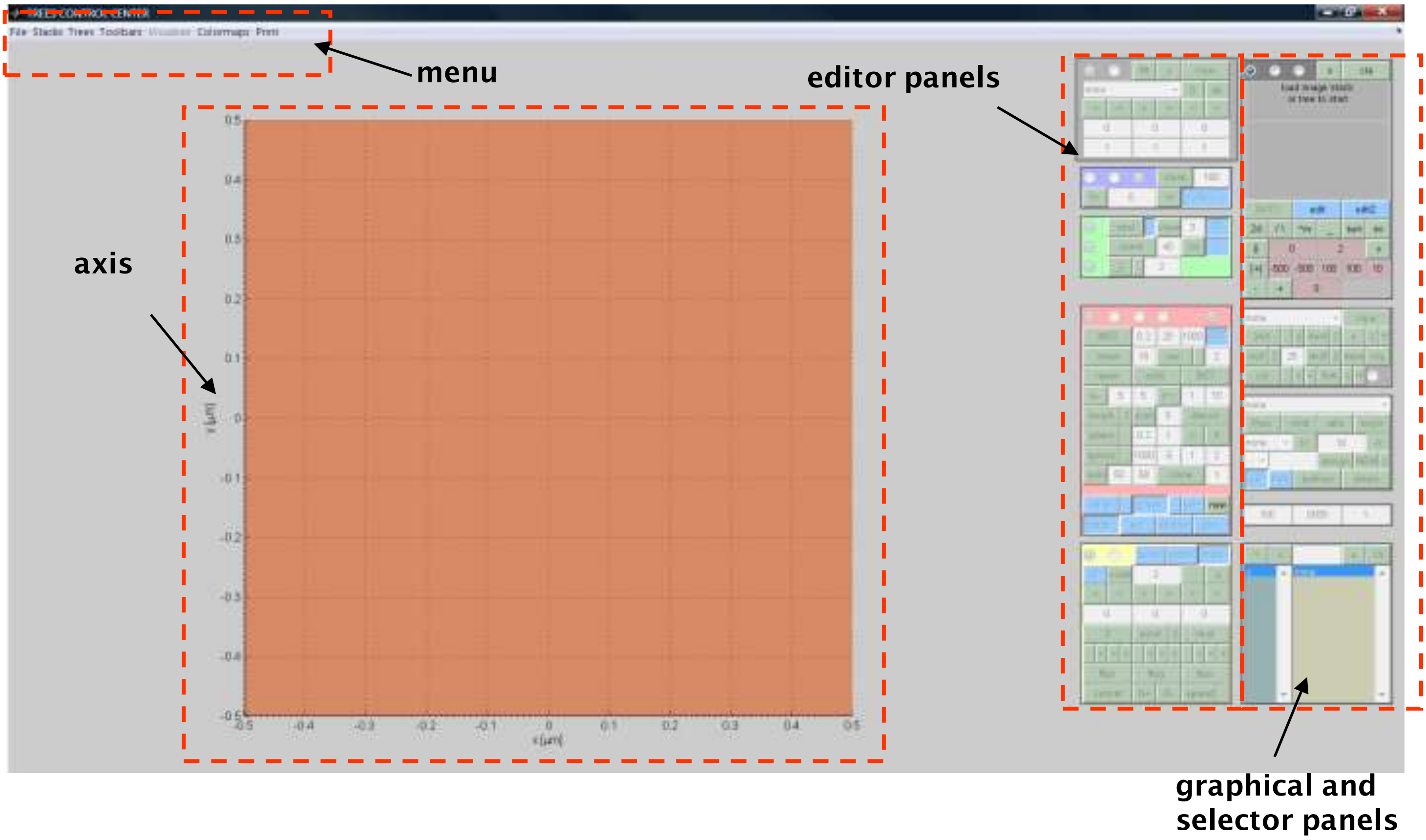

Calling:

```
>> cgui_tree
```

opens up a user interface window. With it the user can browse through directories of trees, edit them, explore their properties. The individual buttons typically link to one function of the TREES toolbox. The GUI is therefore practical to explore the possibilities of the toolbox before starting a research project with the more flexible command line interaction. Aside of that, the user interface allows the automated reconstruction of neuronal branching structures directly from image stacks. The following passages will attempt to familiarize the user with the axis, the multiple panels, the menu etc.. of the GUI.

All the GUI is divided into three parts, one common axis, a menu and a number of control panels. The control panels on the right are there to control the axis, the graphical output and to browse through trees or individual nodes or properties of a tree. The control panels to the left are associated with the process of reconstruction, artificial generation and editing of tree structures. To each of the panels on the left special “edit” modes are associated.

the vis_ panel: control the common axis

The vis_ panel (visualization) controls the one common axis. To each control panel a prefix is attributed (here „vis_“) and all computer code related to this control panel uses this prefix in the core program of the GUI, „cgui_tree“.

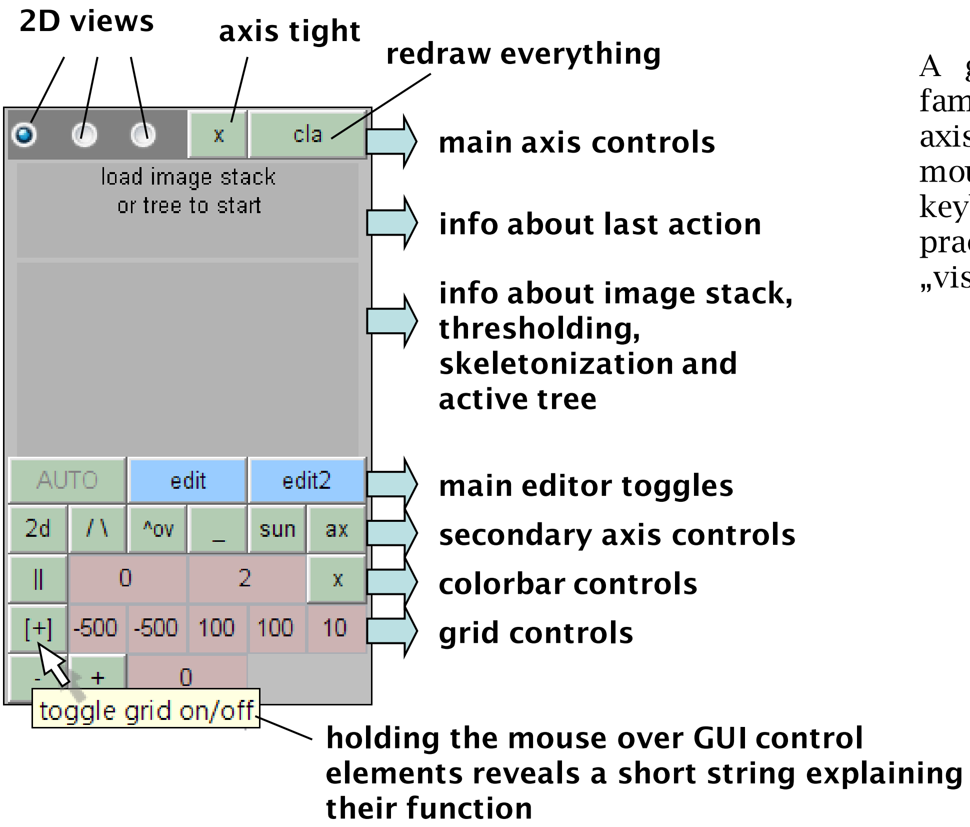

A good way to start is to get familiar with the controls of the axis by keeping one hand on the mouse and one hand on the keyboard. It is important to practice the axis control using the „vis_“ panel.

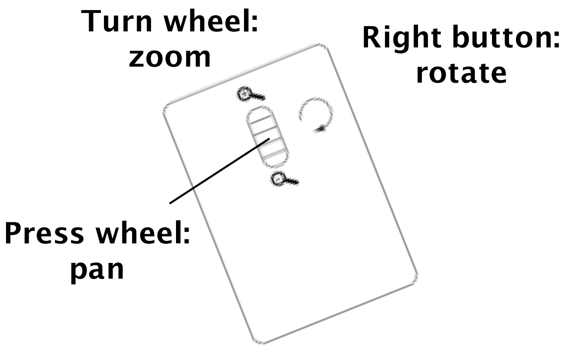

The axis is controled by the wheel and the right button of the mouse. This keeps the left mouse button free for editing.

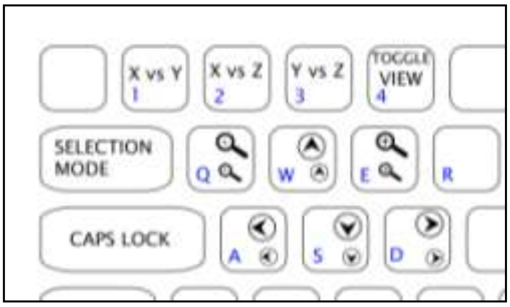

Keyboard shortcuts are crucial in the process of reconstruction from image stacks and manual editing of trees. Here are some keyboard controls for the axis. The keys [1], [2] and [3] are absolutely crucial to switch between different view and editing planes. [q] and [e] control the zoom and [a], [w], [d] and [s] pan the axis.

The GUI the vis_ panel

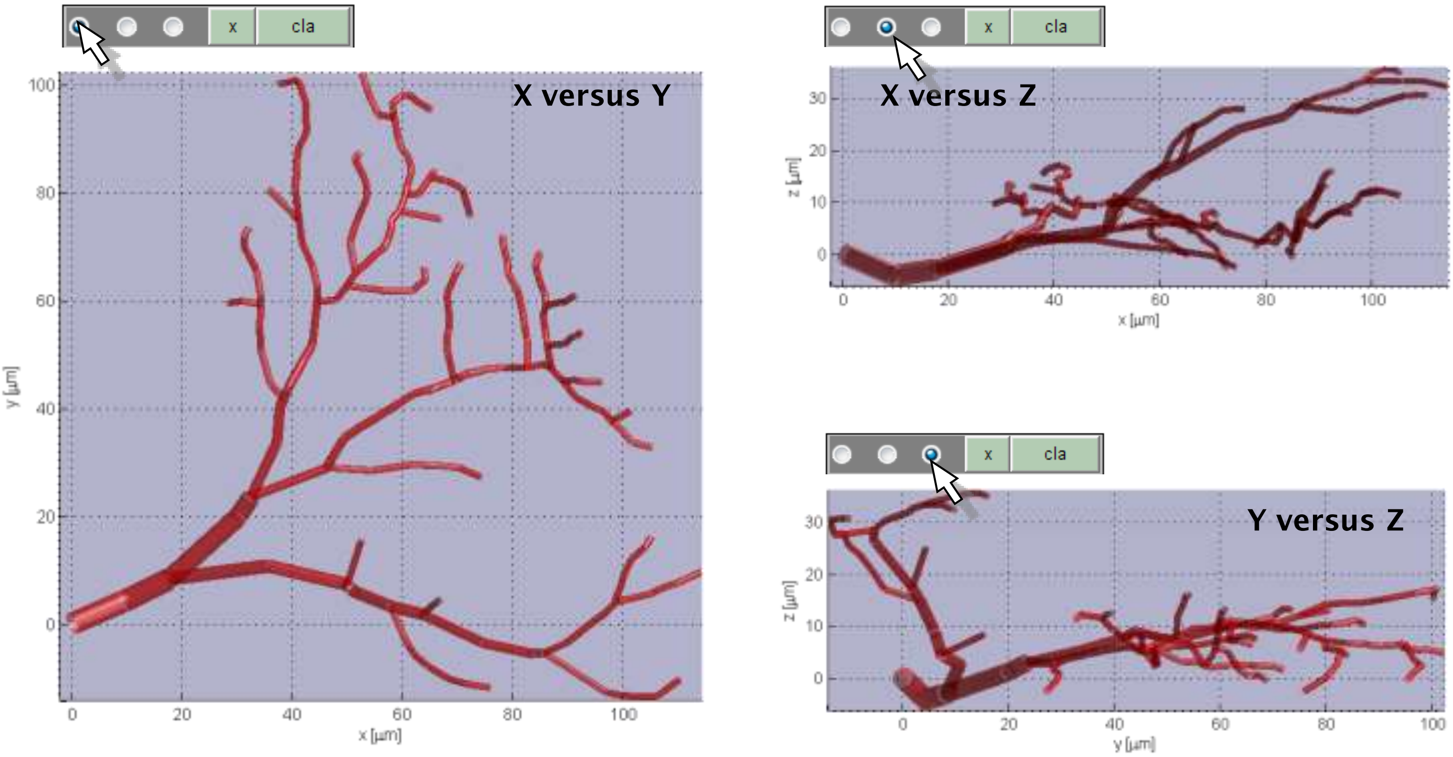

The 2D views are essential for editing trees and image stacks. When one of the three 2D views is selected (by either one of the radio buttons shown on top, by toggling with the „2D“ control or by using the [1], [2], [3] or [4] keys) this becomes the editing plane and all actions are performed accordingly. For example, manual editing of node positions in a tree or the manual rotation of an entire tree happen in that plane. Further axis changes can be made on the secondary view controls:

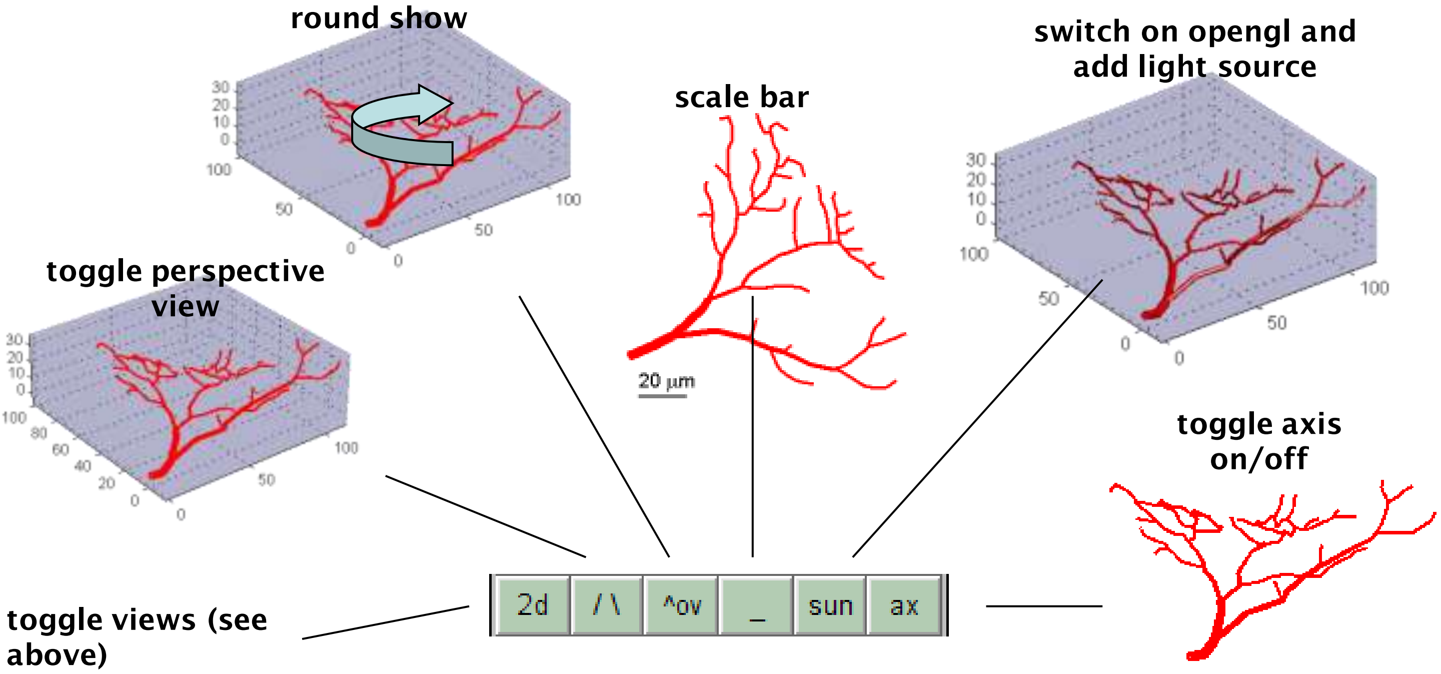

The GUI the vis_ panel

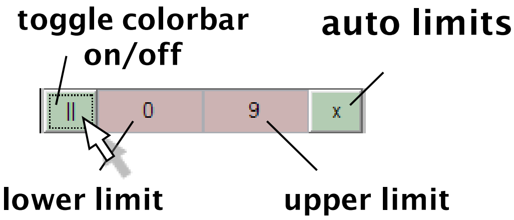

If the plotted image contains color-coded values (e.g. the brightness values in an image stack or in this case the mapping of branch orders on the sample tree), a colorbar can be summoned up using the colorbar controls. By default the limits are set automatically. However, these can be manually adjusted using the two edit fields.

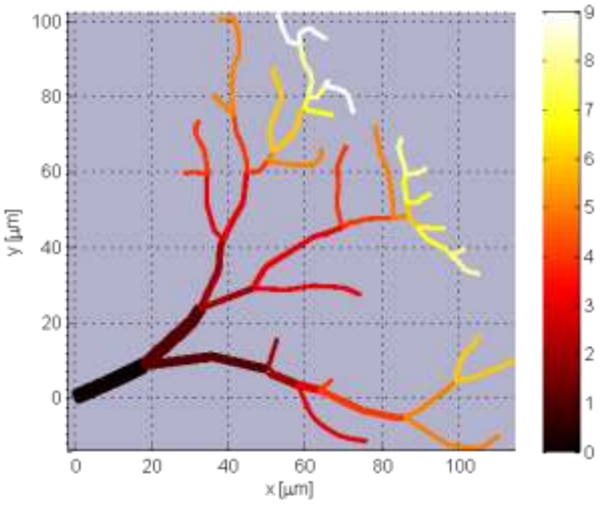

The colormap can be chosen in the menu. Also, by default, the colormap is transparent but it can be switched back to opaque in the same menu. This also affects the image stack representations (but not the trees).

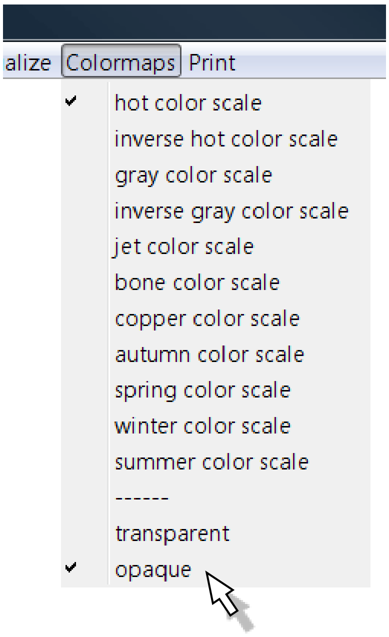

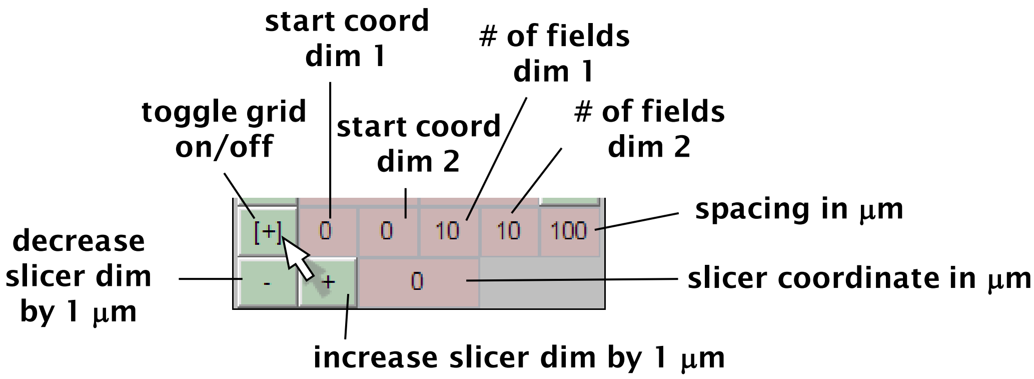

The grid button toggles on/off the grid. It settles in the plane corresponding to the active view at the coordinate given by the slicer field. Grid field coordinates are set according to the two dimensions of the active plane. The slicer coordinate becomes important later for image stack representation and manual editing of a tree.

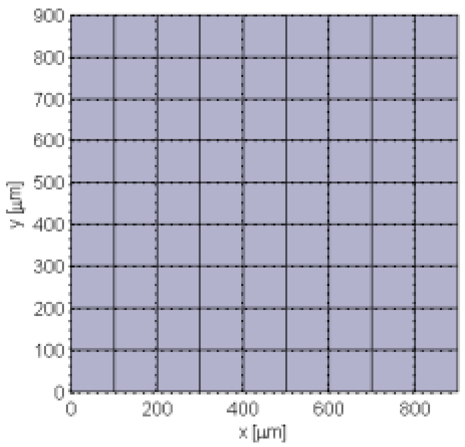

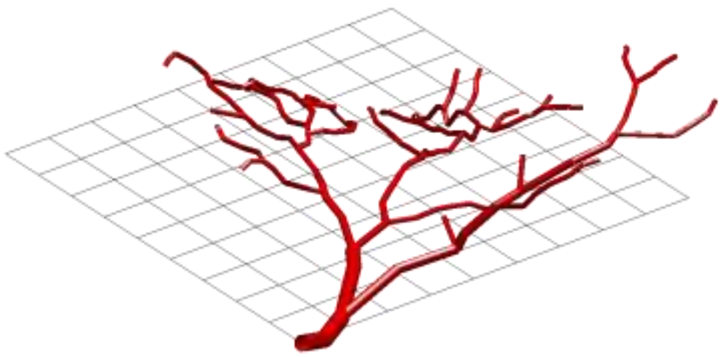

the stk_ panel: sorting the image stacks

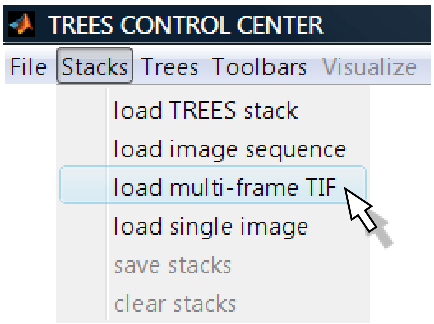

For reconstruction purposes or just to compare a tree with its underlying image stack, load image stacks or single images using the menu. The TREES internal .stk format stacks are just binary Matlab workspaces and can be read out in comand line using the matlab „load“ function (but see also „load_stack“). In the following we will assume that the first goal is a reconstruction of a tree present in tiled image stacks. The stk_ panel is then the first editing panel required. The image stacks are loaded sequentially and the last stack in the popup control is the active stack.

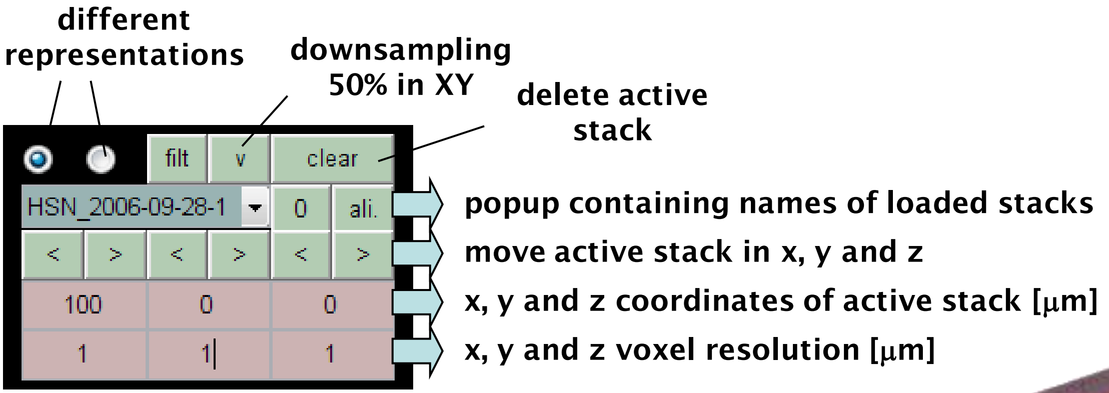

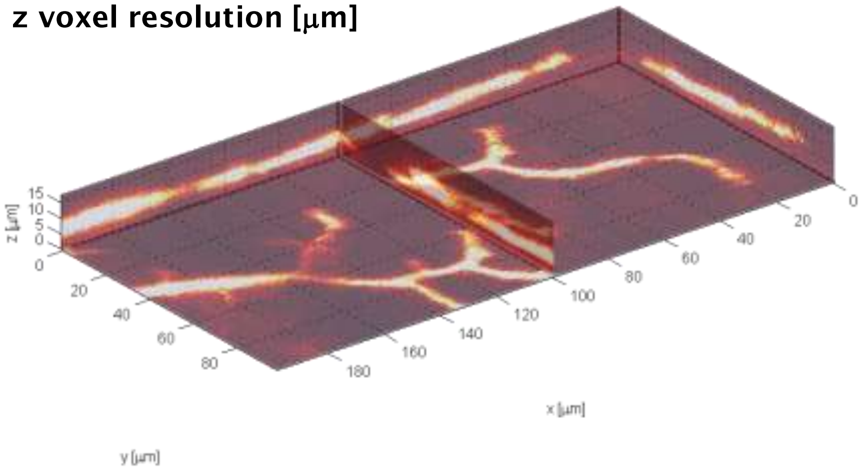

This is a good place to mention that „cgui_tree“ stores all information in a global structure called „cgui“. To access the data relating to a specific panel (here the stk_ panel) first „cgui“ is required to be global in the general workspace. The field name in the structure corresponds to the panel prefix. For example the 2 matrices containing the image stacks can be read out as:

```
>> global cgui
>> cgui.stk.M

ans =

    [100x100x19 uint8]    [100x100x19 uint8]
```

The GUI   the stk_ panel

Two visual representations are offered by the stk_ panel. One in which for each image stack all three maximum intensity projections are shown. The other one (the slicer) shows for each image stack the slice (according to the viewing plane illustrated below using the grid) which is closest to the vis_ panel slicer values (see „the vis_ panel“).

maximum intensity projections

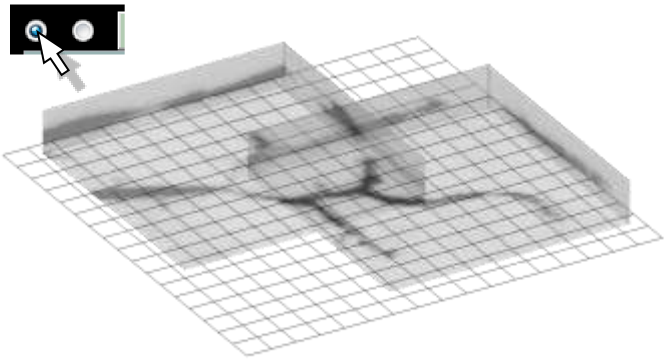

slicer image

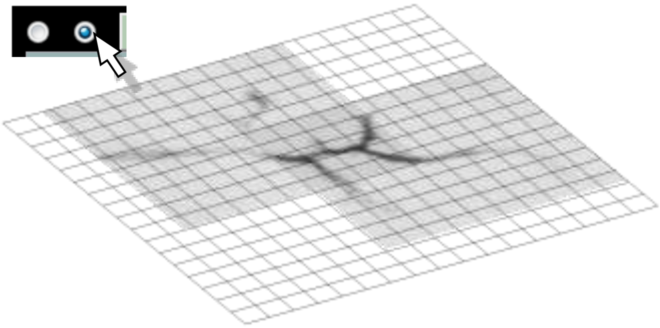

The active stack (the last one in the popup) can be automatically aligned in 3D to the stack preceding it, if sufficient image overlap allows it. A stack‘s coordinates can also be set back to zero with the „0“ control.

The stack coordinates can be set directly in the edit fields. There, the voxel size can also be set (here the z-dimension is set to 2µm while x and y are 1 µm each):

align two image stacks

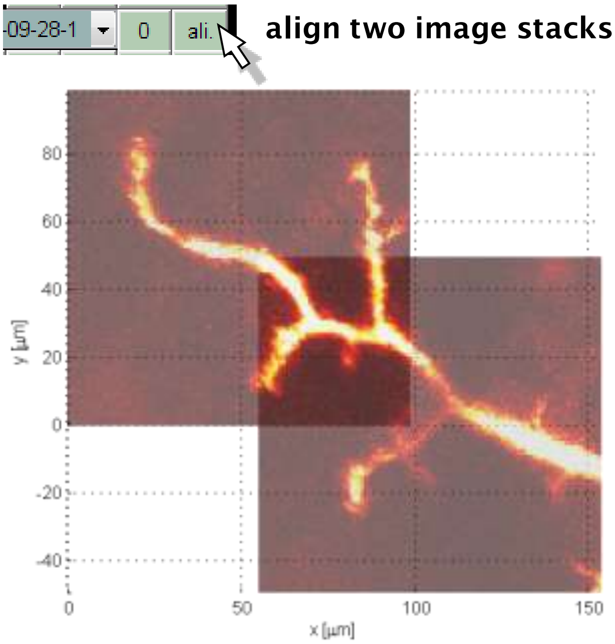

change voxel size

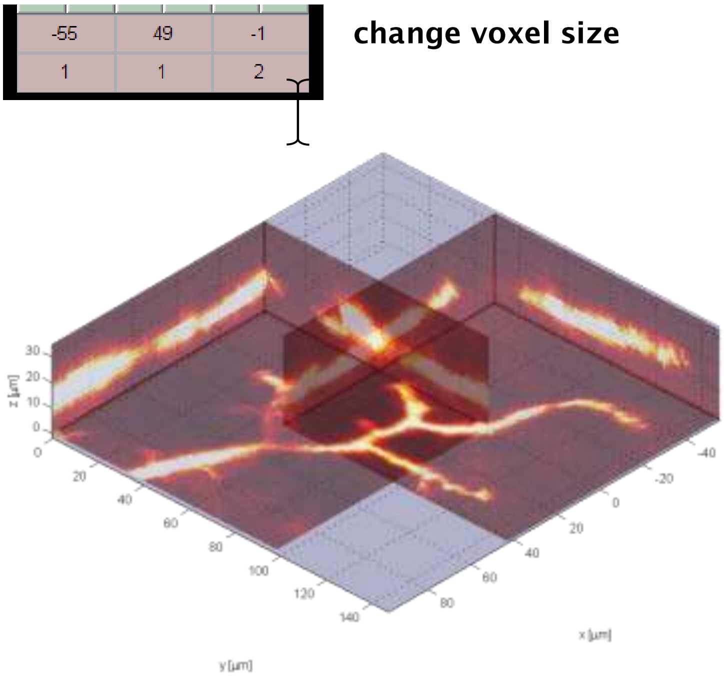

entering the edit mode

One of the most sophisticated features of the GUI is that each of the 5 edit panels (stk_, thr_, skl_, mtr_ and ged_) has individual edit modes; some of them even have an alternative edit2 mode and several corresponding submodes. The selected active panel for editing is chosen by using the up and down arrows on the keyboard. Alternatively, press ctrl [q], [w], [e], [r] or [t] depending on which of the five panels is needed. The selected active panel has an increased frame size. To enter the edit mode press the edit control in the vis_ panel, press the left arrow key, or press shift [1] (i.e. [!]).

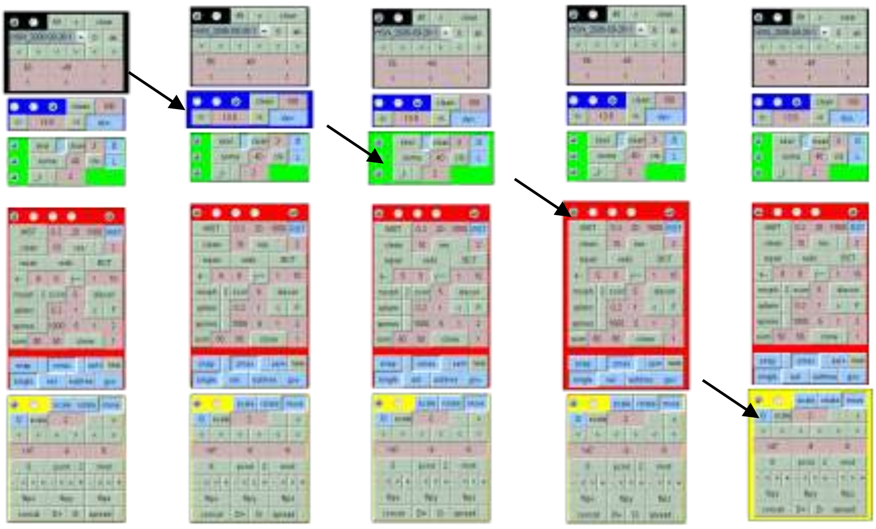

When entering the edit mode the mouse cursor becomes a circle and an edit line appears. The alternative edit2 mode turns on when the edit mode is on and additionally the vis_ panel edit2 control is pressed or the right cursor key is pressed or shift [4] (so [$]). Typically, the edit lines then become yellow.

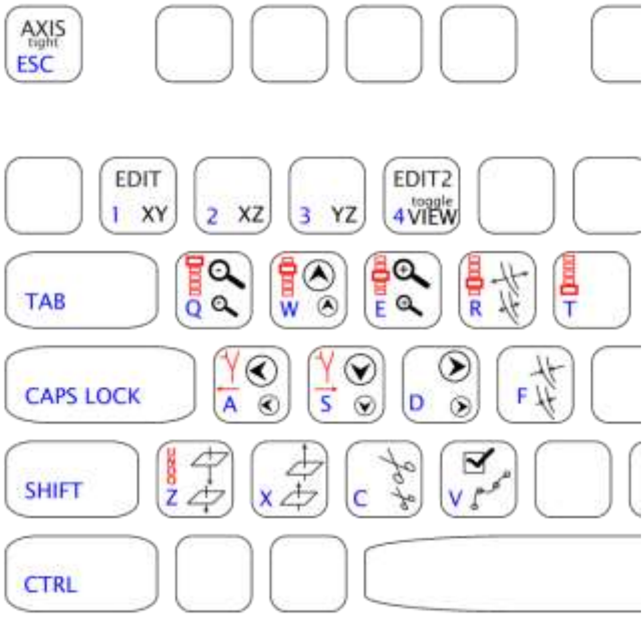

The keyboard layout can now be extended from the simple axis control to the full edit control. In red are keys with additional „ctrl.“ press. ctrl [z] is an undo function in the tree edit mode. ctrl [a] and ctrl [s] switch between two trees of the same group (see later). [z], [Z], [x] and [X] decrease and increase the slicer coordinate respectively. [c] and [C] are general overloaded cutting keys. [v] is a preview for a reordering and [V] performs the reordering of nodes in the skeletonization and tree panel. [r], [R], [f] and [F] increase and decrease the diameter of tree nodes or editing elements depending on the active editing panel.

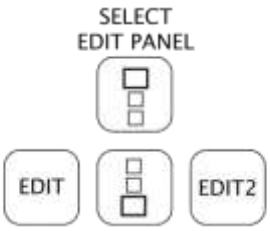

stk_ edit mode
In the stk_ panel edit mode the starting coordinates of image stacks can be set. The editing depends on the selected view mode, so at any given point in time it happens only in one plane. The green edit line indicates which stack is closest. A double-click activates that stack if it is not active yet.

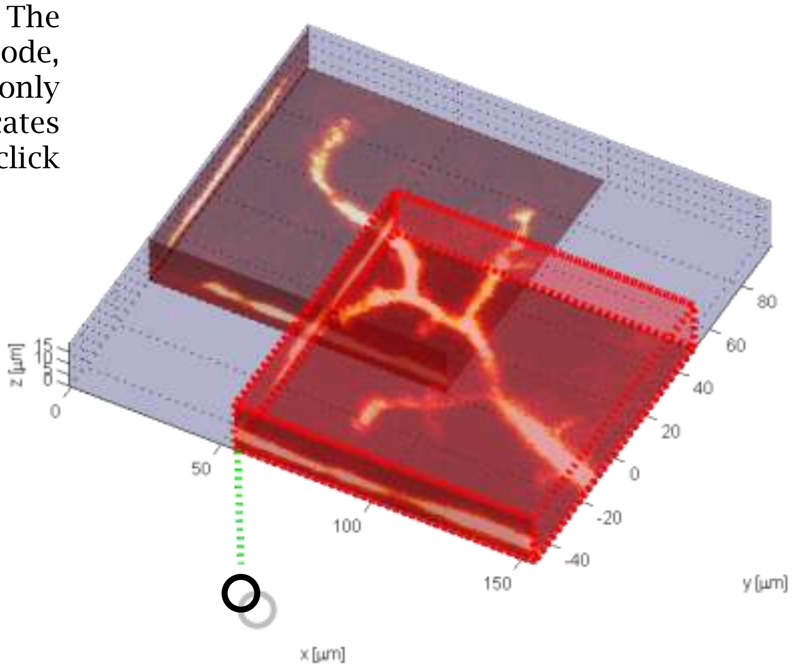

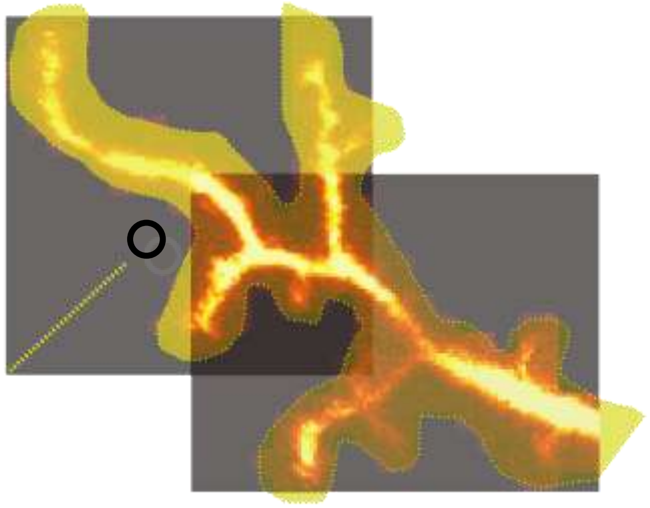

stk_ edit2 mode
In the stk_ panel edit2 mode, image stack pieces can be cut out according to a region of interest (ROI). The ROI is planar and is drawn according to the actual viewing plane. With key-press [c] the brightness values inside of the ROI throughout the slicer dimension are set to 0. With [C] the outside of the ROI is treated correspondingly. The yellow edit line indicates in which image stack the procedure is done: in this way the same ROI can be used to edit different image stacks separately. For speed reasons the borders are not kept sharply. After cutting, the image stacks are cropped rectangularly off of all zero rows or columns.

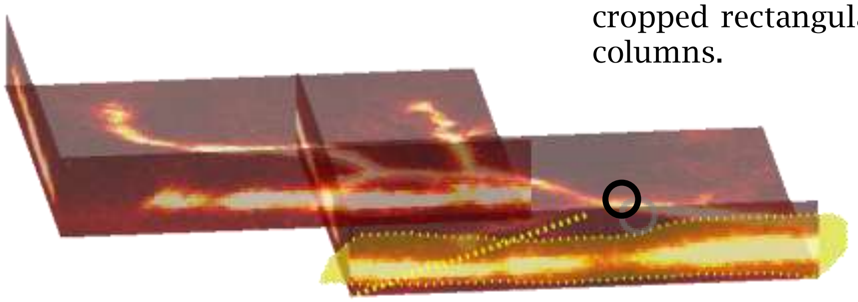

the thr_ panel: binary images through thresholding

different representations

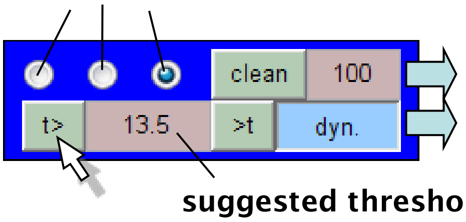

morphological cleaning

binary thresholding

suggested threshold

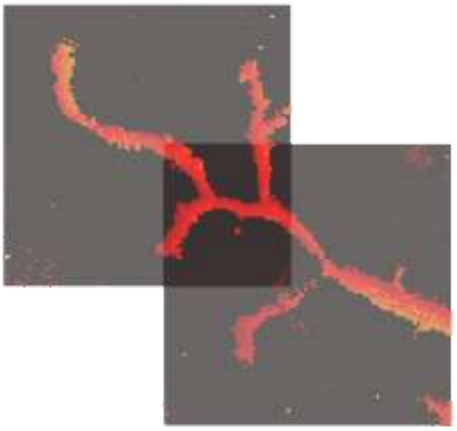

With the thr_ panel a set of binary matrices is constructed by thresholding the original image stacks. If the „dyn.“ toggle is pressed down a local (or dynamic) threshold is taken. This is particularly advantageous to capture small branches. The edit value is an offset to the local mean brightness necessary to be part of the binary matrix. Without dyn. thresholding the edit value indicates the actual absolute threshold value. The vis_ panel displays the percentage of voxels which fall in the binary matrix. That should typically not exceed 2-3%. When the third radio button is switched on, the maximum intensity projections of the stk_ panel show the index of thresholded maximum values, which in the XY view for example corresponds to the z values (between 0 and 1), at which the brightness is maximal in the original image stack.

With the first radio button switched on, little blue transparent tiles are drawn on the maximum intensity projections to show which voxels will be kept in the binary matrices.

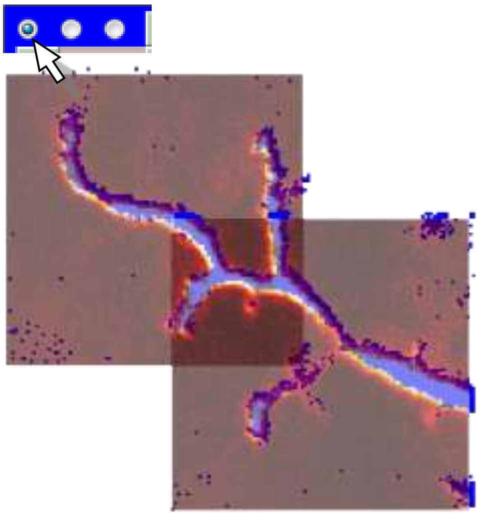

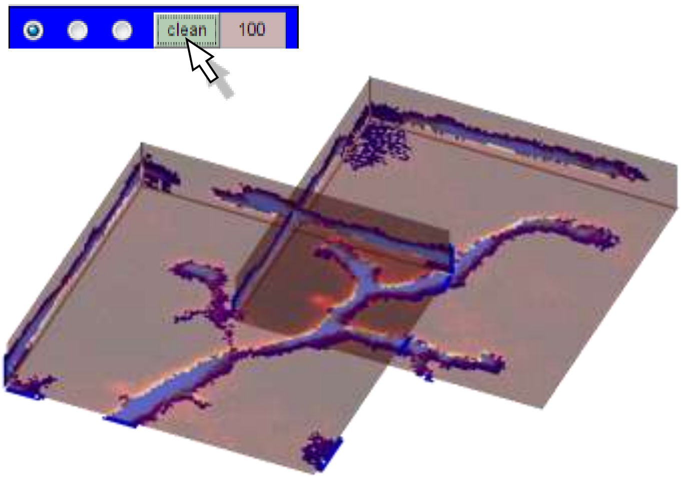

Cleaning the binary matrix involves linking voxels by neighborhood relationships. When a set of linked voxels is smaller than the cleaning size in the edit field, it is removed. This is an efficient way to remove noisy bits off of the thresholded image (see Matlab function „bwareaopen“).

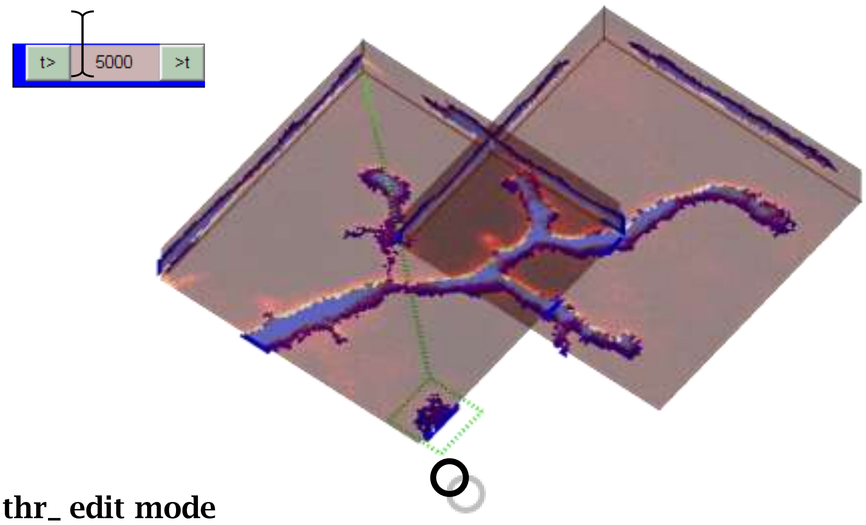

thr_ edit mode
In the thr_ panel edit mode the binary matrices can be manually edited by setting an individual threshold in a marked area. This method allows to change the threshold value in the edit field and then clean the binary matrix locally. The size of the local areas can be altered with the keys [r], [R], [f], and [F]. To switch to a different image stack it is necessary to switch back to the stk_ panel.

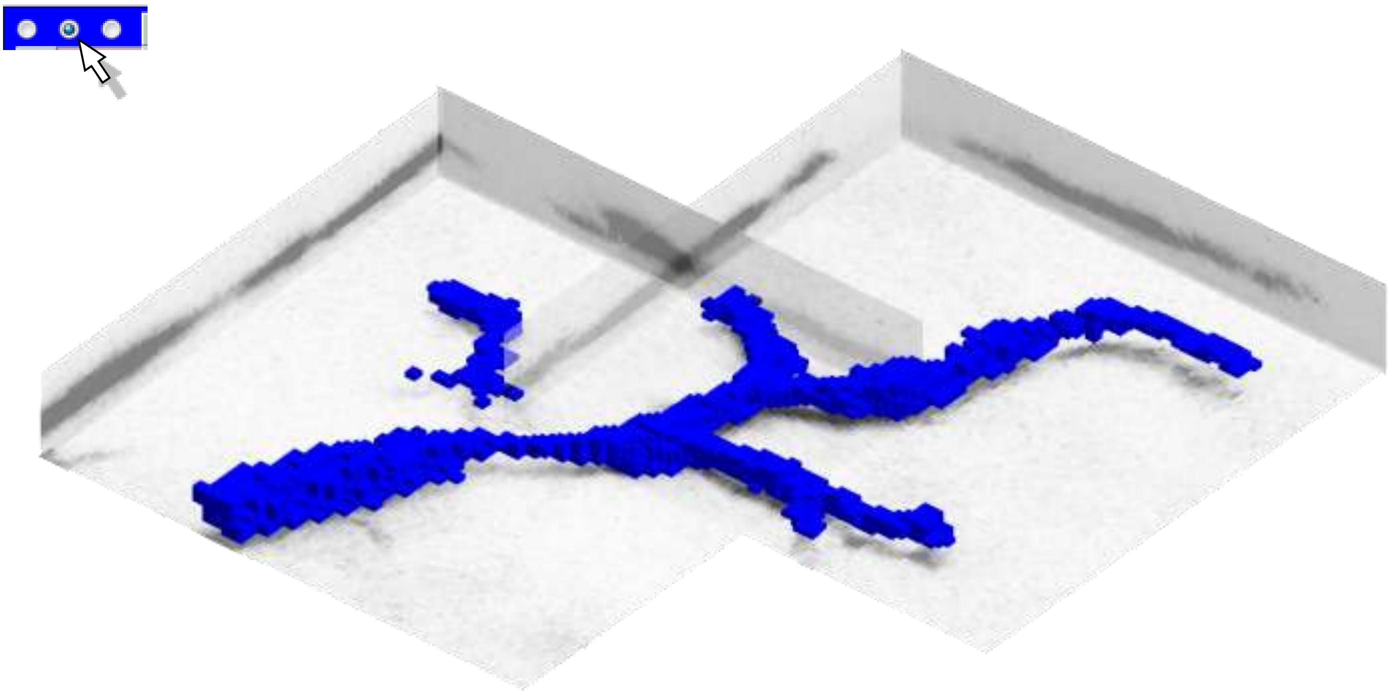

Finally, in order to check the integrity of the binary matrix and its flow between the tiled image stacks, the second radio button can be activated (this can be very slow). This shows a 3D box representation of the binary matrices at a reduced resolution (2x2 voxels in the XY plane).

the skl_ panel: skeletonization

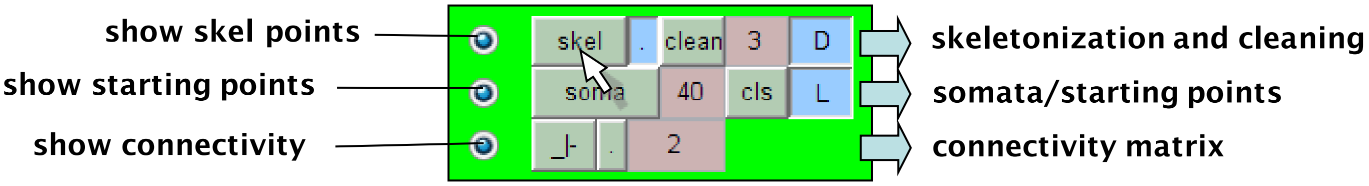

The skl_ panel is there to reduce the binary matrices to a set of individual carrier points and some starting nodes to be subsequently connected to trees. The 3D skeletonization is a process which carves off binary voxels one by one in dependence of their neighborhood (a method introduced by Palagyi and Kuba), ideally leaving only the carrier nodes of the branched structure (see „skel_stack“). Pressing the „skel“ button performs the morphological operation. If toggle „D“ is pressed, a diameter value is obtained for each node from the neighborhood relationships of the binary matrix: *D* is the distance of the closest zero value in the binary matrix (see „bwdist“ from the image processing toolbox in Matlab). If toggle „L“ is pressed, a labeling is performed on the binary matrix: two nodes are connected if walking on non-zero voxels from one to the other without gaps is possible. This is helpful for calculating the connnectivity matrix later. Also it helps to chose starting points: two starting points should never show up on the same „label“ (see „bwlabeln“ from the image processing toolbox in matlab).

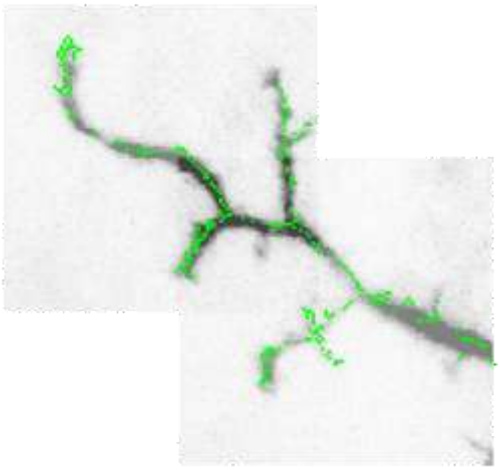

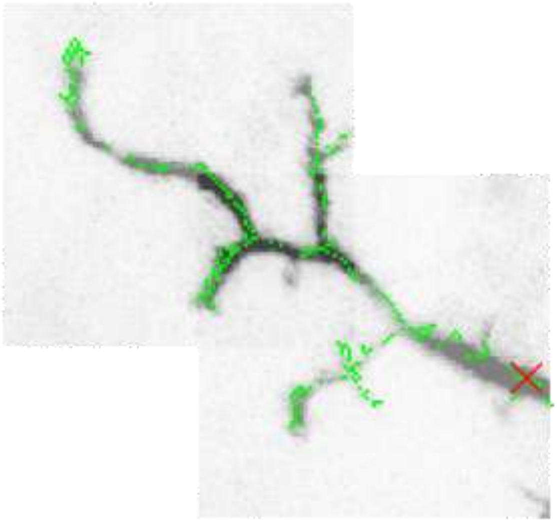

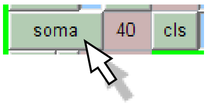

The skl_panel is equipped with a very basic soma finding function. This takes the threshold value (***thr***) in the edit field and finds either all nodes whose diameters were calculated to be higher than ***thr***. If none falls in that category the node with the highest diameter is chosen (as in this case). If more than one node falls in that category then smaller starting nodes which appear in distance smaller than ***thr*** of larger starting nodes are cleaned away. Starting nodes which appear on the same label are also deleted.

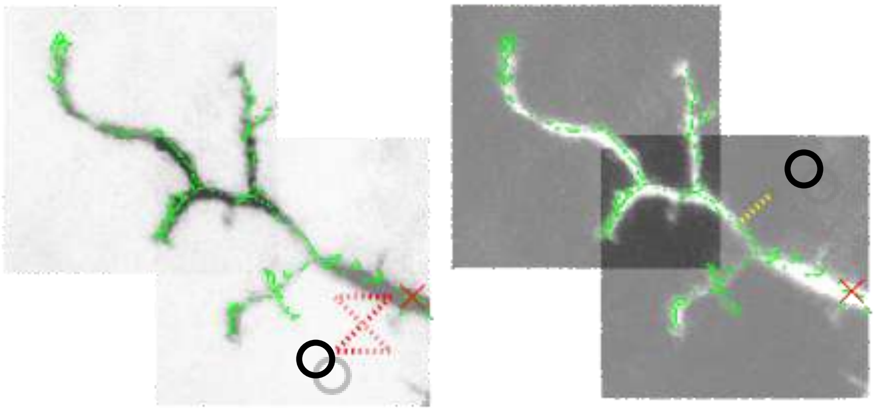

skl_ edit and edit2 mode
The skl_panel allows alteration of starting node locations in the edit mode and of skeletonization carrier point locations in the edit2 mode. Points are moved in the current viewing plane and can be deleted with the key [c]. A line shows to the closest point. In edit2 mode key [C] cuts out all carrier points in close vicinity.

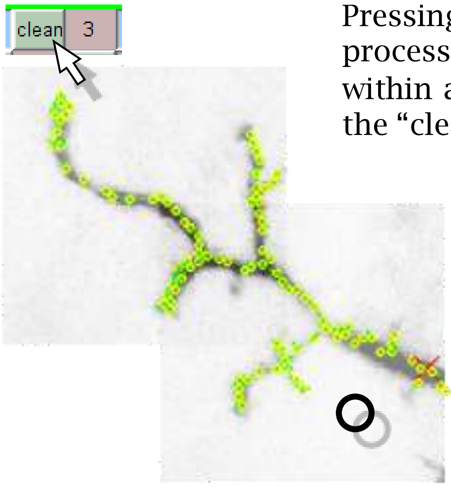

Pressing [v] in the edit2 mode previews the result of cleaning. This process reduces the number of nodes by preventing neighboring nodes within a distance limit in μm indicated in the edit field. Pressing [V] or the “clean” key finalizes the sparsening of the nodes.

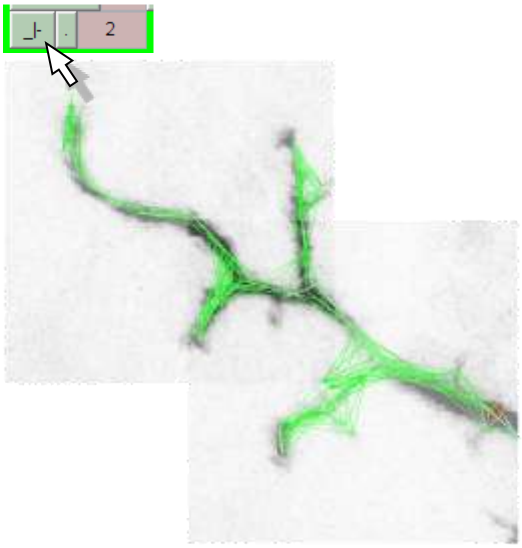

The nodes and starting nodes are then ready for the tree constructor. However to improve the result further the MST tree constructor with “RST” option accepts a distance matrix between the nodes to increase the probability of connection. Threshold-linking is a standard way to do this. The resulting connectivity graph of increased connection probability is shown when the third radio button is active.

the mtr_ panel: constructing a tree

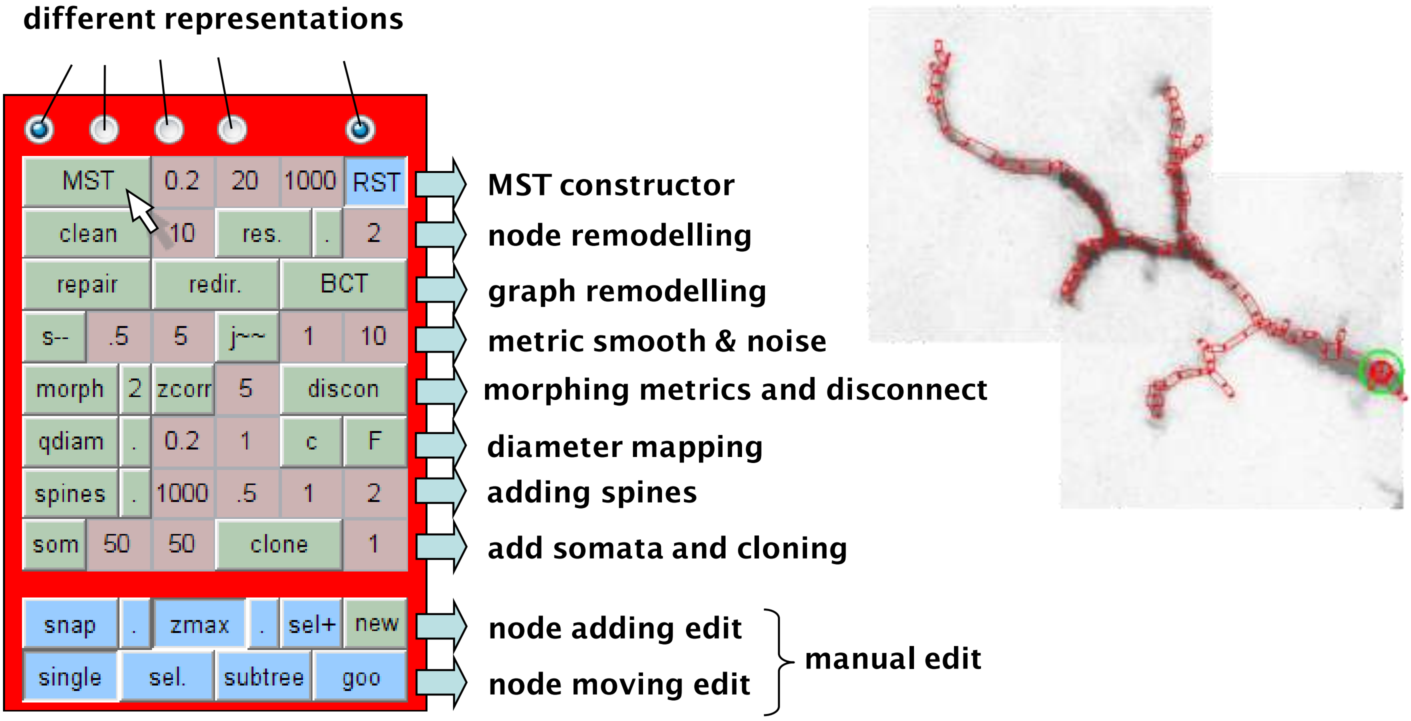

The mtr_ panel is the most intricate panel and also the core panel in the TREES toolbox GUI. Artificial trees can be constructed using different methods such as automated reconstruction, cloning or fully manually. The trees can be remodelled in many different ways including smoothing, introducing jitter, resampling, diameter tapering, addition of spines and somata or manual remodelling. Equivalent trees can be obtained, trees can be morphed or flattened or cut to pieces, etc.. The edit modes in this panel have multiple sub-modes allowing the editing of single nodes, selected nodes, sub-trees etc...

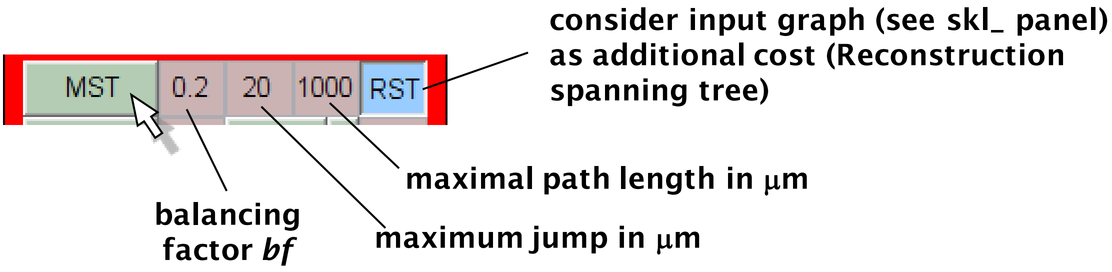

In the automated reconstruction process the first step is to connect the skeletonized points obtained by the skl_ panel to a graph. This is done in the TREES toolbox using the MST constructor (see „MST rule“ in the introductary part) following a greedy algorithm to obtain an extended minimum spanning tree. The constructor is launched with the „MST“ button in the mtr_ panel.

The GUI   the mtr_ panel

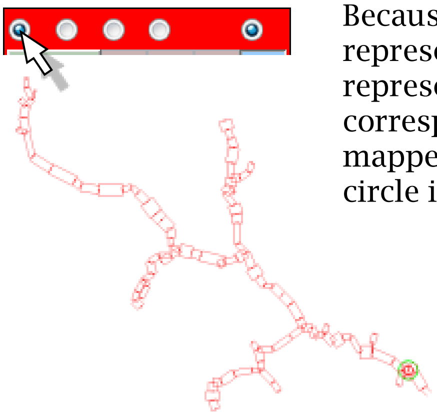

Because of the central role of the mtr_ panel a number of different representations exist. By default (first radio button), a tree is represented as rectangular pieces between two connected nodes corresponding to the cylinder or frustum. The rectangular pieces are mapped to the active vis_ plane, in this case in the XY-plane. A green circle indicates the last activated node (here the root).

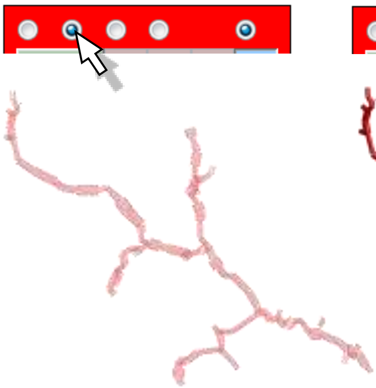

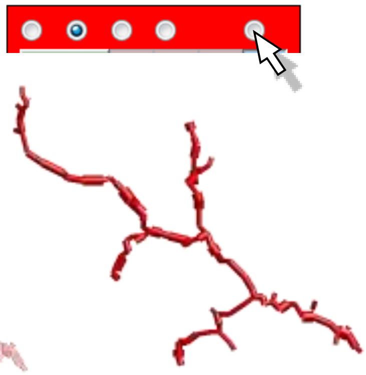

Additionally, the second radio button allows a 3D visualization of the tree. The last radio button toggles transparency. In order to set higher or lower cylinder resolution go to the “Visualize” menu.

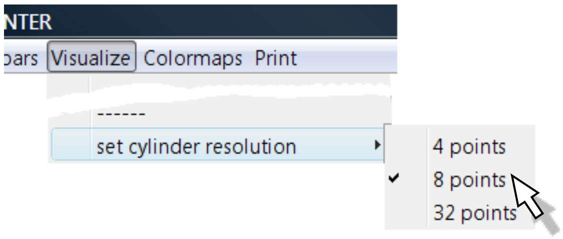

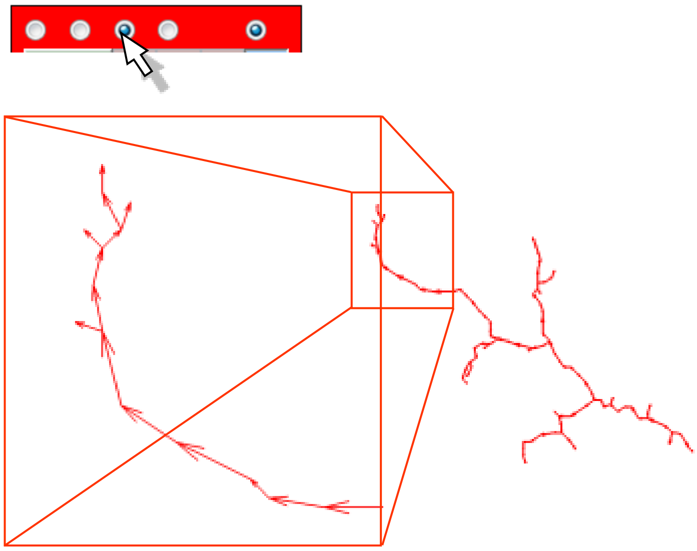

As an alternative to the second option a graph representing the edges between nodes as arrows can be selected using the third radio buttion.

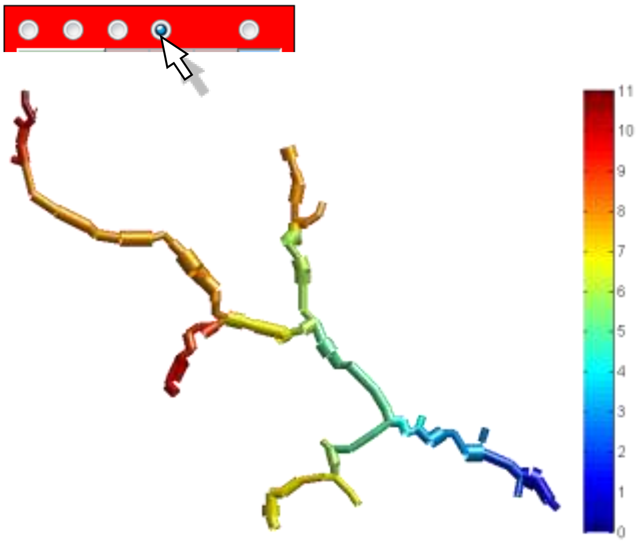

If an *Nx1* vector has been selected with the slt_ panel (see later), this can be mapped on the colour values of the tree again as an alternative to the second option. This happens when toggling ON the fourth radio button.

The GUI the mtr_ panel

The resulting tree from the reconstruction process is ready for manual editing (see later) but can also simply be cleaned up with different cleaning algorithms

radius determining the close vicinity in μm

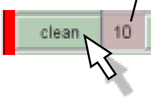

Clean a tree

Cleaning a tree (<u>see "clean_tree"</u>) is a process in which improbable nodes are eliminated. Termination points in close vicinity of other nodes on a different branch and very short terminal branches are deleted. Consecutive calls of this function can be useful.

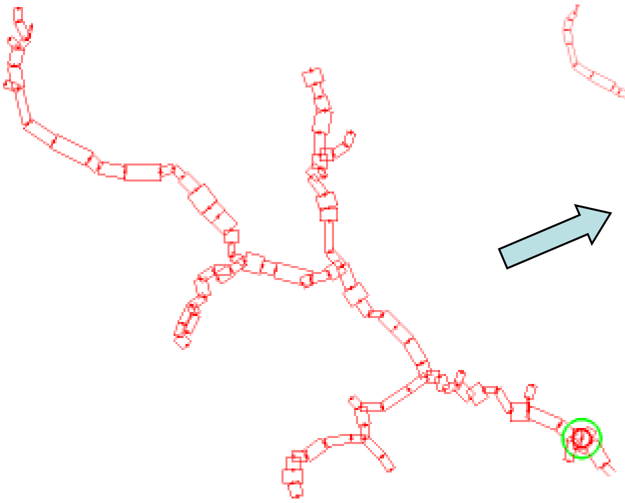

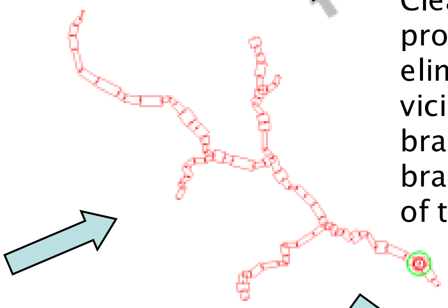

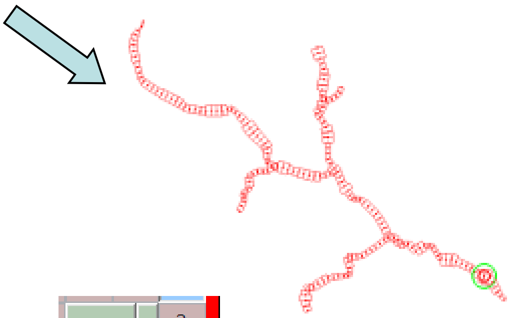

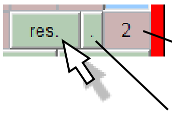

target internode distance in μm

length conservation (stretches tree)

Resample a tree

Resampling a tree (<u>see "resample_tree"</u>) redistributes nodes such that segments are constant length. This is required to get a unique graph representation, and to apply functions such as adding a spatial jitter or a number of spines homogeneously.

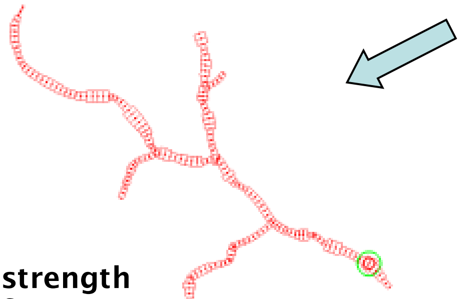

smoothing strength
number of iterations

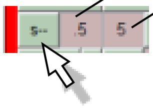

Smoothing a tree

Smoothing a tree (<u>see "smooth_tree"</u>) along its longest paths.

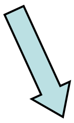

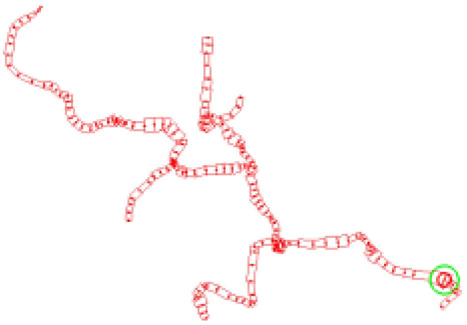

jitter amplitude

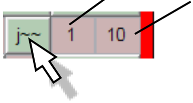

length constant of noise filtering in μm

Add spatial jitter

If additional jitter is desired it can be applied here (<u>see "jitter_tree"</u>).

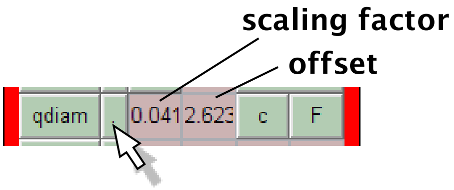

Fit quadratic taper (see „quadfit_tree“)

Fitting scaling and offset parameters for the quadratic taper. In this case it results in an almost constant diameter throughout.

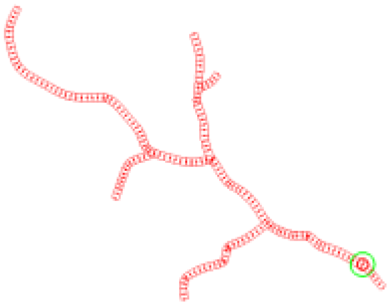

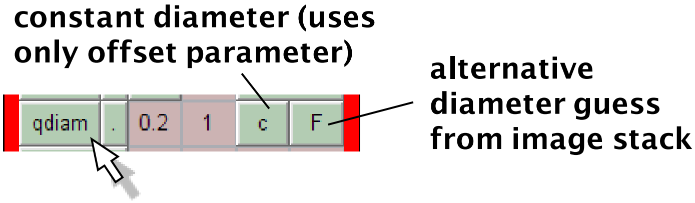

Quadratic taper (see „quaddiameter_tree“)

Scaling and offset parameters can also be set by hand.

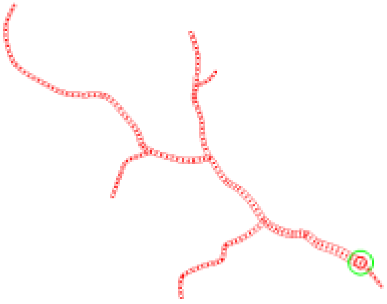

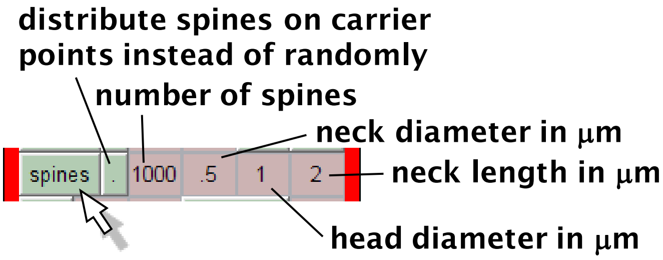

Add spines (see „spines_tree“)

Distributes a number of spines either attached to nodes randomly (among selection if exists) or on skl_ panel carrier point locations.

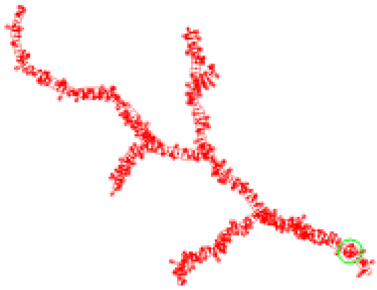

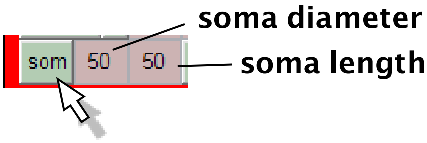

Add soma (see „soma_tree“)

Increases diameter in the vicinity of the root of the tree.

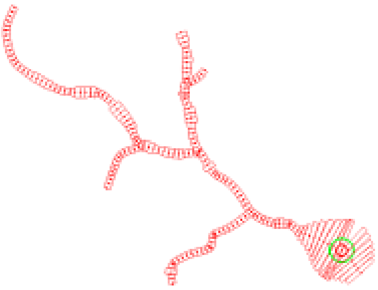

mtr_ edit mode
When the edit mode is turned on and the mtr_ panel is active, the active tree is drawn in thick red lines. The red dashed edit line indicates which node is closest. With simple mouse clicks and holding, new branches can be drawn.

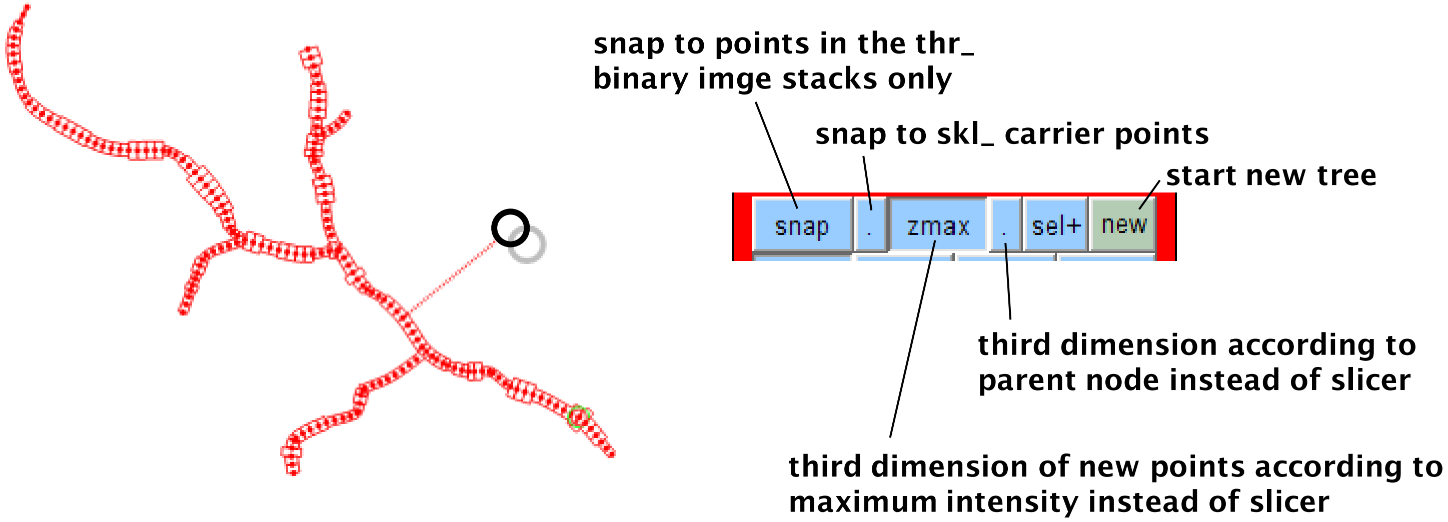

mtr_ edit submodes for altering the node locations
In the mtr_ edit mode clicking near an existing node (edit line becomes green) allows the user to move the node around with the active plane. Different submodes allow the movement of a single node (single), all selected nodes (sel.), an entire sub-tree (subtree) or nodes in the close vicinity where the amount of displacement depends on the distance to the originally selected node (goo)

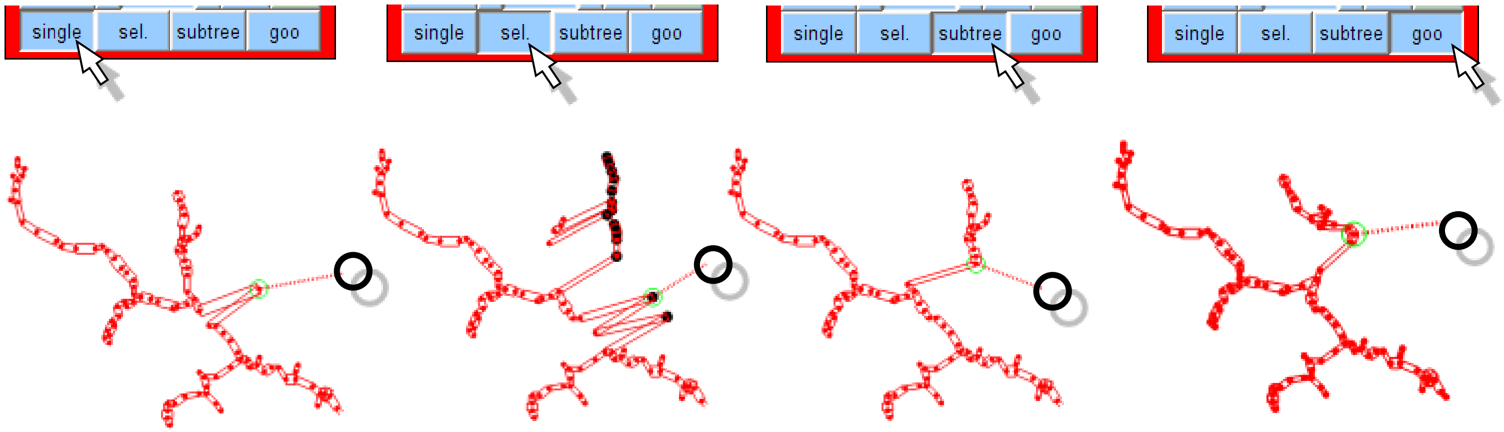

mtr_ edit-select mode

When the edit mode is turned on and the mtr_ panel is active, the active tree is draw in thick red lines. The red dashed edit line indicates which node is closest. With simple mouse clicks and holding, new branches can be drawn. As can be seen here clearly and in the previous example the green circle demarcating the last activated node moves while clicking on the tree for editing.

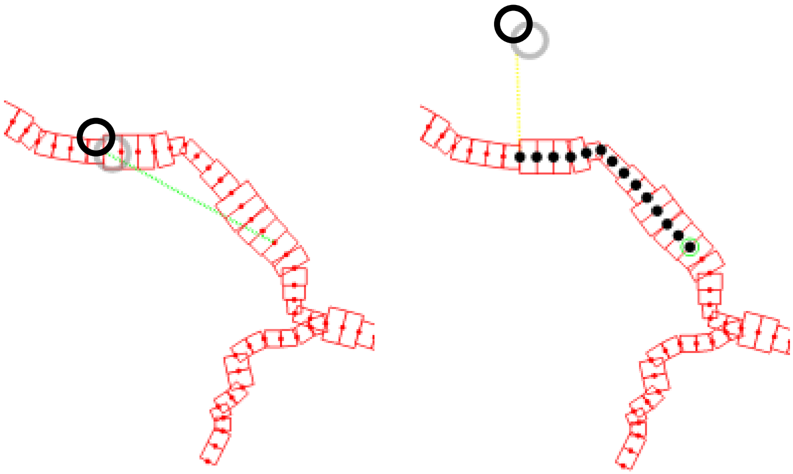

toggle add to/replace selection

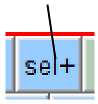

redirect and repair

Finally, the root can be moved to another location by redirecting the underlying graph of the tree (see "redirect_tree") and the adjacency matrix can be sorted (see "repair_tree") in order to correctly perform operations such as the dendrogram plotting and to obtain a unique representation of the tree.

sorts the node labels and eliminates 0-length segments and trifurcations

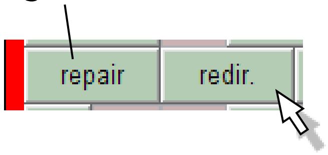

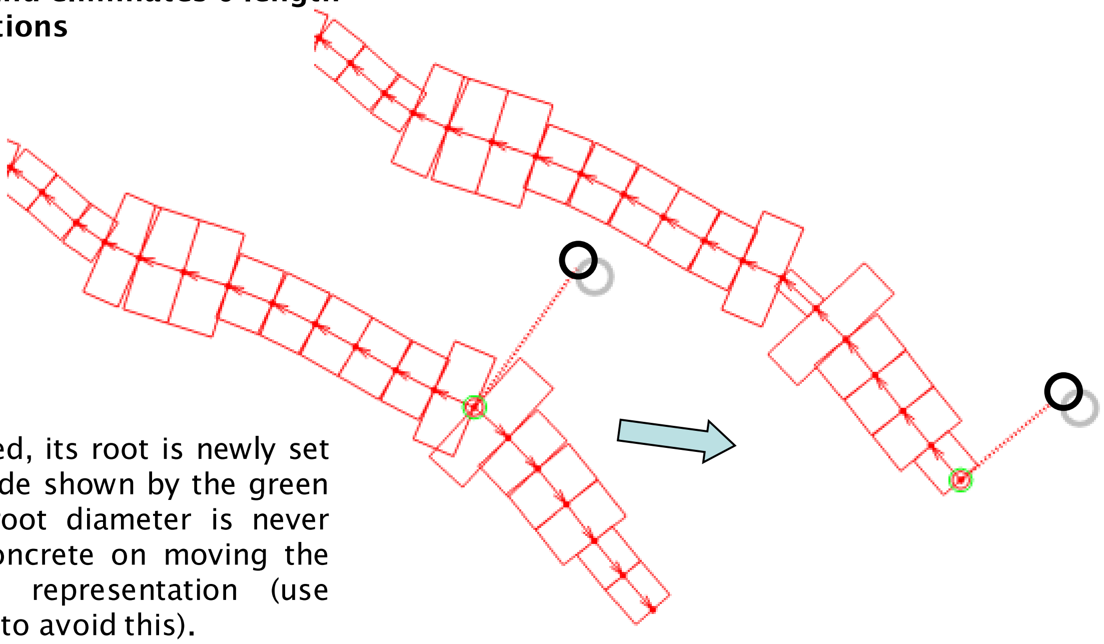

notes on redirect

When a tree is redirected, its root is newly set to the last activated node shown by the green circle. Note that the root diameter is never visible but becomes concrete on moving the root in the cylinder representation (use frustum representation to avoid this).

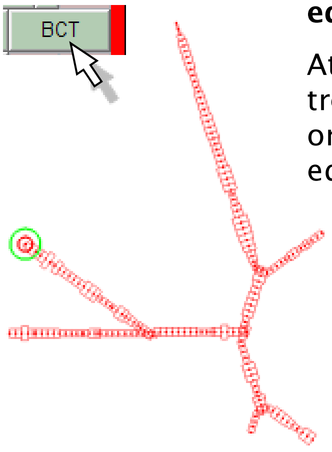

equivalent tree (see „BCT_tree“)

Attributes a set of new unique metrics to a tree according to a dendrogram-type of organization. Electrotonically this tree is equivalent to the original.

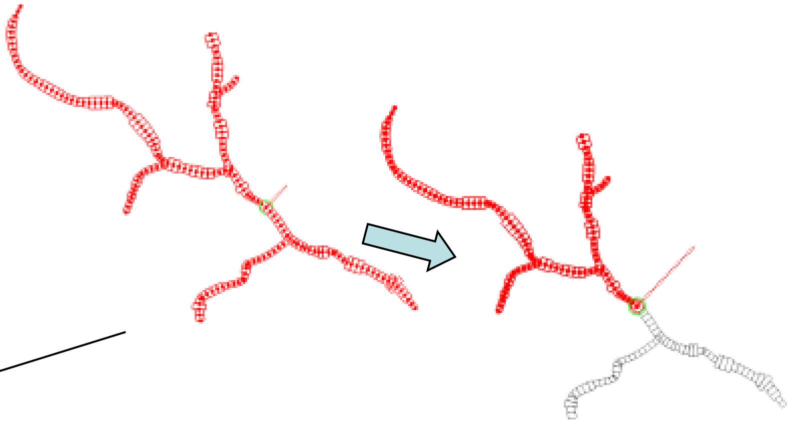

flatten a tree (see „flatten_tree“)

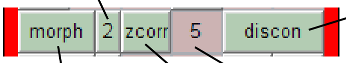

threshold for z-correction in μm

disconnect a sub-tree

Unconnect a sub-tree from the main tree at the last activated node (green circle, is set in the edit mode). The ged_ and cat_ panels allow rearrangement of the resulting trees and a possible reconnection.

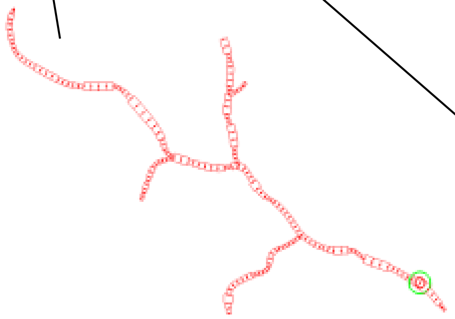

correct tree in z (see „zcorr_tree“)

Sometimes trees might contain unexpected jumps in the z-dimension, this can be corrected here.

morphing (see „morph_tree“)

Attribute *Nx1* vector values to the lengths of the indidual segments selected by the slt_ panel (see later). Here for example diameter values are mapped to the length (doesn’t make much sense...).

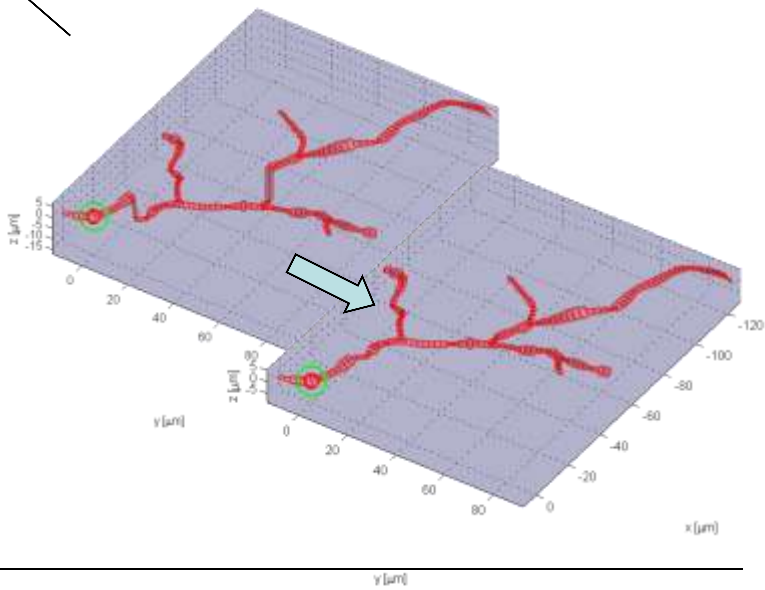

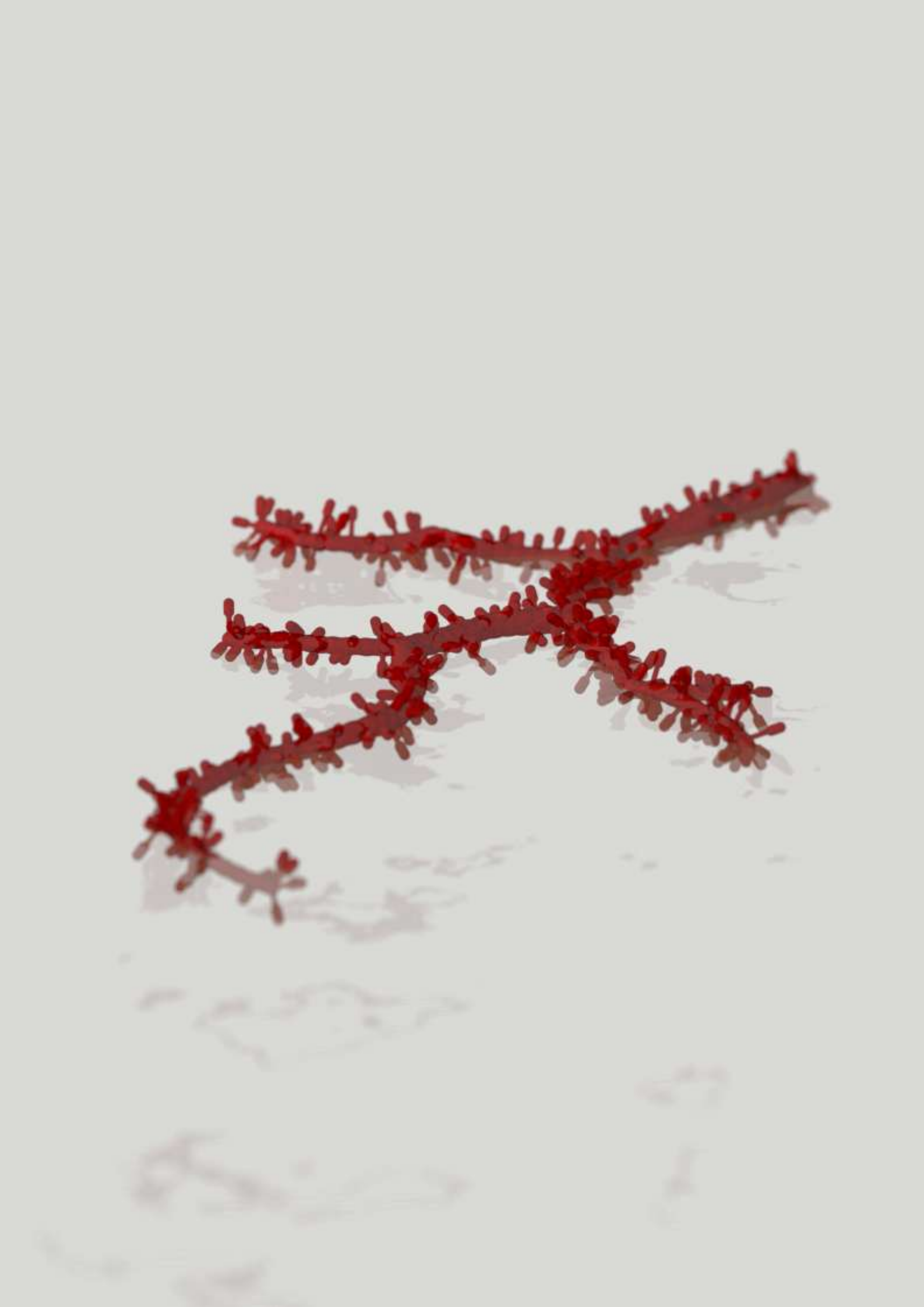

the cat_ panel: browse trees

The cat_ panel allows to browse through the two levels of depth of tree structures. The numbered list on the left indicates the different sets. One set can contain just one tree or more than one. The two „up“ and „down“ buttons reorganize the active tree location within the different sets. This is not yet very practical but allows in principal the rearrangement of any set of trees into new groups. In general it will be better to sort trees in different groups using short bits of code in matlab.

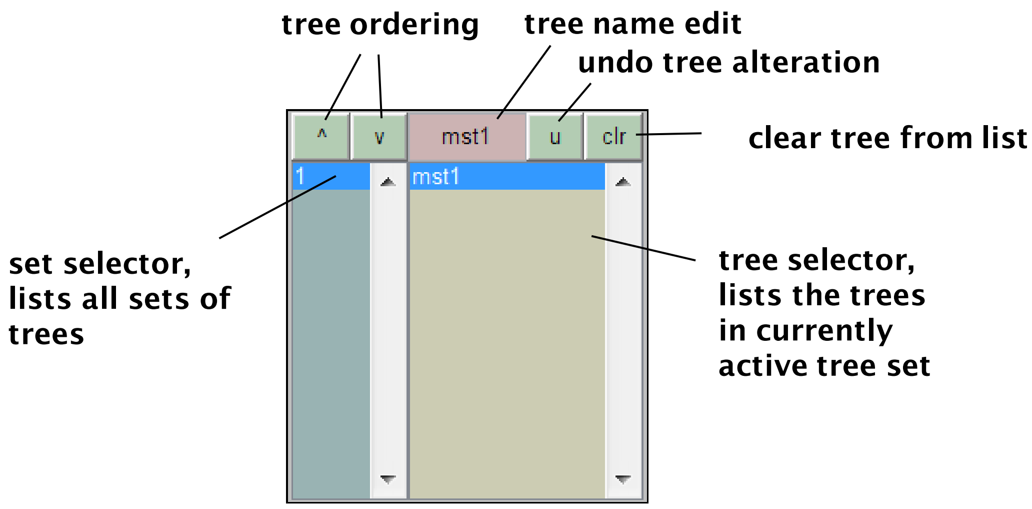

When new trees are loaded or are generated newly, they are automatically appended to the sets of trees in the cat_ panel.

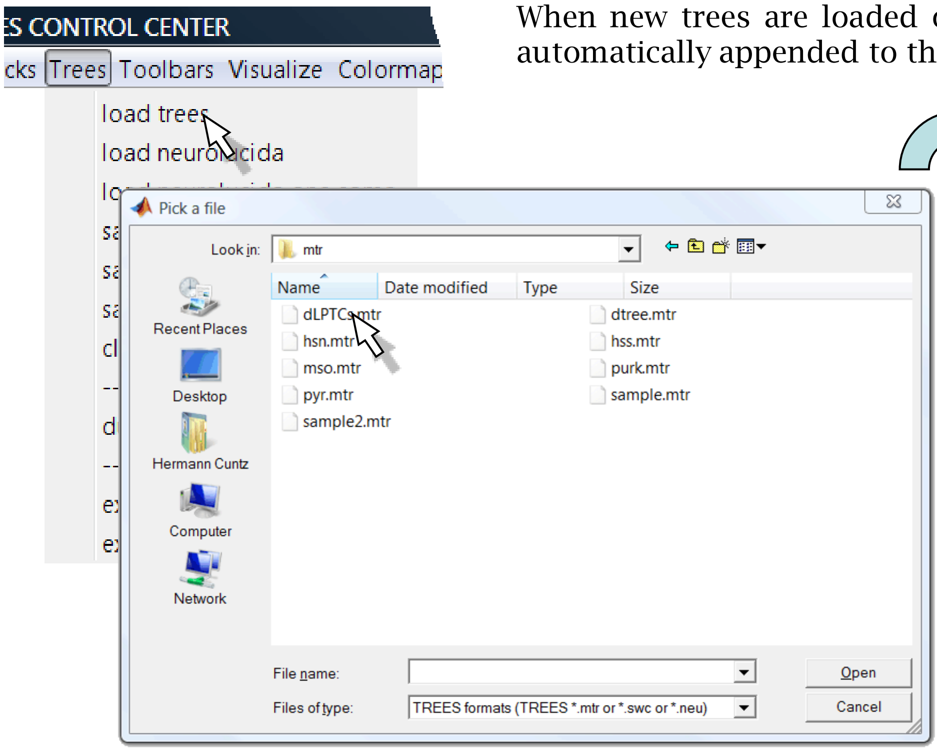

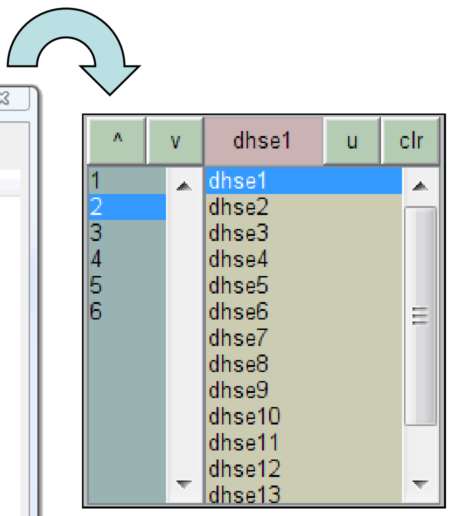

the ged_ panel: global edit for trees

The ged_ panel is useful to rearrange the morphologies of different trees in relation to other sets of trees. Either interactively or indirectly using the buttons on the ged_ panel, trees can be scaled, rotated, flipped or moved and their cable diameter can be globally increased or reduced. All these functions are applicable to all trees in a group directly if the „G“ toggle is switched on. Apart from the edit modes, two further features of the ged_ panel are explained in more detail in the following page.

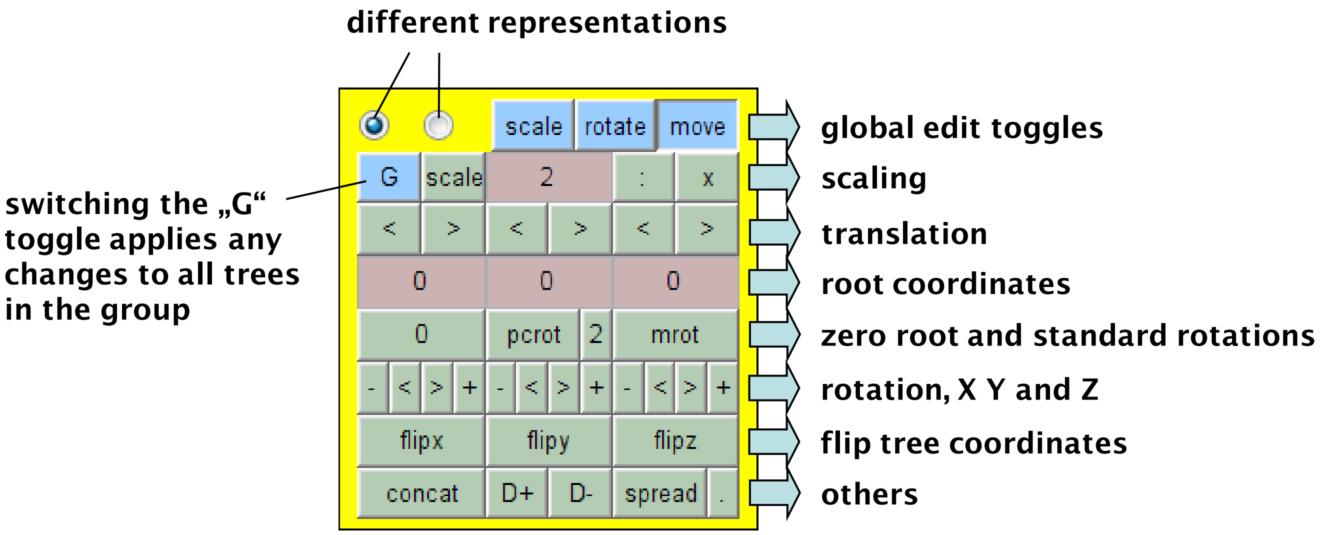

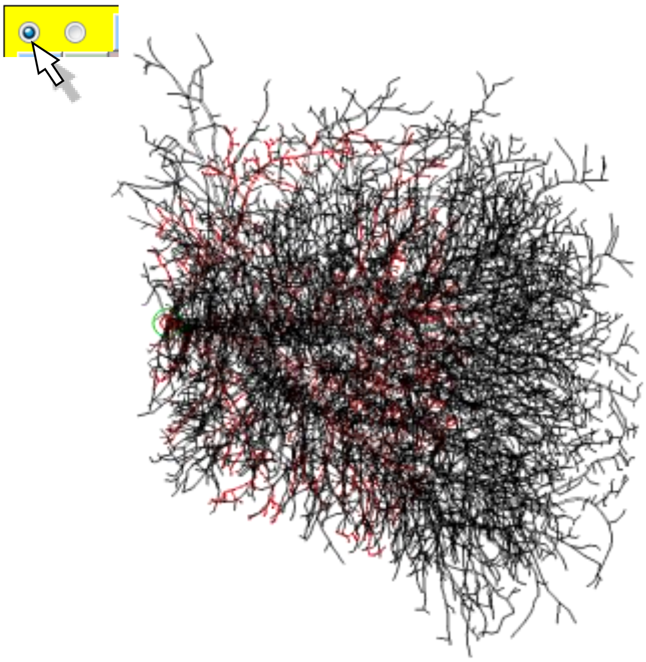

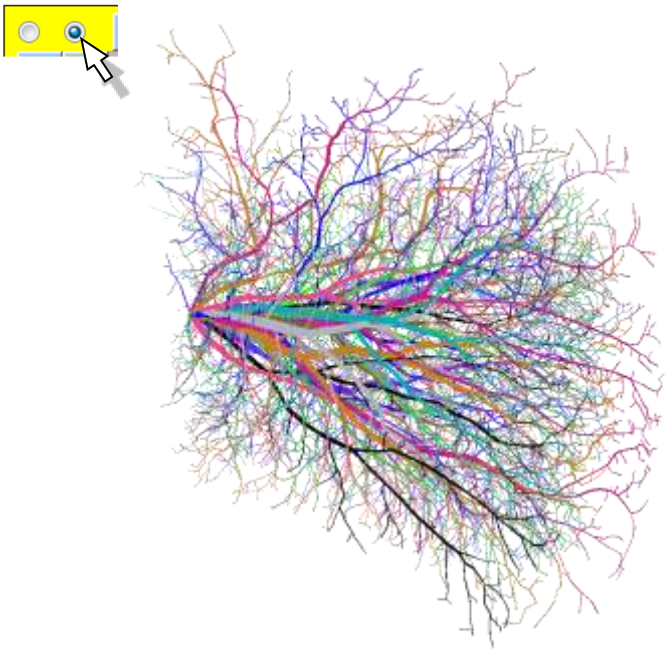

Simple black 2D-patches or full rainbow colour 3D cylinder models of all cells in a group can be displayed using the first or second radio button respectively. For huge groups of trees this can become slow.

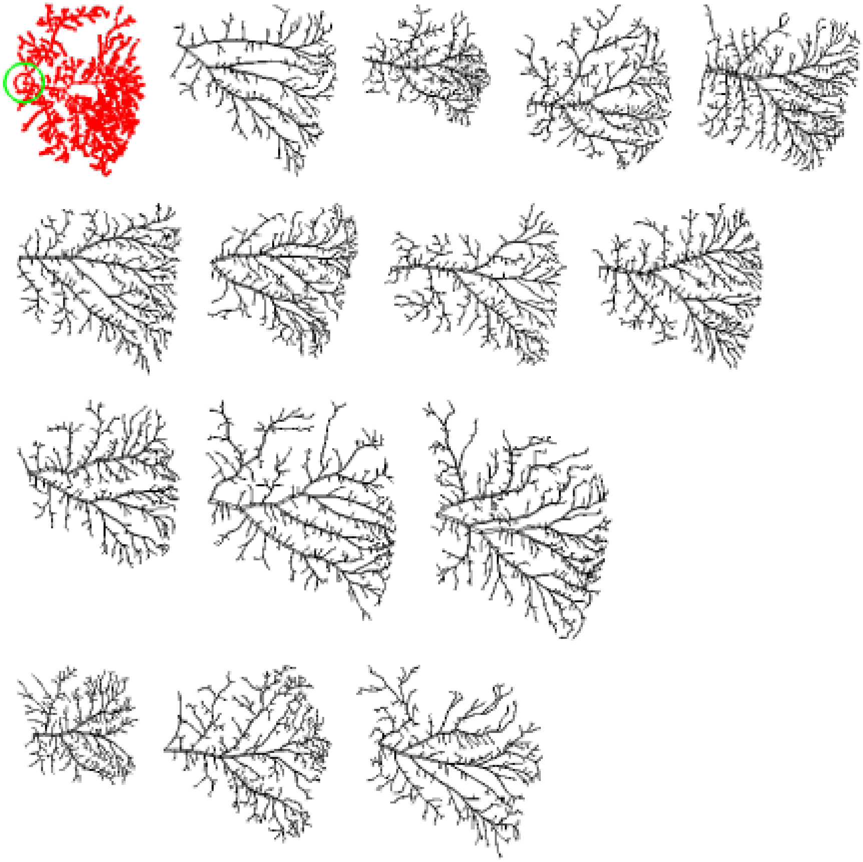

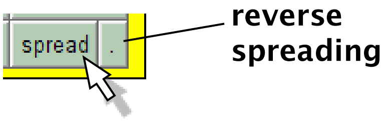

With the „spread“ button, trees of one group are spread out in 2D for a better overview (see „spread_tree“). The process is reversible as long as the tree order in the group is unchanged. To set all root coordinates to zero, first toggle „G“ and then press „0“ button.

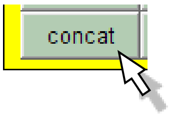

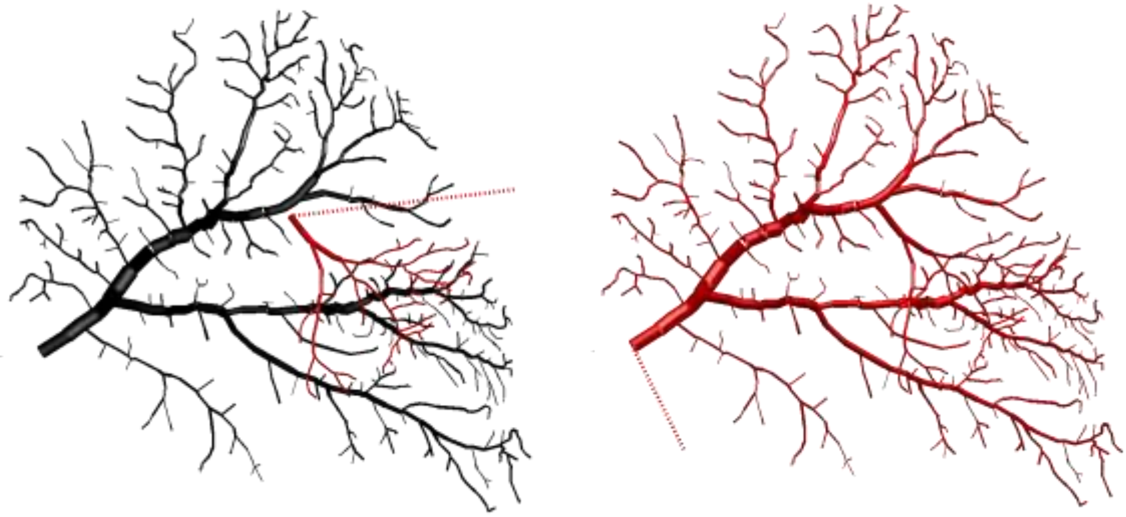

By pressing the „concat“ button, a tree in a group is connected to the immediately preceding tree it (see „cat_tree“). The tree is connected at its root to the node of its connection partner which is closest. Arrange the trees ahead of time using the different buttons of the ged_ panel or the edit mode. A sub-tree can be for example cut out using the mtr_ panel function „discon“.

The GUI the ged_ panel

lbars | Visualize | Colormaps | Print

Menu item (Visualize)	Function
statistics	compare statistics between groups of trees
------	
dA	display adjacency matrix
dendrogram	display dendrogram
sholl	2D sholl plot
3D plot of sholl	3D representation of sholl plot
sse	electrotonic signature
------	
send away as x3d o	export tree to x3d with spheres
send away as x3d	export tree to x3d
send to neuron	export NEURON
------	
send to povray	export to POVRay
send group to povray ▸	brainbow / GFP / parchment / black and white / alien / glass on cork / red coral
send all to povray - brainbow	
------	
set cylinder resolution ▸	

statistics

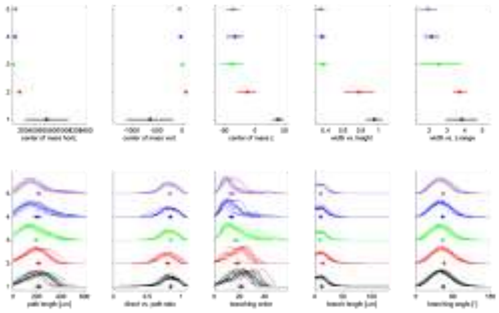

adjacency matrix

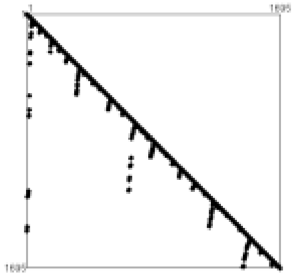

dendrogram

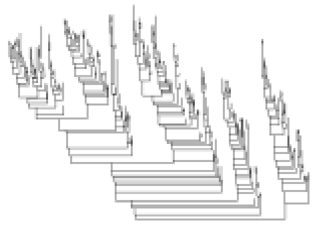

export to default x3d plotter

http://www.bitmanagement.com/

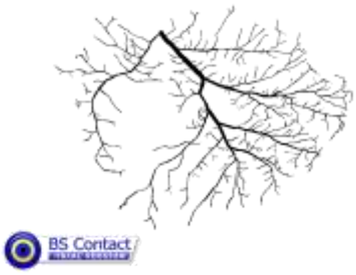

export to POVRay (http://www.povray.org/)

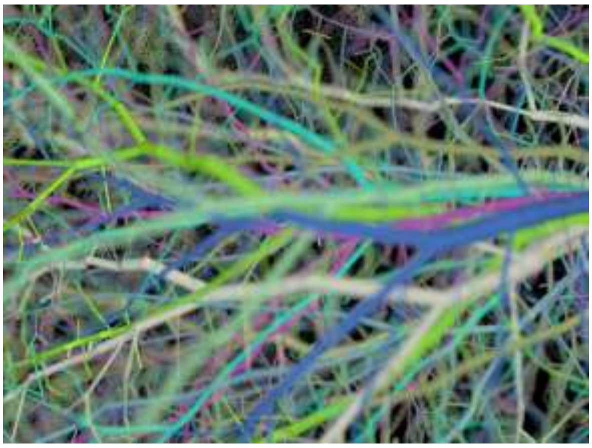

sholl plot

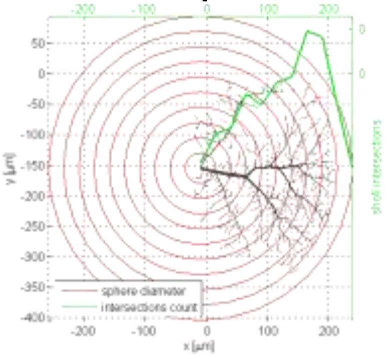

export to NEURON

http://www.neuron.yale.edu/neuron/

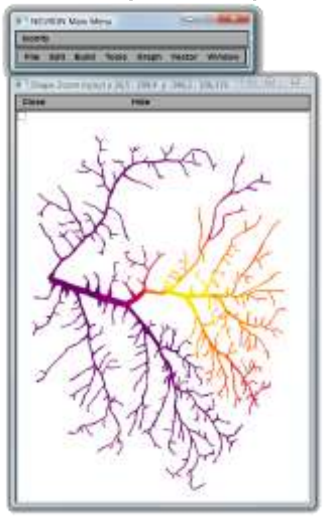

electrotonic signature

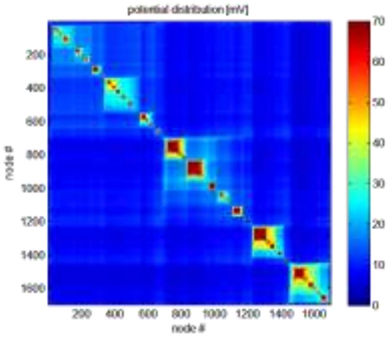

sholl 3D plot

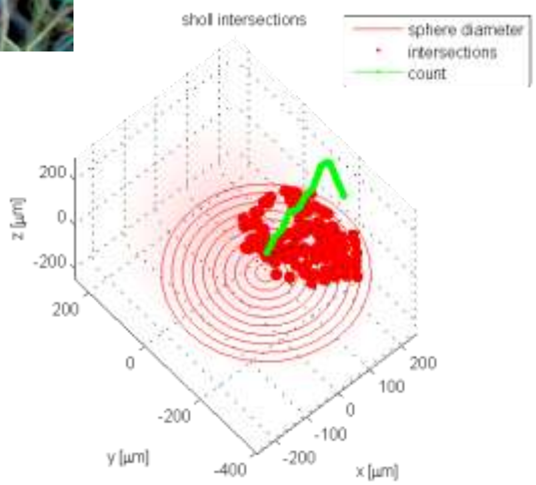

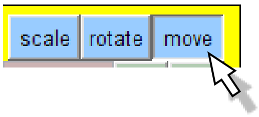

ged_ edit mode
With the ged_ panel, different trees can be arranged in relation to each other by scaling, rotating and moving them individually or together. In the edit mode this is done interactively using the mouse movements. Take for example a tree of an HSN (black) and an HSE (red) tangential cell of the fly and switch to the edit mode. By toggling between the three submodes “scale”, “rotate” and “move” the trees can be arranged to place them together as if they were reconstrucitons from the same Lobula plate. The red dashed edit line indicates which tree is closest to the cursor. By double click of the mouse that tree can be activated. Switching between trees of the active group is also possible with ctrl [a] and [d] keys. The implementation of an interactive rotation is not great yet.

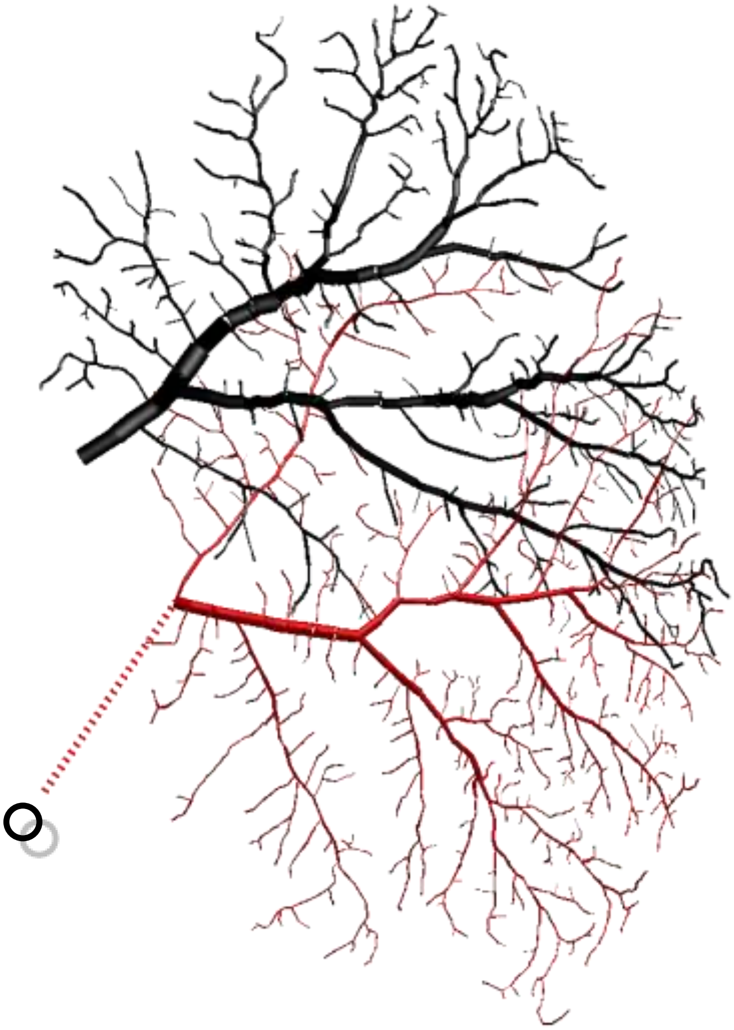

the slt_ panel: node selector

The slt_ panel goes hand in hand with the mtr_ panel to browse nodes of that active tree or analyse their properties and change region attributing. The first popup allows to attribute values to nodes individually by means of one of the typical *Nx1* vectors. If a vector is selected here the values are mapped onto the colours of the active tree in the mtr_ panel or the plot functions in the plt_ panel (see later) or they are used for the morphing function in the mtr_ panel. More sophisticated *Nx1* vectors can be computed using one of the meta-functions (see „Pvec_tree“, „child_tree“, „ratio_tree“ and „asym_tree“). For example the metric path length is the combination of „Pvec“ and „LEN“.

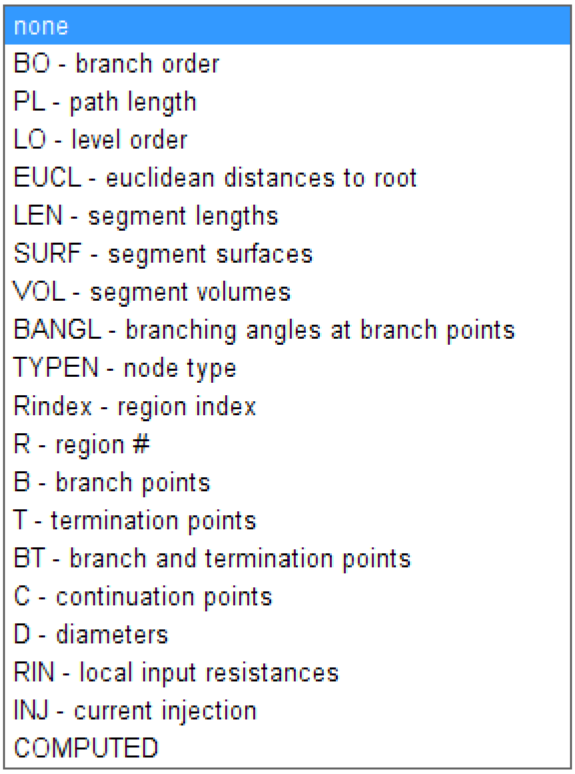

select or compute *Nx1* vector attributing one value per node

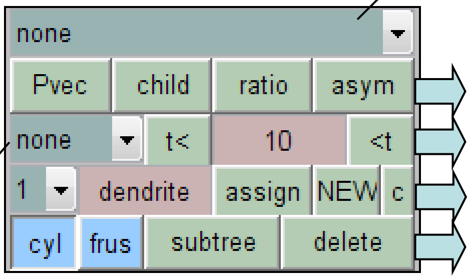

apply meta-functions

select nodes

change region assignement

other

select subset of nodes

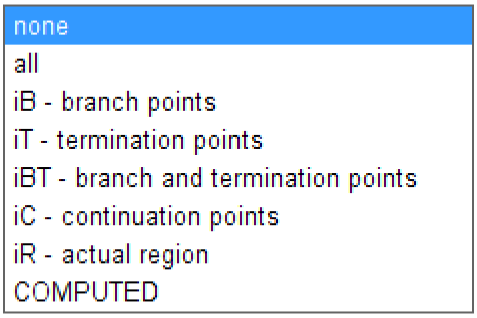

An index (subset) to the nodes can be selected here as well. This can be done directly using one of the options (e.g. all branch points with „iB“) or by thresholding the selected *Nx1* vector („t<„ and „<t“) or by selecting a subtree to the last active node of the mtr_ panel, or... by using the edit2 mode of the mtr_ panel, then it becomes very complicated. The selected nodes can be deleted directly or attributed to a new region. Selected nodes are indicated in black on the mtr_ panel plot of the active tree. plt_ panel plotting functions, spines allocation or current injections will be restricted to the selected index of nodes.

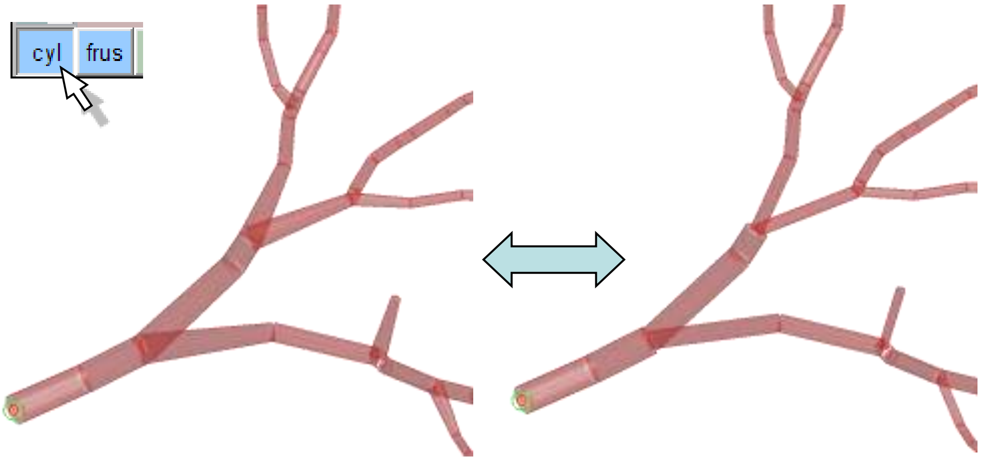

In the TREES toolbox, the conventional representation of a tree is as a set of cylinders, where the diameter depends on the daughter node. However, this can easily be altered by adding a „.frustum = 1“ field to the tree structure. In the GUI this is toggled in the slt_ panel as shown here. Differences in total volume or membrane surfaces of more than 10% can occur when switching. Note that POV-Ray and x3d graphical outputs do not support frustum, while NEURON to our knowledge supports only frustum.

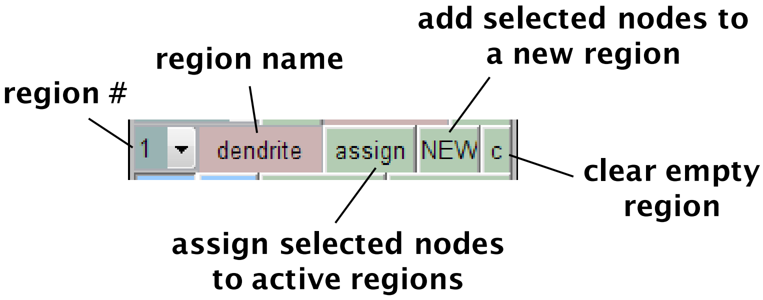

Region assignment is a bit complicated. To visualize which nodes belong to the selected region use the „iR“ selection in the node selector. The popup allows to switch from one region to another. The edit-select mode in the mtr_ panel allows together with the node selector of the slt_ panel to chose a set of specific nodes. These can then be assigned to the currently active region or form together a new region by the control „NEW“. When this happens they are extracted from the other region sets. In some cases regions remain empty after such a procedure: The „c“ control is there to clear such regions. In some select cases still deletion of nodes might result into problematic region assignements and this bug was not identified yet.

The GUI the ele_ panel

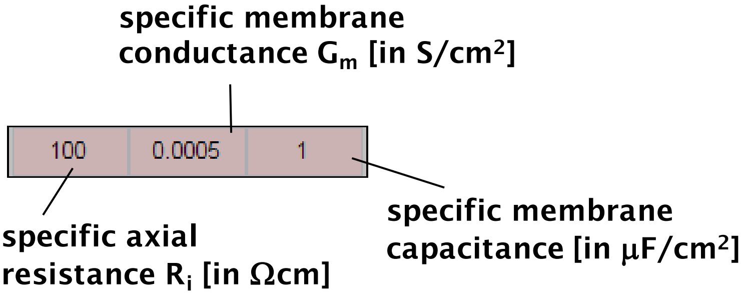

the ele_ panel: electrotonics

The ele_ panel is to be expanded dramatically in future versions. For now the three passive electrotonic properties can be set here. They are used for current injection and local input resistance measurements in the slt_ panel and are exported to NEURON.

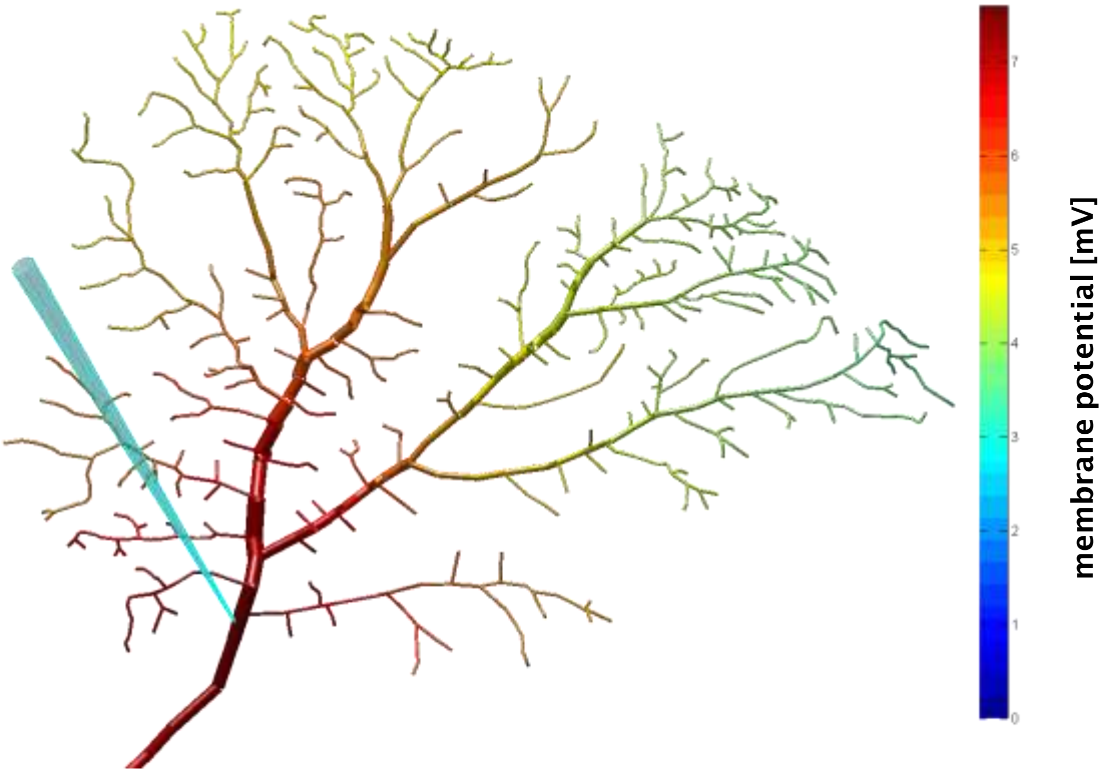

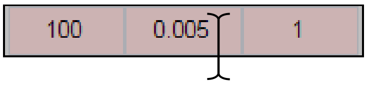

Change these passive properties or edit the tree to see electrotonic changes live.

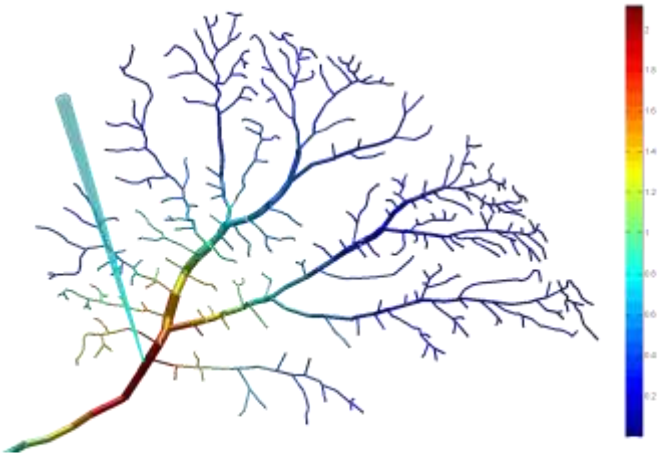

the plt_ panel: graphical objects

The plt_ panel offers a number of ways to create graphical objects relating to the active tree, its nodes and the *Nx1* vector values and node index selection determined in the slt_ panel. The various functions result in objects which are addressed as handle packages (see below). Each new handle package is added as a new entity in the popup and can be edited in various ways. „cla“ of the vis_ panel deletes all plt_ panel handles at once!

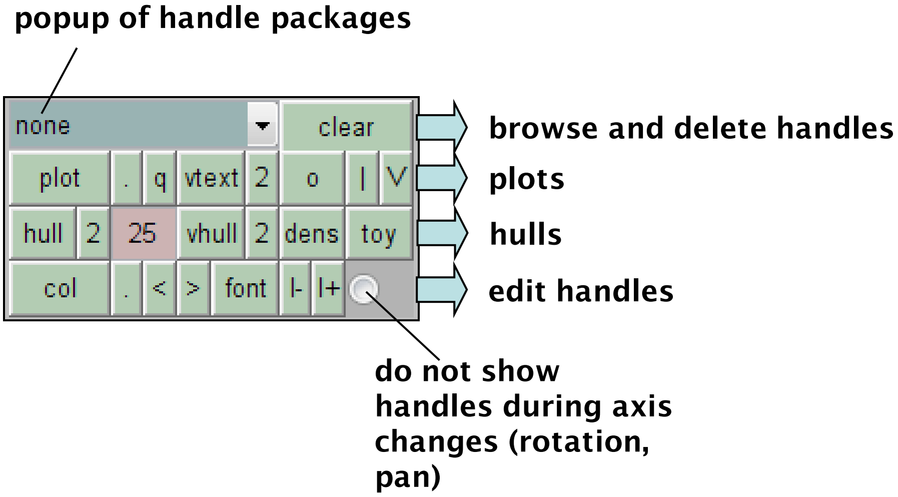

A handle for example produced by plot_tree has a number of attributes accessed by the Matlab function „get“:

```
>> HP = plot_tree (sample_tree)

>> get (HP)
```

AlphaDataMapping = scaled
Annotation = [(1 by 1) hg.Annotation array]
CData = [(4 by 1576 by 3) double array]
CDataMapping = scaled
DisplayName =
FaceVertexAlphaData = []
FaceVertexCData = [(3152 by 3) double array]
EdgeAlpha = [1]
EdgeColor = [0 0 0]
FaceAlpha = [1]
FaceColor = interp
Faces = [(1576 by 4) double array]
LineStyle = none
LineWidth = [0.5]
Marker = none
...

Change one of the attributes with the Matlab function „set“. For example make render the handle red:

```
>> set(HP,'facecolor',[1 0 0])
```

The GUI **the plt_ panel**

The examples on this page and the next are different plotting options provided by the TREES toolbox. For the purpose of illustration, the branch order was mapped onto the nodes (BO *Nx1* vector in the slt_ panel) and a sub-tree was selected (using the subtree function of the slt_ panel). Each plotting function creates a handle package. These handle packages can then be browsed post hoc, altered or deleted.

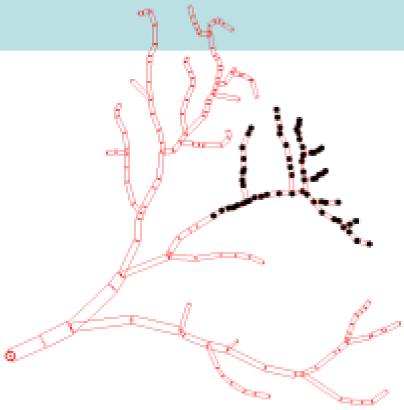

plot

maps BO on colour but disregards sub-tree selection.

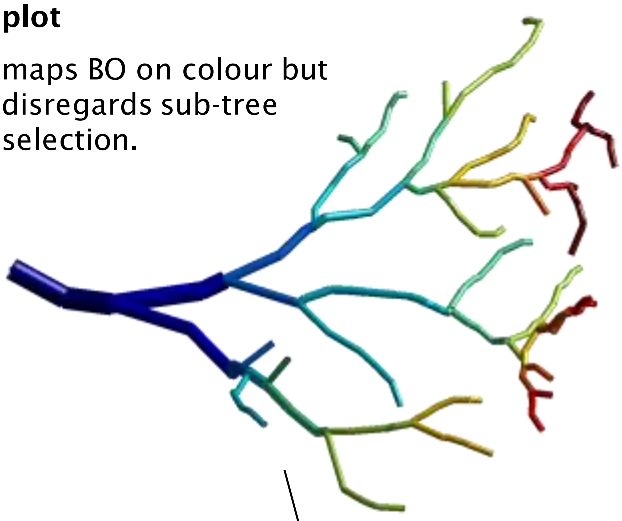

text plot

Plots the BO values as 2D or 3D text and maps BO on colour. Covers only selected indices.

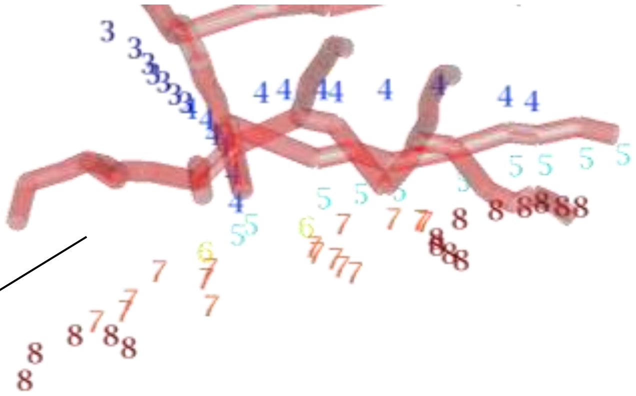

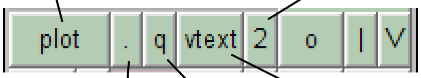

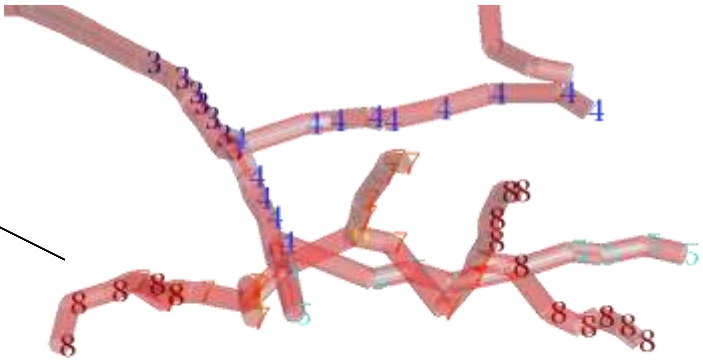

patch plot

2D patches in XY-plane. Maps BO on colour plots only selected sub-tree.

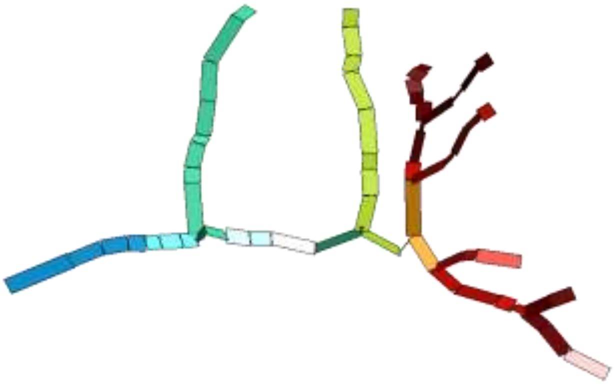

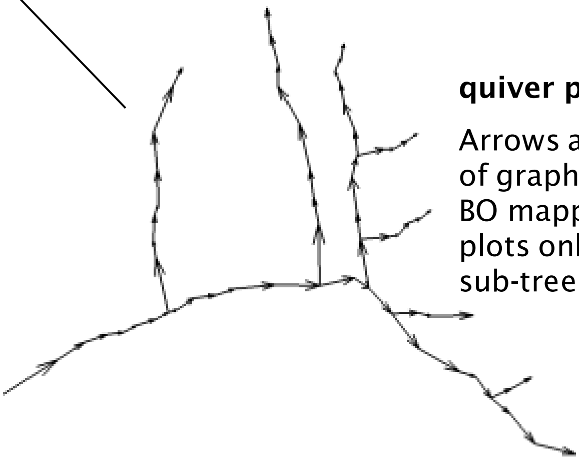

quiver plot

Arrows along edges of graph. Disregards BO mapping, but plots only selected sub-tree.

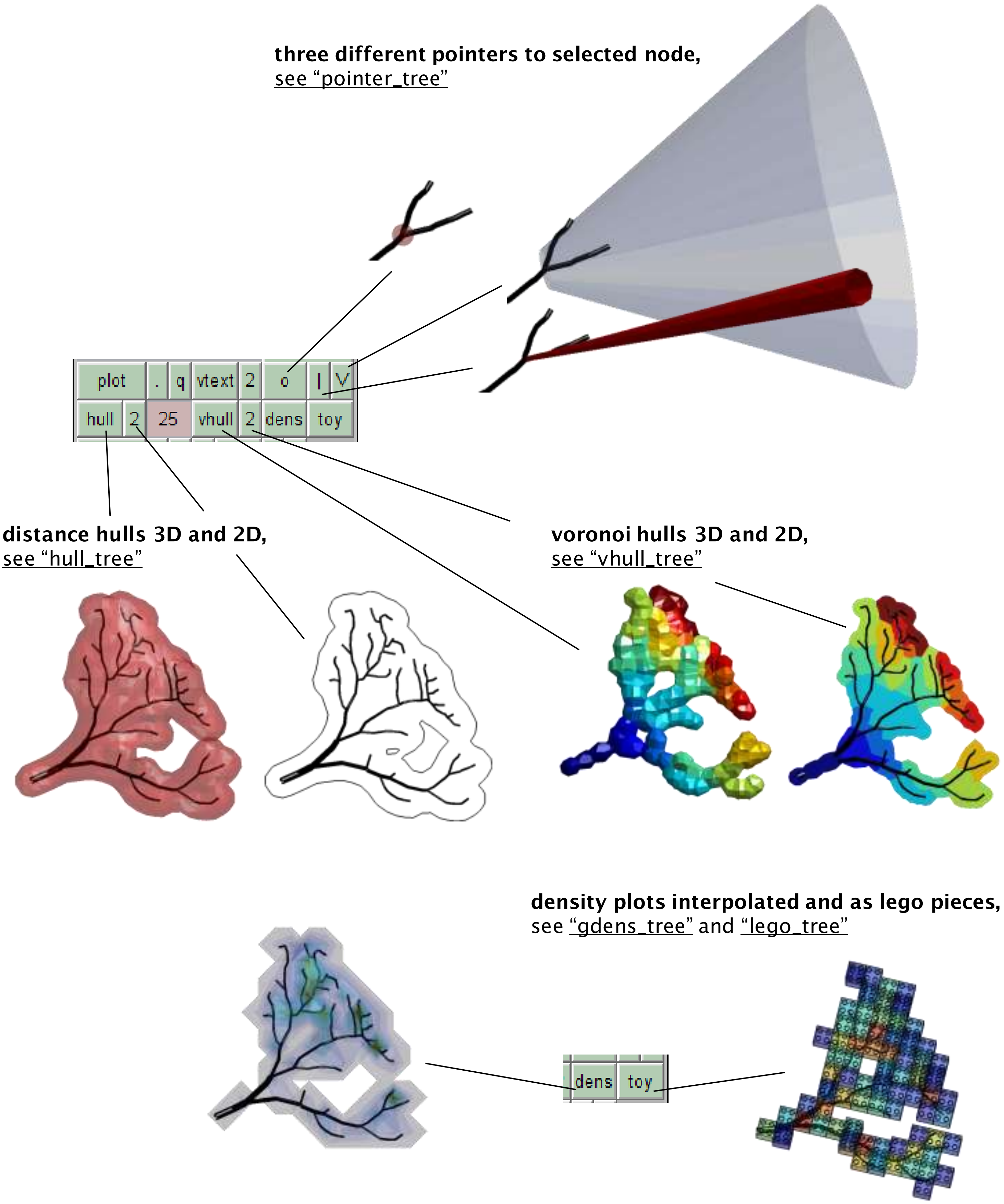
three different pointers to selected node,
see "pointer_tree"
plot
.
q
vtext
2
o
|
V
hull
2
25
vhull
2
dens
toy
distance hulls 3D and 2D,
see "hull_tree"
voronoi hulls 3D and 2D,
see "vhull_tree"
density plots interpolated and as lego pieces,
see "gdens_tree" and "lego_tree"
dens
toy

The examples on the previous pages produce handle packages with different attributes. With the plt_ panel some of these attributes can be altered in the active handle package in those handles in which the corresponding attributes are available.

select font using a GUI

vtext handle font attributes can be altered here.

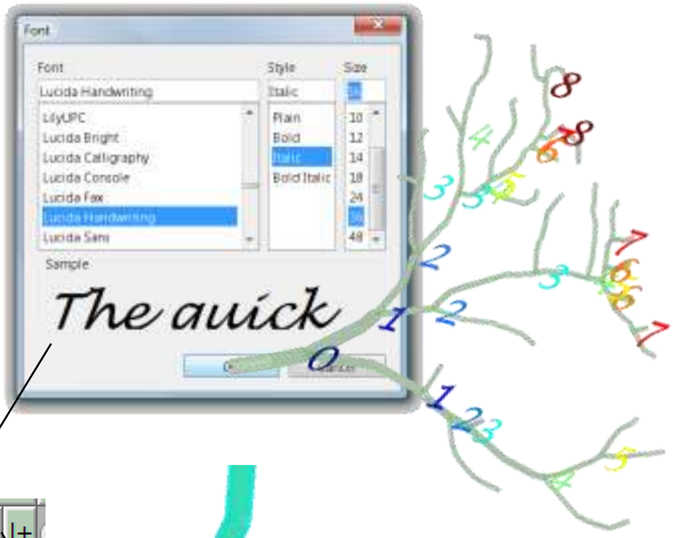

patch colour

select your favourite colour using a GUI

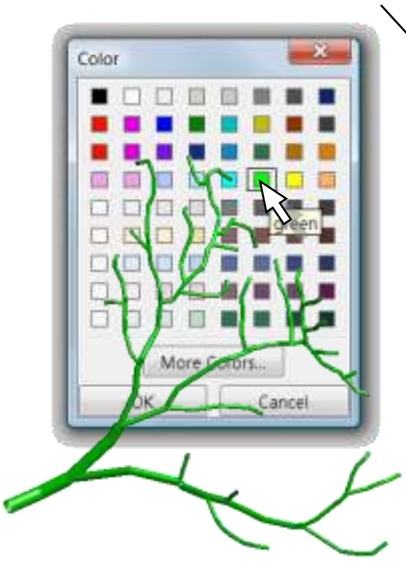

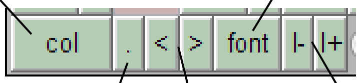

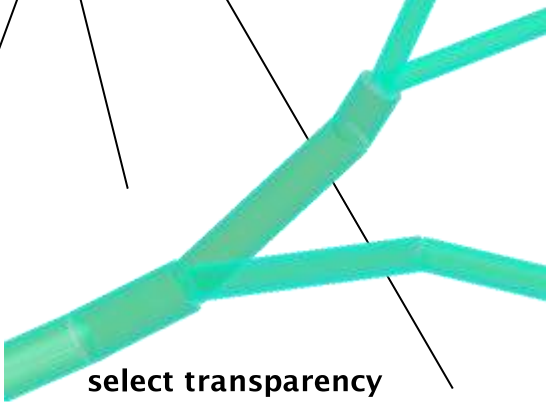

select transparency

increase or decrease transparency of patches and lines.

line colour

add lines to patches and select their colour.

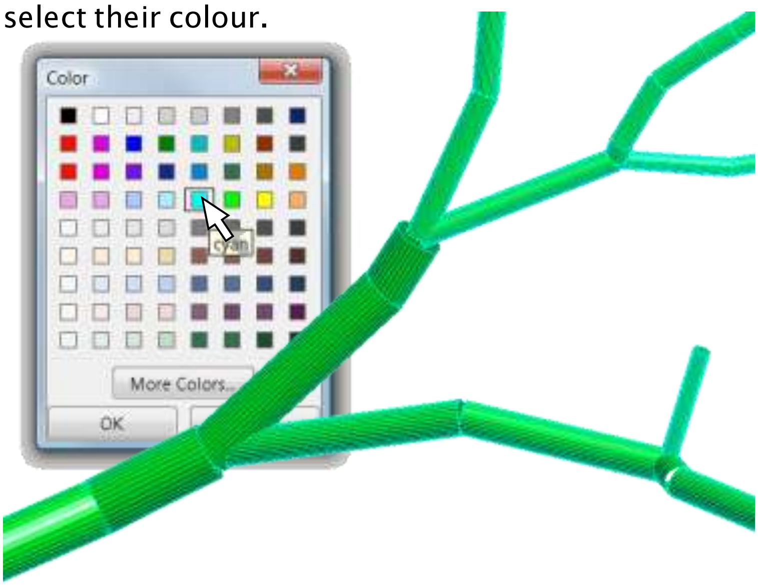

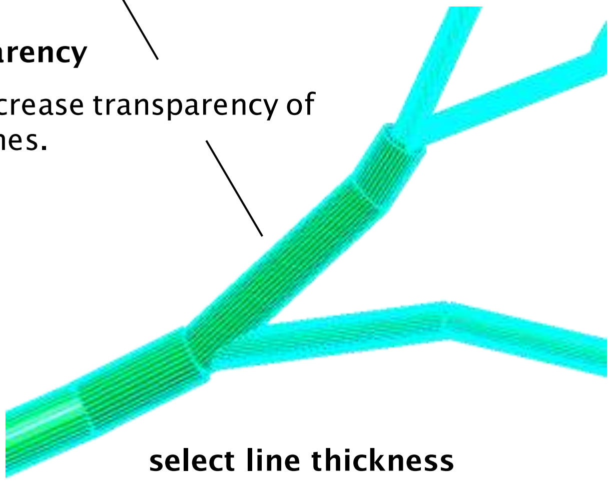

select line thickness

increase or decrease linewidth attribute of handles with lines.
